# The Transcription Factor SomA Synchronously Regulates Biofilm Formation and Cell Wall Homeostasis in *Aspergillus fumigatus*

**DOI:** 10.1128/mBio.02329-20

**Published:** 2020-11-10

**Authors:** Yuan Chen, Francois Le Mauff, Yan Wang, Ruiyang Lu, Donald C. Sheppard, Ling Lu, Shizhu Zhang

**Affiliations:** a Jiangsu Key Laboratory for Microbes and Functional Genomics, Jiangsu Engineering and Technology Research Center for Microbiology, College of Life Sciences, Nanjing Normal University, Nanjing, China; b Departments of Medicine and of Microbiology and Immunology, McGill University, Montréal, Canada; c Infectious Diseases and Immunity in Global Health Program, Research Institute of the McGill University Health Centre, Montreal, Canada; d McGill Interdisciplinary Initiative in Infection and Immunity (MI4), Montréal, Canada; University of Georgia

**Keywords:** polysaccharides, biofilm, cell wall, fungal pathogen, *Aspergillus fumigatus*

## Abstract

Polysaccharides are key components of both the fungal cell wall and biofilm matrix. Despite having distinct assembly and regulation pathways, matrix exopolysaccharide and cell wall polysaccharides share common substrates and intermediates in their biosynthetic pathways. It is not clear, however, if the biosynthetic pathways governing the production of these polysaccharides are cooperatively regulated. Here, we demonstrate that cell wall stress promotes production of the exopolysaccharide galactosaminogalactan (GAG)-depend biofilm formation in the major fungal pathogen of humans Aspergillus fumigatus and that the transcription factor SomA plays a crucial role in mediating this process. A core set of SomA target genes were identified by transcriptome sequencing and chromatin immunoprecipitation coupled to sequencing (ChIP-Seq). We identified a novel SomA-binding site in the promoter regions of GAG biosynthetic genes *agd3* and *ega3*, as well as its regulators *medA* and *stuA*. Strikingly, this SomA-binding site was also found in the upstream regions of genes encoding the cell wall stress sensors, chitin synthases, and β-1,3-glucan synthase. Thus, SomA plays a direct regulation of both GAG and cell wall polysaccharide biosynthesis. Consistent with these findings, SomA is required for the maintenance of normal cell wall architecture and compositions in addition to its function in biofilm development. Moreover, SomA was found to globally regulate glucose uptake and utilization, as well as amino sugar and nucleotide sugar metabolism, which provides precursors for polysaccharide synthesis. Collectively, our work provides insight into fungal adaptive mechanisms in response to cell wall stress where biofilm formation and cell wall homeostasis were synchronously regulated.

## Abstract (precis)

The cell wall is essential for fungal viability and is absent from human hosts; thus, drugs disrupting cell wall biosynthesis have gained more attention. Caspofungin is a member of a new class of clinically approved echinocandin drugs to treat invasive aspergillosis by blocking β-1,3-glucan synthase, thus damaging the fungal cell wall. Here, we demonstrate that caspofungin and other cell wall stressors can induce galactosaminogalactan (GAG)-dependent biofilm formation in the human pathogen Aspergillus fumigatus. We further identified SomA as a master transcription factor playing a dual role in both biofilm formation and cell wall homeostasis. SomA plays this dual role by direct binding to a conserved motif upstream of GAG biosynthetic genes and genes involved in cell wall stress sensors, chitin synthases, and β-1,3-glucan synthase. Collectively, these findings reveal a transcriptional control pathway that integrates biofilm formation and cell wall homeostasis and suggest SomA as an attractive target for antifungal drug development.

## INTRODUCTION

Biofilms are organized communities of surface-associated microorganisms embedded in a polymeric extracellular matrix. They are common microbial growth forms in nature and during human infection (1, 2). Emerging evidence suggests that pathogenic fungi produce biofilms during infection, where they play a crucial role in mediating adherence to both host tissues and biomedical devices and provide protection from host immune defenses and antifungal therapy (3).

Aspergillus fumigatus is a common opportunistic mold that causes invasive infections in immunosuppressed patients (4). One strategy used by A. fumigatus to establish and maintain infection is the production of biofilms within pulmonary tissues (5, 6). Recent studies have established a key role for exopolysaccharide galactosaminogalactan (GAG) in biofilm formation of A. fumigatus. GAG is a cationic linear heteropolymer composed of α-1,4-linked galactose, *N*-acetylgalactosamine (GalNAc) and galactosamine (GalN) (7–9). GAG binds to the surface of hyphae via charge-charge interactions, resulting in a polysaccharide sheath that covers the hyphae. GAG is also secreted and is an important component of the biofilm extracellular matrix in *Aspergillus* species (10). Degrading GAG within A. fumigatus biofilms with the GAG-specific hydrolase Sph3 significantly enhances the activity of antifungal agents, highlighting the importance of GAG in antifungal resistance (11).

A cluster of five genes on chromosome 3 is predicted to encode the enzymes required for the synthesis of GAG (10). These genes encode a glucose 4-epimerase (*uge3*) (8, 9), a secreted polysaccharide deacetylase (*agd3*) (10, 12), a putative transmembrane glycosyltransferase (*gtb3*), and two glycoside hydrolases (*ega3* and *sph3*) (13, 14). The fungal developmental regulators MedA and StuA and the transcription factor SomA positively regulate expression of the *uge3* gene within the GAG biosynthetic cluster (8, 15), while the Lim-domain binding protein PtaB regulates expression of both *uge3* and *agd3* expression (16). SomA forms a complex with PtaB to regulate the expression of *medA* and *stuA*, suggesting the SomA/PtaB complex acts upstream of MedA and StuA (17). However, the mechanisms underlying this regulation are not fully defined. Factors governing expression of other genes within the GAG biosynthetic cluster also remain unknown.

In addition to their role in biofilm matrix, polysaccharides are also the main components of the fungal cell wall. The cell wall of A. fumigatus consists of linear and branched polysaccharides, including α-glucans, β-glucans, chitin, and galactomannans (18–20). GAG shares some common substrates and intermediates with the synthesis of these cell wall polysaccharides. The synthesis of GAG involves the nucleotide sugars UDP-galactose and UDP-GalNAc as the substrates. UDP-galactose can also be converted to UDP-galactofuranose (UDP-Gal*f*) by UDP-galactofuranose mutase (Ugm1). UDP-Gal*f* is a key substrate for the synthesis of the cell wall polysaccharide galactomannan (21). Deletion of *ugm1* results in increased production of GAG, suggesting a link between these two pathways (8). Moreover, UDP-GlcNAc, required for chitin synthesis, is converted to UDP-GalNAc by Uge3 for the synthesis of GAG (9). An increase in the GlcNAc content of the cell wall of the *uge3* deletion mutant was observed, suggesting that the increased availability of UDP-GlcNAc leads to increased chitin synthesis in this mutant (8). Collectively, these observations suggest a link between GAG and other cell wall polysaccharide biosynthetic pathways. However, the signaling pathways that underlie these connections remain unknown.

The cell wall stress response has been shown to link cell wall polysaccharide biosynthetic pathways in many fungi (22). Moreover, the studies in the Candida albicans revealed a partial link between cell wall integrity pathway and biofilm matrix production (23). We therefore hypothesized that studying the effects of cell wall stress on biofilm formation would reveal the links between this process and cell wall homeostasis. In this study, we demonstrate that cell wall stress promotes GAG-mediated biofilm production via transcription factor SomA. Expression profiling and full genome chromatin immunoprecipitation (ChIP) revealed that SomA regulates the expression of genes encoding GAG biosynthesis and cell wall homeostasis via distinct pathways. Our work provides insight into fungal adaptive mechanisms in response to cell wall stress and sheds light on a regulatory circuit that couples biofilm formation and cell wall homeostasis.

## RESULTS

### Cell wall stress promotes GAG-mediated biofilm formation.

**FIG 1:**
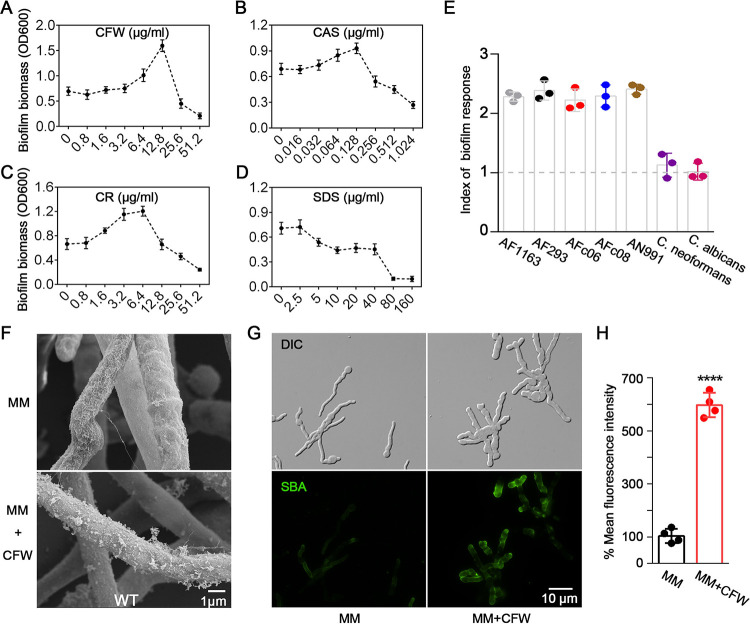
Cell wall stress promotes GAG-mediated biofilm formation in *Aspergillus*. (A to D) Crystal violet staining of 24-h biofilms of A. fumigatus parental wild-type A1160^C^ (WT) grown in the presence of a range of concentrations of calcofluor white (CFW) (A), caspofungin (CAS) (B), Congo red (CR) (C), or sodium dodecyl sulfate (SDS) (D). Standard deviations represent the averages from three independent biological experiments, each with six technical repetitions. (E) Index of fungal biofilm formation in response to CFW cell wall stress. The index was calculated by dividing the normalized crystal violet staining signal of CFW-treated biofilms by that of untreated biofilms. All data were performed by three independent biological experiments, each with six technical repetitions. (F) Scanning electron micrographs of hyphae of A. fumigatus WT after 24 h of growth with or without 12.8 μg/ml CFW. Scale bar, 1 μm. (G) Representative images of A. fumigatus WT hyphae stained with a GAG-specific fluorescein-tagged soybean agglutinin lectin (SBA-FITC) after 8 h of growth with or without 10 μg/ml CFW. Scale bar, 10 μm. (H) Quantification of the mean fluorescence intensity (MFI) of A. fumigatus WT hyphae grown under the same conditions described for panel G. The data are presented as the percentages of the MFI of the WT strain grown in MM, and the standard deviations represent averages from four independent biological samples, each with five hyphal sections measured (****, *P* < 0.0001).

To test the impact of cell wall stress on the A. fumigatus biofilm formation, wild-type (WT) hyphae were grown in the presence of cell wall stressors, including the chitin-binding agents calcofluor white (CFW) and Congo red (CR), the ionic detergent sodium dodecyl sulfate (SDS), and the β-1,3-glucan synthase inhibitor caspofungin (CAS). At high concentrations, all four agents inhibited the growth of A. fumigatus (see Fig. S1A to D in the supplemental material). However, at lower concentrations, CFW, CR, and CAS exposure resulted in an increase in biofilm formation (Fig. 1A to D). This effect was most marked following CFW exposure, with a 2-fold increase in adherent biofilm biomass when hyphae were grown in the presence of 12.8 μg/ml CFW. Cell wall stress-induced biofilm formation was observed reproducibly among A. fumigatus isolates, including the common laboratory strain AF293 and two clinical isolates, AFc06 and AFc08, as well as the nonpathogenic species Aspergillus nidulans (Fig. 1E), a species which is relatively biofilm deficient due to the low expression of *uge3* (24).

10.1128/mBio.02329-20.1 FIG S1 Growth of the WT under different concentrations cell wall stress. (A to D) The biomass of A. fumigatus A1160^C^ (WT) was measured at an absorbance of OD_600_ after culture statically in MM for 24 h in the presence of a series of concentrations of CFW (A), CAS (B), CR (C), and SDS (D). Standard deviations represent the average of two independent biological experiments with eight technical repetitions each. Download FIG S1, TIF file, 0.5 MB. Copyright © 2020 Chen et al. 2020 Chen et al. This content is distributed under the terms of the Creative Commons Attribution 4.0 International license.

Given the key role of GAG in A. fumigatus biofilm formation, we hypothesized that CFW-dependent augmentation of biofilm production might reflect an increase in GAG production. Consistent with this hypothesis, scanning electron microscopy (SEM) of the hyphal surface revealed that CFW exposure resulted in a significant increase in hyphal surface decorations and intercellular matrix, findings that have been associated with GAG production (Fig. 1F). These findings were also confirmed by GAG-specific fluorescein-tagged soybean agglutinin lectin (SBA-FITC) staining; the mean fluorescence intensity (MFI) on the hyphae was significantly increased by 5-fold when exposed to CFW (Fig. 1G and H). CFW exposure failed to enhance biofilm formation by the Δ*uge3* mutant strain (see Fig. S2). Moreover, CFW exposure had no effect on biofilm formation by the pathogenic yeasts Candida albicans and Cryptococcus neoformans (Fig. 1E), which lack the GAG biosynthetic gene cluster (10). Collectively, these data indicate that the cell wall stress induces GAG-mediated biofilm formation in *Aspergillus species*.

10.1128/mBio.02329-20.2 FIG S2 CFW stress could not induce biofilm formation of Δ*uge3*. Crystal violet staining of 24 h-old biofilms of the Δ*uge3* strain compared that of its parent A. fumigatus AF293 grown in the presence of a range of concentrations of CFW. Download FIG S2, TIF file, 0.1 MB. Copyright © 2020 Chen et al. 2020 Chen et al. This content is distributed under the terms of the Creative Commons Attribution 4.0 International license.

### SomA mediates cell wall stress-induced biofilm formation.

Previous work has demonstrated that GAG biosynthesis is regulated by the transcription factor SomA, which forms a complex with the Lim-binding domain protein PtaB to regulate the expression of the developmental regulatory proteins MedA and StuA (8, 16, 17). We therefore sought to determine whether stress-induced GAG-mediated biofilm formation was dependent on elements of this regulatory pathway. Since the deletion of *somA* was incapable of production of conidia, a *Tet-somA* strain was constructed by replacing its promoter region with the inducible Tet-On system (17, 25), which could conditionally express the *somA* gene upon addition of doxycycline to the medium.

**FIG 2:**
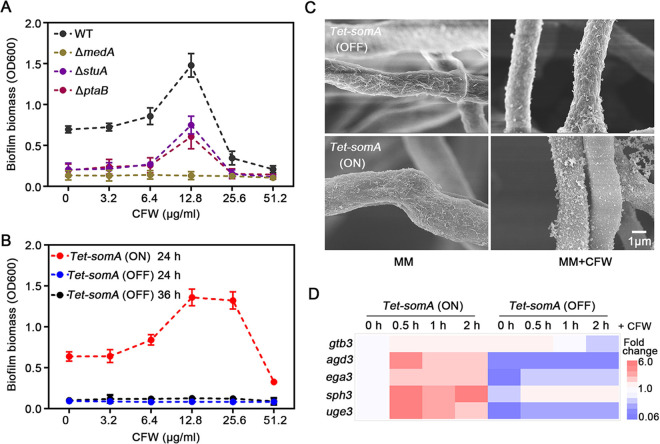
SomA mediates cell wall stress-induced biofilm formation. (A) Crystal violet staining of 24-h-old biofilms of the indicated mutant strains compared to that of the A. fumigatus parental wild-type A1160^C^ (WT) grown in the presence of a range of concentrations of CFW. (B) Crystal violet staining of 24- and 36-h-old biofilms of the conditional *Tet-somA* mutant grown in the presence of a range of concentrations of CFW. The expression of *somA* was induced by supplementing the culture media with 1 μg/ml doxycycline. (C) Scanning electron micrographs of the *Tet-somA* mutant hyphae in the presence or absence of 12.8 μg/ml CFW and/or 1 μg/ml doxycycline. Scale bar, 1 μm. (D) Heat map analysis of the relative expression level of the GAG gene cluster in the *Tet-somA* mutant (ON and OFF) under CFW cell wall stress condition. The *Tet-somA* mutant was first cultured in MM with or without doxycycline for 22 h. CFW at a final concentration of 100 μg/ml was then added to the media, and gene expressions were measured at 0.5, 1, and 2 h, respectively. Gene expression was normalized to the endogenous reference gene *tubA*, and expression was reported relative to the unstressed *Tet-somA* (ON) condition at 22 h growth. All the results were performed by three independent biological experiments.

Consistent with previous reports, the Δ*medA*, Δ*stuA*, Δ*ptaB*, and *Tet-somA* (OFF) strains all exhibited a severe defect in the biofilm formation under normal condition (Fig. 2A and B). Exposure to 12.8 μg/ml CFW dramatically enhanced biofilm production by the Δ*stuA* and Δ*ptaB* mutants (Fig. 2A) but not by the Δ*medA* and *Tet-somA* (OFF) mutants (Fig. 2A and B). These findings suggest that cell wall stress-induced biofilm formation is dependent on SomA and MedA. Since SomA acts as upstream of MedA, we focused on SomA in further study. Consistent with these findings, SEM demonstrated significantly reduced surface decoration and intercellular matrix in the *Tet-somA* (OFF) mutant, compared to both wild-type and *Tet-somA* (ON) strains under normal conditions (Fig. 2C). Strikingly, the CFW-mediated increase in GAG production that appeared in wild-type and *Tet-somA* (ON) strains was abolished in the *Tet-somA* (OFF) mutant (Fig. 2C; see also Fig. S3).

10.1128/mBio.02329-20.3 FIG S3 CFW stress could not induce GAG production of the *Tet-somA* (OFF) mutant. Representative images of the hyphae of *Tet-somA* (ON) and *Tet-somA* (OFF) strains stained with a GAG-specific fluorescein-tagged soybean agglutinin lectin (SBA-FITC) after 8 h of growth with or without 10 μg/ml CFW. Scale bar, 10 μm. Download FIG S3, TIF file, 0.5 MB. Copyright © 2020 Chen et al. 2020 Chen et al. This content is distributed under the terms of the Creative Commons Attribution 4.0 International license.

To confirm these findings, the effects of *somA* on the expression of GAG biosynthetic genes were assessed under both normal and cell wall stress conditions. Under normal conditions, the expression of three of five genes on the GAG biosynthetic cluster (*uge3*, *agd3*, and *ega3*) was dependent on SomA. When exposed to the cell wall stressor CFW, four of five genes on the GAG biosynthetic cluster (*uge3*, *agd3*, *sph3*, and *ega3*) were upregulated from 3- to 6-fold (Fig. 2D) in the *Tet-somA* (ON) strain. In contrast, only a minimal increase in *ega3* and *sph3* expression was observed in response to CFW exposure in the *Tet-somA* (OFF) mutant (Fig. 2D). Taken together, these data suggest that SomA is a key regulator of GAG production under both normal and cell wall stress conditions.

### SomA globally regulates glucose uptake, utilization, and amino sugar and nucleotide sugar metabolism.

**FIG 3:**
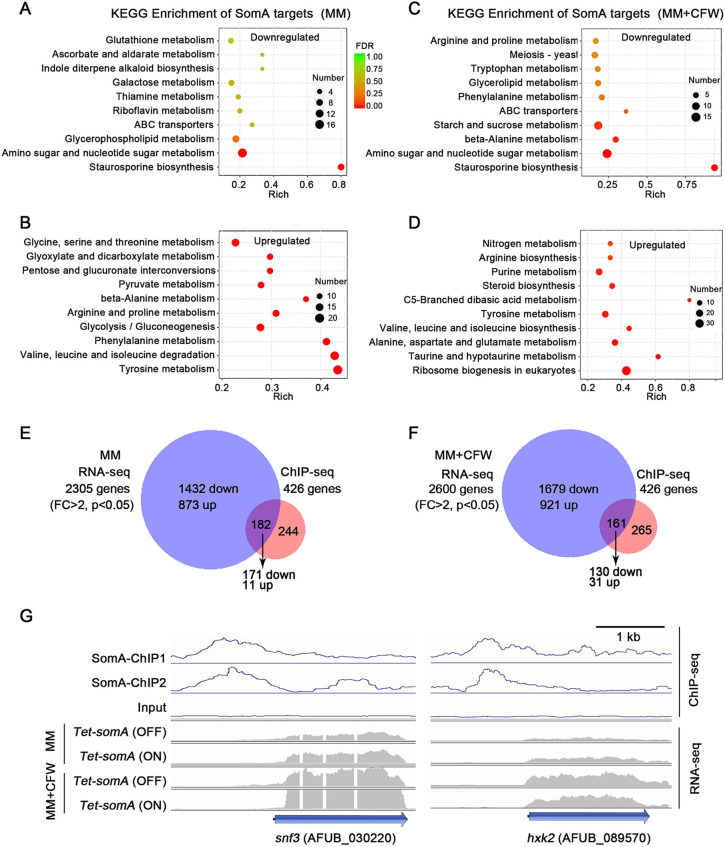
ChIP-seq and transcript analysis identify SomA target genes. (A and B) KEGG enrichment tables of downregulated (A) and upregulated (B) genes in the *Tet-somA* (OFF) versus *Tet-somA* (ON) mutant in the absence of CFW. (C and D) KEGG enrichment of downregulated (C) and upregulated (D) genes in the *Tet-somA* (OFF) mutant compared to the *Tet-somA* (ON) strain in the presence of CFW. False discovery rate values and gene numbers are represented using a gradient of color and bubble size, respectively. (E and F) Venn diagram of intersecting genes observed being differentially expressed (fold change > 2 and *P* < 0.05) in RNA-seq and genes identified as directly bound by SomA in ChIP-seq analyses in the absence (E) or in the presence (F) of CFW. (G) Genome browser images depicting the relative enrichment and transcript levels of two indicated glucose uptake and utilization genes based on ChIP-seq (blue) and RNA-seq (gray). ChIP1 and ChIP2 are two independent repetitions. Scale bar, 1 kb.

Given the importance of SomA in regulation of A. fumigatus biofilm formation and GAG biosynthesis, we carried out transcriptomic analysis (RNA-seq) of the *Tet-somA* strain under normal growth and cell wall stress conditions in the presence or absence of doxycycline. During growth in minimal media, 873 genes were upregulated (fold change, >2; *P* < 0.05) and 1,432 genes were downregulated in the *Tet-somA* (OFF) strain. In the presence of CFW, 921 genes were upregulated and 1,679 genes were downregulated in the *Tet-somA* (OFF) strain (see Table S1 in the supplemental material). To identify potential roles of SomA-dependent genes in specific fungal processes, we subjected these genes to pathway analysis using KEGG (see Table S2). Strikingly, transcripts whose abundance was directly linked to SomA expression were most significantly enriched in genes with functions in amino sugar and nucleotide sugar metabolism under both normal growth and cell wall stress conditions (Fig. 3A and C). In contrast, transcripts that were less abundant during conditions of SomA expression included tyrosine metabolism and glycolysis/gluconeogenesis (Fig. 3B and D). Overall, these findings suggest that SomA plays a key role in the control of carbon flux and the production of precursors for polysaccharide synthesis.

10.1128/mBio.02329-20.5 TABLE S1 RNA-seq analysis of differentially expressed genes. Download Table S1, XLS file, 1.8 MB. Copyright © 2020 Chen et al. 2020 Chen et al. This content is distributed under the terms of the Creative Commons Attribution 4.0 International license.

10.1128/mBio.02329-20.6 TABLE S2 RNA-seq KEGG enrichment. Download Table S2, XLSX file, 0.05 MB. Copyright © 2020 Chen et al. 2020 Chen et al. This content is distributed under the terms of the Creative Commons Attribution 4.0 International license.

To identify genes under direct transcriptional control of SomA, ChIP coupled to sequencing (ChIP-seq) was performed. The two biological ChIP-seq replicates identified 581 and 586 SomA-binding peak regions, respectively, sharing 476 common targets (see Table S3) corresponding to 426 genes (see Table S4) (*q* < 0.001, fold enrichment > 2). We further investigated the correlation between SomA occupancy and mRNA levels by comparing the ChIP-seq and the RNA-seq data sets. Of the 426 genes with associated SomA DNA binding, the transcripts of 182 genes (42.7%) and 161 genes (37.8%) were identified as SomA dependent by RNA-seq under normal and cell wall stress conditions, respectively (Fig. 3E and F). These results suggest that many of these SomA-dependent genes are likely indirectly regulated by SomA.

10.1128/mBio.02329-20.7 TABLE S3 ChIP-seq 476 common targets sequence. Download Table S3, DOC file, 0.2 MB. Copyright © 2020 Chen et al. 2020 Chen et al. This content is distributed under the terms of the Creative Commons Attribution 4.0 International license.

10.1128/mBio.02329-20.8 TABLE S4 ChIP high confident annotated and GO enrichment. Download Table S4, XLSX file, 0.1 MB. Copyright © 2020 Chen et al. 2020 Chen et al. This content is distributed under the terms of the Creative Commons Attribution 4.0 International license.

Among the 426 direct targets of SomA were two genes have potential roles in governing glucose uptake and utilization in A. fumigatus, including *snf3* (AFUB_030220) and *hxk2* (AFUB_089570) (Fig. 3G). The orthologue of *snf3* in S. cerevisiae encoding a major facilitator superfamily monosaccharide transporter responsible for glucose uptake (26). The orthologue of *hxk2* in S. cerevisiae encoding a putative hexokinase plays a role in glucose phosphorylation (27). Hexo/glucokinase-mediated glucose phosphorylation during the first step of glycolysis is crucial for fungal cell wall construction (28). The expression of *snf3* and *hxk2* were significantly decreased in the *Tet-somA* (OFF) strain compared to the *Tet-somA* (ON) strain both under normal and cell wall stress conditions (Fig. 3G). Collectively, these data suggest that SomA globally regulates glucose uptake, utilization, and amino sugar and nucleotide sugar metabolism.

### SomA binds to the promoters of genes related to GAG biosynthesis.

**FIG 4:**
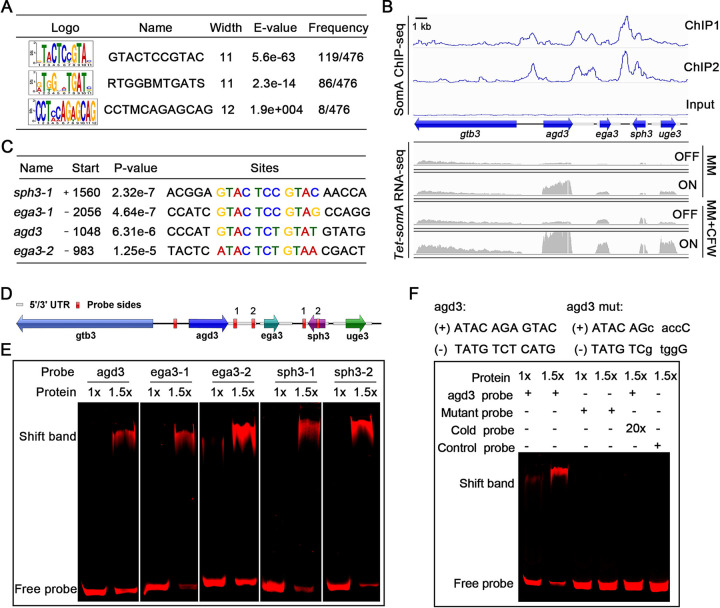
SomA directly regulates *ega3* and *agd3.* (A) Conserved motifs identified by SomA ChIP-seq. (B) Genome browser images depicting the relative enrichment and transcript levels of five indicated GAG cluster genes based on ChIP-seq (blue) and RNA-seq (gray). ChIP1 and ChIP2 indicate the results of two independent replicate experiments. Scale bar, 1 kb. (C) Summary table of the conserved motif found in the GAG cluster genes by ChIP-seq. The start column refers to the location and distance of the motif compared to the transcriptional site. (D) Schematic of GAG gene cluster. Red boxes represent EMSA probe sequence sites. (E) EMSA of SomA binding to Cy5-labeled promoter fragments of GAG cluster genes. (F) Specificity of EMSA binding to the promoter of *agd3.* The specificity of EMSA binding was confirmed by adding mutant probe, specific cold probe competitors (unlabeled probe), and control probe (unrelated probe).

The SomA-bound DNA motifs were identified using multiple expectation maximum for motif elicitation (MEME) of the identified peaks. The SomA DNA binding motif with the highest E value (5.6E–63) and frequency (119/476) is an 11-bp “GTACTCCGTAC” binding region (Fig. 4A).

Next, SomA binding sites that were identified in proximity to the GAG biosynthetic cluster genes and their known transcriptional regulators *medA* and *stuA* were examined in greater detail. Five SomA-binding peaks were identified in proximity to genes within the GAG biosynthetic cluster (Fig. 4B). Four of the five SomA-binding peaks regions contained the identified SomA-binding motif (Fig. 4C and D). The peak intensity map shows that SomA binding was significantly enriched at the region between *agd3* (1,048 bp upstream of the translational start site) and *gtb3* (1,299 bp upstream of the translational start site) which are in opposite orientations on the chromosome (Fig. 4B). Given that the RNA-seq studies demonstrated higher transcriptional levels of *agd3* but not *gtb3* in the *Tet-somA* (ON) strain, these data suggest that SomA directly regulates the expression of *agd3*. Two SomA-binding sites were found on the upstream regions of *ega3* (983 and 2,506 bp upstream of translational start site, respectively) (Fig. 4B). As with *agd3*, expression of *ega3* was positively regulated by SomA by RNA-seq, suggesting that SomA plays a direct role in regulating *ega3* expression. Two SomA-binding sites were found on the open reading frame (ORF) region and 3′ untranslated region (UTR) of *sph3* (Fig. 4B and C), respectively. However, the expression of *sph3* was not significantly different between the *Tet-somA* (ON) and (OFF) strains (Fig. 4B). Surprisingly, although the expression of *uge3* was dramatically reduced in the *Tet-somA* (OFF) strain, no SomA-binding sites were found in the promoter, the ORF region, or the UTRs of *uge3* (Fig. 4B and C), suggesting that SomA indirectly regulates *uge3* expression. Electrophoretic mobility shift assays (EMSAs) further confirmed the *in vitro* binding of SomA to GTACTCCGTAC motif-containing promoter fragments of *agd3* and *ega3* (Fig. 4E). Excess unlabeled DNA or mutation of the GTACTCCGTAT motif to GggtgCCGTAT in the *agd3* fragment blocked the interaction of SomA with the promoter fragments (Fig. 4F), highlighting the specificity of this protein-DNA interaction.

Previous studies have demonstrated a role for StuA and MedA in the regulation of GAG biosynthetic genes (8). RNA-seq and RT-qPCR demonstrated a reduced abundance of both *medA* and *stuA* mRNA in the *Tet-somA* (OFF) strain (see Fig. S4A and B in the supplemental material). Consistent with these findings, multiple SomA occupancy sites were found in the upstream of *medA* and *stuA* (see Fig. S4B), suggesting direct regulation of these factors by SomA. Collectively, these findings identify a role for both direct and indirect regulation of GAG biosynthetic genes by SomA.

10.1128/mBio.02329-20.4 FIG S4 SomA directly regulate the expression of *medA* and *stuA.* (A) Bar chart analysis of the transcript levels of *medA* and *stuA* genes determined using RT-qPCR in the WT, *Tet-somA* (OFF), and *Tet-somA* (ON) strains in MM for 24 h. Gene expression was normalized to the endogenous reference gene *tubA* and is presented as the fold change relative to the WT strain. All of the results were determined in three independent biological experiments. (**, *P* < 0.01; ***, *P* < 0.001). (B) Genome browser images depicting the relative enrichment and transcript levels of *medA* and *stuA* genes based on ChIP-seq (blue) and RNA-seq (gray). ChIP1 and ChIP2 are two independent repetitions. Scale bar, 1 kb. Download FIG S4, TIF file, 0.5 MB. Copyright © 2020 Chen et al. 2020 Chen et al. This content is distributed under the terms of the Creative Commons Attribution 4.0 International license.

### SomA positively regulates cell wall-related genes.

**FIG 5:**
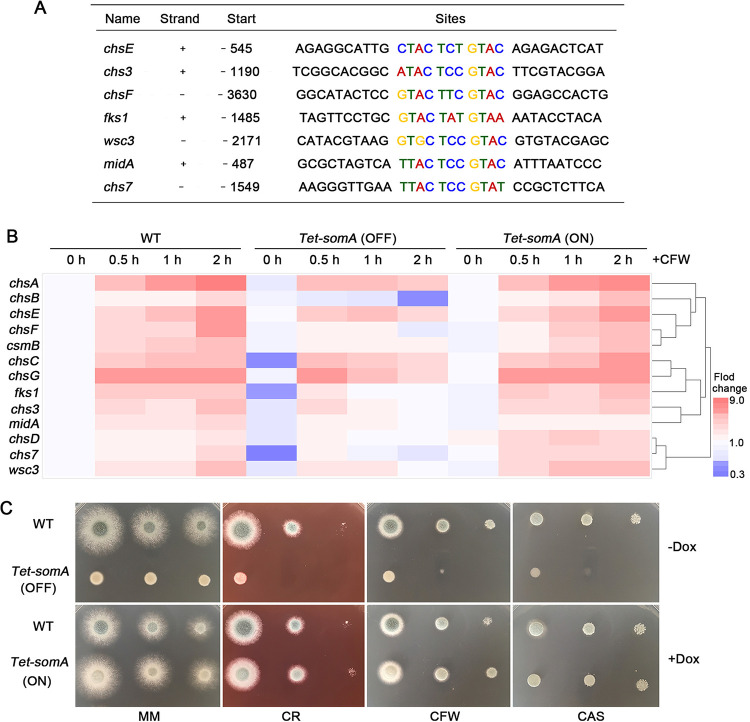
SomA is involved in the cell wall stress response. (A) Summary of the selected cell wall-related genes and the location of their SomA binding motifs. (B) Heat map analysis of the expression of cell wall-related genes. The indicated strains were first cultured in MM with or without doxycycline for 22 h. CFW at a final concentration of 100 μg/ml was then added to the media, and gene expressions were measured at 0.5, 1, and 2 h, respectively. Gene expression was normalized to the endogenous reference gene *tubA*, and expression is reported relative to the unstressed WT at 22 h growth. All of the results were obtained in three independent biological experiments. (C) Phenotypes of WT and *Tet-somA* strains cultured on MM or MM supplemented with or without 1 μg/ml doxycycline and with CR, CFW, or CAS. Colony morphology was imaged after 48 h.

Gene Ontology (GO) analysis of the ChIP-seq data revealed that, in addition to the genes involved in GAG synthesis, SomA occupancy was observed to be associated with genes which encode proteins involved in chitin biosynthesis (*P* < 0.0001), cell wall organization (*P* = 0.00026), and cell adhesion (*P* = 0.00142) (see Table S4). These genes included *midA* and *wsc3*, which encode transmembrane sensors that respond to cell wall perturbations (29); *fks1*, encoding a 1,3-β-glucan synthase catalytic subunit (30); and genes involved in chitin synthesis and remodeling: *chsE*, *chsF*, *chs3*, and *chs7* (31, 32) (see Table S4). Analysis of the sequences upstream of each of these genes revealed the presence of a conserved GTACTCCGTAC motif (Fig. 5A). Consistent with these findings, RT-qPCR analysis revealed increased mRNA accumulation (FC > 2) of these genes following exposure of the wild type to 100 μg/ml wall-perturbing agent CFW for 0.5 to 2 h (Fig. 5B), suggesting that these genes play a role in the compensatory response to cell wall stress. Similar findings were observed with exposure of the *Tet-somA* strain (ON) to CFW (Fig. 5B). Exposure of the *Tet-somA* strain (OFF) to CFW revealed three patterns of gene expression (Fig. 5B). The expression of *fks1* and *chs7* were downregulated (FC > 2) in the *Tet-somA* (OFF) strain compared to the *Tet-somA* (ON) and wild-type strains under both normal growth and in the presence of CFW. In comparison, the expression of *chsC* was downregulated (FC > 2) in the *Tet-somA* (OFF) strain compared to the *Tet-somA* (ON) and wild-type strains under normal culture conditions but exhibited a similar level of upregulation in response to CFW exposure. Finally, *chsA*, *chsB*, *chsE*, *chs3*, and *wsc3* exhibited levels of basal expression in the *Tet-somA* strain (OFF) similar to those observed in the *Tet-somA* (ON) and wild-type strains but reduced expression levels (FC > 2) in response to CFW exposure. These results indicate that SomA is likely part of a complex regulatory network that governs expression of cell wall-related genes under both normal growth and cell wall stress conditions.

To confirm the role of SomA in the regulation of cell wall stress responses, the susceptibilities of the *Tet-somA* mutant to multiple cell wall stressors were determined in the presence or absence of doxycycline. As predicted by our ChIP-seq and gene expression studies, the *Tet-somA* strain (OFF) was hypersensitive to the cell wall-perturbing agents CR, CFW, and CAS (Fig. 5C). Normal resistance to these agents was restored in the *Tet-somA* strain (ON) (Fig. 5C). Taken together, our data suggest that SomA is a global regulator of genes encoding cell wall polysaccharide biosynthesis.

### SomA regulates cell wall architecture and composition.

**FIG 6:**
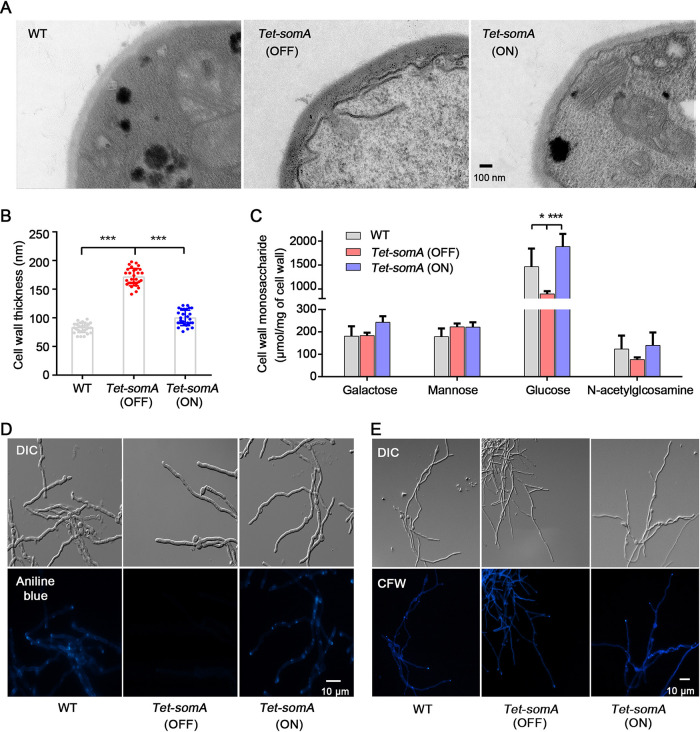
SomA regulates cell wall architecture and compositions. (A) Representative TEM images of hyphae of WT, *Tet-somA* (ON), and *Tet-somA* (OFF) strains cultured on MM. Scale bar, 100 nm. (B) Quantification of the mean cell wall thickness of WT, *Tet-somA* (ON), and *Tet-somA* (OFF) strains as in panel A. The data are presented as the means and standard deviations of three biological samples, with 10 sections were measured for each. (***, *P* < 0.001). (C) Absolute monosaccharide composition of WT, *Tet-somA* (ON) and *Tet-somA* (OFF) mutants mycelial cell walls. The data are presented as the means and standard deviations of three biological replicates. (*, *P* < 0.05; ***, *P* < 0.001). (D and E) Staining of the WT, *Tet-somA* (ON), and *Tet-somA* (OFF) strains for β-1,3-glucan with aniline blue (D) and chitin with CFW (E). Scale bar, 10 μm.

To explore the effects of SomA on cell wall architecture, the hyphal cell wall was inspected by transmission electron microscopy (TEM) (Fig. 6A). Strikingly, the thickness of cell wall in the *Tet-somA* strain (OFF) was found to be 2-fold thicker than that in the *Tet-somA* (ON) strain (Fig. 6B), indicating that SomA plays a potential role in cell wall architecture.

The cell wall monosaccharide compositions in *Tet-somA* and wild-type strains were further analyzed by gas chromatography. Overall, the total amount of cell wall sugars in the *Tet-somA* strain (OFF) was dramatically decreased compared to that in the *Tet-somA* strain (ON) and in the wild type. Among these strains, the amount of cell wall glucose in the *Tet-somA* strain (OFF) decreased to 50% compared to that in the *Tet-somA* strain (ON) (Fig. 6C), suggesting a decreased glucan content in the cell wall of the *Tet-somA* strain (OFF). Consistent with these findings, the total β-1,3-glucan content, as assessed by aniline blue, was dramatically decreased in the *Tet-somA* strain (OFF) (Fig. 6D). In comparison, only a minor decrease in the GlcNAc and galactose content of the cell wall in the *Tet-somA* strain (OFF) was observed (Fig. 6C), and CFW staining of chitin revealed only minor differences between the wild-type, *Tet-somA* (OFF), and *Tet-somA* (ON) strains (Fig. 6E). Taken together, these results demonstrate that SomA plays a crucial role in the maintenance of cell wall composition and architecture.

## DISCUSSION

Given their absence from human hosts and their key role in fungal viability, fungal cell wall biosynthetic enzymes are promising targets for antifungal development. The success of the echinocandins, which target the fungal cell wall by blocking β-1,3-glucan synthase, highlights the potential of this antifungal development strategy (33). Use of these agents, however, has revealed several fungal adaptive mechanisms that can reduce the activity of echinocandins. These include target mutations in the regions of target enzyme, Fks (34, 35), and activation of cell wall stress pathways leading to compensatory effects on cell wall composition, such as increased chitin content (36, 37). This phenomenon may underlie the paradoxical effect, wherein echinocandins are observed to be less effective *in vitro* at high concentrations (38). Here, our data indicate that caspofungin and other cell wall stressors can also induce GAG-mediated biofilm formation. These results are consistent with the observation that deletion of the β-1,3-glucan synthase-encoding gene *fks1* in A. fumigatus resulted in a compensatory increase of both chitin and GAG (30). Considering the important roles of GAG in modulating the immune response during invasive infection and enhancing antifungal resistance, the long-term use of an antifungal drug which causes cell wall stress may increase the risk of biofilm overproduction and subsequent multiple drug resistance and GAG-mediated suppression host inflammatory responses to facilitate fungal survival *in vivo*.

**FIG 7:**
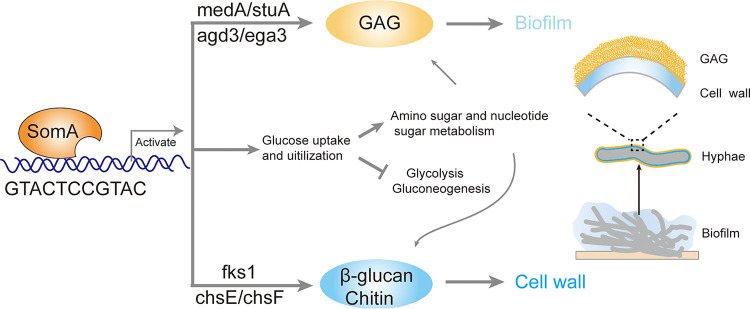
Working model showing how transcription factor SomA synchronously regulates biofilm formation and cell wall homeostasis. Transcription factor SomA plays a dual role in GAG and cell wall polysaccharides biosynthesis by direct binding to a conserved GTACTCCGTAC motif upstream of GAG biosynthetic genes (*agd3* and *ega3*), GAG biosynthetic regulators (*medA* and *stuA*), and genes involved in cell wall polysaccharides chitin (*chsE* and *chsF*) and β-glucan (*fks1*) biosynthesis. Moreover, SomA globally regulates glucose uptake and utilization, as well as amino sugar and nucleotide sugar metabolism, which provides precursors for both GAG and cell wall polysaccharide biosynthesis.

In the present study, we demonstrate that SomA plays a central role in the signaling pathway that integrates biofilm formation and cell wall homeostasis (Fig. 7). Multiple lines of evidence implicate the transcription factor SomA in the regulation of both the GAG-mediated biofilm formation and cell wall homeostasis: (i) cell wall stress induced biofilm formation in a SomA-dependent manner; (ii) SomA regulated cell wall architecture and compositions under both normal and cell wall stress conditions; (iii) the downregulation of *somA* resulted in a severe biofilm formation defect and hypersensitivity to cell wall stressors; (iv) SomA governed the expression of GAG biosynthetic genes and cell wall-related genes under both normal and cell wall stress conditions; and (v) ChIP-seq analysis demonstrated SomA-binding sites proximal to both GAG biosynthetic genes and cell wall-related genes encoding chitin biosynthesis and glucan biosynthesis.

SomA orthologues play a conserved role in the regulation of adherence and biofilm formation in both S. cerevisiae (39) and A. fumigatus (17). In the nonpathogenic yeast S. cerevisiae, Flo8 governs aggregation and biofilm formation by direct regulation of the expression of *flo11*, a gene encoding cell surface-bound protein containing serine threonine-rich conserved repeats. A. fumigatus lacks Flo11 homologous adhesins, and adherence and biofilm formation of this filamentous fungus are mediated by the production of the exopolysaccharide GAG, which is absent in yeast (10). In A. fumigatus, SomA directly regulates the expression of the GAG biosynthetic genes *agd3* and *ega3*, demonstrating that these fungi utilize a conserved regulator of adhesion and biofilm formation despite marked divergence of the downstream effectors of these pathways.

Our findings further reveal that SomA regulates GAG production and biofilm formation through distinct pathways depending on the conditions. Under normal growth, the SomA/PtaB complex controls GAG production through regulation of the expression of the GAG biosynthetic genes *uge3*, *agd3*, and *sph3*, as well as the GAG biosynthetic regulators *medA* and *stuA*. Under cell wall stress conditions, the Lim domain protein PtaB and the GAG biosynthetic regulator StuA were not involved in increased biofilm formation. SomA may form complexes or otherwise interact with new partners to regulate the expression of GAG biosynthetic genes *uge3*, *agd3*, *ega3*, and *sph3*.

Targeting regulators that are crucial for stress responses may provide a powerful strategy for antifungal drug development. One strategy is to combine inhibitors of the stress response with conventional antifungals for treatment of fungal infections (40). Since SomA lacks an identifiable ortholog in humans but is required for stress responses, biofilm formation, and cell wall homeostasis in A. fumigatus, it may prove an attractive target for antifungal drug development.

## MATERIALS AND METHODS

### Strains, media, and culture conditions.

All strains used in this study are listed in Table S5 in the supplemental material. A. fumigatus A1160 (Δ*ku80 pyrG*) was purchased from the Fungal Genetics Stock Center; its complemented strain, A1160^C^ (A1160 *pyr4*) (41), was used as the parental wild type (WT). All *Aspergillus* strains were grown on minimal medium (MM) containing 1% glucose as carbon sources, 70 mM NaNO_3_ as nitrogen sources, and trace elements at 37°C, except as noted. To induce the expression of *somA* in the *Tet-somA* mutant, the medium was supplemented with 1 μg/ml doxycycline.

10.1128/mBio.02329-20.9 TABLE S5 Strains used in this study. Download Table S5, DOC file, 0.1 MB. Copyright © 2020 Chen et al. 2020 Chen et al. This content is distributed under the terms of the Creative Commons Attribution 4.0 International license.

For routine culture, Candida albicans and Cryptococcus neoformans strains were incubated in liquid YPD (1% yeast extract, 2% dextrose, and 2% peptone) at 30°C. For biofilm formation assays, C. albicans and C. neoformans strains were grown in RPMI 1640 without sodium bicarbonate and phenol red at 37°C.

### Construction of genetic mutant strains.

To generate the indicated mutant strains, the fusion PCR method was used, as previously described (42). For *Tet-somA* mutant construction, the endogenous promoter of *somA* was replaced with a conditional doxycycline-inducible Tet-On promoter (17, 25). Briefly, the pyrithiamine resistance cassette and the Tet system from pCH008 were amplified with the primer pair TetF/TetR. Approximately 1 kb of the upstream and downstream flanking sequences of the *somA* promoter regions at positions −802 and +1 were amplified with the primer pairs Tet-somAP1/Tet-somAP3 and Tet-somAP4/Tet-somAP6, respectively. The three purified PCR products were then used as a template to generate the *Tet-somA* cassette with the primers Tet-somAP2/Tet-somAP5. The resulting fusion product was cloned into the pEASY-Blunt Zero cloning kit (TransGen Biotech) and used to transform the WT recipient strain. Transformants were grown on media supplemented with 0.1 μg/ml pyrithiamine (Sigma) and verified by diagnostic PCR using the primer pairs Tet-somASF/SR, Tet-somAP1/Tet-ptrA down, and Tet-somAP6/Tet-ptrA up.

To construct the deletion strain of *medA*, the ORF of *medA* was replaced with a selective marker *pyr4*. The selective marker *pyr4* was amplified from the pAL5 plasmid using the primer pair Pyr4F/4R. Approximately 1 kb of the upstream and downstream flanking sequences of the *medA* ORF were amplified with the primer pairs MedAP1/P3 and MedAP4/P6, respectively. These three PCR products were used as the template to generate the *medA* knockout cassette with the primers MedAP2/P5. The resulting fusion products were cloned into the pEASY-Blunt Zero cloning kit (TransGen Biotech) and used to transform the recipient strain A1160. The transformants were grown on MM and verified by diagnostic PCR using primers MedASF/SR, MedAP1/Cpyr4R, and MedAP6/Cpyr4F, respectively. A similar strategy was used to construct the Δ*stuA* mutant.

To generate a FLAG-tagged SomA strain, the *flag* and *hph* fragment were amplified with primer pairs FlagSF/Flag-hphSR and Hph-flagF/HphSR, respectively. The two purified PCR products were then used as the template to generate the *flag-hph* fragment using the primers FlagSF/HphSR. Approximately 1 kb of the upstream and downstream flanking sequences of the SomA termination codon was amplified with the primer pairs SomAflagP1/P3 and SomAflagP4/P6, respectively. The upstream, downstream, and *flag-hph* fragments were used as templates to generate *somA-flag* cassette with the primers SomAflagP2/P5, and the resulting fusion products were sequence verified and then used to transform the WT recipient strain. Transformants were grown on media supplemented with 200 μg/ml hygromycin B and verified by diagnostic PCR and Western blotting. All primers used in this study are listed in Table S6 in the supplemental material.

10.1128/mBio.02329-20.10 TABLE S6 Primers used in this study. Download Table S6, DOCX file, 0.02 MB. Copyright © 2020 Chen et al. 2020 Chen et al. This content is distributed under the terms of the Creative Commons Attribution 4.0 International license.

### Biofilm formation assay.

*Aspergillus* biofilm visualization and quantification were performed as previously described (15) with minor modifications. Briefly, 96-well non-tissue-culture-treated plates (Corning) were inoculated with 150 μl of MM per well containing 2 × 10^5^/ml conidia, followed by incubation at 37°C. After the indicated incubation period, the biofilms were washed twice with 200 μl of distilled water. Adherent biofilms were stained with 100 μl of 0.1% (wt/vol) crystal violet for 10 min at room temperature. The excess crystal violet solution was removed, and the stained biofilms were washed twice with 200 μl of distilled water. The biofilms were then destained by adding 125 μl of ethanol to each well for 10 min. The quantification of fungal biofilm by determining the absorbance of 75 μl of destain solution at 600 nm.

C. albicans and C. neoformans biofilm visualization and quantification were performed as previously described (43–45) with minor modifications. Yeast strains were precultured in YPD at 30°C overnight and then diluted to an optical density at 600 nm (OD_600_) of 0.5 in RPMI 1640 medium. The 96-well microtiter plates were inoculated with 150 μl of fungal suspension, followed by incubation for 48 h at 37°C. Biofilm-containing wells were washed once time with 200 μl of distilled water and air dried for 10 min. The wells were stained with 100 μl of 0.2% (wt/vol) crystal violet for 10 min and then washed. Biofilms were destained with 200 μl of 100% ethanol for 10 min. The quantification of fungal biofilm by determining the absorbance of 75 μl of destain solution at 600 nm.

### Scanning electron microscopy analysis of the cell surface.

For hyphal surface characterization, SEM was performed as previously described (24) with minor modifications. Briefly, WT and *Tet-somA* (ON) strains were grown statically in MM with or without 12.8 μg/ml calcofluor white (CFW) for 24 h. To compensate for their reduced growth rate, the *Tet-somA* (OFF) strain was grown for 36 h in MM with or without 12.8 μg/ml CFW. Mycelia were washed with phosphate-buffered saline (PBS), fixed with 2.5% glutaraldehyde in 0.1 M sodium cacodylate buffer at room temperature for 2 h, and then sequentially dehydrated in 30, 50, 70, and 80% ethanol for 15 min each. Samples were then dehydrated twice in 90% ethanol for 20 min, followed by 100% ethanol. The samples were dried at a critical point, followed by sputter coating with Au-Pd (Quorum Q150T Es plus), and then imaged with a field-emission scanning electron microscope (Zeiss Gemini SEM500).

### Galactosaminogalactan characterization.

To characterize the galactosaminogalactan (GAG) on the surface of mycelium, an immunofluorescence assay was performed as previously described (8). Briefly, fungi were grown in MM with or without CFW on glass coverslips. After 8 h of growth, the samples were washed twice with PBS and subsequently stained with fluorescein-conjugated soybean agglutinin (Vector Labs) in a dark chamber. The mycelia were then washed twice and imaged using a microscope (Zeiss).

### RNA isolation and RT-qPCR.

To analyze the relative expression levels of genes within the GAG cluster under normal growth conditions, WT and *Tet-somA* strains were incubated in MM for 24 h at 37°C. To analyze the relative gene expression levels of the GAG cluster and chitin synthase genes under conditions of CFW stress, WT and *Tet-somA* strains were incubated in MM for 22 h, and then the samples were supplemented with 100 μg/ml CFW for 0.5, 1, or 2 h. To induce the expression of *somA* in the *Tet-somA* mutant, the medium was supplemented with 1 μg/ml doxycycline. The samples were collected and subsequently frozen using liquid nitrogen. Total RNA was isolated using UNIQ-10 column total RNA purification kit (Shanghai Sangon Biotech) according to the manufacturer’s instructions. For gDNA digestion and cDNA synthesis, the HiScriptII Q RT SuperMix for qRCR (+gDNA wiper) kit (Vazyme) were used according to the manufacturer’s instructions. To analyze the relative expression of the interest genes, the resulting cDNAs were used for quantitative PCR, performed with an ABI one-step fast thermocycler (Applied Biosystems) and AceQ qPCR SYBR green master mix (Vazyme). The results were then normalized to *tubA*, and expression levels were calculated using the ΔΔ*C_T_* method (46).

### RNA-seq.

For RNA sequencing, the *Tet-somA* strain was grown in MM with or without 1 μg/ml doxycycline for 22 h and then exposed or not exposed to 100 μg/ml CFW for 2 h. The samples were collected and subsequently frozen using liquid nitrogen. After mRNA purification and library construction, the samples were sequenced by next-generation sequencing (NGS) based on the Illumina sequencing platform. The threshold value of differentially expressed genes were a fold change of >2 and a *P* value of <0.05. RNA isolation, mRNA purification, and cDNA synthesis and sequencing were performed by Shanghai Personal Biotechnology (China). All the samples were evaluated using three biological repetitions.

### ChIP-seq and MEME analysis.

The SomA-FLAG strain was incubated in MM for 24 h and then cross-linked by 1% formaldehyde for 10 min at 37°C. Cross-linking was stopped by supplementation with 0.125 M glycine and incubation for 5 min at room temperature. The samples were then washed twice with ice-cold PBS and frozen with liquid nitrogen. Immunoprecipitation of DNA was performed as previously described (47, 48). Briefly, after cell lysis, the samples were sheared by sonication (Diagenode Bioruptor Pico) to approximately 500- to 1,000-bp fragments. Immunoprecipitation was performed using Dynabeads-protein G (Thermo Fisher) and anti-FLAG M2 monoclonal antibody (Sigma). ChIP-seq libraries were constructed according to the manufacturer’s instructions for Illumina ChIP-seq library preparation. The output data were processed with a cutoff *q*-value of 0.001 and a fold enrichment of >2. Immunoprecipitation and sequencing were performed by Bio-Tech & Consult (Shanghai).

To identify conserved SomA binding sequences, the output target sequences of SomA were analyzed by using the MEME suite (http://meme-suite.org/tools/meme) with the class mode for motif discovery and a site distribution of zero or one occurrence per sequence (“zoops”), and the minimum and maximum widths of the motif were set at 6 and 12, respectively.

### SomA protein expression, purification, and electrophoretic mobility shift assay.

For SomA protein recombination studies, the full-length cDNA sequence of SomA was amplified with the primer pair pet30-SomAF/R, and the resulting product was cloned into the NdeI and HindIII site of pET-30a(+). The constructs were then transformed into Escherichia coli DE3 (TransGen Biotech). SomA was purified using His tag purification resin (Beyotime).

For DNA probe preparation, the probe was labeled with Cy5 using two-step PCR. Briefly, ∼200 bp of the target sequence-containing motifs were amplified with the primer pair EMSAF/R (e.g., agd3EMSAF/R), which included probe primer oligonucleotide. The resulting products were then used as the template and amplified with a probe primer labeled with Cy5 (Cy5labeled) to generate Cy5-labeled probe DNA.

To generate the *agd3* mutant probe, a fusion PCR method was used. Briefly, ∼1,000 bp of the upstream and downstream flanking sequences of the conserved motif were amplified with the primer pairs agd3mutF1/R1 and F2/R2, respectively. For site-directed mutagenesis, the complementary primers agd3mutR1 and agd3mutF2 harboring the desired mutation in the center position were designed and synthesized. The two purified products were then used as a template to generate a mutant sequence using the primer pair agd3fusionF/R. The sequenced mutant sequence was then used as a template to generate Cy5-labeled *agd3* mutant probe DNA according to the method described above.

The EMSA was performed as described previously (49) with minor modifications. For EMSA, 1 μg of salmon sperm DNA was used as a nonspecific competitor, and 20-fold nonlabeled DNA was used as a competitive cold probe. The reaction mixtures consisted of 30 μl of 1× EMSA binding buffer containing nonspecific competitor, 100 ng of probe DNA, and 1 or 1.5 μg of recombination protein. The samples were incubated at 37°C for 30 min and then separated on a 5% polyacrylamide gel in 0.5 Tris-borate EDTA buffer. After electrophoresis, the Cy5-labeled probes were detected with an Odyssey machine (LI-COR).

### Plate assays.

To test the sensibility of WT and *Tet-somA* strains to cell wall-perturbing agents, minimal medium was supplemented with 50 μg/ml CFW, 20 μg/ml Congo red, or 1.25 μg/ml caspofungin. Then, 2-μl portions of conidial suspensions (1 × 10^7^, 1 × 10^6^, or 1 × 10^5^ conidia/ml) of the indicated strains were spotted onto the relevant media plates with or without doxycycline, grown at 37°C for 48 h, and observed and imaged.

### Transmission electron microscopy analysis of the cell wall.

The cell walls of WT and *Tet-somA* strains were examined by TEM, as previously described (50). After the indicated incubation period, the mycelia were fixed overnight in 0.1 M sodium phosphate buffer (pH 7.4) containing 2.5% glutaraldehyde at 4°C. The samples were embedded in 1% (wt/vol) agar, fixed in 0.1 M sodium phosphate buffer (pH 7.4) containing 1% OsO_4_ for 2 h, and sequentially dehydrated in 50, 70, 80, 90, 95, and 100% ethanol and 100% acetone for 15 min each. Samples were embedded in 812 epoxy resin monomer (SPI), sliced into 60- to 80-nm ultrathin sections using an ultrathin microtome (Leica UC7), stained with uranyl acetate and lead citrate, and imaged at 80 kV using a transmission electron microscope (Hitachi HT7700).

### Cell wall monosaccharide analysis.

WT and *Tet-somA* strains were incubated in MM for 24 h at 37°C. After incubation, fungal balls were collected by Miracloth filtration and washed in 70% ethanol. The resulting biomass was crushed in a glass cell homogenizer in 70% ethanol. Pellets were washed five times with 70% ethanol at 70°C and then in a solution of methanol and chloroform (1:1 [vol/vol]) for 24 h and acetone for 24 h. Pellets were dried, and 1 mg of the resulting preparation was analyzed by gas chromatography-mass spectrometry. Samples were hydrolyzed with either 2 M trifluoroacetic acid for 2 h at 110°C or 6 M hydrochloric acid (HCl) for 4 h at 100°C. After drying, samples were derivatized and analyzed as previously described (13). Briefly, samples were converted in methyl glycosides by heating in 1 M methanol-HCl (Supelco) for 16 h at 80°C. Samples were dried and washed twice with methanol prior re-N-acetylating hexosamine residues. Re-N-acetylation was performed by incubation with a mix of methanol, pyridine, anhydride acetic acid (10:2:3) for 1 h at room temperature. Samples were then treated with hexamethyldisilazane-trimethylchlorosilane-pyridine solution (3:1:9; Supelco) for 20 min at 80°C. The resulting TMS methyl glycosides were dried, resuspended in 1 ml of cyclohexane, and injected in the Trace1300 GC-MS system equipped with a CP-Sil5-CB capillary column (Agilent Technologies). Elution was performed using the following temperature gradient: 120 to 160°C at a rate of 10°C/min, 160 to 220°C at a rate of 1.5°C/min, and 220 to 280°C at a rate of 20°C/min. Identification and quantification of each monosaccharide was carried out using standards and response factors determined for each monosaccharide.

### Calcofluor white and aniline blue staining.

Mycelium were stained with CFW and aniline blue as previously described (51). For CFW staining, hyphae were washed with PBS and stained with 10 mg/ml CFW for ∼2 min. For aniline blue staining, samples were stained with a freshly prepared 0.05% (wt/vol) aniline blue solution for 60 min. These samples were then washed with PBS and immediately imaged by fluorescence microscopy.

### Data analysis.

All statistical analyses were performed using GraphPad Prism 6 software. Multiple comparisons were analyzed by one-way analysis of variance. A *P* value of <0.05 was considered statistically significant.

### Data availability.

The RNA-seq and ChIP-seq data have been deposited in the NCBI Sequence Read Archive under accession numbers PRJNA647130 and PRJNA647621, respectively. Other relevant data supporting the findings of this study are available in this article and its associated supplemental material.

## References

[1] Donlan RM, Costerton JW. 2002. Biofilms: survival mechanisms of clinically relevant microorganisms. Clin Microbiol Rev 15:167–193. doi:10.1128/cmr.15.2.167-193.2002. 11932229 PMC118068

[2] Donlan RM. 2001. Biofilm formation: a clinically relevant microbiological process. Clin Infect Dis 33:1387–1392. doi:10.1086/322972. 11565080

[3] Sheppard DC, Howell PL. 2016. Biofilm exopolysaccharides of pathogenic fungi: lessons from bacteria. J Biol Chem 291:12529–12537. doi:10.1074/jbc.R116.720995. 27129222 PMC4933471

[4] Latge JP, Chamilos G. 2019. *Aspergillus fumigatus* and aspergillosis in 2019. Clin Microbiol Rev 33:e00140-18. doi:10.1128/CMR.00140-18. 31722890 PMC6860006

[5] Loussert C, Schmitt C, Prevost MC, Balloy V, Fadel E, Philippe B, Kauffmann-Lacroix C, Latge JP, Beauvais A. 2010. *In vivo* biofilm composition of *Aspergillus fumigatus*. Cell Microbiol 12:405–410. doi:10.1111/j.1462-5822.2009.01409.x. 19889082

[6] Speth C, Rambach G, Lass-Florl C, Howell PL, Sheppard DC. 2019. Galactosaminogalactan (GAG) and its multiple roles in *Aspergillus* pathogenesis. Virulence 10:976–983. doi:10.1080/21505594.2019.1568174. 30667338 PMC8647848

[7] Fontaine T, Delangle A, Simenel C, Coddeville B, van Vliet SJ, van Kooyk Y, Bozza S, Moretti S, Schwarz F, Trichot C, Aebi M, Delepierre M, Elbim C, Romani L, Latge JP. 2011. Galactosaminogalactan, a new immunosuppressive polysaccharide of *Aspergillus fumigatus*. PLoS Pathog 7:e1002372. doi:10.1371/journal.ppat.1002372. 22102815 PMC3213105

[8] Gravelat FN, Beauvais A, Liu H, Lee MJ, Snarr BD, Chen D, Xu W, Kravtsov I, Hoareau CM, Vanier G, Urb M, Campoli P, Al Abdallah Q, Lehoux M, Chabot JC, Ouimet MC, Baptista SD, Fritz JH, Nierman WC, Latge JP, Mitchell AP, Filler SG, Fontaine T, Sheppard DC. 2013. *Aspergillus galactosaminogalactan* mediates adherence to host constituents and conceals hyphal beta-glucan from the immune system. PLoS Pathog 9:e1003575. doi:10.1371/journal.ppat.1003575. 23990787 PMC3749958

[9] Lee MJ, Gravelat FN, Cerone RP, Baptista SD, Campoli PV, Choe SI, Kravtsov I, Vinogradov E, Creuzenet C, Liu H, Berghuis AM, Latge JP, Filler SG, Fontaine T, Sheppard DC. 2014. Overlapping and distinct roles of *Aspergillus fumigatus* UDP-glucose 4-epimerases in galactose metabolism and the synthesis of galactose-containing cell wall polysaccharides. J Biol Chem 289:1243–1256. doi:10.1074/jbc.M113.522516. 24257745 PMC3894311

[10] Lee MJ, Geller AM, Bamford NC, Liu H, Gravelat FN, Snarr BD, Le Mauff F, Chabot J, Ralph B, Ostapska H, Lehoux M, Cerone RP, Baptista SD, Vinogradov E, Stajich JE, Filler SG, Howell PL, Sheppard DC. 2016. Deacetylation of fungal exopolysaccharide mediates adhesion and biofilm formation. mBio 7:e00252-16. doi:10.1128/mBio.00252-16. 27048799 PMC4817252

[11] Snarr BD, Baker P, Bamford NC, Sato Y, Liu H, Lehoux M, Gravelat FN, Ostapska H, Baistrocchi SR, Cerone RP, Filler EE, Parsek MR, Filler SG, Howell PL, Sheppard DC. 2017. Microbial glycoside hydrolases as antibiofilm agents with cross-kingdom activity. Proc Natl Acad Sci U S A 114:7124–7129. doi:10.1073/pnas.1702798114. 28634301 PMC5502622

[12] Bamford NC, Le Mauff F, Van Loon JC, Ostapska H, Snarr BD, Zhang YZ, Kitova EN, Klassen JS, Codee JDC, Sheppard DC, Howell PL. 2020. Structural and biochemical characterization of the exopolysaccharide deacetylase Agd3 required for *Aspergillus fumigatus* biofilm formation. Nat Commun 11:1–13. doi:10.1038/s41467-020-16144-5. 32415073 PMC7229062

[13] Bamford NC, Snarr BD, Gravelat FN, Little DJ, Lee MJ, Zacharias CA, Chabot JC, Geller AM, Baptista SD, Baker P, Robinson H, Howell PL, Sheppard DC. 2015. Sph3 is a glycoside hydrolase required for the biosynthesis of galactosaminogalactan in *Aspergillus fumigatus*. J Biol Chem 290:27438–27450. doi:10.1074/jbc.M115.679050. 26342082 PMC4645995

[14] Bamford NC, Le Mauff F, Subramanian AS, Yip P, Millan C, Zhang YZ, Zacharias C, Forman A, Nitz M, Codee JDC, Uson I, Sheppard DC, Howell PL. 2019. Ega3 from the fungal pathogen *Aspergillus fumigatus* is an endo-α-1,4-galactosaminidase that disrupts microbial biofilms. J Biol Chem 294:13833–13849. doi:10.1074/jbc.RA119.009910. 31416836 PMC6746457

[15] Gravelat FN, Ejzykowicz DE, Chiang LY, Chabot JC, Urb M, Macdonald KD, Al-Bader N, Filler SG, Sheppard DC. 2010. *Aspergillus fumigatus* MedA governs adherence, host cell interactions, and virulence. Cell Microbiol 12:473–488. doi:10.1111/j.1462-5822.2009.01408.x. 19889083 PMC3370655

[16] Zhang S, Chen Y, Ma Z, Chen Q, Ostapska H, Gravelat FN, Lu L, Sheppard DC. 2018. PtaB, a Lim-domain binding protein in *Aspergillus fumigatus* regulates biofilm formation and conidiation through distinct pathways. Cell Microbiol 20:e12799. doi:10.1111/cmi.12799. 29114981

[17] Lin C-J, Sasse C, Gerke J, Valerius O, Irmer H, Frauendorf H, Heinekamp T, Strassburger M, Tran VT, Herzog B, Braus-Stromeyer SA, Braus GH. 2015. Transcription factor SomA is required for adhesion, development and virulence of the human pathogen *Aspergillus fumigatus*. PLoS Pathog 11:e1005205. doi:10.1371/journal.ppat.1005205. 26529322 PMC4631450

[18] Latge JP, Beauvais A, Chamilos G. 2017. The cell wall of the human fungal pathogen *Aspergillus fumigatus*: biosynthesis, organization, immune response, and virulence. Annu Rev Microbiol 71:99–116. doi:10.1146/annurev-micro-030117-020406. 28701066

[19] Beauvais A, Fontaine T, Aimanianda V, Latge JP. 2014. Aspergillus cell wall and biofilm. Mycopathologia 178:371–377. doi:10.1007/s11046-014-9766-0. 24947169

[20] Fontaine T, Sinenel C, Dubreucq G, Adam O, Delepierre M, Lemoine J, Vorgias CE, Diaquin M, Latge JP. 2000. Molecular organization of the alkali-insoluble fraction of *Aspergillus fumigatus* cell wall. J Biol Chem 275:41528–41529. 11134062

[21] Lamarre C, Beau R, Balloy V, Fontaine T, Wong Sak Hoi J, Guadagnini S, Berkova N, Chignard M, Beauvais A, Latge JP. 2009. Galactofuranose attenuates cellular adhesion of *Aspergillus fumigatus*. Cell Microbiol 11:1612–1623. doi:10.1111/j.1462-5822.2009.01352.x. 19563461

[22] Hopke A, Brown AJP, Hall RA, Wheeler RT. 2018. Dynamic fungal cell wall architecture in stress adaptation and immune evasion. Trends Microbiol 26:284–295. doi:10.1016/j.tim.2018.01.007. 29452950 PMC5869159

[23] Nett JE, Sanchez H, Cain MT, Ross KM, Andes DR. 2011. Interface of *Candida albicans* biofilm matrix-associated drug resistance and cell wall integrity regulation. Eukaryot Cell 10:1660–1669. doi:10.1128/EC.05126-11. 21666076 PMC3232725

[24] Lee MJ, Liu H, Barker BM, Snarr BD, Gravelat FN, Al Abdallah Q, Gavino C, Baistrocchi SR, Ostapska H, Xiao T, Ralph B, Solis NV, Lehoux M, Baptista SD, Thammahong A, Cerone RP, Kaminskyj SG, Guiot MC, Latge JP, Fontaine T, Vinh DC, Filler SG, Sheppard DC. 2015. The fungal exopolysaccharide galactosaminogalactan mediates virulence by enhancing resistance to neutrophil extracellular traps. PLoS Pathog 11:e1005187. doi:10.1371/journal.ppat.1005187. 26492565 PMC4619649

[25] Helmschrott C, Sasse A, Samantaray S, Krappmann S, Wagener J. 2013. Upgrading fungal gene expression on demand: improved systems for doxycycline-dependent silencing in *Aspergillus fumigatus*. Appl Environ Microbiol 79:1751–1754. doi:10.1128/AEM.03626-12. 23275515 PMC3591957

[26] Neigeborn L, Schwartzberg P, Reid R, Carlson M. 1986. Null mutations in the SNF3 gene of *Saccharomyces cerevisiae* cause a different phenotype than do previously isolated missense mutations. Mol Cell Biol 6:3569–3574. doi:10.1128/mcb.6.11.3569. 3540596 PMC367116

[27] Flipphi M, Sun J, Robellet X, Karaffa L, Fekete E, Zeng AP, Kubicek CP. 2009. Biodiversity and evolution of primary carbon metabolism in *Aspergillus nidulans* and other *Aspergillus* spp. Fungal Genet Biol 46(Suppl 1):S19–S44. doi:10.1016/j.fgb.2008.07.018. 19610199

[28] de Assis LJ, Manfiolli A, Mattos E, Fabri J, Malavazi I, Jacobsen ID, Brock M, Cramer RA, Thammahong A, Hagiwara D, Ries LNA, Goldman GH. 2018. Protein kinase A and high-osmolarity glycerol response pathways cooperatively control cell wall carbohydrate mobilization in *Aspergillus fumigatus*. mBio 9:e01952-18. doi:10.1128/mBio.01952-18. 30538182 PMC6299480

[29] Dichtl K, Helmschrott C, Dirr F, Wagener J. 2012. Deciphering cell wall integrity signaling in *Aspergillus fumigatus*: identification and functional characterization of cell wall stress sensors and relevant Rho GTPases. Mol Microbiol 83:506–519. doi:10.1111/j.1365-2958.2011.07946.x. 22220813

[30] Dichtl K, Samantaray S, Aimanianda V, Zhu Z, Prevost MC, Latge JP, Ebel F, Wagener J. 2015. *Aspergillus fumigatus* devoid of cell wall β-1,3-glucan is viable, massively sheds galactomannan and is killed by septum formation inhibitors. Mol Microbiol 95:458–471. doi:10.1111/mmi.12877. 25425041

[31] Muszkieta L, Aimanianda V, Mellado E, Gribaldo S, Alcazar-Fuoli L, Szewczyk E, Prevost MC, Latge JP. 2014. Deciphering the role of the chitin synthase families 1 and 2 in the *in vivo* and *in vitro* growth of *Aspergillus fumigatus* by multiple gene targeting deletion. Cell Microbiol 16:1784–1805. doi:10.1111/cmi.12326. 24946720

[32] Jain R, Valiante V, Remme N, Docimo T, Heinekamp T, Hertweck C, Gershenzon J, Haas H, Brakhage AA. 2011. The MAP kinase MpkA controls cell wall integrity, oxidative stress response, gliotoxin production and iron adaptation in *Aspergillus fumigatus*. Mol Microbiol 82:39–53. doi:10.1111/j.1365-2958.2011.07778.x. 21883519 PMC3229709

[33] Aguilar-Zapata D, Petraitiene R, Petraitis V. 2015. Echinocandins: the expanding antifungal armamentarium. Clin Infect Dis 61:S604–S611. doi:10.1093/cid/civ814. 26567277

[34] Gardiner RE, Souteropoulos P, Park S, Perlin DS. 2005. Characterization of *Aspergillus fumigatus* mutants with reduced susceptibility to caspofungin. Med Mycol 43:299–S305. doi:10.1080/13693780400029023. 16110824

[35] Rocha EMF, Garcia-Effron G, Park S, Perlin DS. 2007. A Ser678Pro substitution in fks1p confers resistance to echinocandin drugs in *Aspergillus fumigatus*. Antimicrob Agents Chemother 51:4174–4176. doi:10.1128/AAC.00917-07. 17724146 PMC2151465

[36] Shwab EK, Juvvadi PR, Waitt G, Soderblom EJ, Barrington BC, Asfaw YG, Moseley MA, Steinbach WJ. 2019. Calcineurin-dependent dephosphorylation of the transcription factor CrzA at specific sites controls conidiation, stress tolerance, and virulence of *Aspergillus fumigatus*. Mol Microbiol 112:62–80. doi:10.1111/mmi.14254. 30927289 PMC6615987

[37] Juvvadi PR, Munoz A, Lamoth F, Soderblom EJ, Moseley MA, Read ND, Steinbach WJ. 2015. Calcium-mediated induction of paradoxical growth following caspofungin treatment is associated with calcineurin activation and phosphorylation in *Aspergillus fumigatus*. Antimicrob Agents Chemother 59:4946–4955. doi:10.1128/AAC.00263-15. 26055379 PMC4505252

[38] Steinbach WJ, Lamoth F, Juvvadi PR. 2015. Potential microbiological effects of higher dosing of echinocandins. Clin Infect Dis 61:S669–S677. doi:10.1093/cid/civ725. 26567286

[39] Fichtner L, Schulze F, Braus GH. 2007. Differential Flo8p-dependent regulation of FLO1 and FLO11 for cell-cell and cell-substrate adherence of *S. cerevisiae* S288c. Mol Microbiol 66:1276–1289. doi:10.1111/j.1365-2958.2007.06014.x. 18001350 PMC2780560

[40] Xie JLL, Qin LG, Miao ZQ, Grys BT, Diaz JD, Ting K, Krieger JR, Tong JF, Tan KL, Leach MD, Ketela T, Moran MF, Krysan DJ, Boone C, Andrews BJ, Selmecki A, Wong KH, Robbins N, Cowen LE. 2017. The *Candida albicans* transcription factor Cas5 couples stress responses, drug resistance, and cell cycle regulation. Nat Commun 8:499. doi:10.1038/s41467-017-00547-y. 28894103 PMC5593949

[41] Jiang H, Shen Y, Liu W, Lu L. 2014. Deletion of the putative stretch-activated ion channel Mid1 is hypervirulent in *Aspergillus fumigatus*. Fungal Genet Biol 62:62–70. doi:10.1016/j.fgb.2013.11.003. 24239700

[42] Szewczyk E, Nayak T, Oakley CE, Edgerton H, Xiong Y, Taheri-Talesh N, Osmani SA, Oakley BR, Oakley B. 2006. Fusion PCR and gene targeting in *Aspergillus nidulans*. Nat Protoc 1:3111–3120. doi:10.1038/nprot.2006.405. 17406574

[43] Pierce CG, Uppuluri P, Tristan AR, Wormley FL, Jr, Mowat E, Ramage G, Lopez-Ribot JL. 2008. A simple and reproducible 96-well plate-based method for the formation of fungal biofilms and its application to antifungal susceptibility testing. Nat Protoc 3:1494–1500. doi:10.1038/nprot.2008.141. 18772877 PMC2741160

[44] Kakade P, Sadhale P, Sanyal K, Nagaraja V. 2016. ZCF32, a fungus specific Zn(II)2 Cys6 transcription factor, is a repressor of the biofilm development in the human pathogen *Candida albicans*. Sci Rep 6:31124. doi:10.1038/srep31124. 27498700 PMC4976313

[45] Ravi S, Pierce C, Witt C, Wormley FL, Jr. 2009. Biofilm formation by *Cryptococcus neoformans* under distinct environmental conditions. Mycopathologia 167:307–314. doi:10.1007/s11046-008-9180-6. 19130292 PMC4278410

[46] Livak KJ, Schmittgen TD. 2001. Analysis of relative gene expression data using real-time quantitative PCR and the 2*–ΔΔC(T)* method. Methods 25:402–408. doi:10.1006/meth.2001.1262. 11846609

[47] Bernstein BE, Liu CL, Humphrey EL, Perlstein EO, Schreiber SL. 2004. Global nucleosome occupancy in yeast. Genome Biol 5:R62. doi:10.1186/gb-2004-5-9-r62. 15345046 PMC522869

[48] Aparicio O, Geisberg JV, Struhl K. 2004. Chromatin immunoprecipitation for determining the association of proteins with specific genomic sequences *in vivo*. Curr Protoc Cell Biol Chapter 17:Unit 17.7. 10.1002/0471143030.cb1707s23 18228445

[49] Huang W, Hong S, Tang G, Lu Y, Wang C. 2019. Unveiling the function and regulation control of the DUF3129 family proteins in fungal infection of hosts. Philos Trans R Soc Lond B Biol Sci 374:20180321. doi:10.1098/rstb.2018.0321. 30967021 PMC6367153

[50] Ries LNA, Rocha MC, de Castro PA, Silva-Rocha R, Silva RN, Freitas FZ, de Assis LJ, Bertolini MC, Malavazi I, Goldman GH. 2017. The *Aspergillus fumigatus* CrzA transcription factor activates chitin synthase gene expression during the caspofungin paradoxical effect. mBio 8:e00705-17. doi:10.1128/mBio.00705-17. 28611248 PMC5472186

[51] Loiko V, Wagener J. 2017. The paradoxical effect of echinocandins in *Aspergillus fumigatus* relies on recovery of the β-1,3-glucan synthase Fks1. Antimicrob Agents Chemother 61:e01690-16. doi:10.1128/AAC.01690-16. 27872079 PMC5278722

