# Supplementary material for: The Transcription Factor SomA Synchronously Regulates Biofilm Formation and Cell Wall Homeostasis in Aspergillus fumigatus
Source: mBio. 2020 Nov 10;11(6):e02329-20. doi: 10.1128/mBio.02329-20 (PMC7667024; doi:10.1128/mBio.02329-20)
Supplement: TABLE S3 [file mBio.02329-20-st003.doc]

**SomA ChIP common targets sequence**

>DS499594:177367-177768

AATGCCCTAGGCAGGCAGCCACTTTCAGGCATCGTCGTTCTACCTGATCAAGGGTAAATGTAAATGGATGCCAGTGGCGCCACGCTTTCCTAGCCGAAGCAACTCGGAATGGCTGTCTTGCAGCCCAGCACAGCGATCTTCGTTTCGCATCTGCTCTCTGCCCTCGTTAGTTGCAACCATCTTCGACCTCCATCCACCAACACCAACCGAAATTGTGAATCCCTGGGGAGACACTTCAGGCTCACCTGACCCTAGCTTCCCGTCGAACACCCAAGAATATTCTCTTCCTCGAGCTTCACTAGTACATGGTTTACTGACCAACGCAGGTGACCTTTACAAATCAATAAATCAAGCAATTGAGTGCATATGTGGAAGCGCAAGCATTATCCCGATGACCATTG

>DS499594:329693-330094

GATTCAAACCCCCAAGACACGTGCTGTGAAATCGTGGCTCGCGCGCACAACGTCAGTGAAGAAAGACGGGGCAAAGAGGTCTCGTCCAGGATAACCCCTTCCGGTAAATTATGTTCTGTGTACTTTTGGATACCGGGCGTAACATACTCCGTCGACCGTTCCGTCATAATTGGCGGAGGTGATTCAGAATCAGCGAATCAGTCGAGTGGGAGGTGGGAGGGTAATAATGTCCTCCACCGGAGTGAGCGATTTCATCCTCCCGAGTGCCCCACTCGGCACGGTGGTTCCGGTGTGAGTCTCTCTTTCCAGAGTTAGGTTGCCAAAGAAGACCGATTTTCCCATCAGCAAGGCCTGCTGGCCCAATGGTAAGGCGCTTGACTACGGATCAAGAGATTGCAGGT

>DS499594:334469-334870

TTCTCCTGGCCAAGCTTTTCTAAGCTTTGGATACTCTTGACCTGCTATTTTGGTCATCGATCAGGATGCACTTAGTATCGACTGTGGAAGGGTGGATCCATCGAGACATGCTCAGCTCTCCATCTCGACCGACGGATGCCGTGTTGAGTGGCTGAGAATACCCTCTTAATCCACTAGCAGGTTAAAGGGCTTCTCTAGTATCCAATCTCAGGATCATGTTGTCCGATCCACTGATGATGCTGTGGTGCCACTTCCCAGAGCACTCGTACACAGCGGCAACACCATTAACAAGCCACCCCCTCATCCCCCCTGTCCCTGTTGATAATAATGACGGTCCTCGATTATACAGCGACCCATCTCTGGTTCCCGCCCTGTTTTGTTTACTCTGATGATCCATGTTG

>DS499594:346589-346990

TGGAGGGTTTCACCGCTAGTCCACAGCAATGGAATCATCGGCGGGGACGATGGTGTGATATCCGCTGCTTCTGACTCGAGTGTCGGGGGTTGGTTCCAGTATCGGTTTCCGGGCTTCCATTCCTCTGGAGTATCAAGGACTCTCCTGAAGATCTTTTGGACTCCACATGATGCTCTAGTCCTCTCTCCATGACCGTTACTTTTCAAACAATGGTCTGTTATGATGAATCAACCTTTTCTCAAAAAATTGATGCGTTGTCATGGGAGTCACATAGGTCGCGTGAGTCGAGAGTGAAAGGACTCGATTACCCAGCATAGAAAAGGGTTCCAGAAAACCAATACACACCAATGACCATCAGTACCACCAGTGATAAGCTGACTAACCGGGTCTTGTATGATTTG

>DS499594:363427-363828

CCTGCAGGTCTATCAGTCCGGCAATCACCACGCCCATCATACCGATGGGATTCGGGTGAGAACCGCAGGTTGGTATTGAAGGGAAATTTATTATTTTCAGCCTCTCGCCCGTTCAAACCTAGATTCGGGGGGGATCCACTGGACAGAAAGCATGGGGAGACGGGGCCCAACTCCGCCGAGCTGGAAGTCGGCACCTAGCGTGATCGGAAAACCGTCTCCAGTCTCAGACTCCTATACCGACCCATGCACATGCAGGTGCTTAATCAGAAACGCAGTCAGTCGGTGGTGGTCTATACTTCGGCTAAGCGCGGATATAATCGGAGTAGCTCCATAGAGCCAATTCTAGAATTTCGCTCGGTTATCCTCGTCTTTGATCCGAGCAAGATAAAGAGATCAAACAG

>DS499594:382187-382588

AAGCGTGTTCCCGCTAATGTCCTGCAGTCTGTCACGCTCTGTCTCTTCTCCCATTCGTCCTCTTACTGCCTCATCCTGCACTTCTCATAGCGCCTGACCAGGGACTGCCGACCTACCAGCTTCGACATCTCCAGGATTTGCCAGCTCGAATTCATTTAGTCTGTTCGCTAGTGTCTATGCTCTCGCCCAATTGTTCATGCTTTTTCTCTCTCTGCATGAAGAAGCCAAGAGGGGCTTTGACGGCCTCGAGCTGCCTGTCGCTTCAATTGGTTCTCAGCTTCTAAGGCGATTGATCAGACACTGCGCGTGTCACAGTTGCTCTGACAATCCCAGAACGACGAATCCTCAACAGTGCCAAGTACTTATTTTTATGATTCTGCACTACTGCGTGGAGAAATTCT

>DS499594:435192-435593

AAGGGCTAAAGCTTGTGGGGTCTTTCCACTAAAAACTCTACCCGCCACCCTCAGACAACCTGCCTATGGATCAAGCTAACTAAACTAGGTCGCAGAATGGCTTATGGCGCCCCCCGGAAAACAACAAAGGACTGGAAAAAGAAAAATAGAACAGTCCAGAAAAAGCCCCAGTGCGCCGAATCAGAAGTTTCCATGACACCTCAGTGGGCTCGTTTACTAGGATAGGTATTTTATGCCCGGCACTAACTAGGTCAAATTTTGCCGTAGTTACTCCGTGGAGACTGCTGGTTCGATCCTCGGAAGTTCTCAAGATTCCGTGATGATAAGCTTGCCCTTTCTATTCTGTATGTTATCGTCAACTCTGTGTAGACAGGCTGAGCTGAGTGGTTCTAAATCATCCT

>DS499594:436125-436526

AGATTGAATGGCGTGAATTGGCCTCGAAGCTAGCATGGAACTCCGACGGGGGATAGTGCTTAACCATGGCGTATCACGGTGGTGCCCGGGAAGCTCAGGCCGAGGTGTTCTGCTCAAATAAGTCCTCCGTAGCGAGACAATCCAACTTTCCTGATGATGAACGACACCGTTACATCGCTTCTTAGTGAACATGGATCACGTGGAGATATTACGAGACTAATAAGCCATGGGACCGCTCAGGCAGGTCTAAGTTAAGGTTGGAGTCTTGAGGATCCTCATGAAGTGATGACAAAAACAGCATCAGGCCTTACATTGGCTGTGATGGCTGGGGTCCAGAGTGGGGAATGCTATCGATCCCCGAGCTCCTGCGCCTCACATGCTGCGATCAGCATTGTTAATTT

>DS499594:506534-506935

TGAGACGGAAATAGCGGTGGGCCAATAGTGTGGCAATGATTATCTATCGTCACTGTGATTCTGTACTGAGGTAACTCGGTACTGGTCCACCGACTTGGATCATACCTAGAAGAAGCCTGAATTCCGTGACCTTACAGCTCATTTGGGTCAAAATGATTACATGGCCCTTTCCATGTTGAAGAATAGAATGAGAATTTGTGTCCTACTCATCAAGAGCAAAGGCCTTGACAGGCGCCCACAGGGGAACCTCAGAAAGCGGCTGTTAATGGGGCTAAAATCATTACGTTTACCTGAATTGCAACTGGACAATGCTAGGACGGAGCAACATGCCGAAAAGAATGAAAACGTCATAAATTACAGAATGTGAAAAGATGCTGTAAGTTCTGTATGGGGGTATGCAG

>DS499594:700436-700837

ATCCCAGCGATCAAAGTGCGAGAATTACCCGAGGACACTGCAGCGTAAACTTGGACGGACGTAAGTATGCACATCGGGAGGGTATGAGGCTAGTTGAAGTCAAGACAATAATGGTCCAGAACAAGGCCGTCTAGACCATCCCGACACCTGGAGCCATTGTTGATCACTTGATCGCATTTGGCATATTCTAGAACAGAGTCATTATATCCCCATATCACAGTCACACTGTATCGACAGGAGGTTGGAAACTGTATTGCCTATGATGGCCTGATATTGTGCATGCTGCAGCGATGGTTCTGGAATTTCACACGGCTTGTCCACGTGCTCTCCCTCAACGGCTGGGACTTTAGGCATTGCGTCTATCTATTGTATACAAAATCGGCCAAGATAGGTACAATATT

>DS499594:824486-824887

AACGACGATCTCAGAACAAGAAGACTGGAAAAAACGCTGGTGGTGATGTCATCGCCGGAGGAAATGTGACGCAGTTTGTCAGCCTTAACAGAGGGGCAGATAGACACTTTCCCAGCCCCATAGCTGTAGTTAGTAATATTACTGTCTTATTATCAATCAGATACCGTGTTTTTTATCGATTTACTCTTTATTTGATAAGATCATACCACTCAATTGTCAGGTAGCTAGGTTTTTTAATGCAATTGAGAATGCTTTGCTACGGATATAACCCACCCTTCCATCTACGTAACTGCAGAGTCGCCCGCTAGAAGTACATCCCGTTGTACTCCGTCCTCCGTACATCGGCCGGCACCAAAGGAGCGAGATCCGAGGAGTAAAAATTTCGATCAATCGACCCTATT

>DS499594:924137-924538

TAAGGCTGATATTGCTTGATGATTCTTTAATGATTGAAGAAAATTACGGCGGTGACTTGATACATCAGGATACACAACTACCACGCCACTCTCGATAGAACCATGCATATTAATATTAATATTGCCATCATACCATCATTATTATTGTCATCGAGTGCTATGATCGATTAGCTCGACTTAGGCTGCCCTGAAACTTGTTGTCGCATCGCGGAATGAGAGCGTTTGTTGACTGGCTGGTAGCTCCTCGCCAATGAAAACGCCCAAACTACCTACTTTAGGTGTTTAGGTACAGAGATGGCATCGGAACCAATCCCTTTCTGGATCGTTCCATGTGCCTTCACATTTCTTTACAAAAGTTGTTCCGCACAAACCCCCAATAGAAATCTTCCATGGATCTCCGA

>DS499594:925946-926347

CTCTCTCTGTTGAGTGATTTTCTCTCATCTATCCGATTTCCGAGAGATCCATGCTAACAATGTGGCAATCGGGCAACATACAACACAATCTCGATGTAATGCGTGCTATAACTGCTGGCTCGAAAGGGCCAGTAATATTTTCGATGCCTGCTTAGACATGCCTCGTCTCTCATCTTGCGACATCTCACGACATCTCACGAATCCGATCATGGAGTCAGTGCCGATAATCATCCAGCTGCCTAGCCATCTTGTGATGGTGTATACAATCGGCATCTCGAACAAATCCTCCCAACTATGGAGAGCATTGTTTTTTTGCAGGATACCCGAGTCCGGCTCTTCCGTCCAGCCGCACCTGCGTCTACGTCCGTCACTAGATCAACTTGAGAACGGGCCGCTTTCTT

>DS499594:950233-950634

TTCTTGTTGTTACGCGTTCTCATTAGTAGGGTTTCAGTCTTTCCTGCCTGTAAGTTAATACTTATTTTGAGTTTTAGAACTATCCCGAGTGCTCTAATTATCCACTGACAACGTTAGTAAGGCTTCATGCTGTAACTCCCCAACTAGCTCAGCTCTGTGGACGATTGTGGTTGGACAGAGCTGTTGAAGAATTGGAGAAAATCAGGTTGTTGTCTTTTAGTCTGGATTATTCCAGCAACATCGGCCGTACGAAACACAACGTACAACAACAATACATGATAACAACCTATGGTAAAGAGGAGCCGATCATCGCCACCGACTGCTATTTCCGAAGCAAGCCACCTTGTTTTGGCTGATATGGTGACACTCAATAATAGTGACACTCAATAATAGTGCAAAGT

>DS499594:1115257-1115658

ATTCAGCTGATCGGAAATCATAATCACTAAATTAAAGGGCCCACGGCAACACGTGTTCCGGTCTTAAGCTCAGATAGATACTAGCACCATGATGCCAGCTATTTCCTAGCTCCCTTCATGATAAAAATTGAAAAATTGATCATACCACTACAAACCCACTTCCGGCAACTGCCCAAGTGTCCGCTCCAATGCAGGATTGAGCAAGCGGGTTCTATCATCGGCTAGAGATGATAGGCCTATTCTATTTCCCTGAGCCGTTCTCCGTCCTAGCCTGTTTTTCAATATTCTCCTCTGGTGAAATGATGTGATCAATTGCAGAACGGTTTCCGAAGCCCGTGTGGAGTGCTCTCGGAATATGGAAGGATCATGACGGTCAGAGAATCAGGACCAGAGCTCGAGCC

>DS499594:1134872-1135273

TTCTGTGGCAGCGTGTCACTGGAATAAAGGGGATACAGGATGGAGTGTCGCTACTATACATGAGAAGGCAGAGGGGTTACGACGACTTGTTTTAATCCTTCGATTTATACCCCAAGTCAGCCGGAATCGATTCTGGAAACCTACGGCGTCCAGGATGACTCCAATGTCGAGTACGGAAAAGACGGATCAATGCTGTGTAAGATGGTGCACTATTATTATTATTATTATTATCCTTTCTGCCTTGTCTTGAAAAAAAGTCAAGGGGCGGAGGGATTGGACCACTGAAGGACAAGAGGGTTTGAGAACCTGGGCGGGCACGAGGTGGATGAACTCTGTGATGGTGGTCAGTTTTGCCCGGCAGACTCGCCTCAAGCGGTGATGCTCATTCGCCAATTTCTCAG

>DS499594:1135927-1136328

AGAGCCGTCAATCTACTATTAGTGCACTGGAACCTTATATATATCTACAATCAAAAATATGGCCGTCCTTGCCTCTATCAATCCTTCAAGGGTGACAACCTCATGATCGACTGAGTCAGACAAAACTTCACTTTCGAGTCTTGAACATGAGCAGCTGACCATTTCTTTTCTTTTACTAATGATTTGTAGCGGAGTATCCGAAGGGAACAATTTTCCTCTGCACTGCAATTTGTCTGGCTTCACCGCAGCCTCTTTTCGGTTGCCGTGAGGCTGCGTCATGTCATGTTCTGTACAGTATATTACCTTCTCGCGCGCCCTTAGTATCAGCAAATACCGACAATACTAATGTATAGTACATGCCATGACATTCTTTCATTACAAAAATGACCGATATGGGCTAT

>DS499594:1177279-1177680

TAGATTGGCTGTATATATGGGACAATATAGAGCACGCGACATTCGAATATCACTGTGGTCGTATCCTCCATCGCAGACCTCACATGAGAAACAAGATCAATGCGACGAGTGGTTACCGGAAGGATTGACACGACTGAAACTCGCTCCGCTGAACCTAATTCGTGGAGTACAAGAATTTATTACCGGACGAAAAACATTCGGTATTTTCCGAAAGATCGCCCCGTCTAAGTCACGGTGACTCATAAGTTCATAACATTGAAGCTGAGATACCCTGAAGTTTTTTTCCACGTGCCTGAAAATTGCTAGGGTGGCAGCAGCTGATTTGCGTAACGTGTCCGAATGTTTTATGGGAAGTCATCTGATAACTTTACAGCGATGACTGTGCTAATCCTAAGGTTGTT

>DS499594:1211456-1211857

TAGTGTCTTTGAAGTAGTCTCATATAAAAGAGAAGCGATCTGATGGACACGTGCCCCGGAAGCATAACAGCCGAAACAGCTGTCAAATTCTCGAATACGCTGGGCACCTGACACGATGACTCGAGCACAGTCCCCGGATACTCCTTCTGCAAGGGCGAAGAAGTCCTATCTCAGTGCTATCGCATGACTCTCGATTGGACGAAACTCCGGCTGTACTACCCGACTAAATAGATATGGCAAGCTTCTACGGTAAGCCTTGCACCTGAATTATTCTTGTGGTTAGGGGTGTGTCATACAGTACCTACTTACTGTCGGATGTTCGTCAAGTCAATCTACGTTCATGGCAATTTTGGATAATGGTTAAGCTTCTTGAACTGCCCTATCTTTGTCAAGTCTATTGA

>DS499594:1292412-1292813

ACAGGTCACTGTTGGTTTTGCACCTGTTAACTCCACCATCCAGTGGCTTGCAAGGGTGGTGACGGAACGAGTGAACCGAACTAATCTGGGGCAGTCGCGCAAGTCTCCAGATCATAGCACAGCGCATGATAAAGTAATTCGTTGAGAAAATGGGCAAGTAAAAGGCTGCGCTTAGGATGAATGAGTTGTCAAGCCACATGAGCATTGCGCAGTGCAGATTGGATTGTTGGCATGAGGTGCATCAATCATTAATCATCGGACAAGCGCTGACTTCCACATACAACCCACTGATCATCATCGTCAGTTCATCATGTTGTTCCACTATCACAGTGGCACCAGTGGCATGAACGACTAAGATAGTCCGGGCGCTATGATCGCTTACCTTACTTTTCCTGGTGGGT

>DS499594:1477860-1478261

AAAGCAACTCTCATCTGACAATAATATACCTCACCAACACGACACCACCCAGAGGTAAGTATTCGATGAAAACCCCGTACAGTTGCTATGTGGGGACCACCTAGTAGATGCCTTTGATTGCTGGAAGATTTGGGAAAGTATGCCACTGGATGAATGAGGTGTGTTGGGCCCAATGTTCGGCCGAATGCAAATATATTTACCTAGGTGTTATTGTCTTACTTCCCTAGTTAAGGTGGTTTCCACTGGAGGTCTATCCGACATAACAATTGGTAATTTGCGATTGGACATGACGTATGAATCTATCAATATGGGAATGATGGGTGCGAATGGAAATTCGGCAATGACGGAAATATAAGCGTATTGTAGGTATCTAGTGCCTGGTGGAAAGTTGCAGAAGGTGC

>DS499594:1479344-1479745

GGACATCCCTTTAAGCATATCCTGCTGACCTCCGATTTTCCCAAAAGGGAAGATACAGAGGACCAGCATAACTGTGCATTGAGTATTGATCTTGGCGGCTCTTGGCGGAGCTCTTGGCTGCCTCGTCAAGTATGCAGTGTGCTCCCTTGAATTTGGAATTCGCCCGGTGGAATGCATTATGCAGGCGATCAGGAGATCGCGATGATGTGCGAGGTTGATTTGCTCCGTCTGCCAGTTCTGATGGTCTCCGGACTCCGTACACCATACACCGTTTGCCGTACAACTTCGGAGATCTATAGTTATCTCACCGAGTCCGTAGAACGATCGGGACCTCTTGGGTCCAGGGCTATCTGGAATGCTTCCAGGTCCATTGACACTTCGGGCTGAGCCTGGCCAAAGCA

>DS499594:1481491-1481892

AGGAAGCTTGACCAAGACTTTGGCCGATGGGCTTCCGTTGATTCGCAAACATGATCCGTAGCTGGCTGGTGGTCCCGCAGTGAAGGCTGGCGAACTGACCATTTGGCGTTCAAAACTCGCGCTCTCATCATCAATCAGAGGGCTGCTTTTTTTACTTTTTCTGACCGCTTCTCTGGCGTCAACCTTCATGATCCAACCTCAGGCTGAGACTGTCAGCTGTCCAGCCAACATCGTATATGTGGAGATTGGGGGACAATCGAAGGTTTGAAGCCTAAAGAGATGTACAATGATCGGAGAGTTCTCAAAGAGATGGACACGTGCTTAGGTGGATACCTCATCGCCACGATACAACGTCTTGAACAATGCTACGGAGTACATAAGCAATATTCTCCTCGGGGTAG

>DS499594:1482840-1483241

GTCTATAGTGTAGCAGAAGTTAAGCACAGCAACACAAATGCGGCACATATAAAAAGCTAAACCACAGACCCAGGTCGTTAGAGGAAATCGAAGACGAAGCGCGCAGAGCGCCTTAATTATTATTTGGCATCATCATCATTTGCTCCAGAGTAGTACTAGTGTTATCCACTTGAGCCAAAGTAGTAGGTTCAGGATTAGGAACCATACAGAGTGCAGGTAATCTTAATCGCCAACCCAGCGATGAGGATACCACTAAACCATCCAGTAAGGTAGTTGACTTTAGATCGAATTGAGTAATACCACCTCGTAAGGGAACGTAACATTTGAAACCTTACAGTCTTGGGGTGATTTTGACGGTGTTTGTATTGCCCTTTGAGCCCTCGCCGCCCCTTTTGTGTGAG

>DS499594:1521951-1522352

TATAATCCTGGTCTTCCCCACTCTGACACCATCGAGATCAAAATCGCATCATCTCCATCTTATCACAGTGCAGCATTTGGCACCTCCTATTTGAATCAAGCTATCCCATCAAGGAATCCTGCGACAGTCACAAATGAGGCCCAATCGCCCTAAACACTGATCTGTTGACTACGTGCCGTCTTACCTTGGGACGAGGTTTGTCACATCAACCTGGCCACCAATTTCACAGTTGGATGGCTGCTCATCGCACTTGGCAACCGCAAACGAAGTCGTTCCACCCGCAGCGGATTGGGTAATATTGCGTTGCCATCAAACGCCAAAAGTCTGGCGCCCCGGTTCGTAGCTTTCTCCGTCTCCTGTCACACAGCAGAGACGAGTCGCCGCATGACTTGACTACTCCT

>DS499594:1525949-1526350

GCGGTACGAGTCGCCTTAAAATGAGCAGGAACAGATGACTACAAGCTCGTCTTTTCTTTCTTTTCAAGAATAGGGATTCTCTTCGTGCAAGCCTTACAAAACTCCGAAAATGGTCGATACGACATTCTCGCTGCTTCGACTGACCCCGTCACAGGCTGAATCCATGGCCCGAGCGGGATTCTGACAGTTTCGATGCAATTCCATGAGCTTCTGTCCGCCTCGCAGCCTTTCCATGGCTGACTGAGGTTGAGTAATACGGCTGTTTCTGACAACCTCCTTCAATGAGCATTACTAGCATTTTGCAGCGTAATAGTTTTGTTTGAAAGTAATTTTCCGCTGTCTTCCACCAACAGCCTGGGCAGAAGAACAACGGTCATCCATATGAGCCCTGTCCAAGGCTC

>DS499594:1556597-1556998

CTTACCCTATCTACTCCCTATTTCTTTCATTGCTGTGTTCTTTTTCTTCCAGGTGGTTTCAATTGAGTCAATACTCACTTCAGTCCCTCGTCAGTTCCGAATTTCAACTTGACCAGGGGAACTTTATATTTGTGTCCAGCAGCATCTAAGGCTACTGTTGGGGCACAATCTGATATGCCTCGTTTCTATTATTAATAACCCTCAAGCCGCAAACCATCATCATGAAGCATGGTCTTTCCCCGCTGGTTAAGTCGGCACTCGGCACGTCACGTTATCCATATGTTAGGCGCCCCGGGTCTCCATAGTTACCCTGGCTCGTCCTAATTGCTAACTATCTCATCTGATTATGAGTCAGAGTTACAGCGGATCTTATGCAGAAGCATATGATTATATTGCTTGAC

>DS499594:1658209-1658610

TTTCTGGGCTTAAATGGCGGTCATCATCTTGGAACCTGGTGATCATCCTTCTCCCCGATTGGTGGTTGGCTCGGGGTGGCCGAACCTGTGTCGAAGTTGACTTCCCCTGTCAATCTCACGTCTATTCTATCCACTACCTACGCACTGGCATGGCTGTGGTAGTTGAGAGGTGGCCTGGGCCGGCCCTTTCCGTGCCGCTCAACTTCGAGTCGTTCCGTGCAAATCCGATGCCAGCTTAAAGGGGTTGTCACTACATGGTCTGACATGGTCTGAGAAGAAGAGAAGAGGAAGAAAACCGAAGCGGAGAAAAGGAGACAAAAAACAGAAGAATCCCTCTTGCTGGTTTGGACCGCAACGTTGCCATGGTGTCGCCAGAACAACCCCGATTTACGAATTAATCC

>DS499594:1810679-1811080

GTTTATCCGTTCTGCCTTTCGTCCTGTTCGCCTCAATGGGCCGCTAAAGCGGTGGAAGCCTAATGCGATGAATGTGATTGAGCCGAATCTAAGCTTAGCCAATCATTAAAGGACCATCGAGTTGTTCAATAACTATGAGAGAAAATCATCATTCTCATGGACAATGGACATCCCAGGACTCAGGTACTGCTTCGCCGCAAGTGTCACGGAACTGGCCGGGAGCTACCCACGGGACCATTTTCACGCGAGCGAGGATCGGATCGATGAGGGGTGTAGCCGTGGATTCGTTGGAGACACCAGCCAACGATAATTCGACGTTCCTGAATGAAAAAGGTTGCATGTAAAACTAACTCCGCACATGAGAGATGTGATCTCTAATGCTCCGCTCCTCCTTATTAGCG

>DS499594:1812815-1813216

TCATCACTTCCTGACTTATCAACAAGAACAACATTAATTCCATCGCCATCATCGTCATAACTAAGAGTACTCTCATTATTACTCATCTTATTCATCTTTGAGCTGTTCATCTACAATTCTGACTAAGAGTGGTGCCACTTCACCGACGTCGACAATCAAGCGTGGCGCAGTCCATACTGTTCTTGTGGCCCTTCAAGTGTTGGCCCTCATCCTGTCCAACTCAAACATTTTGCTTGTGCCTGTTCCTTTTTTGGCTTTTTTTTTTCAAAATTTTCCTTTTGATTTGATATTGTTATTATTATCACTATTATTATTCTGATTTTCGCCGACTGCTCATCTGGTTTGCCCCTCGGTTCACTGCTTCACTGCTTCGCTGCTACAGTACCACATTTTGCCCCCTT

>DS499594:1926040-1926441

TTCTATATCAAGGTCTATCTAGATACTAGGCTAATGCTTGCTATTTACCAGGTGGACACAGACACAGACCGAGGCCTAAAATGCTCGGACGGCATAGTTTCGGCTGGTAATGTCGGGAACAGCTTGCACCCCGCATGGTCAGAACCCAAGTAGTGATAAAAGAGTCGAAACGGCCACGAGACTGGTAGCCAAGATGCATGCTGTAAGTATGGACCCAGGAGATGGGGTGATTGATCATTGTCCAAGTGGCAGTGGTGGATCTGCGATGGTGCAAAGCTGAGCTCGGAATAAATTCCGTGATAAGCAGTTACCAGCTCTCCAGAGTCTAGACTGAATACTTCAATTACACAATTGACTGAGATCGAAAAAAGGTTATCAATGCCCCAGATATGCAGATTCAG

>DS499594:1988094-1988495

CGCCTTCTCGGATTATCATAGGCTCAGTCTTTCTTCCCTTATCGCCATGTAGCTGGCTCTATAGGCAACGAATACACTGTGGCTTCTTGAAAGTCGACCATTCAGAGTCTCGCTCCGCAGATAGTATCACATCCTCGCTTAACACTCATGCCCAAAGTTGGCATCCTCCTGTTATCCGGACTCCTCCGCATCTCACCCGAGGCATGACCGAAATCCAAAGATTGGGCGTGCGCCACCACTACAGCTGACCCTCTCGCTTCGACGGAGTTTTCCAGTGGTTTGAGGTACGTGTGCCAGTCTGCCATTCGTTATAGTGCAAGCTTGCCAGAACTGCCTAGCCACCTAGGAGGCTCGTGCGCAAGGGCTAGATTGAATTTTTAGCCTTACGACTGGCCAGGCGG

>DS499594:2009406-2009807

TGTGGAAGCCCTATGCCCTTAAGCGAGCCTCGGTCACGGGATAATGGCAGTGCGTCAGGTGCTAAGGCATTCTAAATTGGTGGCCTGATGTCCTGAAACCAATTAGATCATCACGTGCAGATCTAGTTCATGTCGAAGAGCCTAAAATAGCCTAGCGGCCGGGTCGTCCCAGCATTACGGGTCTTACGGTACTCGCGTACGGTCCACGGATATGTACCTAACCATTCCTGACGGGGACTCGTACCACTCTGTGGTGTCAACGGAGCGGCTCCCGTGAATGGGGCGTATTGTTATTACCCATTGTTTATCCTGCCCAACTCTGGCTGTTATTATTAACTCACTAGCAGCCAATAGTTAACCCTCGAACTCCCTTTGAGACAACCCCTGTTCGTCCGTATCTT

>DS499594:2049525-2049926

TAAATTTAATCTAGCATTCCAGAGAAATTTCCGTAGAGAGAGTGGAAATCAAATAGAATATTTCTCACACGATGTGGGAAATCACCAAGATAGGGAGTGGCAAGATGCCAAGCCTACCCACTGACCATCCGCCATCTGATACAGCAAAGACAGGAGTATATTACGTAGGGGACTTTTTCTTTCCGTCAGGTCGACGGCCTCAACACCCACTCGGTGCCATCAATCGATTTCTCAAGTCTCGAGGTCAACATATTAGAGGTTAAGTAAGCTCTTGGATTCAGGAACTGCGTCAGATACACTTTTTAATGGCAATTAACCCACCTTACCCTTTTTTTGATAGATACCTTGATTCACCATGTCACCAGAACACCCTTACCGCTATACTCTGTACTTCGAGCCGA

>DS499594:2051589-2051990

CACCCAGTCGTATAAAAATTAAAAGCAAAAAAAAAAAAGAAAAAGGGTCAAACAGCACCTCTTATTCCAGTAGGTGGCAGTGTGACCAACCTAAGCTTTATGGTTTCCCATAGTCTTGGTTTGGTGAAATTCGAAAAACTAGCGCAAGTGAAATTTATCGAAACCCTATGTACGAATGTAAAGTAAAGGGGGGACCATGACGGAAAGCTGGGGCCATCCGTCTCGGACGCTAGTTCGCTTACAGAATTACCTGCAGCCATCCATCATTCCACCCATGGATCTGTCCGACCTTGGGCTGTGCGGACTGAGGCAGTTAATATAATTTTTGAATCTCACATCAACCAGTCCTACCCCTGCATCATCACTGCTGTGAGGAGCGTCATTGCCACTGGATGCGGTGC

>DS499594:2122334-2122735

TTTCTTTAGATTAAGGCAAAGAGCTCGATAATAGGATTGGGCAGCCGAAAGCCCAGCTATTATCAACTCCGTTGTGGTTTGCCGCTCTACAGTCTGCTGTCTTTGTTTTTACTGAAATTAGAGGGTTCATACCATCCCACCCTGCCTGACACGTCACTATGTTCGAGGTACGTTCTGTGCATCTCTGGAATTAGCATACAAATTCCTTCACTTGGTTTACCATGTCCATACGCATCGTTTGACTTTGTATTACGGCGTTGTCTTGTGGTATGACTCTGACGTGTACCAGCGTCGCGCAGCTGACCGCTATCGTCTCCGGATGCACCGTGCCCGGACGGTCGCTTTAACATCCGATGAGATTGTCGAAGTACGAGCCGCGCAACGGACATTTGAAGGCGCCT

>DS499594:2125124-2125525

TAAGAACGGGACGGGGAAAGACGCCAGTCTTATGCCGCCGAAGACTTTGGTCGGACGAGCGCTAGGGAACGAACTGCATTCCGATGCCCACAAGGGATCTCGTGCGTCTAAGGACGGTGTCGGGTTCGCCCTAACAGACACTCCGATTTCTACCGCACCTCCGTCTCCACAGATGTATGTTACCCTTTTCGTTTCCTCCAATGGTTTCCTATGATTTGATGGCGTTTGTCTTTTCTTGATCGGGACCTCGGGTGGTTTCGAATTCTAGATCGTGCAGATTGCAGCCCAAAAGCCACTCCGTTTGGGGTGCCTGATCTGCCGTTCTCGTGGCGGATCTATCATTCCCCCAAACATACCTTGCTTGTATAGCTGAACTGTGTTTCATTTGCTCATGTCATTCT

>DS499594:2170839-2171240

GAAATTATTAATGGGCCTGGCAGGAGCAGACAGGCCAATTAGCGGCAGCCTCATGATGCTGTAGTACCCTTCAGCATGTGAGATGTGAGCTGTCCTCATTTTTTTTTCATCGAAGCTCCCATCGTTCTCTCGGGTAAGATACGATACAGCAGATTACCCAGAGCATCATGTGACCCAGCGGAGTGCCAATATTGCTTTCTTCCGAGAAATCCCGAGGTTTCCTGGCAACTCTTGTCCGCTTTGGGTTCACCGGAGTGATGTGGTTGACAGAGCGGAAGACTGCAACTATTCGGGAATTTGCGGAAAGCCCGTTGTAACGCCCGAAATCGGACTATCACCCGGCTCCGAGACACTGTACAGAACGAACCCCGAAGTCCAAAGACCACTAAGCGAGGGAGAGG

>DS499594:2177826-2178227

AAAAAAATACTCAGGGAATCCTCCTCCACTAGATGCTGAGAGACCAGCTTTGCAAGACAAATGAGCCCAATCCGTCGAGAAACCGCTGGTAAACCAACGGAAAGGCCCAGAACATGTTCTGATGGTTCTTGAATTGACACCAATCATCGCGACACCTGTAGAATTGGCCCGATCCAGCTAATTTCCCTATCGAAGCCCATCGAACACGATGGATGCTCATCGAAGCTCTCTCCTGCTAGAAGCTTCGCTGGAGCTCAACCTCACCGCCCTCCACTATATCAATGTTTTCTCTCGCACAATTTCCTGACTCTCCCAGACTCAATTTTCTCACACACAATCTTCTCACTGAAATTATCTCCATCTTTCCTGTTGTCTCCCTCTCCCCCCGCGCTAGAGACTGT

>DS499594:2185281-2185682

AGCAGCAGTTTGACTCTGTGGCTTTATACGAACAAGCTCAGGCAACAAGGCAGCTTTATTGTTGGGGTGCAAACGATCCATAATTGCCTCCCTAGCACGGCGCCTTATTAATAGAAAGCAAACACGGCAACCAATGCAAATGGCACAAACTGTTTTGAGTTTCGAGGCACGGAATCAGGTGTTCTCCGAGTATCATGACAACGGGTGGTCCCATATCTATTCCTGGAAGTGGATAGACTGCTTCTCTACTACACGGTGGGCTGAGCCCCTCGGAACACTTTCGTCTCTCACAGAGCGCCGGTAGCTCCTGCAGCATCACCTGATCCTAGGATATCAGCCAATTGACCACCCACTTGCATTATCTCTTAAACCATGGCTTTGCCGCGATCATTTATGCCTCT

>DS499594:2264698-2265099

GTGTCATCACAGGGCAGTAATAAATATGGATGCGGGGTCGCGTGCAGTGATCCAGTCAGGTGTTACTCTTAAGGTAGTGTATGCTGACATCTAATGGCAGGTTTGGGGGGGGGAAGATGTGAGAATACTGGAAGTATAGATATAGAGAAGATATGGATTGCCAATCTGAAAGTGTAAAATTTCAAAACAATTATACTGTTGTTATGAAGATACAAGGAATGATTGAAGATTTAGGCATCAACAGGAACAAGAACAGTCCCTATGACTAGAGCAGTGGGGTGGGATGATCAAGGACCTATGCCACATGACGACAGGAAGTGGGACACTGATGCCATCCTTCATCATAATTGGCTTGAATAAAAGATGGTATTGTTTTGAAGTATAAGACCATAGGAGGTACT

>DS499594:2346037-2346438

GACTATGGCACAGATAGAAGGAGAAAATATCTAGCCAATTGTAATTTAGCATTTATCTCCTACAGGACAGTCAAGATAGGAGATTAGTGCTCCGTAAAAGGCATCTAGTAGTCCGTCAGTCTCTAAGGCACAAAGTCTAGCCTAGTCGATTAGCAACTGATGAAGCCTACAAAACTTTGCTGGTCACATGCTGGCAATCAGCTCGGGAACGACGTCCGAATTAGTGGATTAGCTCTTCCAATGCCGGGGACTGCCGAGATTCTAATTGGTGGGACAATACTGATATGCGCAGTGACACCGTCATTTGGTCCAACCCCACTCTGTCTTTGATCATTCCTCCAATCCATCATTCCCTGTTATGGATTGTGGCGACTGGGCACTTGCCAGTGGAGACTTATGTA

>DS499594:2357367-2357768

ATGCTCAATTGTGCAAACTTGCAAAAGTCCTCTGCGAATGCGCACATCATACCACTCCAACTTGCCGAATCCCAAATTACGCCGGGCCCTGATCATAAGACCCGTAAGGCTTCATGGCTCAGTGGAAAGTTATACCTAGAATCGGGTACCAAGCCGCATATACTATTCACCTGTAACTGGATCCGAAATGAGAACGCTGACAATCGTAAACTGCACTTCCGATCTGGTAGTAAATCAACGCGATGTATATAGAATCGGGTTTGAGCTGAAACCATCGAAATGATTATTTGACAGTTTGATGACGGAAAGGTTCAGAGTATCCCCAATGGTGCCGATGAAGCCCGAGCTGTACATCAGCGTACCTACAGTTGTACATATCAAGGTCCTTAAGGTAAGGTACC

>DS499594:2410474-2410875

AATATTACTAGAGAGAAGGTAAGACTACCTCTGAAACACCAGGTCTTAGAAGACTCCATCTTCCACCAAGGTCTGATGTGTACATTGCATATCAAAGGTATCACACCACCTTGGCCGGACTCAAAACCGAGGCCCACCCTCAACACTAGGTAAAGTCACTAACCGCGGTGGCAGTACTCCGTAGATAGTTTTCTAAGATTAGTGAAAGTGTTAGCTGATCCGAGGTGCTTGGTACTTCCATTAGAAGTATTTATCAGACAATAACAAAGGTGACTGATCAGGAGACTGGCTGTAGATCATCACGTGAGTACTTGTTATCTTTGGCACTTTTTTGGTTGTGGCTACCCCAAAATACCTAACCCTCAGGAGCCATTGATGTTCGAATCGGAATGGGACTGTAC

>DS499594:2537747-2538148

GGTGGTGTATCTTTAACCACATTGGCAAGCTGATTATACCAATTCGCTAGTAAACCAGCATCCTCAGCCTTGATACACTTTGATTCCTTCGTCTTTTGCTTCACAGGGCCCAGATTGAGGTGTTCTGGGAGTCGTTTTTCAAAGCGATATGCCCATACCCTACTAACCTGTCTAGCTTCACCAGCGCGTTGAAGTGACTGATTTGCAAACTCTTCTAGTAGCTTAGGTGTCACTGGTATGTTATGATCTCGCATCCAGACTATCCACTGTATTAAGGCTTCCTCCTGGTACCCATCAAGTGCTTTATTCACTGGTTTCCGAGCTGTACGAGCCTGTCTGCCCTTTTTGATGCAATCACGTAGTGTTGAATAAGGAACGCCATATTCACGCGCAATCTTGGA

>DS499594:2738786-2739187

TGACTGCGTGGGTGTTCTGTAGTCTTAGGGACCTACCCTTATCTCAAGCAAGTCCCCAATAATCTGCCTCATCTCCGACAGAGTATTAAGTGTTTCTTTATAGTCCCACCAGCGAATAACTACGGAAACATGCATGTTAGCCCTAATCGCACCCGAGGCTATCGAGAATTCTCAGGCACGATGTTTTATCAGGCCCGGAGCCTTCCGTCATCGATCATGGAACACTGACATCAGTCCCGCATCAGCCCGAAGGTGAGCGAATCTCTCACCGGAAGCCCCACCACAGTCGAGCTTAGCAAACAAAGGCCACCTACCCCGCCGTCGGACCGAAGTTGATCGAAGAGGCTCACCGTGAGCATATTAGGTCAGGAGATTTCAGTCTCTATTTTGTCTGTTTAGTG

>DS499594:2745561-2745962

CTTTCAGGATAGTTTCCATTGCCGAGTCCCACTTTATGGGACGCCGATGGGTGGCGATCCATTCCTCAAGCATCTACTCAACGTGGGCTCGGAAAAAATTCCGACGGATATGCCGCTTGTCATTGATGCTTGTGTATGAATGATCATCCCATTTTGAGATAGGGTGATAGAGTCGCGGACATGTTGCAGTGTTACGGCTGATGGGTCGCATGCTAATGTCGTCTTCCATCCGAACAACAGAGGACTCTGCCCGAAGATAAAAGCTATGAGATGTCAAATTCCCCACTTCGTCAGTCAACCAAAGGACGTGCGTGAGTCAGGTACTTATCGGACGTCCGACCAGTCTAACCTTTTGACAGACAGCGTTCGGTTAAAACGGCTCGTCACGATGGCAATTCTAT

>DS499594:2771755-2772156

AGGATGAGGCTACTGAGACATCTTCCCATGGTGGCGTGCCGCCAACACGCCACCTAACAGGAGTCGACGGAGACCTCGTACTTCTAGGTCTCCCTCGTCGTCAAACTCGATGGGGCAGTGGTACGGATCTGGGATAAATGTTCTTAGACAGACCTCACCTGACTATTGACAAACTCTGCCTAACACGTCAATGGCTCGACGACTCCTAGCCTCTCATGACTAAACCTCAAGAGTCAATGGGCTCTAGACCACTTCTGCGCGGCTGAGTGGAGACAACAAGATCTCAAAGAGTCAAATATGGGTCTTCAGGCAGAGTTTTGTGAGCGTGGTGTAAGACATACATGATTGGACTCCACACATGATCACCTCTTGTCGGAGTTAGACTGCTGGTGCAGAAAGAC

>DS499594:2841625-2842026

GGTTGCTGGGCGATAGACTAAGGGTTCCATGGATGGCCGAGCCCTTGCAGACCCACCGGCTGGTCAGCTGCCAAACTTGATTTTGCGGTGGAGTAGAGAGCGCCTAGAGAAGACCAAGAGGCCAAGAGGGCCGCCTCTTCGTTTCTGGTGTTCGAGAAGAAAATCTTTCCCGGGTAACCGCTGAGAGCTGGTCATGCAACTTCTGATTTGACTCGCTAGAACAAATTGAACGACATGATAAATAAATTGTGGAACAGTGTTCCACAATATTAGCATATAATATGAATTCCAATGACTATCACATATTCTCATATGATGACTTGATTATCAAGAGGAGACTGTAGTCTAACTGTTACTCCTGGTGATCAGTGGTCTAGACTCTAGGATTCAACCGCACGCAG

>DS499594:2844091-2844492

AAACAAATTCAAGCAAGATCGTCGTCCCATCCCAATTGCTCATAAATAGATTTCTCCGGCGTCACAGGCACCGCCGAGACGACCGGAACAGTCAGTCGGGCGCTACTCTTCACTGGTGTGGTGAGCACAGCTGCGGGGGAGGCGCCAGGGATGCTTTCTCGGACGGCAGTGTCTGGCTTCGGCGGTGTCTCGCTCACCGTAGATGATTTGAGGCCGATGGCCTGATCGGCGGATCGCGGTGCAATCGGCGTGGACGGCGCGACGTCGGCGGATTTAGGGATCGGACGTCGGGGGACCCGGAATAAGTTCTTCGCTACGCCCGGGGATTTGTCGTCCGTCGCGGTGTCCATCAGTCCCAAAGACTGCGCCGGCCGTCGCGACGGCGTCTCCTGGATGGCACC

>DS499594:2928549-2928950

ACCCTCCATGTACTGGAAGGTTGGGACCCTTCTGGGGAATCCCGGGCGAGGATGATGAGTCGAGGAGTGCGATGATAGGAAGCCATATAGTAGATTAGACGATAACATCAAAACCGTGGTTCATAATTCCCATTATCGTCGACAAGTCTCGGAGAAACTGATGAAGATCAGGAGGACACCTCGTTGATACTGTCCTTCGAGGTGATTGGGGGATGAGGCGAGCATTGAGTGGTTGTAGATATGGACTTGCTTTGGACCAATCAGCAGCTCTACTGCCAGTCCTCAGCCTGATACGAAGTCCTTTAGGAATTTCTTCTTGACTACCTTACCTATCCTCCCCATCCTTTAAAGGCAAGGATTGTGATATGTTTGGTTGCCAAGATGAATTCCTCTACATTGTA

>DS499594:3023315-3023716

GACCTGTCTCATTCAGGGATCTGCAGCGTTGCAGTCTCATTGGATGATGGGCGCCAGCAGTGCCAAAGATAGCAGCAGCATACGCAATCTTTTACAGCCACTCCGCCTTTTTTCATCCCGTCAAAGTCTTGACCACAGGGATTCACCTCTTATTTGGAGCTACGTATGTCGCAATGCAGGGCTTAGTCTAGGCTCTCCAACTTAGGAAGTGTCTCTCCTTCCCGCGCCCTACGGGCCTACTGACTGGTCGAGAAGATGCCAGAAGCGGATCGAGATGCGGTGGCCCTCCTAGTCTGGTGGGTCCTATCCATCCTGCGGCCATTAATATTACTATGAGTCTGTGAATCCCAGACAGGGCTGATGGCCGATGGCGACTGGCTGTGGCTGATTGTTTCTATTGT

>DS499594:3103497-3103898

TCCGAGTCTTAATACTTCGATGACACGCTGGCAAGCGCCTTGCCAGCTGTCAGTTCGTTTTACCCATGACAATGCCATCATACCCGTGCTACCACGAAGCGCGGGGGCTGAGATTCCAGTACCACCGCTTCGTAGAGTCTTAGGCTGCATTTTTAGTGCCAGAAGCCGTCTTTACAAGCGGGACAGAATCAGGCGGAAACACCAAGTTCATCGATGCTTGGCCATCTTCATCGCTGAATTTCGACCAGAGAGAGTGCTTTGGTCACATTGTGAACCCAAAGAGAGGCTTGGTGGCAGCGACATCCACCGGTCTTGTCACAGTGTCCCTATCATATCGTCTAGCTGAGGTTAAAAGCTGCAAGTCAAGGAAAATCAGCAGTCGGGCTATGACACGTATCGAG

>DS499594:3202084-3202485

CTGTGTTGGCTGTCCATCCCTTTGAAGGAGTCACACTGCGCCTTCGAGTATGGGTCACGAGTCACTGCGTTGTGGGCTCAGGTGAGAGGGAGATCGACCACTTGCACTACTCCGTAGTACAGACACAAATGAATCACGCTCCACCGGCTGATAAACTAGCAGATAGTCGATCCATCAACAGACACGGAGTAGATAACTCCTAAACTGTATCAGCCTATCCTGGTGATAACATTGGCTGCTTCATTTCAACCCGCGGCCGCATTCGGACTAGAACGGGATCCCTGACTAACATCGGTTTGTAAGTTACCTTGTCACGTGCCATTCATCGGTGCGACATCACTATCGTCGTCATCCCATCTTTGATAAGAAAGAACCTGATGAACCCAGAAAACCGGTACAAC

>DS499594:3215750-3216151

GGCAATTTCAGTGACCAGTCTGTAACTGTTGTTATGTGGGTTTCGATTATTGTACCAGGTAATCCAGAGATAGTCAGTCCATACTAGCGAACTCAAGGATACAAAATGTTGACTTCAGCAGTCTCCCCCGACAGTTCCGAATGGTCGGGAAGTTTTTGAGGCGATCAAATAGTGTAGAGCTGTTTAGCACCTCACACTGGGATATGACTTCAGCCAGTCAACGTCAGATAATCTGAGCATAATTACCGTAGAGAGTAAGTAATTGCATGACAGCAAGAAATACCAAGGTAATATTGACGAAGCCCTGGGTCTGAAGCTGATCGCGGCAAAGTCTCTTCAGACAGTCTTGCGGTCTGCCAACTGCACTTTGCCTAGCCTGTCCACGACAAGCACTTAGTGGT

>DS499594:3220322-3220723

CATGCTTTTCCTCCCTGTGATACAGTAGTACAGAGAAACCCTGTACAAGTATAGTAATTGGCTTCTCCTGTTCTCAGTTAGTACGTTTGCCCTATCTATTTGCAGTTAAACAGGGCCCGGATTATCCAACTATCGGCCGCCATCCATACCAGGCTGCCCAAATTGGTTTCATCTGATACACAACCATACAGAGCGATACCTGGCTACCGTACGCATGCATTTTTCGCCTGATCCATCCATGGACAATCCGACTCATCCATACCGCCCCAGGCAGAGGCACAGGCTCAACCAGCCCTTGCCATACTCAAACATTTTGTACTAAGCCGCCAGCCTGTCTTCTGGCCTCCCCTACCTTGACTTTTTGTCTGGTCACAGTCTGCACCGACCTTGGAAGCCACATT

>DS499594:3253706-3254107

CTTTGATAGTGACAAGCTTTCAGTTTTCGCTTTTGCATGTGAAAAAAGGGTTGAATTACTCCGTATCCGCTCTTCAATCATCCGATATCCAGACTGAGCGTCAGGCGATTCTGGGCGAAAAGTAGTGACAACACCCAGCCGAACCCCGTTTAGAACGTGGAGAGTGGAGTCCTCAACTTTTCCCAAAGACGCCAGTGTACTGTGGTACAAGGTTGCAGTCAAGTCTAACACCAATACACACACCATGTCTCACTGCGTGGGTTCGTCCTGTGCATCCTGCACACATGCAATAATAGGTACAGCTTCTAAAGAGTTCCTGAAACAAAGTCCATTGTCTCTTTATCATTATTACTGTCATGAATTTCCGATGAAAATAACATGATTTTTCCCAGCATCCAAGT

>DS499594:3303579-3303980

GTGAGAGAAGATAATTATAGATGAGCCACCACTCAAAATAGGGAAAATTTCGGATGAGGGCAAAATGAGGTCAGAGTGAAGACAAAGACCGAGCAGCATAGGCCGGGTGACGTACGATAACTTGCGACAGGCGGGCGGGATCTGCTTCTCTGCCGGATCGGCGATTGCTCGGGAACGCCTTCAAGCTTGCCAGGCAGTCGCTGATTAATTAACACCCACAGCAGTTTCTTCTCGCACGAGACACACCAGAATAGAATCTGCTAATCGGGTACGGGACCAATGAAACGCTCTACTTTGTGCCCCAACTCCTAATGGCATGATTCTGCATTCCTATGAACCCGACACCACCCAAGGAACTTCTCGAGCACACTGAAAGTAGAATAAGCAATCAATAAACTAAT

>DS499594:3321971-3322372

GCGAGGAGATTTGCCGTGTTGCAGGACCAAGCAGCAAGTGATACGACCAAAGATTGCAGCTAAAGGACTAGCGGAAAGGCGAAGAAAGGCGAAGTGGGAAGCTAAGGGCAGGGAGGGCAGGAAGGACAGAGAACCAGAGACAGAATGGGCCAATGCCAGCGTCACGTCGAAACAGGTCGATAGTGGAACCACCGGACAGAGCACGATGGCCTGACGCTTGGAGAACCACAAAGAGTTGAAGTGGAATTTTGCAGGATGGGAAACACCAGTGAGATTGAGAGAATGATGGAAGGGATGTGTCTGGGTGGAGAGCTGTGGATTCTAGTGAGTCGAAGAGTATGTAAGATGATGATAGCCTGAGCGCCTGAAAATCAACCCAAAGGAGTCTAAGGCACCCAACT

>DS499594:3371166-3371567

TCTACGACGCACTGTCTACTATCTACCGTCTCACCAATCAGCTTTGAGGCATTGTCGCCGTCGATAGGTGTTCCGAATCAGGCCACCAAGAGCTTGAGCCATGATCTCTGGCTAAATCAATTCTAGAGTTTACCTATATTTTCTGTAAGCGATGTTCAGCCACAGCTAAATGCAAAATGGGTTCTATGAGATTACCATGTGGCAGACATGGAGGGCAAAAAGGGAAAAATACTAGTGGCATGCCCGACCAGGTACAGATACCGGGGTGGCGTCTCACCGCCTTTTGGGAACCCAATCGTTGATGTTCGTGACGATCCACTGCCAGAAAAAGGGGAATTGGCTTCTCAACAGCCTTGGCATGCACGAATCCGTATCCTTGCCTGACAGTGCCAACAAAGGAA

>DS499594:3398501-3398902

AAGTTCTCCCGGTGACAGCCCGGTTAAAGATGTCCAAAAGGTCATGGACAAGTCATGTAATTGTCGCTGCCAAGATCCATTACCTGATGGTTTAATAACAATTTGCCTTGCCCATCCCCCTTTCTTTGTTTACTGTTAGTCACATCGATGGTGATTGGTCAGGTGACTCCTGATTGAATCGATAAGGCGGCAGAACCCCGCCAAAAGCGGTGAGTCAAAGGAAGGATACTGCCGGTAACCCCCCCTAGGCGGTGTGACGCGGGAAGATTCGAGTATGAGCTAGAAGGTTCGATAGGCAGCACAAGGGACAGCTTGGGCTTCAGGAACAGGCATAACTAAGAGTAATTGCCCCAGATTGAGTCAGGCTCTGGGAGATCGAGAGCAGTATAAGAAAACTTTTG

>DS499594:3460408-3460809

TGGGAACTGGGTCTGGTATCCGCCATGGTATGACGGTGAGCTTTCGCCTCTCATAGGAGCTCTCAGGCCAGTGTGGACTGCGGTCGAGGAAATACAGCGCCTGCATCCTTGTTGGGAAGGAAACAATCTGAGCGCCCTGGAGCACCGTGGAATTAGAGAGAATCATGAGACATTTCTTCAAAAATGAGGCCCATTCCTCCCATCCGCCACGGTGGGACGAAAGTAAGGGACGATCCCAGTAGCGGAGTCTGGCGGCTCTGATCTCGTTGTTGACTGTGCCACTTTCCCGAGGTCAGTGGCTGTGGTAAGAAATCATAGATAGAGGTCCCCGGTATCTCCATAGAAGCCAATCTGGAATTCAACGGATAGGAGGCGAAAGCAATCCTGCTTATCTTGGTTCT

>DS499594:3465553-3465954

CATCGCCAACTTAAAAGGAGGGCATTCCTGCTTCACATTTCCGCGGAGCTCGTTGTCAGCCCTCGCTATTTTCGTCTATCAGTCTAGCCTTTGTATGAAGAACGATGTTGCTCTGAGGCTGTGACACACGACAATGCTGGCACTATCTGTCTCTTCCGAGCACATGGAAGGTTCTGAAGGCGTCGTTTTCCAGGAGACTGTCCAATGAGATGCAATCCGGTTATCAACACCTCCGACTCATCCTCGCCCATGGCCAGGACCTACCGAGAACCTCACACCGGGCCCTTTGTTCCTGGTTCCATGGTCTTTGCAGTCATTCTGTCGATCAATACGGTCGATATGGCTGCGGGATCAGCAAATACCAATACAGACGCGATCTGTGTCCTGATATCAGGGCGGTC

>DS499594:3476686-3477087

GTCATCACTGCTACAAGTATCTCGCCACTCAAAGTGTGATCACGCGACTGGCCGTTCTCTCCATTCTCCAGCCTCGAGATACTATGTACTGGCACTGCAGATCAATTCCCTTACTTCCTACAATAGTTGCAGAGCTTCAAAGAATGATTTCTTTCCGGTGAGCTGGTGACTTGAATCCTGAATTGTGGATGGATTGTATAGAAACTTTGAAGCAACTGGCGATATTCACACGAGTGCTCCGACGAATGACGATGGGTTCCACTTCATTAGCATGAAGTTTACATACATCGCGTGCCTACTGTGGACAAACTCCAGCCTCGTTCTGACGCTGGTATCGTGGTATTGTGCCAAGTCAACTTAATAATAATTACCAATTGCCCACTTGTGATGATTGTGTGGCC

>DS499594:3528933-3529334

TGACGGTGTAGTACAGACTATGGTTGAGAAACAAGGAAGGCAAGGATACCTCAAGCCTTGATGAAGCAGATGCAATCAAATACCCCAGAGTACTATGATTCACACCCAGAGTATTCGAATACTGTGAGGACACTGCTGCACACGTGGTGTGATGTCTGTTGCGTTGTGATCGTTATAGCCCGAAGCGGAAAGTCGAGATCTAGTGGCACCGAGGATGAGATCTTGGGTCGTAATTATATCCAGTGAGTCATCTGCATCAGAATCAGGCAGCCGGATAAGTCACAATTTGATATAGTATGTTATTATTGGTAAATACAGACGGAGTATCCGCATAAATCCCTACCTGGTTCAACATAGAGAGGAAACTCAAAGTGATGCTTGTACTGCCAGGTCGACGGCAG

>DS499594:3608878-3609279

CTCACTCTTTCCTCGCAGATATCGAAAATGGGCCATTTGCTCCAATGAACCTATCCCCTTTCGGTTAGTAACCTTGTGCTGGAAGTACGCCTTCCTGCTTTTGGACTTAGCCAAGTCGACACGGTGTATGCTCGTCACACTCATGTAACGGTTCTGCAGCGCAAACGTTCTGGTCACGTTATTCGCTTTTGTCCATTTTGTCTTTTTGTGCGGTACATAAACTAGAGGATTAGCGCACTTTGTACATGATGTTCCCGACATGTCACATTAGGCCTCGATTGGTCAAAATAATCCCTCTCTAACCTCTGGTTCGCATACTCTTAAATGCAGCTCTAGTCTCTAGACGGACTAGTTCCTCCTGCAGTGGATGTCTGACGAGTGTCTGCAAGGACCGTTTTCCG

>DS499594:3631380-3631781

GCACCAGGCGCCATTCAATTAATTATGCGTGGTTGTAATTCAAAATGGGGCGGTGCGTTCATAGGGGTCATCAGGGTCATAAGGTACACCGCAGGAGTCGTCACCTCACCCAGCGGAAAGGGGAAAGTGACTTGGTCATTCAGTCGACATCCTGTAAAGATGAACTGGCCTCTTGGATCCTTCCCCCTTTTCTTGTGTTATTAGTCTATTGTTTGAAAGTCTTCCTAGTTTCTTGATACTCCGGATCTGGCAATTGTTTAATGGATACTTCGGAGCACTACGACACTAACATCGCCAACTTTTGGATGTTGATTATCGAGAGTAGTCGACTCTCTTTTACAACTCCGAGAGGGCCTCCGATGATGCCGTAGATGATCGAAAGATGTGGTGCCGAAGGCAAA

>DS499594:3633391-3633792

AGCTACAGTCCTGATATTCTTCGTAGTGTAGACCGCTCGGTCCTGCTGTCGCGGGGATACACTTGGGTGTCGCAGATAAGTGGTTGTACAATTGATACCAAACTCGTCAATTCGGGAACCTCGCCGATGGCCTTGAATGGTAAATGGGCTGAAGTGAGGCAACCAAGGCAGCTATCCTCGTAGCTTAGGCCGCATAGCGCCCCAGCCATCCCATACGTTTGGGCCAGTGGCAATTCAACCTGATCTCGTGTCATATGATGTTCAACTATGTTAGAGCACCGGCAAACGCTACGTCTAAGAGTTGGAGAAATTAGAACTCGAGTCAATTACGAAGAAGTCTTTAAACAAAACACAATGGACTACTGAATAAGTGCGTACATATATAACAAAAAAAAAGTCTC

>DS499594:3637304-3637705

CTCATGTCCTGAACCATAGTCCAACCGATCCCGCGATCCCAGGGAGTGGACCAAATAAGACGATACTTCTTCTATGGCGTCCGCATTCTGTAGGCCGATGATTTCCCTATGCCACGTCGCATTCTGCGCAGCCACATCATCGAAGAGATCGCGGAAAGCCGGATTACCGAACCTTGAACCGCCTTGGTCAATGGAAGGGTGTTTGTCAACGAGTTGTCTAATAGTATCTATCACAGATAGGATTCTGGAGATGTTTGGGGAGGCAGGTTGGTCTTTGTTATCTGTAACAGCACGTCCTCTTACTGAATCAGAGAGGCTGAAAGTGAAGGAAACGACGAGAGTGTAGGTTGACGATGCAAGAAAGGTCTCATGATCTTTTCGTGATAGGATACGCCGAACAG

>DS499594:3637852-3638253

CTGGAGGAGACGACATAGTCGCATGAGAAGGCATCATGTAAATGTTCACATCAAGAACGATCTGGTTCTGTAGGATGCTGGCAAATGACCTCGAAATGTTATCACTTGATAATGAAGTGATAATACCAAGGTAGGTACTGAGCTGACGAAATCAAGTGGTGTCACGGGTGCTTTCGCCGGCAGATCGGGCGTATCAACACACCGCCTGCTAGAAAAGGACTATGACAGCATTTCCATAAGCAGGCTAGCCAACCTCTCCAGTCACCATGTGATCAGCCAATTCTCCGTAGACTAGCCGCTAATGGCTGAGACCCTGTTTGCGTGAAGACAAGCTGAAGGAAGCAGATAGAAGGACAGCTAAAGTTTGGTGTACAGGTGTGAAAGAGGCAAGAAACACACGG

>DS499594:3732201-3732602

ACGCGGGTCGAGACCCGATCAGCCCAGATGGGTCAATGTCAAGACAAGTCAGTCCAGACCATTTCAACTTCATTTACACCGAGTATGCCGCATGAGTCAATTTTTTTTTTTTTTTTTTTTTTTTTCAATCAAACAGTAAATAATAAGTCATTTGACTTTGCTCCTAGGAATGGTGTGATCTTAGTTGAAACAGTGATTAGGAGGTGGACAGGTTTGAGAGAGCATGTGACCAGCCATGTGCATGTCACGCGGTCTCGCTTCCATTTGTTGATACTCTGGGAGACCCTGATCAATATCATGATCTGGATATCATGACTCGAAATCTTAGGAGGCTAGTGCGGAGTAGAAATTGGTAATTTGCTATTATCTATTACTGCGCTTCGGATCTCCGTGGTCCAGAC

>DS499594:3734887-3735288

TGAAGATCATGATCCAGGTCAGATGACCTTCAACACTATGCCACTAGTACTCAATAGTCCAGAGTACCATGGGAGAGAAACAGTCAGCTGACTTATTCAAGTACTGAGCCTCGCAGGCTACAGAGCATTAGCTTGAAAAGTGATCTTTCTATAAGCGATGAATGCACTTGAAATGCTCATGTGCTGCGAACTTGGCCGGCCGAGTAGTTCTGTGTACGTCGAAGATAGAGTCCCCGAAAAGTCCGCCGTCCAGTAGGGGATGCCGAGAGCCGGCCGGCATGGTGGAGCTGCTGACCAGCTATCTCCGAGTCCGTTCCGCTTCACCATCGCCTGTTTCTGCACTCTTGCACAGTCCAGCAAGAATATCATTTAATTCTTCTAATCATGTGCTGACAATTGAC

>DS499594:3755199-3755600

CATGGGCTGGTACAGAGTCGACACAGTTCAATTGTAGCGTCATCGATCATCAACCTTGATCCCGATGCGCACTAGGCATGAATGTGCCGGGCGGCTTTGCATAAAATGTCTGTTTCAGGACTAGCGTCTCCCAGCACTCGGCTCTCGGCATCTCATCTTACAGAACTTATCGTCGTAATAATCAACTCCGTTGCATTGCTGGCTGCAACTGCTGCAACTGCAAGTCCTGATCATCTAATCTCCGCAAGAGACGAGACAGGTCAATGCATACGGAGTACGGAGTACATGCATTGAGATGCCGCTTATCTCCGTTAGGGATTATGCTGTGTAAAGTTGTCAGTAAATCTTTCTTGATTATAAATCACGTATAACACTATCCCATTATCCCATTATCCCATTAT

>DS499594:3805549-3805950

TATGATCAACACCCAGCACATACAATTTCCATCAATTTTCAACAGCTCTGCATAGTGAACCTTCCACCTTCCAATCGTCAAACCGAATAATTTCCATCATGGTCTATCTACCTAGATTTGTAGTTCATCGTAAGAAAGCAGCGAGACTCGTATTGACTTACGTATGTGCCGGAAACTTCTATCTGCCCACTTAGACTACCTAGTGTCTCCATTCGTCGGCTTTTAGCGCGCGAGTCAATCAGTTCACGTATCCGATACGTAACTCCGGGCTGCTCCTCGTTCGGTTCGTGTATTGTTATTTCGTGACACTGTGTCATTCTGTCAAGGTGTACTGTACCGTACGTTGTTGGTTCTGATGTGTGAGTCGGTGAACTGATAAAGTACAATATTATACAATTATA

>DS499594:3809363-3809764

TTCCATCTACTTGAGTGTTGTCCCTTTGGATCGACCGTAAGAGTTGATAGCTGCATCAATCTGTGAATAGTAACCTGCGAGTATCGTTCCTTGATTGAATCACAAGCCACAATTCGCCTACTCAGGATTCATTGGGAGACATGTTTAAAGTACTGTGCAATAGACAGGCTTGGTGATCTCGGGTATCCGGCATCTTCAAGATCAGGTTACACGCCAGGCAATTGGACCAGTAACCATCAATTATTGTCGACACAGACATCAGTATCTGTCTGTCTGGGACACCAAGAAACTACTCCTGTAGCCAAGCACGCGGGCAATTAATGATTTCCCAACAAACCCCATAGGGCTGCCCATCTTGCATAAATGTCTCAGCCAAGATGGGTGACCCCCTAACTTCCAGG

>DS499594:3812086-3812487

GCAAAGTTCCTGCTTGACAACAACCAGTTGAGACGGGAAATATTCAATCGTATTCCGTATGGGCCTCTCAGACCGGACAGGACGTCTGCCAAGACCGCCGGACTGGAATCCCAAAGATCTTGCCGCGTAGACACACTGATGAAAGTGATAATGTGGAAAGGATCATAAATCAAGGCGGTGCGCTTCCGATCCCTGATTGGGCGCGGGTGGCCGTGCTGACGCGTATCGAACTTGACAAATATCCATACGCAACGGTCTGAAACGCAGGTACCGCCCCACTTTTCACTAATGAAAATGGGTTTTCATGAGCTGCACGTAAGTTCCATAACCTAGAACTTGAAATTTCCCTAAGATAGCCCTTGTCAGTTTTGGTATACATTACTTCAAATCCTTTTTCCAAA

>DS499594:3887698-3888099

CGTCTCGCGTAACAATACAGTGAAAGTAATACAAATTCATCTGATAAAATTGCTGCCTAAGGTTGCACCCGGATGTCGAACATTTGCCTGCTCTTCCACTGCGATGCAGAGAAACAGGTAGCGAAAGAGGTATAGCGCGGAATATGTCGGAGTTAATCGTGCGGTAAGGGCTCGTACGACACAGAATATCAGAGAAAATTACCAGCGGCTTACTGGAGGCTTCCTTCGGGTATCTTAGGTAGGCACTCACAGTGCCCAGCCCAGAATGCAAAATGTCAAGTGACTAGGCTAAATTTTCTTGCTTCCACCAGTGATTAGTTCTGTGCTCCATATGTGGACAGCACACAATCTGGACACGGCCTTGGGGAAGGGACGGCAATAGGGAAGCCTATTCTTACCCA

>DS499594:3940324-3940725

AGGCGGAGGTGGGCAACAGGGCCGGGTTTGGCCAGCCTCTGAAGAGATCGATTGGTTCTTCGGGGGGAATTACACCCATGTCGAGATTGTCGACAGTTGTCGCTTGCCACTTGCCAGTATGTGTACGGAGGAGACAAAGTTCAACGCGATGGCTCGGTCTCAGTCGATGACCTTTTTTTTGGCGTTGTCGTTGAAAATTTCAGCGTGGAGGGACAGGGGACACGTCAGTCTGGTGACGTTGGCCGCCGCCATTTGAAATGACCGAGTGTCTGTCTGTGACCAAGGAATACGCATGCACTCTATTTTCTCCGTTCCTTTAGTGTCTGTCCTGATTTGGATAGCTTACGACCAAGTACAGTCAATATGAGCAACGTGAGACTAGAGAGGAATTGGCTCCATTA

>DS499594:4069382-4069783

GGCATCGGATCGTTAATGGCTGGTAGTTGAAATCTCACAGAATCGAGCAATATTCTTAGAATATGACTCCATAGTTAGGCATCATCTACACAGTATACCGTCGACGATTCGGGATGCTTCGCGCCATTCTCCGTGCTGATTAGTCCATCTGTCATCATAAATTTTCACATTAGGTACTCCTGCTTTACAACCTTCTAGACTTTGCTTAGTGGATCTTCGTCAAGGTGGATCCGATTTTCGGGACGATGAGTTTTCACTCCGGCAGCCATGCAGACTTAGAGGAGGATGGCGTATCCTCATCGGCCTATCCCTGACGTGATCTCAGATGGATTACCAGATTGGGCCATAGTGAGTCACACCACAGCACCAGCTCATCGTCAGAGCTATGCCTACCTTGGCTT

>DS499594:4162904-4163305

AGGTTAGCGGGCATCGGTTGGGATCTGCTTTATCTGAATCTGGCTTAGTAGTGAGTAGATTTGGTGTCAATGTCTCGGATAGTGATCCCCTGGATGAGATGGTCCGCCCCGGAGCAAGCTACTGCTGCGTGCATACCGAGTTCCGATAGGGCTCACTCGCAGCTCCGTAAGCATGCGATTGCTTTGCAATTCGAAGCGAACGACAGGTCCGACTAGTGCTGACATAGGTCTGATGTCAGGGATGGTTGTATGATGTTGACACGGCATGACCGCGATGGGCCGCGGAATGGCACGCCTATGGGGCAACCATGGAGTGTCGTCCAAAGGAGAGAAGGATTCTCTAGCCGGTGACGCTGTCACCCTGTACGGTGCATATGCCCAAAAGAAGTGAATCGGACATG

>DS499594:4164906-4165307

CATCCTATTAATAGTATGACAATTTTTGTACCTGGTTCAGTCCAACGCAGGAGAAAAGGACCCCCTGCAAGGCAAACATGTGTAAGTGAAGTATGTGACAGTTTCAGATGGTAAGTGCCACACGAGGACGAACCGGAACGGGCATCAAAGCAGCTAGTTGTAGCAGGCGCCTGGAAGCAATGCACGAGCCGCCTTGCTGGACTCCGTACCTTCCCATAAGTCTACAGGATGCATAGCAATAATGTTTGGTGATTGCCTAGATTTGTTGTTGTACGGGTGTTTTTTCCGACTCTCTTCTCTCCCGATTTTGTGATGGTTCTGCATAAGGAATGTATTCCTAAGATAACCTGCCTCTTTTAAAGTTGTAATCGAAGATCACTGCATACTTCGATTCATCAAAG

>DS499594:4177998-4178399

GGGCCGTTGGCTCCACCTTCAAGCAATGGGCAACAGGTGACAGCTCTATTCAGCAGAGACCGACAGCTGCAATGTAATCTTATCGAATTTGGTCCAGTACTCTCGTCAGTTGATCACAATATTATCGAATAAGTAAATAAACTCAGGATTCACCTGCTTGAGGTTAGCTCGTTCTTTAAGTGACAGTCGATCTGTTGATTATATCAGCTGCTGGCACGTGATGGGGATCGATTGTTCTGGAAGGCGGTGGAACCCACTCGTGTCGTCATGGGGCAACAGAGAGGCTTCTCGACACTGCGAAGAATGGGCTGATGATACCCAACTGCAGCTATATCTAGCTCCGCTGTCATCCGCGCAGGCTGTTTCTTCTCTTCTTTTGTGTTTGTTATCACCATCATTAT

>DS499594:4230949-4231350

TGATCTTATGTATTCGATGGGTAACATGATAGTCGATGATGCCTTCGAGGAAATGGATACACTGCCTAGTATAGAAGTTTTTGCTGCCTATGTTTATGCCACATGACAAGGACGTAGTGTATCTTTCGTAATCATGTAGTGGCTTCTGACGGAATCCCACTGATCCCTCCAGACAAGTCCAGAGGAGTCCACACACCTACAGATATGACTTGATATGGCCAACTAAAGGCTTCATTTTACTGAATTTCCCAGAATAGCAACGGAAAACCGTCGAAGTGATGTCGTGGCGCGTGGTTGGCAGCCCACTGCTTACCCCACCACTGTGGAACTGTTCCAGATGTCTCATGTCGTTTGATTATTGAGACACCGATCTGTCGTCATTATATGGAGACTGCGAAGAG

>DS499594:4252926-4253327

GGAGGGACATCGCCGTAGTTGAAATAGTCAGCTTCGTTGGCATCCACTTTGATGTCTGTGTCATCCTCTGTCAGTTGCACACGGGAGCCCGAGCAACGGGCGATGAGCCAATTGCCCAGCTCGAGACCAGGGTCACGGCGGTATCGTTTTTCCGGATGAGTCTCCTCAAGAAACAAGAAGCCGACGATAACCCCAAAGACCAGGACGACAACACAGACAAGGTTGGGCAGCAGAAATGGAAAGCTGTCGAAGATTGTATGGCGCTGGAACAATCCGGGATAATTCTGGCAGGGCTGTGCCAACGCACCACCCATCGCTGGTCCTATGATCGACCCCAAGCACCAAACAAATGGCATGATCGAGTAAGCCCGTGGTTGATGTTCCTTGACAGTCACGATCTC

>DS499594:4275008-4275409

TATCATATTCATATCGATGATGTTCCCGATGACAATCCACTGTTCCCCCCCTCCGATATCGTTCAAGAGCTGATTCATCCCAACCCAATCGGGAAGGGCAGCGCACGTCCCCGCACCCAAAATTAGACCCAATCTGGCATAGTGCTGTCACCGAAGGGCGCGCACTAGCCAGACATGCAGATGACTCAGGTATCCTTCGGACTTCGCGCGCTCCTCTCCGAGAGGGCAACCGCCTTTCCAGGCATTGGGAGCCGTCATGACAGAGACACGGTAGCTCTCCACTGCCGTCCATAGGAAACGCCGTGGCTGCTCAACCCTGGAAAGTGTGGAAACAGGTGGCCCTTGACCATGCAGTTTGGCGGGGTCAAAATTCTCGGTCATGCATTTCTAGGCCGAACGTG

>DS499594:4416008-4416409

TGCTTTGCTCGGTCAGTCTGTCAGGCAGAGTCAGACACACCTTAGTAGAATTCTTGCGTTTCTAAAATTTCTCCCGGCCGAACTTGGGCACATTCATGCTCACTCCAGTCACAAGTGTTACCACTCATGGCAGAACAAAAGACCGTGGCTGAGGTCATCTTGCGCCTATCATCGTGGGCTGATACCGTGCAGGATGGAGCCTTCTCCAGTTTTGTGATCCACCGGACCGGGGTGTCGGCTGGCCCCTCCATCATCCGATCAATCACCGTGTCTGACCACTTTCATCCCGTTAATGCAAGCTGACCTGTGAGCACTCAGTTCCTACCTTAGACACCCCGAGTCTGAGACAGCGCGCTGGGTCATCAACCAGAGGACAGAGCTAAGATCCTACAGTCCCAATC

>DS499594:4474550-4474951

ACGATTCACAGTCCACCATTCTCGAGTCAAGACGCCCAGGAGGCTTAATTGAAACGATAGAGTCGAGGGTCTAGACTGGGCCGAGTAACACCGCATGTGAGTGGACGGACTTGATCAATTCCGACAGCTATTTTCTCCCGTGTAGGTTCCATAATCATTTACCCAGATGGGTATGTCGTTCGCAGGTCCGTGGTACGAGGACCGGAATCCTGCCGAGCTCTCCTCAGCAGGAGGATAAGCTCGGATATCTGCATGATCTGTGGATCGGTAGACAATCAGGCGAGACTCGGACGTTGGATGGATTATCCCACGCAGTACCTACCTACTTCGGGCCTGCTCAGATGGCTGTGATCGTGCTGCGCCTCGACCGACAGAACAAAGCCAATCGCTTTTAAAAGATA

>DS499594:4513213-4513614

TGTCATGCTGGAGCGACCGTCCCAGTGTTGGGGATTAGAGGCCAAGAAGTGCTTAATCTCTTTCAGGTCACTGCACAAAGGATAGTTTCCAGAGAGATGAGCATAATCATAGGTTACTGTCATGTGCAAAAGACTCACTGCCAGTTGAGAACCGGAGTGGCTGACTGTCGGATTGCTTTAGCCTATCATCTCGAGCCAGATTTATGCCCCACATTCCATAGAGACAGATACTGATAGGTCAGCTCTATTCGAAGCGTTCCTGGATGAAGGGCCCAGGAGTGGCTGCCGGTATTTGCTGGTGAGTGAAGCTGGGTGGCTTGCAAATTGTGATAGGTGCGGCTGGTCGGCCGGCGATGGACTATGGATGGGGACTGATTTTCAAACTGCAGAAAGCCTCAGTC

>DS499594:4608434-4608835

ACCAAGCAGAATTGCCACGCCAGAATGGTGTTGTCTTGTATTTGGGCAAGATCTAAATAGTCTATGGGATAGAATCTGTGACAATCACTAGATCGGTCCACAACTCAGGCCAGAATGTACCACTGCAACGGGTGGTTTGACTGCCTCTAGGATCAAGACAATCAGGGGCATCTCCTGACTGAACTGTATGTTATTGTAATCACAATACATAAGTTACACTGGGGTTTCCGCCTCGACACTGTACAAGTGTGTGTATAGACTATGTACCATACAGTACATTTTCACCCAGCATCTGATCCCCAGTTGATTAGATAAGTCGGCGGCCCGGATCTGAGTTCTTGTGGTGCCACTTGACGATCTACTACTTATCGCGATGGGCTGCTAGGGCCTTTTTTTCTATC

>DS499594:4670895-4671296

GGAAATTCCGGAGAGCGCGGTTCGGGGTCTGTCCGTGCAGTCGGGGGCACTGACCAATGGGAACGTGTAAGGGTTGCCTGGACCAATCAGCTCTCCGCGATAACAAGATCAGAGAACCTCCAATTCCAAGGCGACGCCAAAAAGCCCCACCGAGACCGCCTGCAGGATTGATTGGTTTCCACCCTTGGGTTTGGACGCCACAATGGACTGGCAAGGTAGCAGTGCCCAATCGAGAGAGTAGCAGCCCGTCGTTGCATAGAGGAAGTGGATGGATTCCTGGCTCATGACCACACCGCTAGGCAATGGTTTGGGAACCAGACCGATAAGGGCGGGTTATTCAAGGAGAAAGGAGGGATCTACCTTCAGCATCATCCTTTCGTCAAATGTTTGTCATGAGGCTA

>DS499595:25962-26363

TTGAGTGCCATCATGCCATATCTACTGAGTAAAGTTTTGCAGTCATATTTCTGGAACAGCGGGAGGATTCTAACCGCATCATTTGAATTTAGTGTTACATCGGCCAAACTCTAAAGAATTTAAGGCTTACTGTGTAGGTTCAAGCCAAGAGGCAGCAGTTGGGAAAAAAAGATCATGAGCCCTGGAAGGCGCGATTGGCAGATTGATAATCTTGACAAATGAAACGCGGAGCCCCCGAGTTAGTCACTGGTTTCCTCCAGATTCCAGAATCATTTCACACTCGATAGACGACGGATACCCGATATCGGTCCGGGTGGCCAATTTTCTGAACGGAAAAGATCCATCCAATGACCTTTTGGACGAATGGGACTTGCTGTCCAACTCCGAGGCCAAGCTTTGTG

>DS499595:165303-165704

TCAGAACTTGGCCTCTTAGACTCGGGATCTGGACCACTGACAGCGCCCAAACGTGGCCGTTGACGGGGCAAAAGTTGGAAGCGTCTTCTCCCGGCCCTTCCGGCCCACCCCGAAAGTCCCTTGCGTAGTGCGAAACACGATTGGCGCAGCTTTCCCTGTTTCTCTTGATCTAACGGCCAGGAAACAAAACAGGATCAATAAAATCTGCGCTTGCGATCTGTCCATTCATCTTCGGGGGTTGGTGCCGAGGCACCTCCGGACACAGTGGTGGTTATCTATTCGACGGAGGAATCTATAGCCTGAGTGGTGAGGCCCAGGCCCCCGAACTAAAACCCAATAGTCGTTCGAACAAACGTTCAATAGGGGCAAGTCCGGGGGTTGTGCGGCCCCGACTTTCCCAG

>DS499595:257783-258184

GGGTCCTTTTGTTCCCGGTTTGAATGCAGGATACGTAACTGTAGGAAAAAAACTACACTAGTGGAGGCCTAAGGCTAGGAGCGATTTAGTGACTCCAAAAAGTCGTTTGTTTCGGCTTCGCTTTCAGTCGAGCATCGAGCAACCAACCAATCAGGACGTTGCGCCTTGGTTCTATCGGTGGATCAACCATGGAACCCGAGGAGAACTCGGACGTTTACCACCAAAAAAAGACATCGGCAAAACAAAGGCTGATACCGGGCAGTAAGAATACTAGACTAGAAGGTGGCAATGCGACTCCGTACGGAGTACTCCGTAAAACTGCTTGCTATCCGACCAGAAACACTACAATTCATTCCTTGCGTGTACTCCATACGCCTGAATAAAAGAATACGTAACCTCCG

>DS499595:288265-288666

GATAGTTTAATAGATCCGGCTATTTATGGCAACTGACTGCGATGACGTAAGAAGATGAAACGGCTTCGACAGCGATCGGTCAAGGTCCTTCGAGAGAAGGGCAGGTAACGTTACAGCCTTATACAGCATTTGAGGTGTCAAACAAAACAAACCCAGCTGAATATTGCTCTCACTGCTGTAATACAAAAACGTACCTGTCGGTTTGCCGGAATCGCTTCTTCCCGCGATCATACGATGTGCTCCGCCGAGCCACGGCCGAGATTCCCATGTTGCCCCAAAGGCTCAGGCACAGGCAGCAATTCGTTTCCTTAGGCACATCTGCAGCTCAAGGAGACTGTTTCGATACATCTGCTTACTACTGCTCAACAGATTCGCCTTCGTTACTGTTGAGTTGCTCAGGT

>DS499595:332988-333389

CGTCCTCCTGGCAGGTCGTCATTGCTCAACCCTTTCATACCATCATTTTCCAAGCCACAAATTTTCTGGACTGCGTCATCGCATGGTTTTTGATTTTGACTGTCTTTCCTAGTTTCGGTTCGTACGGCAAACGACCTGAAAATAGCGTCCGTCGGATGCTTGCTAAATCAACAACCAATCAGCGATTGGGATAAGGAAGTTGGGCCCGCTTTGGTTCCCATTTGCCAGGATCATTTGACTCAGGACAGAAATCATGTCGCAGGTGAGTGAGATATGTCTTCTACTCTGCACTACTCTGCACGCGCTGTATCCTGCCTTGAAACACCCCTTACTACCTTTGTGACTCTCTATCTGCCTTGGGAGAAAGCATGCGTGTCCTTGTTCTTTTGCTTTTTTAGCTT

>DS499595:450246-450647

ACACAGCCACGTATAATTAATACAAAATGGAGCAGACAATTGAAGGGCTGCCGCTTTAGCCGACAGCACCAGAACTTGCCGACACCAGTCCGATGATGAGGTCATGTTGCATGAAGAAGTATTCCGTAACTAATGGTAGGACTCTTACTAGCAAAGCGCTGAGAAATTTAGTAAGGGACTAGTCCACAGGTAGATTACTCCGGAGACTAGCTGCAAGCAAATTGCAGAAAAGTTGCCGAACTTGCAAGGAGTTAGAGCTTCGGGGCCGCTGTCTGAGAGCTAGACTCGTAAAAGATTGCTGGCGCCAGTCAAGCCTCCAAAGCAAAGCAAAACAAAAAGTGTGGCTTGATCCACTAGCCACCTTGTACGGTAGACCATGGGAACCCGCTCCGCAGCCAAGA

>DS499595:507079-507480

TAGAATAGGGGATGATTCCTTGCACAGTGGACTTGGACGGGAACAAGCCCACAAGGCTGTCGAATCGCAGATTTACTGTGAGTGGACTGTGGAGTAGGAAACAGTTGTTCTTGGGCCACTTGGTCGACTAGATGTGACTGTACTCCGTAGTCTACTCCGGAGTAGAAGGGAGAACAGAGTGATGATTGAGATTGTTTCTCCCCGGAAAATGTGCCGACTGCCGAGTTAATTGAATTTGGTTCAGTGATTGTGATCTCATCGAAGCAGAGAATAACAGACTAGATGACAAGATCGTTGCCTGAGGGTCAGCAGAGGGACACATGACTCGACAATCCGTACTTTTGCTAGCGCTCTCTCTCTCTGTGTCCCCCTCGCCCTTCCAGGTACTTCCTCTTGGGCTG

>DS499595:574047-574448

ACGTCAGAGCCACTGGGCCGAGAAGAGGAGAATGGGGAGGAATGGAGACAGTTGCAGTTGAGAGTGAGTTGGACAACGTCATGGACGTGATATGCACTAGTCCCAGTTGAGGTTAGTACTAGGCATTAACGTAGCGGCTATCTGGAAGAGGTTAGCGGGATCCGGTTAATGATGACGTCGAGATCATCACTTAGTGTTCTTGGCTCTTCTGTACTGCGGAAAAAGCAAGAATCAGTGGATGCTATCTGCTGGTCGTGCCACACATCGAGCAATTAACTTTCAATGAACACGGAGACTCATGATGCATCCTCATTATTATATCACTACCCCAATAGTTTCTGTCCTTTCCCGCAGAGGTACCCGGGATCACCGGATCGTTGATATAATTGCAACGCCCAGGT

>DS499595:643563-643964

ATGCACATTATATCTTGGGATAGTATTTGTAATTACAATACAGATAACGTGGTATCACTTCCACAGGTACCTTGGTACTTACGTATCTAGACGAGGTCATCTGAGCAGCAACGCCCATACAAAGATATTGGAGAGCCCCGGGGTCGTCGACAGGGTACATAGAATGAGGAGTGAGATGCTGGAATTTTCCAGATTGTTATCTGCCTATAAGCTATTTGATCCAGGTATGAGTGTGGCTATGAGGTGAGATGATCAGATTATGCGAAGAACACAGCCTTTTGTACCTCTCAGCTGTTTCCTCTCTACTGTTTCGCCGCCCTATCTGCCCCAACCAGTCAGTTGCCACCCATTGATGGAACTCACTGTATCTGATGCAAGAGGAAATGGATGAAGAGTGGTGG

>DS499595:686129-686530

CAGAGTTCTAACTCTATCTAGTCGGAGACGCTGAACGAAGAGTCCGTCTCCCGTGGTGCAGCGTTTCAACTAGAATTAGACTAGGCTATGATTGTTCCCCTACTCTTTTTTTCCCTCCGCGACTAACCACAATCGACTGGGCGGGATCAGCGCAACGGAAGCCGTTAACCCCGAAATCCGAGCGAAATCACCGCAAGGGATTCTGGGATTTTCGAGGTTCCGTCCTTTCATCGCTGGAACCCGGTGGGTTGGCGAGAGGTGGCGGTGGTCATTTGCTTCCCCCCTTCCGAGTTTCTGATGGTCTCATGAGCCATGAATCCAGGATCTAGGACTTACGATCCATAGAGTATCAAAACGGATCCCGTAATTGATGCAATTGTGTGTGTGTGTGAAAGAGTCTA

>DS499595:696360-696761

GAGATCGCATCATTAGGCGTAATCAATCAGGTAATTTACTCAGTTACGGAGACTTCCTTAAAGCGCTGCCCCACTAGCACCACCCATTGCCCGTATCGGAATTGCCTCATCCTATTGATTGTATGAGATGCTGACCCCACTGTTCAGTCTCATGTATGGGCATACTGTAACTCCCGTAATTTTGGTCACTGACGCCGAACACTCTGTACTCTGGTTCGCTGGCGCTGCTCTCCCATCGACCGGTCTAGGGCTTTGGGAAAAAGGCAAATGATGTCATCTATGCCTTACAGTACACTGAGCACCTGATCCCCGTGTCCATCCCATCCCATCCCAGATAGGCCTGTTTCAATGTTACATGCCGGTATCATTACCCATGGCTTACCAGAAACCCACGTTGCTTG

>DS499595:763173-763574

TAGCGATTATGCCATTGATGAATTTAAGCCATCATTTCGTTCCGTCGTTAAAATTGCTGATGCCTAATTCACATCCATTCGCGCACTTGACTGGCATAAATGCCAGTCAAAGGCCAAGCCACAAATGACGGCACGGGGTCTCAGTCTACCTCCAGTTCTTCGGTATTCCCGTAGATCAGGCCATACTCCTTAATTCGTACTAGGCCATGTCATGCCATCCAGCCGTGGTTGCATCTCAGCACGTGTCGTCACATCCTTCCTCAATCTTGCATAGACTTGGCCTTTGGACGAAGTCCGTCATGGAAGACAATCTACAGTAAATCAGTGGAGCTAGGAAGAAGAGAAATCGACACTACACAAACACTAATTGCACTCCTTTCAATCAACTTCAACTATATTCT

>DS499595:768110-768511

TAAGACGGGAGGTGACCAGAGTATCAGAACTACTACTAGCGCAAGGATGCTAGCTAGCTTATGGCTGGTGTTTGGTGGGCCTGGCCCTGCCAGGCAGTTCCAGCCTAGCGTTCTGACTCGAGCATAAGAGAACGATCCTGGTCTATAACTCCTGTAGGTCTCAAAAAATGGTGTGGGACCTCGGGGTAATGTCATGGAATCAAGCTGCGAATGCCAGGCTCCGAATCCGTGATAGAGCAAGTGGCAATGCTGTCAAGCAGGAGACGTAGGATCTCACGCAGGCTAGTGTTTGTGACCTGAAAACGTACCCACTCAAAGTTCCCTTTGTTTTATTTTCGTCGACAGCTTCCCTGTTTCTCTTCGGATGGATTTTTCTTTTTCTTTCTTTTTTCCTTTTTCGC

>DS499595:769003-769404

AGTCCCTTGAGGCAATGGTGAAACGAATTTTCGCTCTCTCAAAAGAAACCGCTAATAGTTGGCCAAGAAATAAAAAAATTGAGGTTGCGCGATCCCCATGACTTAGAAACCGAGACCTACTGTGTACCCTGGAGCTACCGGAGCCAGCCGGAAGGAATGGCGTTAGTCTAAGCCTTCAAAAAGACTGAGCCAGAATGGAGCAGCAGGAAGCCGGGAGGTTGGGAGATGGCTGAGTTGCTTCATCACTATTCTGCTGGGTAGAAAGACAGATAGACAGGAAGAAGTGGTTGTCAAATGTCGACGTAGCGTGGGTCCTGTTGGTGGGATGATCTGAAGCCATCGTCAAACAATCAAATCATCTCCCAATTAAAGATGATTCCAGCCCAGCTCCGTCTTGAGTG

>DS499595:810276-810677

GCATTCGGTCAAGTCAATTCCCCGACGATGGAATAATCTCGATCTCCGATCGAAAATGATAATAGTTGTGATCTCACTTCTTCAAGGAAGAGAAGAATGACCTCAACCATGCAAATCATCTGAGGGCGAAATTTCGAGAACAGTCAATCCAGTCGGTCCATTCACCAGCCACAACTACCGAATCAGAATGGTAAGGTATTATTGCCCATTGATAAGCCGGAGTCAAGATCCTTGTATTTACAATACAGAGTACTTACAGGGCCCAGTGGCAGGACCCACAGCCCCCAAAGTCAATTTTGCGGCTTCTGCCTGGTCAACAGTCCAAGCACAAGTCATTACTCCGGACTATGGGGTTTTTAGATTTCATAGCGCTACCCTTCATTTCCAGGAGCGCGCGATGC

>DS499595:851923-852324

CATCCCCTGTCTTCAAATATAGGCAAGTACGTAATACCCATTTATCAAACGAATCCGACCTCCATGACCATATCATCTGTTGATCAAGAGCAAAATTGACAACTGGCAAGAATTATCTCCATAGGATCGTCCAATATCCATGAACCCATCAATCCATAGTCGCACCTTCAGCAGGTCACAAGCCACCTTCATGGACGCGGATAACGCATGCGACCCTGGCGCTGATAGCCTGATGCGGGGGGTCGAGGAACATGCTCAACTGGTCCGTTGGGTTTAAACACTCGGCCGGAGGACAAGGACAGGCGCATACCAGGGCTGCTGATTCGATAGGTTAGTCACGTTACCATTTTACATTTAATATTTCCATAGTAGTACGATGTTGTTTTCCGAGCTACCTTATG

>DS499595:1011278-1011679

GTCAGTTGACCAACCACTGGCAAAGTTCAGCCCATCCAGGTACCATGATCCACCTCCCAAATCAAGGCAAGACACCACTAAACAAGGCACGGGCTATCACCTGCTTCGGAGATTCGACGGGAGCTGTTTCCGAACGACGCTGACTTGACTCTGACCACTAAGAGCAGATACCTCGGGCACTTGGGTCAGTTCGGTCTCAGCTGGTACACGAGGACGTGACGATTAACCGGGGTTAATGATTCCTCAATGCGTCAGTTTGTCATTTGTCATGACTACGGGAAAATATGTCGCACATATGCAGCTAACATGCAATATGCTGAGTTGCAGGAAATCTAGGAACACTTGTAGACTTTGAAGATTGGCGGGGTGGGGACACGGTGCCTGTCCAGTTGTGTACTTGG

>DS499595:1014795-1015196

GCCTTCAACAGTTCCGGTTGGTCTTGATTTGACCATGTCTGGCATGATCGTTGAGAAGATCAGTGTCACCTCTAATCCTTGCAAGCATGATCAGGGGTACTACCCGTTCTATTGAAGTAAATCTGGAGATTCGACAGGTCAACTGTCTTAAACTTTAGCATCCCAGGGAGAGATGTACTCCGTATCCCGTACCCCGTAAAGATACGACAATAATACACAATACCATGGAGACCGTCATGTTACACATCCGCTGTATGCATGAGACAACTTGTTTCAGCTTTCAGGGGGCCGAAGCACACCATCGACCAACAAGATCTGTTTCCCCTTGCAGATGATCAGTTTGGGCTCAAGAATCCATCTAGACGAGTGATACTCCTTTGATATTACAACTCCAGGTTAAC

>DS499595:1020238-1020639

GCATATCCCATGTGGAACGTCATTGGGCCAAGCTGGACTCCGTGCGGCTATAAGGCCATCACGTGATACAGGGCAGGGCCTGAGGCTGCCAATCAGAAGCAGTGGAAATGAGGGGTGGCGTACTCCCAAGGTCCTCACGGGCGGGCCGAAAAGTCTTCGTCGTTGACCTCTGTTCACTGGTTCAGCTCCCTGGTTCCCTAAAAGGTTTGCGGAACCGCTTTCTCCCAAGGTCCCCATCAAAAGACCATTCCCACTAATGAGACAGATGATTCAGAGTGATGTGGGAGAAATACGGAGTACTGTCACCATGGTCCTCTTTCCAACCATCAATCCTCACGTCTGTACTCTATACACTTCCTCGACGAACCTATCCCGTCTCACAGGGATCCTATCTTGTTTTC

>DS499595:1083919-1084320

ATTCTTCATTTCGAGGATAGCAGAGGGGAAAAAGTCTACTACATCATACCATACATAGTAGGACGATTCCATGTGCTTCCCAGCAAGCAGAAAACAGGGACATCGCAGTTTGATGCAGTGGGGTGTTCCAGACTCGCGACTCTTGTTATCGAACTGTCTCGAAAGATCCCTAACCATAGTGGGGGAACTGCCAACGAGATATGAGACTTCTTCCATACATTATTAGTCCCCCAATCCCCCTAGGCGCTCTAGATTGCCTAAGTACGGATCTGCCCGGCACTCAAGACAGGAAATACGCTGCCATTGGACCATTCCCTGCTCGAACCGGTTTTGTCAGAGAGTTACCTTACCTCAGGCCGTTGAATGTATGGCATATCACATCTGACAGGAAAGTATGCAGG

>DS499595:1103833-1104234

ACAAGCAAGTCCACCCCGTCAGATCTGAAGCAATGGCCTCATGCTAAACCAGTTATTCGTCAGTCAGTAATATCATCGACGTACGGAGTACGGAGTAAAGGTCAGCCGGTCAGACTTCAGCCGCTGATAACTTACCCAAGAGGGAAACGGCGGGCGGGAAGACGGCAGAAAATCAAGCCACAGTCCGAGGTTTTCAATGATATTGTTATCATTTGTTGAGTCGGAGTTGAAACTTGAGAGCGAGCTCGTAGCCTAAGTGGATAAGTTCGTGTACTCTGGACGTGTTTGGCTTTGGTGATCACTAATGGAAACTGGGCCAGCTCAATTCTTACCTTAGCGTCCTTGGTGTGACAAGCATCCTGGCAAGCCACGTCCGCCATTGGTGCGTTTCTCTCCCACCG

>DS499595:1137372-1137773

GTAACCTCAGCGACAATACCCTTTTCGGGTTCCTTAGGACCATTTCAAATTGACCATGAATTCGGAACGATAGAAACACGGAGCACCTTCAAAGCCTCTAGTTCTGCCTTCCGTGGCCAACGAAAAATACGAAGTTAAAGGGAGGAAGAAGTATGGTAAATGCCGCGGGTTAAGCTTCCATCATGTGACAACGAGTTGTTCTTGTGACCTCTTTGTCGCCACTGAGACGACAGAGGCTGAGCTTTTGCCCCCACATCAAACAGTACTCTGTACAGAGTACAGAATACAGCAGCATCACTGTGGTGCTTCGTACTCCTTATCGATGCTGAAGACAAGTTCTCAGCTTCGTTGTTTTAACCTATTCTAGTTGGATTGAAGTTTCCTCCTGCTGAGTGAATCTT

>DS499595:1138940-1139341

TTGGCTCTATTTCGGCCCGAGCCTACCACTCTCTGTTCAGAGAAGGACTTCTCCCTCTCCTCTTCATTTCCCGCCTGCGCCAGGAGATGAGAGTATTGAGCTGGTCGGCGAGCATGGCGTTGCAGAACATCATGTGGCTGACAGTGTGTGTCCATACAAGAGGATGAGGTGGGGGAGAGGCGAAATGTACTAAATCAAACAACTCAGCCATGAGATGCTTCTGGGACCTTGGCTGCGTTGTAGCACATCACATGATCGTCAAAACGTGACCTGTACTCCATACTACTAGTCCTACTACTACACTATTTAGTTGATAGTAATTCTAGGTGGCTGTCATGATCACCGTAACCAAGGACTACTTCTCTGGGGGAAATTGCCACTGGTATTACCAGCTGTTTTGT

>DS499595:1140843-1141244

CACCGGGACCAATCCCATTACTAAGGTATTCTGTATGTACGACCTTGAGTATTGTGGCTTTACCTACTCCGTACTTTACTCCAACACAAAAAGCTCTTTGGATTAGTACGTAAGAATTTTGGGCCAAAAAGTTCCGTACTTTCTTCTTGGGGGTGGAACTCCGGGAACAGACCGGTAAAATTACTTTTGGTAATGACATTGCACGAAATACCAGGTGACCCACGGGAATACTCCGGAAGTGATACACAGCATTCGGGCGAAAAAAATGAAATAAGTAAATTGATTATTAAAAAAAAAATCGGCCCCAAAAAAAGAAAAAAAGAAAAAAGAAAAAAAAAAACTAACAGGAGGTAACCTTTCCATTATTATTGTATTGTACTTACCGCCCATCCCATCAGTCG

>DS499595:1144666-1145067

TTACGTGTCAGCGATAATTAACAGGTCATTCTTCGCAGGGATGCATCTGATTGTTTAACAACCATCAGCACTAATGCATGGGAATATATTAACCTTTTCAGCCATGGATCGGTTAATGTCTTGATAAGCGCAACCGACTGCCGGAAAGGCGGATTCCCTCTGCATGGCCCTGCTCGGGCGGTGATCTAGAATCGTCGCATTGATCCCTTCACATGAAAATTGGCTCACAAAGACTGCCTGGCCAAGGCACAAGCTAGAGTCCAGAATCTTGATACTGCACTTTCTTTTCCCATTATTAATTCCATTTGATGATTTGAGCTTTCCTTATCTCTTTCGACTGCCGATGTAGAGGATCTGGAATTGGTAGACAGGTACTAATTAGTATGGGGTATGTGCGGAGT

>DS499595:1146158-1146559

CACCCGCGTACGGAATAGACGACCATTTGCTTTTGGACAATGTGGACGGTGGGCTACTCAGGCCTCAGCTCCTAGTTTTCCCGATCTCTGCGGGGTGTACCAATATAAAGGGCCAGAGGGCTGAGAGAATTTGCAGGCTGATATCTGCCAGGGCACGCAGACGCGGATCCCATAACGCGACCTCTGTGGCTGGTGACATGGACGCGTGTGGACCTACTCTGTACGGAGTAAGATGTTGCTGATCCTGAGGTCTTGTCAGGTCCAGGTCCTGGCCTAATCTTCGAGTTTCTACTCCAGCGGTGGCTGTCACTGTAACTGGACAGGAATGCTCCTATAGGCAGACAAAATCAGCAGCCTTCCAGAAGAAGGTTAGAGCAGGAGAGGAGCTCTTCTTGTGTTGC

>DS499595:1148596-1148997

AACTCAGTCCCTCCGTTCCTCCAACAAGTCTTACCGTAAAACGAGCTCAGGTGGTAATCATGTCTTCATCCAAATATTGACCTCATTATTATCCTTGCAACGTGGAATTGAGATCTATCCTCAACAATCTTAGATTAAGAGGCCTGTAGTTGTTCTGCAAAAAATTTTCAGGTCCGGTGACTTGAAAGGTAACTGAGACGGGTTATACTTGGTAGGTCCTTACCGGATACGGGAGAGCTCCTGATTATTTTCGTCTAGTGGACCCAGGTTGCATTCCGATGTTTTCATCTCAGCTTGGAGCGTAGTTTGGAGTCGCGCGGGGAGGGGTCTAGAAGGCTTCTGATTTGGCGGGGAAGTTGGGACAATCCCTTAGTGAACTTTTGACGCGATCCTGGACGTTA

>DS499595:1150386-1150787

ACGAGGTATATAATTGCCATTTCTATTAGCAATATTGTGCTCTTGATAAGAATACGAGTCCTCACTCATCGCCTGATATTTACTTTCCCGGCTTCGACTCTTCTCTCACGTAACAGCTGCAGCATTCTGATATTCTGGATCAGTTGTCCTACGGTGCATCTATTAGCTACCGTCGATCTTGCGGTCACCGGATCTCTGCGTTGGCAGGTTGATCACACTTCACAGTACTTCGCCCCTGTCCTGGTGTCCTAAGATTGAGTGACTGACAAGTCATCATAGTCGTTTGCGGAAAGGTATCGTATTAGGTGTGTCTCTTTCCGCGTGATCTATGGGGCATTTTGCTACATTATCACTTCTACTAGCTAGAAATGGTTGTCAGACGCCTTTTTTAGACAGTAGAA

>DS499595:1377077-1377478

TCAAGTTAGTCATGCTCTTCAGCTCTTGTAGGTCGGCATAGCGAGAATACTTCGGCAAACGGCATTGTTCAAACTCATAGACGGCATTTTGCCGTTCGGTTAGTAGACGTCCACCGCAACCTTCACCAATACATTTGCAGCAGCGTCTTAAGTGACGCAATGGGTCACCGAGTATATCGAGCAGCAACTAAACAAACTCGGAAAACCGAACACCGAACGCCGTTCAAATTTGCATCCCGATTTTTCTTCGGGCAGTAACTCACTGTAGATGCAGAAAATAGGGAAGACTGAGTCATTCGCCGTGTGGCAATCCCGGCTTCCCCCGAGGTCACATGGGCAGACTCCGGTATCTTACTAAGATACGATATCGTATCCAGATGACAGATACGAGATTATGCCAG

>DS499595:1556135-1556536

CGCTTTTCGCTACCAGGGCTACGACGTGCTCTTAATCAATGGATACAATCTCTTCCGGAGGGAGTTGTCTAGACCCCTCGACCGCTTCGTGTGGGGAGTGGGATTCTGCCCTTTGCAGTCAACCGTCAACAATCGTGTCGCCGAATATGGATGCCCGATACACTACCAGAGTGTCTGTGCCAGTGAGTATGCCAGGGATGGGCTGATCTGGCGTTGACCTGGCAGTCCCATGAACTCCCACATCATCTGCGTGAGGGGCATGATTGGATTGGATTCTTATCTTGATTCCCAGATCCCGATAATCGTGGCTTTGTCGGTTAACAGAAAATTGGAAGGGGAGAAAAGGCAAATAGTGTCAAGGTTCGTAGCTTTATCTGGTGATGTAGACTAGATTATCCGGA

>DS499595:1556935-1557336

TTGCGCGTTCCAATTGAAACCTCAGGCACCAAGCATCCGTTCTAGACCTCCTTTTGATGATGATGGCGTGACTCACCTTCTGTCTGTTGACAAAGGACAAGACATCAGCCATCGATTCCATCATCAATTGTTATTATTATTGGATGCTAAGGAAAATACGGAGTACTGAAGACGGATTGTGTGGAAATCTCTTCTGGAAAGGACCAACAGGCTAACGCATTGTGTACATCATGCTTTCTCCATGTAGCGGGACTGCTGGACAGAAGTTCTAGAATTTCCCTCGGCGCTGAGACCGGCTCCTAATTATACCTTGCTCCTTTGTACTCGGATACCACCTTTTCTGGGTCTGCGGACGACCTATCTACAGGGTACTCTCAATTCATCAGCATACATAATCACTG

>DS499595:1578846-1579247

TCAGAGCATCCCCAACAGCAGATGAGTCGCCTGGTTGGACTCTTAAATGGAGGTACTGGCCGCAAGTGTCGGGTTAGTCGTTCCTAACCCATGCAGCGTACCGAGTCCTTGGGACACACCGGCCTGTAAGAGACCCCGACTGCGCCCCTGTAGTGTCCGGCCGTGGAGAGGGTGGTTTCTCCGGCGGGTACGTGATGGTGCTGGATGGACGGTCCTTGTCGCAGTGATGCGTAGCGCCAGAACAGGGGGCAAAGTTCAAAATTAAACAGGTGCAGGCAGAAGTGTTGGTAATGGTGGTGGTGTGATGGTGTTGTAACGAGGGAAAAGAGACACTTGAAATAAAATTGGAACCTTGGAATGTGCCAGACACAGACACAGAGAATTGAACAATGGGTATAATA

>DS499595:1581628-1582029

GATGGGGGGGTACAGAGTGTAGGAGGGAAGAAAACCAACAGAAACGGCCGAAATGAACTGTGTTTATTGGATGCGGAAGCGTGAAGTCAACAGTCCAACCTGCAGTGGCTTTGCAGGTGAACCGTGGTACTGGGACCAATAGACCAATCAGATGCAGCCTTCTATGCCGCCTAGCTCTTTCGGTCTCAGATTACACAGACCGATTGCTGAAGGACAGGCGATAATGAGAGCTAGGTTCTTACCCGTCACAGGTAAGTACGTACCGGCCCCAGGCTTACCTACTTGGCTTCAAGTTCGGGGGTTCGTAAAAAATTGACACTTGACAGCAGCATGCGATATGAGTGGGTCTTTTAAGTTGAGGATTTACTCAGAGTAGATAATACGACGAGGGAGAAAGGGAA

>DS499595:1583457-1583858

TACGTCCGTCACTTGTAGTGCAATCATGATGATACACATCATACTAAAGAGGCATAGATAAGCAGCATGACTCATGACCTCCTTTACTTCATGATTCTGCATTAGTAATACCCTGATGGTAAGGTACCATTTTTCCTTAATGAGTGGCTGGCTTTTTGGAAATTGCGGCACAGTCAACTTGCATAAGCTAAGAACAAATAAACATTGCTAGTAAAAAACAGTCACATAACTCGCCACTTTAACATGCAAATCAACCAGTTGAATATCCATCCTCACTAATCACACTCAAGCGGCCAAAGACAACTCATGTCCAGCCTAGTATACCAGCCTCCCATGGTGAATATAGGCGCCTTGACCACATGATGTCTCACAGCACAGCACAGCAAACGTTTCTTTGCTCG

>DS499595:1584881-1585282

TCGCCAGTTTGGACCGCTCAGTGGCTGACCAACTTCAACTAGGAGCTCAAGAGAAAGGGCATGAAGGCAGAGGTGATCCACCCTGAGGCTCAAGGCTGGAAGAACGGTGTCGGAGCTATGCTAGGGTCCGCTTCGAGGCCCAATGCTGCCAGCCTGGCACAAGACAGGTTCATTAATCCTAATGTCCCTGGTATTAGTGCCATACGCCAGCACCGCTAGTCTCTGTTCGCCTCTTGAGGAGGAAATACCTACGACGGAGTACAAAGTACATTACGGTACTTGTGTTGTCTACCAACCTAGGTACATTATTGAAGAATGTGATTGGTACAAAGGAGAGGAACTGACTGCCTAATACTGCATCTGAAGGAGAATAGAGTGATTTGGAGATTCCAGGAACACCG

>DS499595:1588716-1589117

GATGTTACTGCTTTCGTCACTTCGTGCTCATAAATACTGACCGTCCTGCCCGCTCACTCGCAACGTTTCGTCCATGAAAGCTGCTAACGCTCAACTATCACACCCTCATCCAGACTGTCGGGTCTGTAGAGTGGTATCGCCCCATGCAGGCATCAGACGGTGGCCCCCCGACTTGCTTGGACGCAATCAATTATTAATCTAATTTTGATGAGCCCGGTGCCTCTTTTGGGTTTGCGGGTCGCCTAGCCTCTGGACCTTTCATACCTGGAGCCCGCGAGCCTTGAGCCCTGAACCGTTCGCTCACAAGTCGCGCATTATGGAAATGTCGGATGCGAGTCACTTCCCACATCGAGCCATATTGCGTATGTCGGTTGTGACCTCGTGAATGGTTCAACGTGCTA

>DS499595:1598331-1598732

GAGGTGCCTTTACTGTAATTAGCCCAAGAGGAGGACAAATAATAATATTCGCTGTGTTTTGTAGGGGTATTGTAGATTGATTATTAGCATGAGGGATATTCCGTCGGACCACCTGCAGCGGGTGATCTTATGATCTTATCATATCTGATGGGATCCTTGATTGCCACCTGATTAAGGAATGTGACTCGACTGCTCTCCCGCATTCCCGCAAGTACCCAAGTCCAACTCGACTTTCACCCCCACTTGTCATCCTCACACGGCCCTTGTCTGACTTGACAATCCGAACTTCGTCTTGTTCAGTTCTTGTCATGGTTTTTTGACGATATTCGAACGATCCACCACGATTCCGGATTTCGATGATTCCTCTCTTCTCCGATTGCCGCACAACCTGATGGCCGAGG

>DS499595:1621272-1621673

TCAGAACCGCCATTACTGGTAACTGGTTTGCTTGCATTCCACCTGCCCAGCATACAGTAGGGTATACATTCCTCGGCGCCAACACCGCTCTGACGTCGACGATTATTGGCACCGCTGCACGGTCCAGTCAATGTATTTCAAACGGCCTGTAGACTCCATTTGCATCGAGACAATGGTGGATCCATTGAACCACACAATTTAACAAGGGGGTTTCCGTGCGCATCCGCTGTAGCTCGTAGCTGTGCCATCAGCCATACCCTTAAGCCTATAGGGCTGATACTGGTTTGATTATGCACGTGAACATGAGTCTCATGACTCTTCGCCAGGCAACAGTTTATCTATGTACAATCAATCGTCTAGAACTCTAGCTACACATCTATCAGAAAGATCAGAAGGTTACC

>DS499595:1627095-1627496

CTGATGATACATTAAAACTCGAGATTCATAATGATAAGTACTTGATAGACATGTTATCTATCCTTCTTCTCCCTGCATAACAACGAGATAAATTACACCAACTACATCATGCACCATCAGCTAACACCAACCTCAACAACCAGAACGACAAAGCGAACAATACACCAATCCATTCATTTCTATGAGTCATTTTACGCACAGCAAAGCCCTCGAAACTCTCCAAAATTACACCAGGGTAATTCCGGAATGCCACCAAGTTCCCCGTGATCGCCCGACCCTCAACAGCCTGCCCAGTTTCGGGAAATGCCTCGGTAAAGTGGCTTTCCTGCATGGCCCTGTTGGATGGGTTTCCTTCTTCCTGCATCATACGGCAGTTATTGCTCTGGATTATGGTGGCTTAA

>DS499595:1639462-1639863

TAGAGAGCGGCCAAATCGGTGCCCAATATAAACAGATTAGAGACCCAAGGCACGCCTCCGGCTGATAGGCTGATATATGAATGTGAGTGACGATCCCGAACCGAACAGCGCCGCAGCCGGAGGAACTCCAGAACTTCAGATAAGGGCATTCTGGGCACGAAGCTTGGGTTCCTGAAATGCCCGAACGAGACTTCGGGAGAATAAGGGAAATCCAGCAGGAACCACCCAGGGCTGATTTGGGCTGAAATGTTATTGGCCAAGCTTCTCGTTATCAGTGCTGTGGGGCTTCTTCGGCTGCAGCCATACCACATCCGGAGCTAACGGGCTCCACCCACTCGGCAGGTGGCGTCGAGGACCTTATCGGGACCTCACAGCCAATCGATTTGCTTATCAGGGTGAGC

>DS499595:1669414-1669815

CAAAAAGTGACGGATTCATGTAGACAGTGGATAGTGAGCGTGCAAATCAGCTCCTTGCTTCCGCGCAGAGCCGGATGGACCATGTGTGCCAATGATGATGCAGGCGGACAGGGGAGAACAAGAAGAGCAAATCGGATCGGCAAACAGGCAAATATGAACCGAACAGGGAAACATGAACATGAAGGTGATCGAATCAACAGATGGATCTGAACGGAGGTGCATGGGGTTGTCTCTGAACGGAAGACGACGGATGTGGACAACACCAGTGGTGATCTTCCGTAGGGCAGAGTATTATTAATTGTTTGATAGCTTGGTATTGCAGATGGATCTTAATGGAAACTCGGGAACAAATTGATGACAAAAGATGATGATGATAAATACAAGCAGCTTACTATTCGTGA

>DS499595:1717159-1717560

TCGAGGCTCTGACACTCTGCCCCTGAGTGTTTCATAGTGTGTTGTACAGTCACTGTTCATTGGTTTATTGTCTTCTTGTGAGGTGGGTCGTTATTCCTGAATGACGCCACTTGGAGCTATTCTTCTTAGTCTAGACTCTAGTCTAGGCAGGCACAGAGCTGGATAGTTACAGGTATCTGTCTGCTATTTTAATCCCATGCTCAGTACGGGCTAATCCATACTAACGGTACTGGACCAAGGGACGGGCTTCCCCTCTGCTAAGGTTAAATAGGTAACCATGGATTGTCATCATTAGATAACTTACGAAGTATGTAACCTAGCTGCTGTCAATCGTTTGTTGATATGATGGTCCTAGGTACTGGGCTTTTTAACTGTGCTTAGGAGGCACGTACCGTCTGACT

>DS499595:1942506-1942907

TGTACAGTACAGGTACGAATGTTAGACCCAGCTGTGGGGTCGACGGATCATTACCCGATGCTTAAGCATGGTCAGGCAACTAATTGTCTGTCAGGGGAATGAGCACACTCGGAACGATCACTTCCTTGTCTCGCTGACTTATTCTGCAGAGATCGACTGCGGTGTGCGGAAATATCCCTGAAAAAGAGAGTCAAGGACCCCCTCCCAATAAACCAATCATATGCGCTCAGGTGGGTGAGAACGTGGCATGAGTAAATCACGATCATCGTGACCATCCCGGCTGGGTAGGAATCGCCGAGTGTAAACGTAGCGTAAGCGCTGATAGCGGACTTGAGCAAGTGGTCTGATTGTCTTTGGTCGCGGCTCGAAGAATATGCCGCACAAAAGAGGACGAGCCACAA

>DS499595:2004392-2004793 stuA

CCAGCCAGGATGAGCGAGCTGACAGGGATCGGTACAGATGGCAGGGATAGAACTACTGGTCGAGAAAGCACCACGAAAGCTGGCGGTGGCGCGCGAGGAATAGTCGATCCAAGTCCATAATTCCATGCTATCATAGGAGTACTCCGGAGTAGGTATCTGTCAACCTCGCTGAAGAGAGGCCCGTGTCAAGCTTGGCTGATTGACCATCATCAATGGCCTACATACAGTGCACTGTTTAGGCAAGGACTGAGCAAGTGACCAGGAACCACGTAGATTGTTCCAAAGTAAATCTCGAGTCTCAACATAATAGGATCCGATCAAAAATCAAATCTCCAACAATAGTCGAGTGAGAATCGACGCATGAATCAACTAGTTCCCTCGTCGGAAGATTCTACACATAC

>DS499595:2005343-2005744

CAATAACTAAGCGAAGCATCAGTCAAGGCCTCACCGCCTCTTAAGAGATACTGCTGGTCGACGTCAAGCAAAGTCAGTTGGATAGGTCCTCTAATTCCAACTGTCTCATACTCGGCTGACAGGAGGAAAAGGAAGATTGATAGGCCAAGACGACTCTTCTTGGAGCAACTCTCAACCCCTTGCCTGAGGTGGGGCAATGAACAAACTAGTACTGAAATGGCTAGGTCAATACGGCCTACATACGGATACCGGCTTCTGGGTTAAGGCAGTCGCGACAGTGATCGTGACTGATCAGGTCAGACTCTGAGACTCTGACCCCACCACAGTTCTGCGATGCATTGAATCAACCCCGCTCTTCTAATCAGCTTCGTCCTTACTCATCCGTCCAGCCGAACTGTCCG

>DS499595:2006666-2007067

GAGTTCCCTCATCCATCAGGTAACTAGTTACGTACGCAGCAGTTCCATTGTCTTGAGTCAGGGCTTAGGAGCTCGGGCCTGTCCAATCCTAACCCCGCTCACTAATTGCATTAGCCAAACTAGCTGAATGCTTTAGGTTCCGCAGCGCAACCATGGATAAAAGCCAGCCTCAACCGCCTGGATCTTCAGACGGCCAGGGCTCTCGTCATAAGTCTAGTAGTGGCTCTAGTCCGGCGGCCAGCTGTCAAGGATCAATGTCATCCAGTCATCCGCCACTGCGCTAGGACGGAATCCGCAAATGCAGTGACAAACAGGCAGGGCCAATGCGGATTTCGGCGCTCAGGATCCTGGTCGCTTGCTGTGATGTTTGCAACCCTGCAAATTGCAATTTAATTAGTGGC

>DS499595:2007796-2008197

CTGCCCTCAGATCCATCCTTTACGCGCAGTCTTTGTATGGAATAGTCAGGCATACACGAATAGACCTTCTCCACGTTACACCTGACTATCGTTTACGACCGGTGTGACATGGTAGGGATTGGATGTGAGGAAGGCACCCTGGTAAGCGTCCTGCTTGCAGTTTCGGGATTTCTCGCGCCAATCACAGCAGCCCAGCAGACTCTAGTCGAGCGCGCAAGGCAAAACTCATAGAAAGTTGCCTTGTCTCCGTACGGATACAGTCAGCATCTTGAAACTGGTCCTGTGCAACTTTACGGAGTATATGCTGTGCTACGCTGTATCTTCTAGAATCCAGCCTGGATGGAATGGAAAGGAGTTGAATTGCCACTCGCCTATCTGCCTCATGGCACGTTCCGAGGTGA

>DS499595:2009160-2009561

AACAACAACAAATCTTGGCCAGGCTCCATGCTCTGAGGTCGGTCTCTATTCGACAAGAAATAGAGGGGCAGGGTGGTGAAAAATGGACGAATGCGATTGGTGGGAGCGAGATCTCATCCCTGGGCTGCACATGTTGAGGGGCGTCTAGAGGCGGATCGACTGGCTGCGGGAGCGACCACGTAGTCGACGAGGGGACACGGTCACCCCATGATGCAGCGAACCGTGAGCCTCCAGTGGTTACTGTTATCTAGGCCATCTATTAGACAGAAGGTCTAATAATAATAAATACGACAATGATAAAGATAATGACAAGACAACGAAGACTAGTTGAATCAAGGAAAAGAAGAACGAATTGAACCTACCACCGTCGCAAACTGGTCATGGTCACCACGTAACAACAA

>DS499595:2079031-2079432

TCTAATCGACACCGCCATCGCCTAAACAAGCACAGAGCTTGTTCTAGACGCTCCATCCAACCTGTCTTCTGATCAAGTTATGTGCCTACGACTTGCTCCGTACAATCATTCGTCTACCTGAACGGCTGGATTTATCGCATGCAGTAATTATTTCGACCTCTCTGCGTTCACTCCCCATTCATCTCGGTACATTCTCCGCCAGCATGTGGTCTTCGTCTATTCGTCTTTGGGTTTCGTCTACTGTGTAACGTGTCTTGTTTCCTCCTGTACGACAGGCGATCACCATTATTCGACCCCATTCAACTGTTCCGACTGTCCGGACCCCAGCTACGTCTATCCGCTTGGTCAACATTAGTTGGATTTTGGTTCGGTCATCCCACACCTTTGTTTTTTCCCCCTTT

>DS499595:2206064-2206465

ACTCAGCGGTCTGCGACTAGCTTACATAGTGCTCGCATTCTCGGTTCTACTGCCGTCCTTGTTTGCTCTAGTCATGGAGCTTTACGTGATTGTTCCTGTGCATACGTACCTGGGTGGAACACGGGCGCACATCATCCACTTTGTGCAAGACTGGACGCTTGGGGTGCTGTATGTTCAAATGGCTATCAAGTTTGCTCTATGGCATTCTACTTCGCGACCTGCAGCGGCCCTCAACGGTGTTTTCCGGAACGGCTGGCTGAAACCGAATGTCCGTCTTGCCACCCGAGCAATAGTGCTTCCTGTCACAATCCTGGCGACACTTGCGGTGGCTCTCCCACTTTCTTTCGGGTTCGCACTGAACTCGACTGTCTTCCGTGACAACCCAGATATCCAAGCCAAGG

>DS499595:2346978-2347379

TGTCATCAATTCGTATTGGACTCGTCACTAGCTATTTCATCGCTGCAAGTCTGTGGCAAGAGTGTATTAGCGATGTGCCGATGCTCTCGGAAGGTCGAAAGTTGTCGGTGCTGCAATGCGAGAGGAAACAATTTTGTACACCTGAGTTTATTACCGAACAGATACGGGGCACATTTTGTGGTCTGTAATGCAGGTGGGGGTATATTCTACCATCAGGTATATACATGTCTTAGGGCCAAAGATCCATCAGACAACATGATCATAATGCTCCCGTCAACGCGTCCCCGACCCAATCCATCTGCACCGCGCTCACTCCTCGTGCATAACTGCGATTCGTAAACACAAACTCATTATACATGATAGCCTCCACTTTCTTGCCGAATAAGACGCTGGACGGGTGG

>DS499595:2373780-2374181

AGAAATGGAGTAGTCACGTTAGTGTGACTCTGTGCCGTGCTGGGGATCTCCTGGGTCGGGATGGCTGATCAGGTCATCTTTGCATTCGGATGAACTCGAACCTTACATCCGAACCATCACAGCGGGTCGTGTCGGCTTCTGATAGATGGAGGATCGTGCCATCAAGCACTCGGACGCGATCCCGAATTTGACCGTATGCAGCGATTACTCGTGGCCATTCGTTTAAATGGGATCAACTGACTCAGGTGGTCAGCGAGATAGGGGCTGCAGTCCATTGAGCAAACAGAACTTAATACGAATGTCCTCAGTTGAGTTCCGATCATGGAGCCGGAAATTCCCTGTACACGAGGCCAGCGACGGAAAGCTGTTCACGTCACATGTGATGCTGTACAATGTGTCCT

>DS499595:2413983-2414384

CTCTTGTTGATTCAAGACACAACCCCCATCCTGTCCGGCCTGCATAACTCCAATGGCAGCCCCTCTTCAGCCTTCAACTGCAGGACGGCTACTCGGCACTTAACTCATCGACATCAAGGCAGCTTTCCCCTCGCAAATGAGGGGTCAAATGAAGCTGTCAGCTGCCTCTACATGACTCACGGAAACTAGACTTGAGTCCGACTGTACGTAGTAGACTACACCAAATAGAGGAAATACTAGCAAACTTGCGGAATTCCAGTGTTTCAAGGGGAAAATTCGGGATTATCGGAAATAAATTCATCTTTGAATTCAGAAGTGATACCCAAGAGAAAATTTCCCGGAGTCCTAACTCCGTTGATGGACTAGTCTAGAGTTGAGTTGACGTGAAGAGTCATATCCGT

>DS499595:2416695-2417096

CTTAGTGATGTTTTTCTACCTCTGGTTGCCGTGATCCCGTCTAAGTCCGAACGGCGATCGGACGCCGGAGAGAAAGCGAGCCTGACATCCATGGCTCGGGCACAACGGGATAACTGGGTCCACCTGAGAAAACCTGAAAAAGAAAAAGCGCCTGATTCAGCCCTGGCGATCAACCGATCAATGGCGATCCAAGGAACAACCATGAAAAATTAGTCAGGCAGGTAGTCGCGAACGCTGCAGGCTCACCGCTTCCTTCTGACAGCCAATGAGTTACAATGACTCACTTCGTTAAAATCGCCAAGCTTAACTCCTTTAGGAAATTCCTGACCAGAAGTGCCGATCCAGCCCTGCGGATGGGGCTCGGATGACCAATCTTTCCGCCCTTATGAACACTGTGATTG

>DS499595:2418886-2419287

TCTTCTATAGCATCCGACTACAATAAGGTAACGATACTGCCAAATGCTTGACATTTGACATTACACTCCATCAACAGCCTTGCTCAATCACATGACGGCAATCACTACGACTATCAGAACCAATCACCACTGATTTTTCTGCCAAACATTTTATATCAAGGCAGATAAAAATCCCCACATCCCTTATCACCCTTAGTAACTCCGGAGTATACGGCATACTCCGTACGGATACGATAATTATGCTTATGCTTATACATTATGCACATATGCATAACCAAAAACGCATGTGCCACTTCATCTGTGTGAAAGGATAACTAATCGGCGCCGGCGGTCGGGGCTTTCTGGCTCCAGTCGCCGATATCCTTGTCCCGTCTCCACTGATTTCTCTACGTCTTTCTACG

>DS499595:2449494-2449895

CTTCAATAGCCAGCAAATCGAGAGCTGAGTTATATCAAGCAATGATGCAATGATTGCATAGCTGGCTGATGATCAGATGCTCTTCATCCTCTTACGAACACGTTGTTACCAGTACAATATGCTGACTTGGCTCGCCAGTTAGCAGTCGACTGACTCCACCAATGCCTGGAATTGATTGCCATCTCGGCGGAGTAGCGGGAAATCTTCTCGTCATTTGGGAATCAAATGGACGAGAATATTCCGATACTAGATTGATCATTCACCGGAACCTGGAGGGGGAGGAAATCTAATAAGATCATCATCATCACCTTCTCCTCGGCCTATTCCAGCGCGGTCCCCTCGTCCCATGGATCGACGTTAGCAAGCCCCGCACCAAAAGAGATCATCGGAATGATTGGATT

>DS499595:2676925-2677326

ATCATGATGGAAATCGCTGTTTGGTTAGTGAGATTTGTTATCACTACCTACCTTATAATACCTACCTTCTGTACTTCGTGGTAACGATGAATTCTAAAATCTTCATATCATTTCGGCTTCCAATAATTTTCATTCCAAATGCTAAAACTACCCAAATCCGTGCAACAATTCCGTGACGAGTGTGGGTTGTTGACAGGGAGAGGTCGGGCGTGTTCCGAAGACAGTTGTCCGGAAGTACCAGGGTGGTATACTTACCGCATACAGACACGTAAAAGATACTTGGCGGTGACTTGTTCACAGGTGACGTGCCACGGTTCTTGAGAAGCCTGCCAATCTTGAACCAGAACGCAAGCGGTTGTGTGCTTTTCTTTTAGTTAAGCCAGATTCTGGCCTTTGATTTA

>DS499595:2681544-2681945

GGGAGGTCATCGTCTAAGAGGTCCTTGTCTACCCCTCTAGGTTGTAACAATAGTGCAAATATTGTTATCTATAAACCCCTGTCAATCCTTGAACCGAAACGGCGCAATGATCGACAAATTGAGTAAGCTACACGCCGTGGAGCAAAATTATACTTTCCTAGCCTGGATAACCTCATTGAGCCACATGGTTGGATACTTGGTACTCCGTAGTGTGTTGTAGCTTGCAACGGACCCCACGACAGTTTGTAGGCTGACGCCTGAAACCTATAGCCTGAGCATGTGGATGCCGCTCTTTCAGTGAGAGGATACCACTAGCAGTCTGCCGCATTGCATGTGCTGTAGACTGAGTGACTGTCGGGTGACTTTTCGGCAAGGCAAGCACGGAATAAAGCTTTGTAATC

>DS499595:2705836-2706237

GCCTGAAGCACCGGGGCTCTCTTATGGTCCTCCATGACGACTACAGGCAGTGCTAGGCATTGGAATGGAATAGAATGGCGCTTTCAGTGGGTCTTGGTAACTATATCCAACGAATACTATTTGCACATTCCTCCGTTCCTGGCCTCACTTCCTTTCTCCTTTCCGTCAGTCACTTCCCTACATTTCTCTTCCGTTCTCTCAGCACCGTAACCGATCGAAATCCAATGTTCCATTACCCCTGGCTTTTCCCCTTTCATCAATCCGTGTTCACTTTGGTCTTCACCGAATCCGAATCGTCCTCACGATACAGGATTGGCCCTTTGGGCTCGTCTTCGGGCTCCGCCTCGGGCTCAATTCTCGGATCAGGTCGGGAACGGTCGACGAGGACAAGTAGCAGACTC

>DS499595:2809128-2809529

GGTATGTGTTTCACGCCTTACACTTCATCCCAGAAATTGTGTTTGATGGGCCGACGAGGAAAAATAGAAGATCTAATCTGAATATCCCTATCCCTAAAACATGTCATTGAGTCTAGACTTGTCTTGTCAATATCTGCTAGTCATGGGATGCTTCTCTTGCTCCAAATCGGCGCAACTCCGATGCCTCGATGCTGTGGCTGTCCGGGAGACTTGTTTGGGATATCCTTGGAGTCAAGAGTCAGCTCCAGCCTCGGTACACCTCCTTGATACGTCCATGTATTATTAGGCTTAGGCTTCAGAATTTCCTCCCCTGGATAGCTGGACAAGAATGGAAAGAAACGGAATGAGACGGATATGGATCCTGGACGATGCGCCTGTATTCTTAGGGTGAAGCCATCCCG

>DS499595:2841407-2841808

CCTGGTCATTTCCAGAATTCGGCAAGATTCCGTTGCCGCTTTTCCATGTGGATCGATTGGTAATGGTTACATTGCATATTGACCATGCTAGGTACTGAGGTAATGTCCATACCATACAGAACACCTCCTGGTATTTCACGGTTACTGCGTGACATTGGCAGAGTTCCGATTTACCGCCTTCGGGAAGATCAGCCACGTGTGTCTCCAGCCAGTGTGCTTTCGGAGATGACCAAAAGTCCGCGGGTTTTTCCTATCAGCCGACCTTTTTATCATTTGGCGTCGGAGAGCCAATTGCGCGTTTGAATCAAGCCCATATGACCCTTGTTTTTCCGTTGGGTTTGCCTCCAAGCAAAGGGATCCAAAATTGGACTAGTATCGCTGATCTTCATATTTATCTTGCG

>DS499595:2951335-2951736

TCTCTTTCCTACCCTGGTCTAAGTAGTCTAGATACTCGACCAGGATCCATCTGACGTACCATGCCACATGCAGGCTTCGGAGCTTCACCGGACTTTCTGGGATTGGGATTTCCAGGACGTAATCTTGACAGCTCGGTGATTACCGTACAGATTACTTGACCGTTTTAGACCCCGGTGAGACTCGGCATGCCGCTTCACTCAACTCGGTAAGTGACGGGCCGACCTGTGCAGTAGTGTGGCCGTCGAGGCTGTCTTGCAGAATGGGTTGCGCATGGTGGCAATGTTTTTTGTAGTAACTGCGTGTCTCATACATGGATGTGGTTTTCTACACAGGGTACGGAGTACAGTATCTGAAAAAGCGCAAAATGATCATGCGACCTTTACAGTCCCAAATAATAGTT

>DS499595:3005516-3005917

AGACTCGGCACGTTGAGAGATTTTTCGAAGACACTCGGGAAGACAATGCCGGAGGGATACCGCAGAAGAACAGAACCGTTTAGCTAAGGGGGGTACGGAGTACCCTCCAGTCCAAAGAAGGAAAAGAGACGGGGAAAATTCCCTCTTCACCGAGGAGCCTCTTTTGGCCGCTGAGTCGCTGAGAGTCTCCGGATCTTTTCCTTTCTAGGTACCACTAGTCTAAAGATGGTGTAGTGGTCTTGAAAAATGGTATGGTATGGAAAAATAAAACTTTGCGGAGAGAGGGGGACCTCCCCAAATCTGGGAGTCGCATTCGCAATTTCATCCCGGCCTGGGAAAGGACCGGCTTTTCTCCGAAATCATCCGATCCCAGCTGAGCTGGCTTTGGATCTGTGATCTCG

>DS499595:3012044-3012445

AGCCACGGCATTATCAAAATACTTGCCTCGGGCCTGTTGCTGTTCGATCCATATGATCTATGATCTGGACAACTATGAAGTCTGAAGAAGCATATCCAGGTGCCGTGCTCCGTACTGCCGACCGTGACGCTGATGCAATGCCCGAGCTTCACCGCAGTACTTCAGTAGAGACACAGAGTGACCATTCCGGCTTATTTTCGTACTCCAACTCAGCCTGTAGATGTGGGCAGGACAGCCACATCTGATGATGAATCTATGAGGTATTGGATCTCGTTCATTTATCGTATGACTATGCCAGATTGTATCTGCGCTTCCCTTGACACTACGTAACCTATGATCGCTGCCCCCGTCACGCCGGGGATGATGTTGCTTTTCTATTCGACCTCTGCATTGCTTTGGCT

>DS499595:3016038-3016439

CTCTTTCCTTTGTGAAATAGATCCAAAATGGTAGTGAAATGGGTAGCACTGTTTCGAATCACGCTCATTCTCGCTCTAGAGTCGTACATTGAGCATCTTTCCGGTACGTCCCTCGAATGAATCTCCCGATAGCTGGACTGGCTATCATGTGATTCACATGTGATGAATCTGGACCAGAGCCTCGAATTGGTCGATGGGACCTAGGGTTTACCTTGGGGCAGGGACCAGCTCATGAAATCCGGCCAATTTGTGTTTATCTTTGCATCTGATTTGCATTTTTCAGTTAGACCTGGCGAGATCACTTTCAAAGGTATCCTCCAAGTACGGAGTACTCCGCTTGACTTCAATAATGAACTCGGCTTGCTGAAGTGCGATTGGTGCCTTCAATCACGCCATGTAGA

>DS499595:3027831-3028232

CGCACGGCAGAAGCCAGAGTAACTTTTTCCCTTTTGGGCAGGGGGAGATGAGACACCGACAACCTCCGAAGGCGCTGCGGTTTGGTACGGTATCAAGCTTACGACGACCAGAGGACATGCCAAGCTGCCAAGGCACTGCGGGGGCTTTTTGTGGTACCCAAGCGGATATGGTTGGCTGAAAGGCCGTAAAAAGAGGGCCATGATCCGCGTTCGGAGGTGCCATCGCTGGCTTGGCAGCAGAATGGCTCGATCTCGTATGGTATCATGCGTACGCCGGATAGCCGGTGAGGATTTCCTGCATTTGGCAGTCCTTAGGAGAAAGTAAACCCATCCAATGACTCGACGGATCTTCTGTGATCTTAAAGGTCGTCATTATAAAGGACACATTACACAGAGATTAA

>DS499595:3187595-3187996

AGAACAGAAATAGAAAGAAATTCGAGTTCGTGAAGCATGTGAAGCTGTAGATCTGATATAGCATGTGATTGCGGCAATGTTCTTCATGTGATTGTGGATCTGATTCCAGCCTAATTGTACAATAGTACCAACATACAATAATAGTAGCAACAATACCCGATTTCATACAGCCAAGCATCCATGCATCGGAATCATCTGATGCTCAGCGAAGGTGGCTCCTGTATTTAGCTGGCTCTTATGCATGCTGTCCATACATCCGTCAACCCGTCAGACCATTTGCCGGGCCAATCGGGCCGACCGTTAAACGCACAAGGCAGCCATCAGCTTATTTCTAGCTCCTAACTACAGCTCTTCAATGTTCTAGTCGTCCAGCCTTAATGGGGGCTATCCATCCCTTACTG

>DS499595:3291491-3291892

ACTGTCAACTTTTTAATCTATGAATATGACATTTGGATGATCCATATAGAAATCCTTGGTTCCTTGGATCCTTGTATGGTGTGCAAAAGGAGGCCGAATCAACAATGCATTGGTTCTGCATCCTGATGCATGATCATACCACTGCTAACAGATCAGGTCACCTCTGAAGCTTATGGCTCATGGGACATTTGACAAGGTCAGTCTATTATTTCCATTCCTTCCTGTTGATCATGAAATACATCTTCGAAGATGACAAACCCTTTTTTACTTCTTTACTCTCCGAGTCGCCCTTGACGAGACATTTCGGACCTCCCTTTCCCGGTTGGTCTGCTACTTGCCAGTATATTTTGTCTGAGAACTGTCTCAATTGCTCTTGAGTTTGCATGCCATATAGTCGGAAT

>DS499595:3341540-3341941

CTTTGGTCTAGAGCCCACCACATGAGTGGATGTCCTCCGTACATAGCAGGACATCCTCAGAGCTGGACCAGACGAACAGCGTGGCCTGCGTACCGGACCAATCAGGGAACAATGACTGAGTAATGGGTGCAGGGATCATTCCACCAGATGGCTGTAGTATTGGCGAGAGGCACAAACCGTTGATGGGCGAACAGCAAGACGAGCTTGGAAAGGCGACGGAGTCTGACTCCACTTGACGATTGCTGGAGCTAAGTGTATACCTGGGACTTGGTGTCAATATCCGCGAATCATCAGCAAGCTGGGGGAAGACCAGAGAGTCAGAAGACTAGGACGAGTAACTTCCTTCAGGCAGCAAGGCAGGAAATCCGCCTTGCCAGCAGGCACCTACACCAACACGTGAC

>DS499595:3355610-3356011

TACAAACAGGGAAACGCGGCAGACTTTGGCATTCGGGCACCCGGACATTGGAGAGGTGCGTGACCAGAAAAAGACGGGCTAGACCTCGGCGGGTGGCAATAGCCCATCTATTGAAAGACCAGCTCTTAGTGGCTCCTCGCTCAAAAACAAAAACTGCCCGGCCTAACTGGTATTATTGGCAGGATTAATGGTTCCGTCCAGCCGGGAAGAGGGGGAGTGGATTTTGTCTGCGGTTTTTGACTTTTCGTGGGTCTCCTGATTGCGCCGGGTCGCTCGGGGAATGCGGATTCCCACTTCGCTGTAGGCCCGTCCCAGCTATCCGAGAGACTGTCATCTGTTCTAGTCTCTTAGTGGTCGGTAGTTCTACTAGTGCAGTGAACGGTGAGAGGTTCGCTGGGGAA

>DS499595:3389597-3389998

TCAAGAAGGCTGCAGAAGGACGGTGGGGGAACGAGGAAATGCGTGGTTGGTGTAGGATCACTATATGGTATACCTACGTACTTGATACCTACATCGCACTTGCTCCTTCAATTGATTCCTCATTGATTTCAACAATACCAAGATGGCTAAGGAATCCGAGAGATGTTGAGTAATAGGAATACGGATACTGCTGGATAATTCTATGATGCTTGTGGAGTTTGTTTTGCGGTTCTTTACTCTCTGCCGTTACCGCCCGAGGTTAGGAGGGTAGAGCTAGTATCCACCAATACAGTGGGACAAGTGGGAAAAAAAAAGAGTCTGCCTGAATGACCAGGACAAATAGAAAAAAGACAGCTCCCTAGCTAGTATGCCAAGGGGTAGTTCACTAAGGACATCCTAAG

>DS499595:3391374-3391775

CCGTTGCAGTACCTCTCTACATCATAGACAGAGCAGTCACCGCTCAGCTCAATAGTCAATGATCATACTTCTTTGCTAAGTCATCTAATGACCTCACTTGACCAGATGGAGGAAGTGTAAATACCAGATCGGGAAAAACGCGGATGTAGCGGGCGATCTAACCAGCTGCTAAACATAGCAAACCTTGCATGGGGTTTTCGCCTATGCAAGGATTACTTGTTTCCTATTGGACAGATACGGCTGGGGTGGATCACCCCACCGACCAAGCGCCTTCCAGGTCCAACCCGTCCCCTTTCAAAGATGGATGGCCTCGGCAATGATTTGCTAACAATCAGTCCGACGTGGCACGATGGAGCAGCAAGAGCAACTGTTACCGTATTAACTAAGCCTCTGTATATCTT

>DS499595:3445393-3445794

GTGACCGAGCGTATCAAAAAAGACAATCGGAGCCGTAAAAACAAGTAGATTCCAGACCTAAATTCTATAATTGGCATCATCGTCTCACCTGGACTACGGGTGGTTACTCATCCATAAATGGATAGCCTATACAGTGCAAGTCTTCAGAACAGAAGTTGAATACTGATGCCGGTGAGGCCACGCTTGTCTCAGTAAAACACACCAATCGAGATTGATAAAATTGCCACCTTGAACAAGATTTTCCGCGCTTCAAGCCTTGATTCGATCGTTGATCCTTTTCTCTTAACAATCATCTGTCATATGTTAGATGTACAAGAGTCATGCAGTGCGATACCTGCATTGGATGTCCCCACCGGATTGTTCTTTTCAGTCATGGTGCTATACAGACTCTGCATGTCTAG

>DS499595:3469297-3469698

AAGTCAACACTAGTACCTTAGTACGTGTATAAAATCGGCCAGGTAAACACAAACAGCTCATTATGAACAAGGGATTCCTTCAATTCCCGAGCAAATTCAATTCGCTCAACTCGAGAGCCGGAACAACTGGGCGAGGTAGTAACTCGCGCGCACTGACATGAGTCCCTCGGTAATTGGACCGGTTTTATGACATGAATATCCTCATTGATCGGCCGTTGACCTACGGAAAGCCATCCTCACATGCAGTTGAGAGGATGTGGTCATACCACAGCAAGCATGGGTGCGGAATGCAGACTCCGTCAGTTGAGCTGTCAACCTCAGCTGTGCTAGGAGATGCACGTGACTGCGATTTTCGCTTGGAACTCGCGAAAGGGAATAGCGTGTGGGCCGTGCAGGAAAGA

>DS499595:3585440-3585841

ATGAAACACTAAAAGACATCAAATTTATCAACACATTTCGTGCTGGTATTCTGCTGGTATACTTATTACTCAGACAAGAATCTTCCAACAAGGTAAAATGCTGTAACCAAAATCGATGGATGAGGTGATGGAGTTTCCAAGCTTCGCAAGCGACAACCCAGAACCCTGTCAATGGCCTGGCCCAGTAGCCAAGGAATAGCTGCAGCCACAATGTCCGTGCAGACTGGTGTACATGCACGGGTAACGTCCAATGAAACAACGCATATCCCAGCTAGAAAAAGTTAAACTGCCAAGGATTGACTTTTTTAGGACAACTCCTGTCAATAGGTGGTGGGAACAAGGAAGTCTCGGAGGTTTGGATTAGGATCCTGACGTCAGAGAAAGACAGTTTCCAAGGAAAA

>DS499595:3587427-3587828

AGATACCCATGGTTTTGGTGTGATTGTCAAACCACTGAATGTTAGAGCGACCGGGATAGGTAGGTAGCAAAGGGAAAGTCAGAGTCATCCAGTCCTCTTGACACCCGGAGTGCAAGGCTCCCTGATAGATAGCCATGGCTCCACCCATCTAAATGGATTGCATTGACTGGGCCTTCTGGGGATCGTCCCACTGTCCCAGACCGTTCGATCGTTCTTAAAGTTCGCCTGTAATTACCAATAATACCCCATTTTCCCATCATCACCTCATCTGTCGCCCTTTTCCGGGCGCTGCGGCAACTATCAATGTGCAGTAGTGCCTCTATGTACATACTCGGCCGGCTTCTCGCCCTATTCAACGGTCATGGCGGATAGAAAGTTCCCCTCTCTTTAGCACCGTACAA

>DS499595:3795079-3795480

CTGTCAGTCAGATGCGAGAATTTTCTCTTGTCTCAGCTCGTAGCCTCGACCACGGAGGAGACCCCAACAAGACTTGATCTTCAGTAGTCAAGCCAAGCCACTGAAATCATTTTTCCCTGGCAATTAATTTATTTTGGGGTTCTATTATTACGTACAGCTGTGGAAATTGGATCATTGGATATTTTGGCCGAAAAATGGCCGGGATCGTGCTGGTAGAACTGGCCCGAAATGGCACGAGGGCGAGCCGACAATCTTGCCCGATTCCGGCATCTCCAGGTTTTCTTCCCGACTCCGGTGTCTGTTGTTCGACTGCGTACACCTCACTTTAGCAACACTTCTCCAGGTCCCGCATTCGGCCTTCTGTCGCTTGTTCTTCCCCAGTCAGCAGAGTCAGCCGAACG

>DS499595:3844404-3844805

GGATGATATGATAACAGTCAAGCACGAGGTGAACGGAGATAATAATGATGATGGGGTGGTCCCAAAAGAGTATCAATCAATGATGATTGGGTTGGCTGAAGTACAAGATCGGTGATCTGAAGCCAGTGACCAACAGTGAACGAGTCGCAGGCCAAGCAGGAGAACAACGAGAAGTCCGTGAGCCTTGACTGGAGGGCAAGCAAGGGACTGGAGAGGCCGGATGACCAACGAGAGGAAGCAGAGAGAGAAAGCCGTAGTGACGAGAAGGGGCGAGCGATGGGGCATTTGTACCGGACGCATCGAGAGGGGAGCTGACTGGCTCGCCGGTTCGTTTAGTGGTCGAGGCGGCGAGGATTCAGACCAGCGAGTGGTCAGAACCCTTACCGTCTTGTACTGTCAGG

>DS499595:3880978-3881379

CATTTAACCGTTTGTGTAGCATAAGATCCAGGACCTGCTCAGTCCGCATGAGTCTATGACACATGAGCCCCCAACTCGCCCGAACATCGACATTGACAATACCTTATGAATCTACGTATTTTCCCAGTGAAAGATAAGTATTGGAAGGCGAGATGAGGGAACTTAATGCTAGCTGGATCAACAGGTCACTGGCCACTGACCACATCTATTTACTGCTTACCTCGAGCTAGAGAATTAGCGCCCCGTAGCGCTTTCACGTGGCTACAGTGAACGATCGCCCTCAGGGGTCCCACGCTTCGCGAACTGCCTTAAACGGTCTAGTGGGGTCACGTTCAAATCCGTGGCAAGTGTAGTGGGCCGTGGTATTTGCACTGGAGAGGAAGAATGGTCTGTCCTTCCCC

>DS499595:3881623-3882024

GACAGGTCGAGGTGCGGCTGCTTGCGGGTGGCTTGAGCAGTTTGGACATCATCGAAACATGAGATCTTGGCAGTCTCCTTCTCAATTCTGTTTTAGATCTTCTCTCTTTTGATTTTTTGGCTGACGCGGTAGGACTCTAGGGTTGTAGGACTAGCAGTGGCACATGTTTTGACTTTCCAGCAACGTACAGACACATGGCGACCATGGTGGAATGATCGCCGCTAAGGAAAGCCGTCTGAGTCTTAGGCATTCGAGTATTGGGCGTTGGACTCCTATTGGCGCAGCTACTCGGCTTCTGTCCGATACTCCAATTCATTAGTATTCATTATTATTCACTGCACATTAGTGAATCAATCCCATGTAGTTTAAGTCTTTCCTTCAGGAGAGACTGTCCTGATACG

>DS499595:3993943-3994344

GCCTTAGGCGTTAACAGATGAAAGTTTATTAATTCTCTTCATAATTCTCTCTGGCTATCCACTTCAGCGGATAGCTGTTATGACTGGAAGTACTTCTTCAATCATCCCCTGAGCGCGCCTACAGCGGTACCAGCGAGATCGCCTTTTCCACGGCACCCTCCCAGCCAGCGTGGCTAGGCTCAGCAGCGTCAGCAACACTTTGAGCACGTAGTACTGCCACAGGTTGCAGACCAGAAAGTGACCTGACTGTGCCCTTCGACTCGAGGTCTGCCACGTTGCTAGCCTACACCGCATTGGGATCCGTCATTACCTTGCTGCCAGTCGCCGGGCGTCTTAGGCCAACTCCACGCTCAGATGACGCTCTAGCCAATGGTCAGATATGTATTTTCGAGCGCTTGGAG

>DS499595:4015915-4016316

GAACAGACGAGATTAGCATGGGGGGGGGGCCGAATCTCAAATTCTGGTGGCAACATTGAAGCCCAACAGATATGATATGACACTGGGTCACAGATATGGCACTTGGTATGGATTGATGGGATCATGGAAAGGGCTGGAGGAGGATGTGGAGGATTGGTTTGGAAGAAGACGGATGATAAGCTTGCTGGGGCGCTCGCCCAGGGCCACTTTTGAGGCTTGTCCGTGATAAGCCAACGATAACCGCCGCCAATGATGGTCGAGAATCCGGAGCGGGAAAGCCGAAGGAACTTGGCCTTCGACTGTTGGACACCCAAGAAAAAGGGAGGAGGGGGGAGAAGAAATTGGGCTACAGCGGATAATTCCTTGGGATTTTCGATTACTGTAACTCTGTAACCTCCCCC

>DS499595:4026269-4026670

CGTTAAACCAACATTCCATGAAGATCCCGTTGCCTTTAGGAAGCAAGAAAGGTCAGAAAAGAATGGTCTAAAAGAGTAGGAGCTTGCCTTTAAAGATAAACCATGGATCCATCTGCCAACCATCTGCAGCAATACATTCAATAGTTGTTATATTCTCACCCTTCTCAGATTCAGCAAGATCAAGGCAAGAACCTTTTAATCCAATCACATTCCTTGCCTTGCCTTCGCCAGGTCGGAAGCCACATTCATCAAAGTTGTATACCAATCGTGGTGGTGTATCTTTAACCACATTGGCAAGCTGATTATACCAATTCGCTAGTAAACCAGCATCCTCAGCCTTGATACACTTTGATTCCTTCGTCTTTTGCTTCACAGGGCCCAGATTGAGGTGTTCTGGGAGT

>DS499595:4274640-4275041

ATTATTTTGTTTCCATGTTTGACGTTGTAGTCGCATATCTTCAGCAAGTTCAGTTTGCCCCTCTGTTTGCCCCTCCATAGAGGGCTATGGCTCATACCCCGCATGATCTTTGGTGTCCGACGGGTTCTCAGGTAGGAACCATGGATACAACGATGGACAGCTGGAGCTGTTTGGAATTGGCTCGCTACCAGGACACCAATCATGTGACTTCTTAGATCAATAACACCATGCGGTTGTCACTGCAACTGGACGCATGTCACAAGCAAGTCAGAGACAACCCTCAGAGATGGAAGGGATTCCCGTAAAATCCCGAAGATTCTTGGTGGGGCTGTGTCTTAGTCACAAATACGGAGTACGGAATAGAGAGGTCCATATGGAGTAATACACCAGAATCTGGAGTT

>DS499595:4281558-4281959

CCACTAAATACCACGGATAATACTGTATGGTGTACAGAGTACTCCGTACGTTACGGAGTACCTACTTACCTACCTAAAGACGCGGGTACCTTGATAACTCAATGCGCGATTTACAATACGGAAACAATACCGGTCCACACAATGGTCGAGACTCGAGTAGTCGAGCCACTGATTCCATCAATCTATGGACCCAGAAAGGGCCCGCACCTCCATAGCCTGACTCTGAGAGGGGCTTCCTAGATAGAGTCTAAAGACCGCTTACTCATTAGATTCGAGCCTATCTATGTCGTAGCGTGCCCCGACAGCTCCTAAATAACATCCAGGTCAGCCCCATTAGGGCAAAGGGAAGGCACTTCCCGAGGAACCGTAGATACAAAACACTGAAAACGACGATATCATAT

>DS499595:4355228-4355629

ATTACTCAGCAAGGTGGAACTCATGAGCATGAACCATCATTCTTCTTCTTCTTTTTTTTTTTTTCTCTTTTCATTTATCCATCTCTTTGATTTCCTAATTCTTTTGTCTCCCGAGGGGAAATCGTGATTGCTCCGTAGATCCGGATAAAATCGTTAGATAAATATCAGACAGGGTCATGGTGGCATGACACGGACAAAGATACAACGGCTGATGGTTCTAGTCATTTCAGGAGATGATCTCATCAGTCCTGGACTCCTCATAACATACTCTGTACCTCTTTAGAGACTTCATCCTAATACAGTGCATGCCTCAAAGATCGTTATCTATTTCCTTGGTGGCTTCCAAACCCAGGCTAAGCACGTCTCTAGTCTGCCAAGTATCTACATACTCCATAATTCCA

>DS499595:4551419-4551820

GGAGACTAATTATTAGCAGATGCATTAGATGCTTCTCGTCTCCGACCGTGATACGCCGATATGATGAGCAGTGCAAAGCTAGCAGGGATTGGTGTCCACCACCCATTTGGTCAATGCAGCGGCAGGCCGCGATAGGGTACCTGCTGTTCCGTCAGAGGGTGGTCATGCTGTGGCTGGGCCTGTTGAATCATTGCTGCGTGTCACAAGTATCATAGGAAACTGGGAGGACGGTACCCGCAATCCATCTAAGCTAACTTGCAGATAGTGTATTGCACCGGCTCAGTATGGCGCAACCATCCCGTTGAGGGTGCTTTGAGTCTTCGATAAGGACGCTGCAGAATGTTGGGTCACTGATGATGCCATTGTGCGCGTCATCTCAAGCAATGACCATCAAGCGGTCC

>DS499595:4553636-4554037

CGAATCCTTCATTGTCCTGTCCATTCCTGCGTGGAACAGTGCGGATTTACCGCCGCTCTTAACTAGTCCATGTTCTGTTTGGGGCATGTATGCGGAACCTTCCGCCACCCACATGAGCCACGGATTGATGGACCCAGCCTGAGGTCTAAGCGTACCGACCGAGATCCCATCCAATCCTCTAACCGCTCGCCTCCTGAGCAATTTGAGGTGCCCAATGGGCTCTATGGACAATTACCCATATTTCTAATCCCCCTGGTCCACCGCCCTTCTCGTCCGAGGATCATTGACTGAATCGGTTGGGTGCGTTGGTAGTGCGAGTCACGAGGTCCGGGGTACTCGCCGATGAGATTATTGTTATCCTGCATTTGTCTCATTATTATTAATGCAGTTACATCCGTCCA

>DS499595:4651763-4652164

GTTTCAAAACAGCTAAGAACTTCTACAAGACCGCCGAGACCGAGGCTCGATCACTCCCTGCTGTGCGATATCATTGCATAACCATAACTCTACAGCGTCAATGTTACATCAATGTCTTACCAAGCAAGCAAGCTTATTTGGACTCGTCCATCAATGCTTTGTCTATTTCGGAGTACTCCGTACTATTATTGCACATGATCTAGTGACATAAACCAGACTATGGGAGGATCACGCGATCAACTGCCCATCTGAGTTCTGAACCCACCGGATATTGGTCCGACAGTATCACCAACAACGGATGGACGATAGAAGGGTGGAGATGAGCCTAGTCATGCCTTCTGACTCCTGAGTGAGAATCTTCTATAAGGCTGAACATACAAAATACAGAGTACAGATACAGT

>DS499596:193517-193918

GTTTCTTGCTCCGAGGCTGACCCGGAATGTCGGCACTGCGCTTAGGAAATTCCGAGCCCATAGTCTGGCGCCTTACCCGCTGGAACTGGGTCTGATTCTAAGATCTATACCGCATCGGAACACTAGAAATCGATGCCGATGAAGTAAAAGTGGGCTGACACTTGCAGCATTGTTGAATTAATAACAGGTGGAGAACTACATCGATGGGTGAGTTGATGAAACACATTCCGCGTCTCCGGCGGACAGCGGAATTGATGCGGACTCATATAAACCTGAGGCATCAACATCCATCTTATTAGTTAAACTGATCAGTTTGACCAATAGAAAATTTTATATACAAAGATCCACCACAGGGTGTCCACCAGAGGATCTGTCCATGACTCCATGATCAAGTATACACT

>DS499596:196207-196608

GTTGAAGCCATTATGTACCACGGGCGTATTTCTTACCTGATTGGGATGTGAGGGGGAGGCCCAAGGATATCGGAGCCTCGGAATTTCGGAATCGGTTTCACAACGGAAGCTTTCCGTGCCTTACGTTGTGGTTGGTCGACAGTGCCGGAGTGGGCTGCCCCACTCTGGGAGCGCCTAACCATTTCTGGGATATGACACGCGATCGGGTTCATTGCACCTAGCTAGATAACAGAGACAGAGTCACAGTCTCACAGACAAGCAGTTGAGAAATCATGGGTTTATCTTTGGATACTTTCGACGCGAGCATGTGATTAAGGCTAAATTAAGAGTGACCCTCTGGCCACCAAGGTACACAGCAGATACTTCTAGGGGAATTGTCAACGGATTAACAAAAATAATTG

>DS499596:209086-209487

TGTTTGGGCGGAGAATCATGCAATGAGCCTTCAACATGTATGACAGCGCATCGGACAAATGAACTGTGATCAACGTAGGACGTTCCATGGACAGCATCTCGCCGTGCATTGAATGCTTCGAAGCTTCACCAAGCTGCCTAAAGCTTGCTCTTGCTCCATGAGACTCATAGTGGCGAGGCCGTTTAAGTCACGTGACCATAGAATCACCACGAATCATCGAGTGAGCTCCAGCTATGTCCAGTTGGCTATGCTCGATGATTCATGATGCATCACGCAACATCCAGCGCTCTCGAGCAACACTTCTGAAGTCATTGTGATAGAATCATGGGAGATTCTCGAATCATGGCTCTGGGGTATATAGAGACCTCCAGCACCTGGTCTACTCGAGGTTCACTTCACCA

>DS499596:218340-218741

ACCTTGGGATTTTCTCCATACTCTGGGTGGATGCTCCCCGTGGATGGAGCACAGATTTCACTATGGGGACTCAAGAAGAGCAGACAGTTCATTCTGCACAGCGATGGCGGTGGTCCTGTCAGCCAATCATACCGCGTACTATCCGACATCGGCCGGGGAGCTCAGCACCAACTCCGATCACGTGCCTACCACAGCTCTACTGTCGTCCTCGACAACGAGCTTATAAGTCTCACCACGTCCGTCCTCAAGGAAACTTCCCTTTCCTCTTTTATTATTTTCTTGCAAAGCATCAAATCCTTGACCAATCTCCTCTCCTCTTCCTTCCTAGACTCCCTTTTCCTCCATCCTCACATCCTACGCATCACAATGGCTTCCCGTGGACTCCCCCGTGCCCTCCGTCT

>DS499596:276479-276880

GTGGCCTTTCTAATTCGGAACCTTGGAAGAAAAAGCTGCTTCTCCGGTGGGGGATGGCATGAGGATTAATAATTCTCGCAGGAAAGACAGGATAGAAGGAGGTTGGCGTTGTGAGTTATAAATTGGTCGTGTGCTCAAAGTACTGAAAGAGATTGACCGAATAAGCCATGACTGGGTGAGGAATATTGATGGCCATTCCAGAACAACCCAAGTCTCCCGGCAAGGACGGATCATTCATCAGAGTGTCAGGAGACAATATATCCTGGCCAATGAATAGAGATCTTTCAGACCGGAGAATAAGGGGGCGAATGTCCCGTGGAAAAATGAGGCCGATATAGGCAAACGCCTTCAAGCTAACTTTTCGCAGAATGAGTGATGATTATTGAGCACTGGGTGGGCCA

>DS499596:302982-303383

CTTTCATCGCCACCTGTTGATCTCACTTGGGTCAAGATCTTTGGGCAGATTCAATATTTCTCTCTCTTGCCGTCGTTGCCGATCTCTTTCAGCCCTACGAGTCAATGAATACCCTCTGCGGGGAATCAAGAATTGGTTTGAGGCCAAAGGCCAGATTCCAGCGAATCAGCTTTCGCTATACGAAAACTGCTCATAATTTGCGGTCATGTTACTAGAATCGTCTGGAAGCTAACGACTAAAGAAATGTAGATGCCTAAATGAACCGAGTGTTATGACGCATTGCCCGGAATGGATGTTTGCCCGGAATGGATGGCGCCCGTGAGGCGCTCTTCACTGTTGGGTAGATCATGCATTCCACATGAGCAGAAATTGTGAATAGTAGAGGGCACGGGTGAGTTGAA

>DS499596:317650-318051

CGATGAATGTTTCTATTGGCTATGTGCCAGTCTGAGAGCTGCGGGCTTGCATCATCCGCCGATTCCTTCTCGAACAATTATGAAGCACTACGGAGTCGATTGGATGAGAGCGTCTCCCGCCATGTATCTCGTGCAGGGCAAGATATGCGGGAGCCTTCCAAGATATCCCTCAATCTGCCTCACATACAGAGTTAGAGCAGAACATCATAGAAATAATACTTATGGTGCGACTCATGCTAGAGAGCGAGTCAGATCCAGCGACAAGGTCACAAGGCTACAAGCGAGAGGATACACTCATGCCGTCCCTGATCGGCCCACAGCCAGTGAACGGCGGCTGATGGAACACGCTGATCAGCCAGACTATCGGCAGCTCCTTTACTCTTGTGCCCCGCTTTCCCTCA

>DS499596:384822-385223

CTAGACTAGGCTTTCAGGAGGACCATGGAAATTCCACTAGCCAACAGTATCACCATTTCATGAAGTTTGCACCAATCCTTCTCAAGCTCCAGCGGAGATATCCCGGATAAGCAGCAACAACCAAACTCAATCTACCAGACACTAGCTGAATTTAGGTTACACCACATCGCCATAGCGTTGAATCGGACGCTTATTTGTAATCATCAGATTGTGCGGTGAGAGTAACTTGCGAACTCACCTTCCGTTTTTGAGTCATGAGAACTCCTGATCAATAGTCTCACAGTGGCGCCAGCCCCCCTCTCCCCCTCCAAAAAAAAAGAATCTCTTTGTCGTAAAAAGCGAGTCCGATGGCCCATGCAAGATTGGAGATGAGATTAGATCAGGTGAGATGAGATCCATGC

>DS499596:505889-506290

TAGTGTAGATAACTGACATATGAAGTGTCGGCGTCGACCTATACCACTTAGGAAGTTGATTTGGGGATTCTGGCGCGCACATGCCGATATCCGAAGCACTGCCTCCGCCCGACTGCGGAATATATCAATTAGGTTCAAACTATACTCATGCTGCGCCCTGAAACTTTCCCAATGTTGTAACCGCCCACTTGACCACTCTGCCATCTCAATACGAGGGTTTTTATCACAGCAACTAGCAGCCTTCTTACGTCACAGCTCCATACCATTAGCTCAAGGGAGTTTATCTGATTTGCTGGATAGGCTGGGCAATGCATAACCAAGGGCCGCTAGAAACAATCTGGAAGGCAATGCAGGCTCATGTGGGTGCACGTGTTGCCTGACATTGGCGGTGGTGCAGCAGA

>DS499596:555165-555566

CAACTGGGAGAGGGCCAATATCTTCATCATCTGACATCAAATCAATATAAATGACTCGAAACCCGTTGACATGTCTTATCAGTTCCCTCGGATGAACGTCAGACAACTAATATCTCTGACCAACCGATAAGAACCGTAGATAGCTATTGCGAGCCGCGTTTTCCTTTCTCTCAGTTCTTCTTCTCTTTGCACTGAACTGATGTCTAGGGAAGTCCAATGTGTTGCACAGTATCCTGTCCGATCATGTTATACTTCTAGTGTACGGAGTAACCAAATTAGGCCGTTCAGTTTCGTGCTTGTGGACTGTGTTACTGAGTTGAAAGATACAGACATACTGCGTACTTCTGAATCTGGATACTGACCATGATCCAATCCATTCAGTATCGACGGCCATAGACCGC

>DS499596:582646-583047

CAACTCGTAAACCGTACTCTGTAGTCTGGTACTACAGCCTCGGAAGGTCACATTCGGAATCATACAGCCAGCCACTTAATGTACTTACTGTGTATGCAAGACGGACTTTTAGCTTGGTCAAGCCTAGCTGGTGCTAGTAGCTACTACCGTAGCTGCCCTAGCAGTACTACTCTGTACCTCTGCAAGTGTTACCTGATTCCAACCCCAGAGAGTCACTCCATTCTCACATCAAGTGTCAGTGCTCGGTCATTGACGGTGGGCCAGTTGACTCTATTTACAGCACGGACTACGGACTAGATGTATTATTTGGTACTGTACTGTGTAGATACCGACCAGGAAAGGCTCTTTTCGAGTTGCTCACCCAGTCATACTCTGAGAACATTTCAAGTTCGAGCCCGAAG

>DS499596:636598-636999

TCGCTTGTTCGGCCGAGCTAAAGCAGCCCGGTTAGCTCGGCCGAGGATCTACAAGCCTGAGCTCGGCCGAGCCTTCTTGAGAGTTACCGTACATACTCAGTACTGTACTCCCGTACTCCCGCACCTTAGCGTAGGCCGCCTGGGCCTACTGAGCCTACTGCGGCCCTAATTGTGTCTCGGCGGCCACCTCGCCAAGCCCCGTAACTCCAAGCCTCTAATGCTTCTAAACCCAGACTCCCTCCGAGCACGCCTTCCATAATCTTCAACCCATTGTGATTCCAGACCTTTTTACGAGGTGAGCTGACCATCGGCCGGTTTTCCGTCCTTTCCCTTCCCTTCCCTTCCCTCGTCTCACTTACCGAAACATTCTATCTTGGTCAGAAATTAGACTCTTCGAGCTA

>DS499596:748668-749069

ATGGACAGAAAGGAAGTGGGGAGTGTACGTATCGAAGAATTGAGCATAACGACAGCGACTGAAGTTAAGCGAAGCAGATACAGAACAATGCATGGTACCGGCAATGCTGGATCTGTCTCGTCAGAGATATGCAAATATTCGGTCGGAGATCTCACTACTCACTCGAATCCAGCAGCACGAGGTCGGTATAGACATATTCCTCCGTAACGCAGCGCATACAGCACTAATGCCAATGACGACAGAGGTCAGAGCAAGGCTGGCATATGATACATCCTTAGCCTTTAAGCAGCCTCACCAGCTTTGGCTGAAACCGTGGAGGAGAATACAATACAACTTCAAAACTTCATGTCCCAATCATGAGGCTTTCCAAGGAATAACGAGGCTGGGTTTGTTCCAGTTCC

>DS499596:749498-749899

AACACTAGCGCACAGAACCAGCTGATTCATGACTTTCTAGTCAGGGGTCGCGTAACGTCAATATCACAGGGTTAGGAATGAATTACACAGACAGATCCTATCCCACTTACTTTAGAATATGGTCTCTTTTAGGGCGCGCCACACTCTCCAATCGAATTGTTTTATGAAGTCAAGCCCACCACATAGATAGCCACTTAGGGCAAATCACCACGGATGGGCGCTCTAAGTGGCCCATTGTATGACGCACCCCGTAGATTACCTTAATACCTAATCACCTCATCCTCAAATCCGGCCGAGTCCGGGATTTTGAGGAACAGGGTCGCTTCTAGATGCGACATAAGACAATGCCAATGACAAGAAGAGAAATATTAATTTAAGATGTTTACTTTGTAAGGTGGCAG

>DS499596:860433-860834

TGCAGTAGCTGTACGGAGGAAACTACTCAGAGTATACTCTAGTCTACGGAGATCGAAATAGCAATAAACTGAGCAACAACTCAAGCATTGATGTCTTCTATCAGCAGCCAATAATCCTCTTGACGTAGATAGTAGGAATATCAGTGGATGGTTCAATGTTCACAAGACCCCAAACAGGCCAAAACCCAGCTCCCTTGCCCAACCGCCAAGGAAGCTAAATCCGAAGCCAGACTCAAAAAGTCGCAAACTAGTCAACGCCAGAACTTTCCTAGGGCCAGTCAGCTCGCCCTTTCCGCCCGTGGCATGTCTACCAGGATTTGTTGTTCGGAGCCGGGGGCTGAATGGCTGTGCTCTGGGCCCCTGGTGGAATGTGAAGGTACTGACTGTGTTTACGGAGTACG

>DS499596:1019442-1019843

ACATTCTTAGACGCATCCATCAATTAGCGCCCACCTTATCGGGTTCTAATTGCCTTCCATCTGTATTCGAATTTTCTCCATGATTCCAGGGCTTCAGGGCCAATGTATGTATGAAGATTATTATTTGGGACACCCAGCAGAAATCATATGGTCTGACAGTCAAGTCTTGTTAGTGTGGATGGACGATGATGTCACGTGGGTCTCACGGCCACTGAAGATTGTTCTAGTGGGCGCTGGCGCGTCTTCAGGTTATCGGCAGTCTGGGCGACACTTACGGTACCTAACCGAGCTTCCTCAGCCGTTCTGCTGATTCGACCAAGACGATACTGTGTATATACCGCCCTAGTTGGAAATAATGAATGAGGTTTACTCTACTAGTCATAAATGGAAGTCATATCCTG

>DS499596:1066871-1067272

CAGCAGGTGATTTCAGCAGTGGACCATTGTCAGACTTTGACAAGATGCTTTCCTGCATGGCTCTAGATGAATTCCGATCTCCGTACTGGACGACGAAAAGAACGAGCAGTGGTGCGTTGGCCGGCGGTGACTTGCTGCAGACGATGCCCATGTCTTCCAGCCAGACGAGGGTGGGGACTCGGAATGCACAGGAATTTCCCTGCGATTCTGTCGGAGCTGATGATCTAGTCTTGTTTGACTTCTAGGCCAGTGAGAGAGTGTGACAGAGATGCTCTGTGAGAGGCCAATATCATCAGATCAGCAGACTTTGGGCATGAAACCATCGTGGGACACATCGGGAACCACTACGATTCGTTGTTTACGGCCTCCGATAACCTTAGGTCGTGTAGGATGACCATCCA

>DS499596:1099445-1099846

TCCTCTTCTGATAGAAATTAGTTGTTAGTACTCAGATCTCGGGATCTCATCCTGCAAGATCATGGTGTTACTTCTGTGACGACTAGACGAGAGTATCTACTCATAGCACACACTAATTACACATTAAAATGTTCTTTGTAAGTCCCAGGTTGCAAAAAATCCAGGTGGGGCACGGAAACTGGCCCATGAGTGGCCGACTACCATTCAATATGGGCCCACCTGTCGCCCAGTGGCCACCCAGCTGGTCCGACATACCCCAAGGAAAATTCCTGTCGTCGTCACCGTGATCTCATCCAAGTTTTCCCTGCCCCGTCTCCTCTTCTGCCCCCGTGATTTACCTTCTATGCTGCTTCTGGACTCTCGTGTGCTGGGTCTGGCTCTTTGATTTCACTCTTTTCGAG

>DS499596:1133095-1133496

TTTTCGGTCAGAATTTGATTGTCCATGGGCGAGACTCTCAGGTTCAGTTGGCAAGCTCCACATCGGCACGACCCGCGTGGAGATATCCATTCCCTCCAGAAAGTAAAGGAATGCTATGACATCATGCCAAGGCCCGAGGAACAGCCAGGTCCAGACTAAACGGAATTATTGACGTCACCATCCTGAGGAGAGTTTGGCGCATCAAGCGGCAGAATCCGCTTTCCTGATTCTGTACTCCTTACTCCGTACCACAGAGAATGGCGGGTTTTGGCCACATCCTTCCTTGACAAACACTCTTTCTAATTTATAGGTAAAATGGAAACAAACTGTATCATATGGTTGTAACCAGAAAAAAAAAATATAAGGAGCCCTCCTGACAATACAAAACAAAAACAAAAAAT

>DS499596:1212895-1213296

TCAGGTCGGCTGCATATTCAAGGAAGGCCAATAGCTGGACGATTCGATGGAGAATACCTACCACATAGACCAACTACCATTTTACACAGGACTCCCCAATCAGTCAAAAAGTCAGGTCGAGAAATGTCAACCAAACTGAAAGTTCAAAGGTCAGGCGTCCTTGGCTCCAGCTAAAATTCTTTTTGTGCATGCCCATTGGAGGAGCATGGTCTAGACTGTCTCAGCTGCATTGTGGCTATTGGCCAGCTGACTCTTAGTTGCAGCGCTAGTCATTACTCCGTACATTTAATCCCGGCACGGGTGTTTAGGCTTTTCCAGAGATGGTATGGTGGTACGGTAACGTGGTACCGGGTAAGGCCAAATCTGAGACCTCCGGACTGGCAGTACAACTGTCCCTTGGG

>DS499596:1221139-1221540

CCCCTGACACCCCAGAAAAGCCCAAGCAATTCTTGCACATTGTAAGTATCATGCAAGTGTCTTCACAGTTTGATTTAAGGTCGGGAGATATGGCAACTTCTTTTGGCCTTTTTGGCACTACTGCTACCATCTCTCTTGGCACTAACGGCAAATACATCTATTCCTGTCCTGTTGTATCCATCTGTTCCCCCGTGACCATCAGCTGATGTGGCGAGATAAACAGGGGAGAGGCAAGAGGTCTGATGGTAAGACAAATTCGGTTGTGTGTGCATGCTTATATTATGTGGTATGAGGGCCATCGTTGTATTAGCTGCTCTGTCCTCTGTCCAGTTGCAAGACATATCGAGCATTCTCCAAAGGCCTGTCATTCTGAACAGTAAGGGCAGATCTGATTACTCCAT

>DS499596:1221900-1222301

ACAATACCTCACTGGCATCCTGTTAGCGAGCTAGCTGCGAGATTACCCCACGTAATAGACACCCCTCATCCCCTGACTGTTTGCCAAGAGTGCAGGGAAAAGCTCAGATCATCAGATGATGTGCCCCGTGGCTGATCCTCGTGATGTCGCTCCTTGCCTGTGTCGTTGGTTTTCGGCTAAGCCTCTTCAAGACTAGATAGATGATGATAGGCGACAGGGATACAAGGGACGTGTCTCCTTTCTTCTTGCGGGAAGTGCCAGGGTGCCAGGCATTCCGGAGGTCTCCTGTGACAGTAACAGTTTTTGAGGTAACTTGGCCCGCCGCGGAAAGTCATGTGCCATCCCCCAGAATCAATAGAGGCAACAATGCTTCCACTAGGATTAAGTACTCATAGCCTCAT

>DS499596:1345543-1345944

TATATGAGTTGCCTCCTAGGCGAGTAGCTAGGTACCTAGGTTTTGCGAGATGGCTGCACCTATCGAGTCCCGACAGTTCTCCCATGATAGGCTGAGATCAGAGAGGCTAGCCTCCGTCTGGATCCGAACGATGGCAACAAAACCGGTTGCCGTGGCTGTACAAACTCCAGAACCACAGGAAGGACTCGATCCGCAGATCCTTTGATTATTTGCTGTTTGGTCTGGGAAATCCTGATAAGAGGCGGTGAACGGTGTGACAGGTACATCTACCTTAACCAAGGTATGCAGATGCCCCACCCCATACCTGCAGTGGCGCAGTCTCATCATTGGCTGAATGCGGAACCCCATGGATGATGCATTCCGCACTGGGTGTTATGATTGGGGTATCCTCCACCAGGAGG

>DS499596:1346886-1347287

TGAGGCAATCATGGAGGGCCGCCTTGACCAATGATTAGCCCAGTTCGTCCATGAGGAGAACGCCCAACCCCAGCGAATGACAGCCAGCAGAGTTGATGATTGGCCCTTTTGTTTCCACGTTTGCTGTCTGAGAGTTTGCTGGTGGAGACGCCTAGATCGGGGGTGTGGAGCACACTGGCTGTGCTATGCTACTGTCCGAATCTAGGTCCACCTGGTCGCATAACTTGTCAAAGCAAGGCGTCCATTCCGTGTCTGACAGTCTGCATGGAATATGGGAAGGATCACGTTACTGTACGGAGACTGAGTGTAGGCTACGTAGGATTAAACCAAACCAGCTGGTGCACAATGCCCCCCCCCCCCTTCCCCTCAATGGAACCAGGGGACATACAGCGCCGCATAAA

>DS499596:1398709-1399110

ATCAATCCTTAAGCAGATTTGATCCACGAAGGTACTATAGCCAAATTCACCGGTTCTTCAATCCATTGAATGCCATGCACGCAGCATCTTGGTATTCAACCGCTCAATAAGTGAGTTCTGGCTGTGCTTGTCAAGCATGAGCATGTATGGGTCAATGTAACTCACTGATGCCACGAATGAAATTCCATTGAGAAAGCGAGACAATAACGCAGCCCAATGTCAGACCAGGCCAGAACAGTCTAAGATAGCACCTCGTGGCATGATCATCATCCAAAGTAGACTTGTTTCTGACTTGGCTGAGCTAAGGCACTTGGCGTAGGTAGCTAATACCTTCTTCGAACACGGCATGTCAGCTGTCAGTGATAGATTGCTAGTGATTGATTGCTCTTCTGTTCCTTACT

>DS499596:1509572-1509973

GGGCATGACAGATCCCGTCTGAAATCCCTAGAATGTAAGGTGCGCTTACTCCGTGTTTTACCGTCCACTAGCTATTTGAACATGTGGCCATGGAAATGTGATCGTGATTGGGAGACATGATACAGCCAGATGACGGTACAGCTGAGCCCTCAGCCAAGTGAGTGTTGCGATGACCATCTCTCCGGGGCGCAACAATTGGTTCGCAGGCCATTCTTCGACATGTTGCATGATGTAGTACGGAGTAGGTCAGGGAGTGGAAGAAAGGTGCGCAAGGTATCAGTACATTGTGAACTGTATGATGGGTAAATATTATATATTTCAATCGCCCTTTGAACTAGTTTACTTCATTCAGATCCCGCATCGTTGATTTTCGATATGTTGATTGGGGAGTAAGAGCACTC

>DS499596:1511037-1511438

GCCTTGGGGACTGGCTGAAATATCTTACATTATTACGGAGTTACATAACCAAAGGATCCAGGGATGGCCGGCTCTTGGCGGCAAGGCGCTCGGCGTTTTCTTTGGTCCGCTGCGGATGACTGTGTGATCATTGTGGGTTAAGCGAAATTGAGCCTGAAGATCCGCAGCTTCCCATTGGTACCTAGCTGTTTAATAATGGATCTCAAATGTTAATCAGCCTCAAAGTCAGAGACAATGATTGGATAATTCTTGAAAGCTCAGAAATCGAGCGTATCGCAGAAAACACCCGGTACCTTACCATACCGGTCTTACCTAATTTCCCAATCTTCTTTTCTTTTTCCCTTTATTTGTCGAGCTGTTGAGTTTTGTCACATCCTCTTGTTGGTCGGCGACAGAACTTC

>DS499596:1593337-1593738

GCAGGGGCCAGTGGCCAGTGGCCAGTGGGCCAGACCATGTCATAGAATCGGTCGTACTGGAGTCTACAGTCCAGAGTCTGCAAACAATGCGGAGTAATGGACATTGGCTACGTACGCCAGCGCCACAGTGGGACAGATCTTTGAGAGTGCGCAAAATTGTTTCTCTGTGTGGATCTGTCTGAACCAATGACCAGCGTCTGTGGCCACCAGTGCTTAAATAAGCATTGTGCCGACTAGCCGTATGCGAATGCCTTATCTGGTTGGTCAGTATGGGCAGTTGCTGCCTTGATGCCTCATTCTCTCTCTTTTTTCATTTCTTTTTCTCCCTCGTGTCAAGACGACTAGGTTGATCCTCTGTATTCTGGAGTCTGAACATGTTAATAAGCAACGCACGACGCGTC

>DS499596:1605857-1606258

TTCAAGGTGCGCTTTGATCTGACATCATCGTATGAAGACGCCGAAAGGCCGCAACAACGGCCAAAGTTGCGCCGCCATGCCGAGAAACGTCGTAAGCTGGGGCACAAACCTTGTTATGTCTCGCTCCTCTGTACGGAGCACTTCTTTACTCCTAGGAGATAGTCGGTGCCACCTATTGACAGGGGCCAAAGAAATTGCTCTGCCCAACGGACCCACGACGCCTCCGAGAACCACCTGACCTCGTGTTGCTCTTCCCCTTCAGGGAAAGTCAACCGAGAATGGGGCCGATCGGACGGATGGTCTGATGCAGAACACCACCTATGGACTGTGGAGTCAGTTAAGACCACAAACATCCCAATCCCGTTACGGAGTAGTTTCATCTTCATCCCTTTCGCTTCTTG

>DS499596:1885264-1885665

AGTTGATTGACTTTTGGGCCTAGCGGTATTTTACCTTTCTGAATTTTCCTGCATTTACGCAGTATTGCCGGCTGCCTAGATTCTGGTCTTGCAATTCTATTAAGATGGAAAGAGATGCTTTGTCACACTTGAGCACAGTGGCAGATTCGGGGTCCGAGGAATTGCTGGTATATTTGCTAAGTTATGGTCTCCTTTCAACCATTGAAAGAGTACGTAGTGTACCTTAGTCTACAACCAAGATAGTCGATTGATACCAAGATCCTACTAACCTAGGTACCAGAATAGACATACAGAGTACCAGAGCCTTCTACGCACTCGGCAGTCAGGTGGCTTCAGTAATAATATAGCGCCATCATCCGACTCGTGGGAGACAGTTCAGTCAAGCAGCACAGCATGGCAAT

>DS499596:1971520-1971921

TATGGATGTGGTTGCTTAGATGGTGACGTCTCCTGTGGTCGCAGCAGGCTCCGGACCTGTGGCTGATGGCCTGATGAGTTCGACAACCCAGCCAAGGGGGAATCTAGACTAGGGGGAATCTAGACCCCCGGAAACTCGGAGAAATGAAGGATGCCTAGTGCATGATGCAGGTCAGCGCAGTCTGCCAGGCTGGATGCAGGTGAAGGTATCACAAAGCGACAAGAATGAACTTGAAATATGGAAACTGTCCACGAACTTGGCAACCCCTGGCGATGGCGGGTAACGATGTAGTATTCAGTTAGTCAGGGACAAAGAATCGAGATGCATCCACTAGCCACTTTTGGGCCTTTAGCTGAGACCTAATCCCTCCTGAGCTCGGAACCAGCATCGACTGACAAATA

>DS499596:1988796-1989197

AATCACACACGCCCTTTGCAACCCAATCGGGCTTGGTGCAACACCGTCAACGGGGATCGGCCTGCAGCTAGCCTTTCTCCACGTCTGACTACTCCCTTTCCGGACTAGGGGGCCGCTACACATCCCATGCAAAGCAACAGTGGAGATCAGGCCAGGGCCTTGGAGTTGATATTAGCCGGGGTGCCATTGGTCTGGTTCTGATCTCGGGCATCACTGCTGTGGAAAGCTCTGGGGCTTCGACCGAGCTAGGCTAGCATTTCGGGAAATGAATCATCTGAAAACTGAGGGAGGAGGCTTGCATCCTGAAGTTTATACCCCTTGGCACTTCTCCTCTGGCCTCTTCGCTTATCAATGGTGGTATTGCTTACAATAGGTTCTTCGGAGTACTCTCACCCGTCTTC

>DS499596:2002505-2002906

ACGTCTCTGGGGCTGTGATCCTGCCTGTATTTACCGCTGTATTACGGGTCGAAAAGGCAAATTCAGGATCAAATTCAGGCTGGGATAAAGATATCAGGGGTATCATACCCGAGAAATACGACATCAGTCTAATACAATTGCCGCTTTGGCTCCGTTCCACCCTCTCTGGCCCTCTTAAAGGGGGTTCTTTTCGACTTTTTGGGGTCGTTTGATTCAGGTATACTCTGTAGAATGTTGGCTAAATACGGAATAGTTTCGGAGCATGCAGGTCTGATGGAAGTTGTAAACTTTGAAATCTGAATGGGTCGGACAGTTCAACAATCCGAAAATGAGGACCAACGGAGTCGGACACTTTGGGACCCCGACCAAGCGTCAATTCAATTGGAAGACAAGGAGGCACC

>DS499596:2003377-2003778

ACAAACTCTCTCGTCCAGTAAGTGACGGCCTCGTAGCCAGAACTCACGCAACGACTACTCTGTAGGTTAATACCTACCTTAGGCAGCTCAATTTCAAGTGGTTACGTACTTGGCTGCACCCCCAAAATGTCAAGTATGTGCGGAGTCTTTTTGAGCCTCATCCCTCTTTCACAGGGTCTGGCCACTCTCGGAAAGAGCCTCTGACATATGGAAGGGAGAAGTTTTGTTGCAGCTCGACCGCTCAAATTGGGCCTTGATCCAATCAAAATTCCCAGATTATCACCAGATGTCGGTGACGGTTTATTTTGGGGCTCTTCTTTCCCTAGTCTAAGACTTGAAATGAACAACTGGCATCATCTGATACACCTTCTGCTGATCGGCTCACAGTTGGGCTTCAGTTT

>DS499596:2049281-2049682 sph3

AGGGAGCGGACCTGGACCCAACGCACTGCCGGATGGCAACTACACGCGTGAGATTCCCAAGCTGGCTTCGTACGAGAATGTGCGGCTGCTAGGCTATGTGGCGACAACCTACGCGAAGCGCAATATCTCGCTGGTGCGCCGGGACATCGAGACGTATGCAGCGTGGCCAACGAACTCTTCCAACCCGGCTCTAGCTGTTCGGGGGATCTTTTTCGACGAGACCCCTCAGCAGTATGATGAGGATGCCTTGGCGTATCTGCAGGAGTTGACCGACGTTGTGAAAAATACCCCTGGTTTGGGGCCGGATCATTACGTACGTTTCCCTGGTAATTCATTATCATATTTGGTAATTTTTATTTCCATTTTTATTTATTTTTTTTATTTGTTTGGTTTTGTTTCCT

>DS499596:2053868-2054269 ega3

ATATTATACTGCCCATTGACAACTTTCTGACAACCAACCTGCAAGTCTCAGTTCCAAAACCCCGTGTCAAGAAAGACTTTTCAGCCGATGGAACATGGCCAATCATGCACTGCCAGAGAATCCCAATATGGGGCGCTTAGCGCCGGACGCTAAAGCTTAGCGTGGATCGTAGTACCGTACCTGTAATTTGTTTTTGTTGCAGATCGGGCGGTACGGGGTAATAAAATACAAGAACCAGACAGTCAGGTACCTAGGTACAGTACCTATCGTCAGGTACCAGGTACGTACGGTCGGGCTAGTGAGAATGGCTGACTCGTCCCATGGATATCCACATTCCACACGATGCCACGTTGTTGACACTGGCACTACACTTGACTACTGATACTCCATACTCTGTAACG

>DS499596:2055045-2055446 agd3

GAATATTGGCGTCGATTTGTTTGCACAGAGTCATGCGGACTGGACAGTGGATAAACATCTTGTGACGAGTCGATTCGGGATGGCTGCCACCGGTTCGGAGTATACTCCGGACTAAGTCGCTAAGATCCAGTGTGGATTCTAAGATTCTCTAATTCTATATTCTATGGTTCTATGTTCAGGACCAGGGACCAGGGCAAGTCGTGGATTTCGCGTGGTGTGATCGTTGCATGGATGCATGGGCTGATCCCGCCTGGTGCGTTGGGGATATTTCCTCCAACTCCATCGTACTCCGTAGCCAGGTTGATTCAGAGCCTATGAGCCATGGCGGGCGAATCCTGACAGCGGATGTTACAAACAAATGATTCCCTTGGTCTTGGTCCGCCGGATGCTTGGGAATGTCT

>DS499596:2058815-2059216 gtb3

CGATTCAGACTGCAGCCGAAGGAATTATTATTAGTATCCCCAGTGCCAACCATGATACCATGCCAAAAGAGGGGGCTTGAGGCCTTCCATCATTCCATGATCCTGGAGGGTTGAGACCTCCGCGTATACCTGGATACAGGGGCTGTATCATTGGTACATACGGAAATACCAACTATCTCGGGTTTGCAAAACTTTCGTTACCCATACACACGTTACCAAACAATACCCGCATACCCGCATACACCATACATGTATACATACATACAGAGTACATGGGTACATACGTACTCCACTGGACTAACTTAGGGCCTTGACCTTGTTTCGCACTTCTGCATCTTTGAATATGAGGAATCCACTTCGGCCGCCCTCACTCAGTTCCTCAGCCCACAGCCCTCAGTCCG

>DS499596:2119306-2119707

TCCCTTAAAAGATCATGATGATATGGTATCTTGCCAGCGGTATTGCGCTTTGTACGGAGTAGCATCGTTGCTGACCCTTCTATGCACGATGCACTCGCATCATCTGTTTCTTCGTAGAGCGGCGAATCGGTGCCGACTACCGAGTTCGCCCCCCCAAATCCTCCACCCCTCCAGACCATAAGTCCGACTCCATACAGATAGTACGAAGCAGAAGAACATCTGGGGGCACAGTGAGAACAAGGACTCTGGTAGCATTACGTCTGTGATACATGAGTGTAAGATACTGCCGGGATATCGGCCTGCCAGCTCGATCAAGCCTATCTCGGAATAGGCGAAACGAAATTCCATACGGGATGAGTGGTTACCGTCTGCGGCTGGCGTCTCGTTAATTTTGCCCCGTC

>DS499596:2386043-2386444

CTGACCAACTTAAAGATAGGTCTATGCTGTAGGGCTTCCAATAACGAACCCCATTCTGCAATGAGAATATGGTAGAATGATGGGTTGACATCAGATGACGAGTTCGCAATGATGTATACAGGAATCAGATGCGTTTGAAGTGTAATTAGTCAGACGCCAATCGCCAGCAGCTGACATTATCATAAACGTGCTGCGGAATCAGGCCACTGAATGCGGTTTGCCTCCCTGTGATTAATGTGCTCGGGGTCTCGGTGTCACTAATCCAGTAGAGTCTTGAGAAAAGGTGACTCTTTCTGCCTGTGATGACCCTTAAGCCTTCCTATTGAAGGTCTCGGACGGTCTGTCCTTTAGATTATGCATCCGAACTATCCAGCTGGTGCATCAACCCCTTCATTTAGTCT

>DS499596:2387680-2388081

TCTACTGACGCGGGATTTGAAGGTTTCGGTAAGAGCGGGGCCGCACCAAAAATTCAGGGCAACGGTGAGGTGAGTTTCTGGACCGTGGCGTCTGGCAGACCGCCAATTGGCAAAAGTGCGCTATTTCTGGCGGAGTCCCCAACGGGAAACTCTCTTCAGGTACGGAGTACGGGAATGAAGTAGGAGTACGGAGTACTAAGGTACCTACCGAGTATGGATGGCATGGCGCCATGAGAGACTCAATAGAGGGACGTGTACAGAGGTACGGAGCACTGTTGTCAAGGTGGGAACAAAGACGGCTGGGCGATGCAGGTAGCAAGTACACTCCGTGGCGCATGTGTACGTAGTAAAGTCCCCTGTACTTCGTGCACCGTTTGAATGATTGACATGGGAGGTCCCGT

>DS499596:2501387-2501788

TTGCCCACTTTCGGGGATCAGTGGAGTCTTACACATTATTGGGAGAGGGCAGGCGGGACGAGTAAGGTAGGTAACAGAAAATAGTTAGGCACTGCGGAGTAGTAATTTGTTGATCGCACGCACATCTGGTCATAGGTTGGGCCATTGCCTCGTAGGCACTTGAAATCTTGGGGCTAAGGCGATCGCCTGATACCCTGAATTCTTCAAAAAGTGCTGGGTTGTGGAATGATTCGCCGAATAAGCCGAGACCACAATCAACAGTTGACAGGCGGATTCTCCGTAGAGATGGAGCGGAAACACAATCACAGAGATCCAAGTCCCTGAAGACAGGTAAAATTGCGTGATAACTATTGATCAGTATGATTTGTTGGCCTGTTTCCATGGCCATGAACCACCGGATT

>DS499596:2638689-2639090

ATCATAATACTAAGTAAGTAAGTATGAATAGGTCATACCTTAACATCTCTGAACAGTTACCCGTACAATACGCCGTATATACAGCCGGTCCCCGACGACCGTCTGGTATTACAAGTACTGCAAAGTTCAAAGGGATCGCCGAACACAGCTAGCCTAGATCCATATTTCCTCTTTGGGTATAACTGGGCCCTGGCTGGGTCTCTTGGAGTCTTGGGCGCTGCGTCAAGTAGAACGGATTCACCACTACCTTGCCTTGCCTTGCTGTAGATATTCAACGCAGCTTCGGGATGGTCGTTGCCAATTGGTAACCTTCGAAGCTAATCACAAAAAGTAAATATTACTCATTGCCTGAGAAATTTCGAGTAATCAAGAAATATCTCAAGTATTATTCATAATCTCCC

>DS499596:2641783-2642184

CTGGTCAGCCTCACTCACAGTCCGAGCCCGGTTTATCTCCAAACCCCGAGATTTCAGACTGATTACTATTCCATTTGCTTGGCTTACAACTCAGGAGGCTGGAATCCAAAGAAGCCAGCGGACGTGAAAAAAAAAAAAAAAAAAAGTCCGAGATGCCGACCGGCCGACCCAGCTTTTTACGGAGTAGAGTTGGTGGCTCCATTCCAGTTCCCGATCTCCCATCGGAACATGGCACCAGTTTCAGGTAATTTCAACTCCTCTATCAGTTCACATGCACCGGTTTCACTTGCTAGTTATCTAGATGCTCCGTAGACTACTGCGCAAAGGACTCTTTCTCCGTAAGTCGGCGAAATCCTAGTTTACTTGTAAAAGTTGTCTCCACGAGGGTGTAATGTTTGTTT

>DS499596:2644972-2645373

AAAAAAGTTCGATGCTCGGGGGTCCACCCACATGAAATCGAACGGCGTCTGGCAACGGCGTCTGTCAGGCCATCTGACGAGTTGAGCCTGCTTAGGGTTGAGGACCTATACCGCGGAGAAACGGTCATGGGCATAGTTCGAGGGCCTGTCCCATCCCATACTTCGCGCTGAGTTGGCAGCTGGGCAAAGTAGTATTAGTCTGGCGTACTCCGTAAAATGGCTCATCGTTGCCGTCCCTGAGCTGCCAAGAGCTTCTCGAAGCCGACTATGGGTCTTCTAGCGCGCAATGATCGTCCATATGATCGTGCGGTCTGCACATATCGACTGGCTGTGCTCCTCTGTCGTCTGTGCTATCCTACTTAACAGTGTATAAAGATCATATGACCACCAATTCTCGGCGA

>DS499596:2774795-2775196

GGCGTTGTTCTGTATCTACTACCTGCTCTGGAGGGATAGTTGGGATAGCTTCAGCAGCCTGATCCCTGCGTTGAATGATCTCCTGCCCTTCCTGGATAGATAATCCCACTGCAGTTTCTATCTGCCGCCTAGATCGCTTCTGCTTTTGAAGGTGCTTTTCATGTGCAGCACGTAGATCATGATTTTCCTTTGCAAGAAGGGCAGCGTTATTCATTGCCATCTCACACCCCTTGATAATCTGACCTAGCACAGCCTTTGTAGGGGTAGGAGGGCTGTATGTACGCTCCCTAAGTAGCTTCTTAAGCGTAGTTTCCTGCTTCTTCAGCTGCTTGAGATTGTAAGGTGTTTTTGGGACAGAATTAGTTGATCGGCTGCCTGGTGGTGTAGGTGTTCTAAGCTGG

>DS499596:2849851-2850252

TAGTGTACCTACAACATGGTCCGTCATCCAACAATATGTCCCGAAATGATAATATCAGACATGCGGGCTGGATCCCTGCACACATCAATCTGTTGAACCGGCCGGAAGGAGGAGGAAAAAGCCGAGATTTTGGGGCTGGTTGAATAGGACATGGCCCAATCAGGCATGGCGGGTCTTACAACCTCCCCAAAGGTCCAACGGAAATGGTTGGGTTGAACACGATAAGGTTGTGACAATACATGACATGACATGACATTATGTTCAGAGGACATCGGCCAGGATGAGACTTGACGGCAAGCCGACTATCATGTGGTGAGTGGTATACTGTAGAGGTATGGCATGAGATTCACTGTTCCCCTATATCTCCTAGCTGATTTACAGGTTATAGATGGTTCACTCTA

>DS499596:3141710-3142111

AAAGGGAACTAGAGCATGCGACCTACCGCAAAGATCTGCCCACAGTAATTTAGTGCTCATTTACCAGTTGCCTTGGCTTACTTTTAATGACCTATTCTGAATGGCTGAGGCCCTGAAACTAGTGTGATCTGGATGCAGCGATAAGAGTATGGATGGGACCACACCGTTTGCGCACTAGCCTCTGATAAGCCCATATAAATAGTATCCCACCGAGAAGGGTTTTCCGCTTGCGTCATCCTGGGAGGAGTACCGCCGGTTACAGGTTCAATACGGGTAAGACACATCATTCGAAACTATTCCATGGCTGACTCCCTCAGCAGTACCAAAGAAAGTATCATCTAGAAGATCACTGAAGAGGAAATTTCCCCTGCCAAACCTTGCTGGAAATGCTTTGCAGGAAC

>DS499596:3144639-3145040

ACCACTTGCCAAGTGTGCGGCTGACTTAGCGTGCTTCGACCAGACGATCTTGAAGAGGGCTGCGCCAGTTGGCGGGGCCTCCCCACTGCCGCCCGTATTACCTCCGACTACGAGTAATCTCGGCCGAACAAATCTCCAGGCGTCGTCAGTGATGGTTTTGGTGCTGTGGATAGATAATCTGTAGTGCGAAGACAATCTAAGCCCCGAGGCCAATGGCATGGAGCGCCAAAGGAAAAGAGCGGTTCGAAATAGCGTCTAGATTTGTGTTGCTTGTCCCTTTGGCATGCAAGGGTCAAAACGCACGGTGACCCTTGTGTTACCAAACCTGTCATGGTGTACGCGACCGGGTAGGTAATCATCGGCAGACAAGGATGACGCCTGAGGGCCCAGACCGTATGAAT

>DS499596:3159753-3160154

AAGAGCTCCATCTGGGTGCGGCCGCTAGCCTGGCGTTCCTGTTTATCTCCCTGGCAGCCTCATTGGCTTGCTCGTACTGCAGACAAGGAGACGAATCCACAATGGGACTATCCCAACATGCACCGCTTTGCACTACCCTGTGCAGACTACAGAGTCGAGCTCCACCAAGACCTCCAAGAACCACAAAGGCTTGGAGGATGCTTCTTTGGCCTTGGGTTTCTTGTTCTGCCCGCTGCCTAGTGCCCACTGTCCCGCCACAATGCTTATCCGAATGCAGGACTAGTCCTCCTTTCGCTGCCTAGAGTGACCCCCCCTCAGCTGATATGCTTGGCATGACATGGGGGGCCTGTCCCAACAATCGGGATATCATATCACTGGAATGGAGCCCGCGGCCCCCGAAT

>DS499596:3191135-3191536

GGATGGGGTCACAGAAGAAGATTTCTCACCGAGTCCACTGTAAAATCAAGTCCGAGTCATTCCTAGGCCCACTGCCAAGTCATTATCAGATCAAAACAGGAGAATTACTATACTCTGTATCAACGCTAATGCCATGTCCACATTGAAATACAGCGTATGGTACGAGTGAATATGCCTCAGCTTGTATTTCCGTGAAATACAGTCATTAGTGACTTTGTGGGTTCCCAGTACGAAGAAATCTCGATCAGATAACGCTGCCAAGATGCCAAGGGACAGGACAAGGATTCCAGCCTTACGGCGACAGAATTTTTCGCATCACTTGACCTTCGAGTTCTATTAGGAAGAAGAGGATAGTCTATGATGCGGTAGTCAGAAGCCCTTGGAACTCACATAGGGCCAGA

>DS499596:3193998-3194399

TAGCTAACGACGGTGTGGACACCTTCCGATTGCGATACACGGCAGCAAGGTTGACACACTGTTACTAGTCAAGACGACAACATGACTCTCATCCAATACAAGGCAAGTCGCACCAGTACAGAGGACAAGTGACAGGGGTATGGATGGGAGTGGATGCTGTAGGGGGAGGTCCATCCATACCTAACCATCATGACAGGTTATACAGCTGGCCTGTATTTCACCGTATCATATAATACTTGAACGTATGAACTAGTAATGTGGTGGCATGATACACTGTTATCAATCAGTGGAGACTCCCTATGACTGGGTCATTACGAGAAATCTCCGGGACCTATGATTTGATTTACGGATCAGATAAGTTTTACGTAACATTACAACGAGTATGTCGACCGTTGATCGTC

>DS499596:3205432-3205833

TTATCTGATCCCACTAAAGCATTCTCAACCAGTTAATAGCCATTCTAACAAGCCTGGCTTGCGCAGTTCTTTTACTATCTGACTTTGGTTAGTGATATTACCAGTAGAGTGGGTAAGCATACATACAGTATTACTGAGTACATCAATACACGTCGCGTATGGTATTACACTCTCGTACGAAATAACGTCAGTACCCTCTGTTCACAATCAATATTCCCTAGATAAGAACTGTATAAAATGGCGCACTTAGCTGTATTCCTTCATCCTGGCAAAATTGACAACCACCTGTTAGCAGTTTCACAACTTAGACGGCCTCCACTGTGGCTTTCGCTTCTCCACAAATGCTCGTACTCCCTCGTGGAAATTTTCTCCAGCCATGAGCTTTGCATACCACTGGTCAA

>DS499596:3208876-3209277

TTTCTGCCACTGGCAGCCCGGTAGGGCCTGACCTCCTTGGTTGAATACGTAGAGTGCTCCATAGTTGGATCAGATAACCGTGGCGTGTAATGTCCGGTCTCATTACAGATGGTAAGATTACCCGCCAAACTTATGGTACAATACAACGTGTTTAAACATGGTGTGACATGTAGGAGTACTCGTCTATCGAACTTTCTTTAGTCACAAGCCTAGGCCAAGGTTGAGAAGCTCATGACTGTGCATGTGCCTGTGCGTTCTGCTGTTCTTGGGCTGTAATGATGGAATCGCTCGTCTCTATTGTCTACCGCACCTATTTGGCGTCCAAGTCTCCAAGACCAAAGAGTCGCGTTCATGAACCATGGCCCCTGGATGCATTTCCTTTGTATATCCAATATGAAATA

>DS499596:3223496-3223897

GAGAATGATTGAAGGAAAAAGAGGCGAAGGATTTAGCAGGATCTTTATCCTTGGACGCCAGGTCGGGATGGCGCTGAACGGGCGAATCTTGCGTTGCATCGGCGTTGATCTAATGGGCCCAAGACGCGTTAAGGCGACGCCCAGTCGGAACGATCAGCCAATCTAGGGCGCTAGCGGGAAACAGATACGCTGCATCCCTGCATGGAAATGGATCGAATCATCCCTAATAGAGGACTACTATTGCACCATTTGGTTATAGTGCCGGTTAACCGGCTGCTCAAGTTCTTGTATTATTAATCAGTTGGGTTGCTGTATCACATCTGCTCACTATGGATTCTTTGTTGCCAAAAGACATATAGATACAGTGCTGGTTCCTCTCTGTTTGGGGAGGGGGAATGAGT

>DS499596:3275323-3275724

GTTAAACCAACATTCCATGAAGATCCCGTTGCCTTTAGGAAGCAAGAAAGGTCAGAAAAGAATGGTCTAAAAGAGTAGGAGCTTGCCTTTAAAGATAAACCATGGATCCATCTGCCAACCATCTGCAGCAATACATTCAATAGTTGTTATATTCTCACCCTTCTCAGATTCAGCAAGATCAAGGCAAGAACCTTTTAATCCAATCACATTCCTTGCCTTGCCTTCGCCAGGTCGGAAGCCACATTCATCAAAGTTGTATACCAATCGTGGTGGTGTATCTTTAACCACATTGGCAAGCTGATTATACCAATTCGCTAGTAAACCAGCATCCTCAGCCTTGATACACTTTGATTCCTTCGTCTTTTGCTTCACAGGGCCCAGATTGAGGTGTTCTGGGAGTC

>DS499596:3627521-3627922

TGCGGCAGTTGCGAGCCTAGAACGCTACTGTGAAGCTAAGATCGAGGCGTGGAGTATTTTTCTACGACCTCTGTGTGAATCATACGGTCTTACCGACCGAGAGTGACGATCCGAGATCCGCGACCCGACCGGGCCTGCCGCGGCACACCAAAAAGTCTCCGTGAAACCCACAGTCGATAACTTGCAATCTGCCCGACTCCGAAAGGGTCATTGGTTGGCTACAAAACTCTAATACAAACAGGGTTAAAGAACTGGAAATGTGATTGGACAACAGCTCAAAGATTGGTCTGGGGCCCCGGGCTCGGGTGGACACGGATCCCCTCGCCCCGAGGTCCGCCGACACAGATTATGCCTTTTGGTCCTGTTGCAATTGTGGAGAGAAACTAAGTCAGGACGGAGTG

>DS499596:3717064-3717465

TAATCATCGCCCTGCCAGTTCTTCACTCCCACCACCTTCCACCGTGCAAATATTCGACCCAGGATGATGAGAACACCAATTCCGTATTCCGTCCACGCCTCCTTCTGAAACGGCGTTGCCTGTGCCATCTTGGGCGACTGGAGAATGAACAGCACGAACAGGATGTGGGACGCAAGGAATCGTACGATGTCCGATGCGATCGATATCGTAAATTAGCCGGCGTGGCGTGCCCCGGATGGCGGTCGCAACTCATGCACCTGGAAGTGTGACTGGCGCACTCCCATCGATGTTAGTGGATCGGTGTCAGGTAAAATACCTTCAAGCTTGGCCTTTACCTGTGCGTCGAGGTCCCTGTCAGGGGAAACCGAGCGACTGGATTTTGGGTTCGGGGAATCTCACTT

>DS499596:3718833-3719234

GGACAAAGACATGGCACAAGCCGTCGGTCTGCCCATGGGAATGATCGCCGGTTCTCCCCGCCAAGAGTAAGCCAGCAAGGATGATGCAGAAGTCGACACAGGCTATCTGGAGACCAATTTCGCTTCCAAAATGCTGTCCTTGTTGTTGCCAGACCAGTTCTGGCCCCATCTCTCTTGGCTGTCGACCCATTCATGGGAAAGACGATCGCCAATGTCTCACTGCTTACACGGAAAGATCCAATCAGAGGCCATGATATATGGCCATTTCCTCCCATCAGGCCTATAGAGTTCGGCCGTCACGACGGCCAGAAGACGGGCGATAGTTTTCCGGGTAACCAGCACCATTTTCGTCGACACGCGACGAACTTCACCGTCGACCCCGCTCCTCCCTGCTGCGACTT

>DS499596:3906717-3907118

AAGGATTGAAACACATGTCTTACTGTAATGTCCCGGAAGGGAAAGAGGTCTTCCTATTGGTCTGGAGGGTACAAAGCTGCAATGACAGCGCACATATCTTGGGCAATGCATGTCCGCTTGTCTCATTATCATGTCAACAACGACGAATCGCGACGAGCGACAGGGTCGAACAGCGTGCTCGGGTTTCAATCAGGGGTATTCCTGATCAGGCAGTCCAATGAGAATGGTGAGAATAGGTCTGCCAAGGCTGTTATCTTGCTATGGTTGCATCCTTCGATATATCGGGCCAGACGTCCGAGCATCTGACTGTGTCTGGTCAAAAGATGTCTTCAAGGGCAACTTAGTTTTCCAAGTTGAGGGCGAAATATTTCGCTGGAATACCATGCGAAAGACCTCCATTG

>DS499596:3914329-3914730

ATTTGTGGGATTTGCATCAATAATACGGAGTACGGTGTACAGGGTAAGAATACTCCGTAAAGCAAAAATCATGTTTGGCTGGCTTTTCCGAAGACCATTATTAAAGGAACCTTTTGACCAGTGAGACAAGACCAATCAGGTCAGATTCTGTTTCCTGGTCAGTTTTTGAGCTGCCAAGTGTCCGCTATTGCCCGACCCAGACTTTCTTGTTGCAGAGATTTCGTCGTCAATACATTGTATTGTATCATACTTACGATTTCAGATTTCAGATACTCACACGGTACTCCGTACTCTGTATCGTAATTTTCAGGTAGGCCTCAGCTCACAGGAAGCACTCTTATCATGCGATGCAGAGTAAATATCTGTGGCCTATGATTGAGTCTTAGCGAACGCAGTCACTG

>DS499596:4093283-4093684

ACAACGCCCTCAACGGGCACCCCCACGCTGCAGTGACTGCCATATTCTAGGCCATAGGCGATTGCAATGTCCGCAGCGCAAGAATAACTAGATTTAGTAATAAAATCATGTTTTAGGGGTTCAAAATAGCCTCCAATTTCGGCCGCGGCCAAATTCTATGGTATGGTGATCCGCTCGGTTGCGTGATCCGCTCGCTTACCAATTACATTACATGCTGCCTTGCTTACAAATCAGCCTGATTTAGTTAGAGGCGCTGGATCTGACCTAGTCGGGACCACTTCCGGTGCCCCTGGCCAACTCTGCCCTAAGTAATTCCACTCCACCGTACGGAGTATGGATAACATATGCCTGTCTTCTGTCAAGGGAGATTGGAGGCAAGGGCATAAGCCAACATGGCGAGA

>DS499597:94885-95286

CGTCAGATGGCGTGATCGAGCTACCCGGGGGGCTATTTATACACTAGTTTCTCCTCGTCAACTTTTCAGTCCCCTTGCCTAGAAGAATGTTGCTCCCCGCATCCTCCTGTAGCGTGGACTGGTCCATTCGTCATCGACCTCACTCGGATGCCGATGCTGATTGATTGTCCGTCCCCAAACAGTGGGGGGGGTCCGGTTTCTGTCCGAGAAGAAGTACTATTTGACAGGGCTTGCTTTCAAAAGCGGTCTCTTATTGGGCTGTCAAACTGTCGATCACTCGATGAGGAGCCATGCCATGACCAAAGCAGAATGCCATCTCACGCACCTAATGGGAAGTCTCATGATCCCACTCTCGGCCTTTCGGCCACTACGAAAACCGAGACGATGTGCTAGACAGGGAT

>DS499597:126329-126730

GCCTTGTGCCATTAATTGCCTGGGTGGATTTGCTCCCACGAACCGAGCTGTCTGATGTGAAACCAGGTGATGCGCAATAAGATGAAGGAAGTGGATTCTGCATCAATTGCCTGATCCTTCAGTCCAAAATGGTCTAGCATGGCAAGCGAAGCGACGAATTAACGCAGAGATATGGATTGAGGATATCAAGTAGACGGTATAAGTTGACATGACTGCTCCAGTCGAGTGTGACGAAGATTACGCCACTGAATCGCTGCAGTTGTCGAGGAGCTGCACTGCACCATGCCTAAACATTCGGACCTAGTCTGTCTATATCCTAGCGAGGGTTACACCAGCCGAATAAGCCCAAGGTCTGTGCCGTGTTGTTTCTTAGACAGGCACTAATCCTGGCGTCCCGCTAA

>DS499597:159496-159897

CCTTTCTCGCCATGCGGTCTCCACTGCTGAAATAGGCTTGATCTCCTCGGGGACCGCGTCAAACTCTGCTTATTTCACATGCGGCGGACGATGAGCATGGAATCTGGCGAGTCATGCCACTACGGCATCGTTCTATCCTTGGGAATAGTGAGCCAACCAGTTCGTAGGGTTTGGTCTCACGTTGAACTCCGTCCGAACCCTATACTGTTACTGCGTACATCTTCAGACATCGTTCCCCATCGGTTGTAAACAATGTGGCAGAGAGCGTTTGTAGACCACCCGATTACTTCTTGGCCGTTCCGAAGATCCACTCTCATCCACCAACAACGGCATGGAAGGACAAGACAGGAAACGATCTCCACGGGTGCCATGATTTGTCAAGGTCTGGATGCTGATTCTGT

>DS499597:423968-424369

ACCTATAAGAACAGGGATTCCTCGGGCACTTTGTCTCAAGAACACATAAGTCATTTCAATAAGGTATCTATCTACTTTGTACAATGTCGCGGAGTGTTGGGAAATGCCCTGTGGAGCTTCTCTAGTAACCCCTTACTTCATCCACATCACACATGCATACGGGGTAGATACATATGAGGGGACTTTTGAAGGATTCGGTTTAGCCATTTGCTGAGTTGGTATTTTGCAGGGGATGCCACGTCAGACTGCTCGCCACGGAAAGAACTCATATGACTGAGTACATAGCAAGGCTGAATTGACATAGTTGCGTTGCATGGTCGGTTGGAATCGGAACATATCATACATCAATCAAAGAGGCATGAAAATTCAATATATATAACCAATGCTCAGCTCATATCCGA

>DS499597:424974-425375

GGCTGCCGTCCCGTCCTGCCGTTTACATATCCATTCCTTTGGTCTATCATTCCTCTTTATGATAAGTTTAAGATGGGCCTTGTGGCGTTCTACTAGCTATCTAGTGGCGACCAGGAATGATGCAGGCTGCCTAAGATATCTACCTTGTCCATTGCCTGCGTCAGCGTCAACCGATTCCACCATCCGAGTCGAAACAGTAATAGACCAGAACAAAGCGCTACAGTAGACCACCGAATCTGACATCCCGCATCGATGACACGTTTGGTCAGCAGACTGAGCCATCTACGGGCATCCGGCCAGCGACTCCTGATGATCATATCGTCTCCTTTCCTTCGTTAGCCTTAGCCCCTGGTCCTCGGTGGCAGTGGCAGTGGCGACAGTGGCAATTGGGACAGGTCGGA

>DS499597:437409-437810

CGGGAGTTTCTCTATCATAATAACCTAGGTATTCCGTAATCTATTACCAGTCTTTCCGAAGAGCTGGTAGCAACTGCACGAGATTTGTAGGAGCGAGTACCCGGCTGGACGAGCACGCAGCACGGCTATTGGTCAGCATGGTAGCTACCGAGGGGAGGCAGGCCGCCCAAATATCGTGAGTCTCCTGCTTTGCCCGGTGTATGAAACCGGAAAAGCTGCTATAGAGCTTCTGGGCGGCGCATGTCGGGAAACCAGCAGCAAGCTGACCCAGAAAGACCCGTCCTCAAGCCATTACCGTACTAATCAATTATTTGTGTAGCAACACTGGGAAGCTGTAGTGCATAGGCTGGAGCAGCTATTTGGCCTTTAGCCCCGTCTGTCCGCCCGGTGTGCGGTTTCGA

>DS499597:679756-680157

CAGATTGCTACTGGTCGAGAATCCACATCCCTGATATGCGTTGAGAGGGAAAAATGCCTGGTTCGGCTATTCCTATTTGTGATTCGACTCCACGACGACTGGGAAACTCGAGGCGTTCAGTTCAGTTCCCACTTGCCTGAAGCCCCGATCGTACCCGTGTCTGGCTGGGAACCAGTGGGCCAGTCTCCCCACGACTGCGAACAACTGATGTTCGGGTCCACATTCGCGTCGGTGGTTCTATCGCGAGTGTACGGAGGAGTATCCAATATGAGTATTGCAGTGAAGCAATCCTCCATCCACTTCCTTCGCTTTCTACAGCTAATCTACATTCTCTACGGAGTATTACTGAATTCTTCTCCAGGGATGGTGTCCGCTATCGGCGATTATGTAGTGGGACTGTC

>DS499597:705782-706183

GATAAGGATGGGGCACAGCATCGTTAGCGGCTCATAGACAATGCAACCTCCGTATCGAGCCAATCGGGTCTCGGCTGGACGCAACGGCCTTGGGGTATATTTGTTTTTCTTATCAGCTGATGACAGGGGTGTGCTCTCAACAGGCAACCCGGTGGTGTTCCAAAATTCGCCAACGATTTTCCGTGCAACAATGAGTGCACGCTTGGGAAACTGGAGCGATGATTCAAGTTGTGCTTTGTTCACAACATCCAGTCCAGTCCATTGGCTTCCTCTTCTACCGGGAACACTTCTCGCGCATATTCCTCCATGTCTCTCCAGCCCTCCCACAGCTGTCGAGCCTCGTCGGTGACTCGAAGTGAGACGACCCCTCCGGGACCCTGCATCATTCCCGCCCCCGCCCC

>DS499597:708677-709078

GAGTCTCTTACCTATCATTCAAATCAAAGAATCCACATGGTTCGATTGATTAATGAATCTGGACCCAAGTCGGAATGACACCAGTGACAACGCACCAGATGGAAGGTGGGCAGCAGGACTAGACAGGAAATTCAAGTCGATGGTGCATGATCGCAAGAAGCGCACTTTGAAGGCAGAATTCACCAGAATCTTCATTGTTACTGATACGTCTGAAATGCTGGCTGGAATCAAGGCAGGGGTGGTACGTTGGACCATACACGTCCTTTTAATTTACGTAACTCGGGATGCACTGCGATGCTCAGGGATGATGGCAGAGAGGGGTCTCCATAACTGACTTCACTGGTTGCGTAGATTAGGGCTTGCTGTTAAATACAATATTAATAGGTTTTTTTACGCAGTAC

>DS499597:709747-710148

ATCCTACGATATCATGGTCAAGTGACAACTCCAGAGCCTACGGCAAATCGTTCCGGCTCCTTTCCGGACCCCAGCATTGAAGCTGATTGTTCCTCACCATCGCTGCTGGAGCTGGCTGCCAGCTGGAATCAAACAACGCGAGCATCAAACCCCATACAAGCAAATGACACAATCTTCATCAATTCTGGCTCCTCATCCCGTCATCGGCAATTGGTGCATCCCCCGAATTCATCATCGCCCATCCGGATCGCACCACGAAGAAATCAATTGATCGGTCAATCACGAAATCACCTCAAGTAATACCATTATCACCGCCGATAACATCGTAGAAGTAGAACGGGCCTAGTTCGCATCTAGAAAAATCCTCATCAACCACATGACAAGCTTGATCACCGGTTCTC

>DS499597:762754-763155

TGCCGTCCAGTAGGCGACCAGAATGTCAGATATCGTACGATCCATTTCAACTTGAAACATATGATATGGTGCCAGATGGCTTCAGCAAACAACCGCCACGAGGTGCAGCAGTATCAGTGACGTCTCAGCATCTCTGGAACCAAGTCCAGTTTGGAATGGCGCAATCGCGCAGTAGCTATCTCGTACAAGCTGAAAGTCCGCCCACTGGAAACTTGACCGGTGGCGGGATCTGACACGTCATCATGCACCCTCATTATTGGCCCATTTGCCATGTATGCAACACGGCGCATGGTCGAGGCCTACCGGACCACACTCGTGGGATGAATAGCCGTGAAGAGACTTTCCCCTTGCGAAGGCTAACGAGAAGCATGCCGATGCCGATATGCAGCATGCACCATGCG

>DS499597:824281-824682

ATGAGTCAGGTTCAACAAATTCATCAGCAACAATAGTGGACCAAATGGACACTGCCAACATATGCTGGTTTCGGAAAACGAAATCCCGCCATCAGCCCTAGCCACGAGTCGCTGAGAATTGCTTGAATGGCTCCATGGACTCCGTGGCCCTAAGAGTGTGACCACTCGGCCCCCTTCTTCCGATCATCTGCCAACGGATGTGTTGAATATGGATGATCTGATCTGACTCTTGAGTTCGCGAGAAACTTTTTCCTTCCCTTTCATGTTCACGCCACTCTTTCTCCCCTGCCTTCATCACTCAAAGTGGGTCATTGTTGACGGAATCCCCGCGGTCTCTCTCTCCACCAGGGTTGCCTGGATGTTGTAAGCACCCTGAGAGGCCCAGAGTAATTTATATTTAG

>DS499597:866102-866503

TCCAAAGGATACAGAACCAAAAGGGTAAGGTACGAATAATAATTACAGCTGGGAAATGATACCAACTGTTACCTTACTGTATCATAGGTACCTACAATATGCCGCAAACGGACGACAAGGTAACCCACCTAGAACGGATGGGTCGTTGCAGGTCCTGAGGTACCAATTCAAATGATTGGCAAGCCCTCCTACGTTGACCAATGGAGTCTAATCACTGGCTAGGAGTTATCAAGTTATCAGGTGCTCTTCGCATGAAGAGTGGGCTGTGGATTAAGCCACTTTCTGCGGTTAAACTCGTAGTCTATATTAATAACATGCACACATTGGCCCAGGTCAATAAAGGACACTGGGTACCTATAAAACATGCATACCTGCCGTAATGGACCTACATACCCGGTACC

>DS499597:927643-928044

ACTACGAGGGAAGGAATTCTTCGATAACCTCCGCGAAAGTCGGAGACGTTTCCCACTCGGTCGCCTTGCAGATCGTCTAGAGGCAAGATGATTCTGGTCCGGTCGCAGATCGTCCCTTCTTGACTCGACGGATGACTGGAGATGGCCACGAAAGCGCATCGGCGTCAAATGAGAGGAGTTTTTACTGGCTGACTCTGGAGTGACTAGGTACCTCGTCTATTGTAGTTTCCTATGTAAGCACCTTTCCGCAGTACCTAGACCAAGGGTGTCGTATCCTGTCATCGTGCCACAGACCGATGGGGAGCGTTGATGCTTGAAGGGAACGGAGTACGGAGTACACAGGAGACAGACCTGGATAGTTGATGTGTTGGCACTGCATGCATGGCGGGTTGTCCTTTTTC

>DS499597:935627-936028

AGAGGGACCATGACGTCGTATACTCCACCATCTGGAGATATGGCCGGAAGCTCGGAAGAAAGAAAGATCCTTTGCTGTAGACTTGGATCCTGAGGTCCAAAAATTGCTACTGAGAGAAGCGAGAGAGCGTATAGAGCATGGCTTTAGTCAGGCACAGGAAGGTAGTTACATTTCCCTATCTAACTTTGCCCTGACTATCACTTGCAGTAATGGCAACAGATCGTCCAGCATTTGTATAAGTTTCTCCGAGTGGTGAAAGAGTCAAATGACGCGATAATTAGGTAGGTAGCCTACACTGATAACGCTCAGTCCATCGAATCATCCCACCAGGGCTCCTATAGGTTACATTCAGCTGTTGTAACATTTCTCAGAGCCGCTCGAATGGGGCAATCGCCACCGGT

>DS499597:951285-951686

CGGAGAGTACGGAGTACTCCGTAGAGAAAGCCAAAATAGGACCGTACGATCCCAATTGTGTGCGGTGGGCCGATGATCTATCTATCCATGGGTTTTATGGGGGAGGTCTATGACCTCGCTCGTATAGTTCAGCCAGTTGAGCGCCACCATCCAGGACAGTTCTCGCTGCCAGGAAAAATCCATCCAACCAGAGGGTCTCTTCGTTATTCTGACTTTCTCTTTTTCGCGCCGAATCCTACTTTTCCCTGTTGTTGCCGAGGTTTCCGTTCTTGCGCCATTTTTATCAATCAGATAATCGGGGGGCCTTTTCCCCTCGTGACTATACGCATTTTTTATCAATCAATCAATCAGGCCATATGCTGTGGCCACACAATTCCTGCGTTCTGCTTAGATAATATTAT

>DS499597:1036136-1036537

AGATGTGGTCGCAGCGGATCGGGGGGACGTTCGAAAACGGAATTAAGAGAGAGCCAAGATAGAGATGACAAGAGGTTGCGGATGAAGCTTCTAGATCCCCTTCATACCATCAGCAGCGTGATAGACAGGGGCTAAGAGAAGCTCCAGCTCGTCAGAAGGCACTGGAACCTTGTTGGAAGCTCGACCAGTGGAACAGCGGAGAGCATGGAAGTTGCTGGATTGCCGTAGAAACACCGCATCGAAATTTTGTCTCCCGCAGCCCAGAAAACATTAAGTCGGCCTTCCCCCCCCTTCAGCTATTTTACTTCTTTCTCCTCAGTCATATCTTGTTTCTCATCCTCAAAAGTCTCACTCTTAAACCTATTTCCTCTCTCCCCTTTGACGCCTTCGCTTCATCTCTC

>DS499597:1059506-1059907

TGCTCTTTTGCGGCCCTTTTTTCCATCTGACCGTATTGATACAGCCGGTACATGATTGTGACCGTAACTCCCGGCAGGGAAGCCAGGCAATGATGGGCAATGGTGTGCATTCCGTACATTACGGTAACCCCCAACCCAAGGGAACATGGAGCCGGACTCCCGAGTGCCAATCCACCCTTTTTTTTTTCGGCGAGCTTCCTTACAATGACGGGGATTACCGTATTTGCGCCAACCAAACGTTGCGGTTGGTCTCTCTGAAACTCCACCCGCCAAATTCCGTCGCTCGGGAAAAGAGAGACGGGAGTACGGAGTACCTACTTTAATAAACTAGTGCAAAGGACCAATAGTTAGGTACGACGCGCCAGACGTAATCAATATAGCAAACCATATACCATCTACGG

>DS499597:1223502-1223903

GGGAACCAACTCACAAATCCTGCGAATCCAACTCGCTGGAATCATCAATCAACTGCCACAACAGAGTTCATTTTTGTCGATGGCCTGGATCCCTGTCCACCCGATCCAGAAGTCAAGTCTCTATGGCCCGGGGGTCCGTATCTTTGCTATTACACTGACCCAAGTCACCCAACCCCCGAACAGTGGGCACGTGGCGGCAAGGAGGAGCATGATCTCGCCATACTCCAGCGGTATGATCCTCGCACGATGTCGCTGCGGATTAACATCCCCATACTGCATGTTGCTGGAAAGAGAGATCCTTTCTACCAGCAGAGCGATTTGTTGGCTGGCCTCTGCTCGGCGAATGCTTCCTCTGTCATACATGATGGCGGTCATTTTGTACCCAAATATACGCACACCAC

>DS499597:1308896-1309297

CAAGCATAGTAAATATCATCCAGCATGCTTTTCCTTCCCTTTTGGAAAGTCAGCAGGATGACGCTAGTTCCTCATTCTCATTCAGCTTCATGCTTGGTGCCAGATTGCTGATTATCGCTTGATCTCACAAGAATTGGTAATATTGCTCCGAGTGACGTTGTGTTTACCGGAAAGAGGCAATAACAGGCAACAAAATTGAGGCTGTGCATTCTCCCTGCTCATGCATACTGTATGTGATCTGATCATACTTTAATATTATTATTAATTACCCCCGCACGTCAGTACCACGACCCGGTGCTTGAGTGGCTTTTCTCTAGGACAATTGGATCAGTAGATGCTCTCAAGTGTCAATTAGCATCATCAGCTAGCAAGCTGTGGCGGGTAGTCCTCCCCTCCTGGAC

>DS499597:1310194-1310595

ATTTTTTACGCATTATTGTTATACTCCTAAGCTGAAACAACCAACCCAAAGACGGCTCGAACAAGAGCCAGTTCCCGCTTATACCCTCGAGCCTAAGCAATCGTCAAGCTCTGGCAAGATAGACCCCACGCCATGCTGCAATCATCTGCACTTTTTCACTTCCCACATCTAGCAGCTACCAAAGACATGCTGTCTTCTTGGCGCATCATCAAGCCGTAAGCACATTGAAATGGTGGCCGTTCAGTAGGTGCAGGATGAAACGAGGGATTTTCAGGGCCAATCAGAACAAGTGTGATAAACTAGTCTGATCGCACCAATTAGACACCAGTTTACGGAGCATATTATTGTCGATTGAAAGCAATGTATTGCGCAACGCTGAAGGGAGCCGGTGATGAGGGGCA

>DS499597:1400801-1401202

GGAACCAGGTGCTCCATTGTTAGATCCAAGTCAGATAATGACGTCGTTGAGTTCGTCTATGGAAGGCCAGTCATCTCATCTCATGAATGGCTTATACACAAACAGGATGAGCAGGCAAGATGAGGCGACAAGTGAATGGGCCCACTGAGATGCACGGTGAGCTGCTTATTTCCCATGCTGTCCAACAGTTCGTAACGTGATGGGGCAGCAGAGACGTCAGAGACTTCTTCATCTGGAGAATGGTTCTGAAGTTCCAGCGTGGAGACTGCTTGGATCAGACCATATCTACGTATCCTCGGGAACCGGCCGGTGGATGACGGCACGATCTCATCACATTAAGAGTTCGTCGTTTCTCCAGATTGCTGCAAGTGGGCATTTTCAGCAGCCAGGCGAATGCAAGA

>DS499597:1409319-1409720

CCGAAGACGGAATGGAATTGCGTGAGGAGACTTCCTCGGCCCACCATGGAGGATTCCGAGCATATGCCGTCGGGCGTTCCGCATACCGTACGATACTTTATTTTACCACCTTACCCACCCTTTCCCCAGTATCGGATCATACGGCTACGGTAATTCCGGCGAGTCTTCAAATCTTCATTACCAGCCAATCACATGCTGTGTATTAGCAGCCACCTGTAGATGTATATTGCCCCAACAGGCTTAGCGCAAAAGTCTCCCCCTCCCGCGGTGCCGCACAGTTCTCCCCATCGGTGACTGAGGCACTGAGGCAATGAGGCGATGAGGCAACGGCCGAGTCACACTTTGGCTTTTAGTCATCCTCCGGGTTCTTTTTCTCGGTTTCTGCGTCTGTTCTGCAGCCA

>DS499597:1479669-1480070

AGAGTATCAAAACACCTCGCTAGAAAGGAATTCAATGAACGACTTGATGCCACCACCCTCAACGATCAAGTAGAGGCAAGCATCAAGCTCCTATTGCCCAATTGGGATTGAGTAAAGGTTGCCAGATACGTAAGGAAGCATAGAAAGTGATGGAAAGTCATAGAATCATAAGAGTGCCGTCAGCCAACAGATAAAAGAGCTCTATTTGGTAGTGGCAGGGGAAACAGACCACAAGAAGGTGGTGTGGTGTTTGTATGGTTGATCCTGATGGAGCTTATCGCGTGATGCCAGCCAGGGCGGTTGATCTAGATAATGTCCGCAATTGGAATTGTGGTGATCGGGGAAGCAACCTGAAGCAGCTACCAAGTTGGACTGATAAGTCTCCTCAGCAACTCAGAGGC

>DS499597:1480982-1481383

CCCTGTAATGACGTTTTCCATGCTCTGAAAATGCTGGGGCCAGTTACCTTAGTCTTGACTGCCCCAGGCTTTCTCAGGTCTCAGACACTCGAGCCAAGTGATTATTGGTCACTGCGCTGTCCGATTTGGTGTGGCGCCATGTAGCCTGAATTTAGTAACGATTCGGCGGCTCCCTATAGGTTCAGCTTGCAGTAGCGGCAGTGCATGTAGCCGAAAATGGAGTAGTTTTACCGCCACCGTTGCTCCGCCATCGGCGAGGGTATTTGCTGGGGTTGGCCCAGTATGCATCATTGTCTTCCAATCTAGTTGAGTGGTTCACCACCGAAGGTCTCGCCCAGTATGGACTCCTACATCATGTTTATCCATACTCAGCAGCCCTTAATGGCGAAAGCCATTCATTG

>DS499597:1482126-1482527

ATTGAGATTATACTGTTGATGACGAGGATGTGAATTTGCTGTAGTTATCTGCCCATAGGATTGAATCATTCAGGGGGGGGGGGGGGGTGGTGTGGGCCCCTTTTCCGAAAACAGCCTCGACCTGCGCCCATCTGCCAGCAATCAGGAAGGGTGGGCCGACCAGAGATCACCCCTTCGCGCGTAATGTCGTTACCTCGTTACCTAAGATACTTTACCTACCAGTTACCACGAGGGCGACGTACTGGTATGGAGAGTATGACAGCACATTGGCTCAAACGGAATCCACTGATGGATCCACCTCGATATCGCCCAGTCACAGGACGTTTCGCGCTCATTCGGCCGCTTTCTGCCCATCACTGGCCTCGTTACCTTACTTCCTGCTCCCCAAAGGTTCCGGTGCC

>DS499597:1800375-1800776

ACTCTGACTCCTCCTCGGGAAGTTTGGCTCAGCTACGAGTCCACCGGAATCGAGGAGGTTCTTGCAATACCTGACAGACACTATTTCTGTCCTCGTGCGTGACAAGTAAATGCAACTGGTCACTTGATCAGTACACAAGAATGGGCAACGGGACGAGGAGAGAAGAGAGAGAGCGGCCAGCAGACGGCGGCTTATCCCGCTTCCGAAGCATCCAGAGAATGGTCCTCATCCTCTGTCAGATGACCCAATTCTACGGAGTACACAGGATTCCAGACGAGCACAAACGAGCAAGATTTTCCACTGGGCCGGGATCTGAGATAGATTGGTTTCATGATGCGAGGCGGAGGCCACTTACTAGGGACTAACTGGTTTTGCTTACTCGACCATCTTAGTCTGCTGGT

>DS499597:1841965-1842366

AGTGGTTGCAACTGCAACGCCTGGCCGAGCCTTTATTGAATCATGTGATAGGCTCCTTAGCCACTGTATATATACTATTGACACTCTGTACTTTACTCTCCTGTGCGGTTTGTGACTGCCATTATTTCTCATCGGAAATAGAATAGAGATGGTGAGATTGGATGCAAATATCACGGATATGGCCGAAACCCCTTGGCTCTCCTTTTGCAGGCAACAACGCCGAACGGTATCCAATAATAAACACTGTAATTGATCGGATGGGATCAGACCAGAAGATGCTCTTTTCCGGTCCTTTCAGTGATCATACGGTGTTCCTGAATTTTCTCTCACCAATAAGAATCTAGCACTGCGGAAGGAGGAAAGCCAGTCTTTCACATGTCACGCCACTGTCCACTCTTGGC

>DS499597:1929189-1929590

TTGCCAGATAGGAATTGACTCCCTTTGCTGATAATGAAAGGAATTTCCGGTGGCGACAACGAAAGACCATCCTGATGGTTGTTGGACGACCAAAGTCAGGTGACTCTTCTACGCTTCTGGTCGTTACGCCATACCGGACCAACGCCGCAGTCCTGCTCCCATTACTTGTCCCTCGCCACGTGAGCGCTTCTCTCTACTACTCGACTCTACTCCTCCAATATCCCTTCTTTCTATTGTAAAAGCCGCCACTTCTTTCTGTTTGTTTTGTATTCCGGTCCGTCCGTTGTCTTTACATTGGACGTTTTCATATTGAGTCTTTCCTCGCGATCAAATTCGATTATTGGTCTTCGCGATCGAACTACGATATACCTACCTTCTGTTGTTCCCCGTGGCAGGTGACT

>DS499597:1976790-1977191

AAAAAAAGGAGAAAAGAGAAAAACATCATGCCACTATCCAGCATCCAATCAGTCCAACCTTTGCCGTCATTGTATCTCCATCAAAACGTGGACTATCAAGGATCTGCCTCACTTTCTACAGCTTTGCTCGGACCGACCGACGTCAGGGTGGGAAAATCCTGCCGAAAATTTTGACTCTCCCTGCCATTTCGAGGCCCTATCCCGAGGGTCTAGAAGGGAAAAGAGAAAACTCAATTGGTTATGGGTCTTTTGGTTCATCGGGATCTTCCACTGCTCGGCTCTCCGAAATTGGGTGGCAAGACGGGGACACTCGGGATCAGTCGCCGCCGTTTGAGAGTGATCTAGACTTATAAATATAAAACCCCTTGGGGCTTGCTTTTTTCTCACTGTGGAATATACCT

>DS499597:2128697-2129098

GTTGTTCTGTATCTACTACCTGCTCTGGAGGGATAGTTGGGATAGCTTCAGCAGCCTGATCCCTGCGTTGAATGATCTCCTGCCCTTCCTGGATAGATAATCCCACTGCAGTTTCTATCTGCCGCCTAGATCGCTTCTGCTTTTGAAGGTGCTTTTCATGTGCAGCACGTAGATCATGATTTTCCTTTGCAAGAAGGGCAGCGTTATTCATTGCCATCTCACACCCCTTGATAATCTGACCTAGCACAGCCTTTGTAGGGGTAGGAGGGCTGTATGTACGCTCCCTAAGTAGCTTCTTAAGCGTAGTTTCCTGCTTCTTCAGCTGCTTGAGATTGTAAGGTGTTTTTGGGACAGAATTAGTTGATCGGCTGCCTGGTGGTGTAGGTGTTCTAAGCTGGATA

>DS499597:2134885-2135286

CTGTGGCTGCTAGTTTACTCTGTAGATACCAGCACAAATTGAGGACGGGAGTGGAGGTCGATCACCGAGATAGACAGGCTAAGAGCTAGTGAGCGATTTTCGGTTCTGTACGGAGTATGGCTGTTTTGATCGCGAAGACTCACGTCCTCAATACCTATGATCATGGGTGCTTTGCTCGGGAGTCTAAAGTACGGGAGCACCATGTACCCACCTAGGTACAAATTGATACCAAGCCGCTAAATATTCAGATGACGACAGGCCTCAAAGAAGATCCCCCAATGAAATGTCAGGAGCCAAACTCGAGTGGAGCGAAGGGTGTTGAGTGCGACTATTACTGATGGATTTACTGCCCAATCTCGACCTTCACTAGACTGAGGCCAGCTGTCATGATAATTGCATTG

>DS499597:2140693-2141094

GGATCCATCTATGGGTCTTTAGACCATCCACAGATAGGCTTTGTTAACGAGTCTTATGTCGTTTCTTGGACGGATAGGAACCAAATCTTGAGTGTGATGGCTCCGCCGCTCCGGCTGTGACGGCCTATGGCAGGACAATCGACTTGACCTGCCGCTGTTTATTTTTTGACCCCCTTTGAACAGCGCTTCTTTGTGCACTTGAAGCGTCATAATGCACAATGCAGGAGGGGTTATTACTGAAAAGCAACAGGATTCAGGAGTCTGCAAATGGACTCAATGGGCAATGATTGACCAGCCACTTTGTGCTTAGGACTTGGGGTTGCACAGTGGTTTGCGATAGTTGTATCCGCTTGGAGGTGAATCATCCGAATGGGCGCCGTTAGGCTGTTTCGAGTTTATTT

>DS499597:2163506-2163907

TGGCTGGTGCGTAGTAGTCGAGTAGCCATGAAACCAGAGCAATCACCTTCAAGATCAGAAGCACTAGGGTGGAGCGCCTTAGATCAGGGCTGTTGGTGGTCTTGAGAGGGGAAAGACCACGGCTTCTCCGTGTGATGAGCGGCGGCAAGTGAGATGGGGACGGCCAATGAAACCCCAGCTCCAATGACGAAGCTCCACGATTATCGTCAGCATCAATTGCAGATGGGCCAGAGCGAAGGAAAATGGAAACTACTAGAACACTGTAACTAGTGAGGAACAGTATCTGCTCCATGTTCGCAGTTGATTCTCGCCATCATACTGGCAGAGACAGCCCAATCCTGCTCATTGATTACTCGAGTGGGTGAGGAGAAGAACCGCGCATAAGCCACCACCCAAGGTCG

>DS499597:2207825-2208226

TGTGGGCATTTGGCACATTGGCAAGGGAAAAACAAGGGCTGGTGGGTTGTGCTTGTGCTCCTTCAATTCGACCAAGGGCGCGGTTGGCTCATTTGGGCTGTTGGGCCGGCGTATTGGGCTGCCTGGCCTTGTCTGGTGCCACGACGACTCCACCTTGTCATTGGTGAGAATTCCGAAAATAAATAATGATCGAGGCGAAAAGAAAGGTGAAAAAAGGATAAACAAATGATGATTACGAACTGACCTACCTACGGAGTAAGCATAATACTTACTAACTAGTTACTGTACGAGACCACCTTACCTCAGTTCCTAAGTCAAGGTACCTACCTACCTACCGAGAGAGGTTTCACATCCGATGACTGTGTTGAGGACTCCCCAAAACCTGCATGGTGACCAATACG

>DS499597:2208899-2209300

GAGCAGTCGAAGGAGAGGCGTTGGTGATTGGCTGTTGTCGTAGCGGGCAGGTAATCGGCAGAGTGCAGAGAGTTGGGCAGAAATGCGTGGCGGGCTAGAGGGCTCTTTGGGCATCCCAACCCGCTCATTTGTCGATCCAGTGTCTGGACCCAAGGCTTCAAGTGCGCCAATCTAAGGTAACAATTGTACCCGCACCGGGCCGCTAATCTACCTCTGGTTGTGCTGGGCGCTTTTCTGCCGTTCTTCCATGGATGAATTTCGTCATGTGAGGGAGCGGTACTTATGAGATACTGGGGGCCCCAGTGACAAACCGCCTCACTATGACCGGTGCATGCGAGGTACTCTTAAAAGTTTGAGACCCCTGCCTAACAAGGCCTCTGGTGGTGATCATGATCTCCTGT

>DS499597:2216502-2216903

TGCCTGTTGTCTAAGGCCTGCAGCGAAACGGATGAATATGAAAGCCAAACACAACAAATCCAAGCGGAATAGCTTATTCAGAGTCCACAACTTCGATGTGTAATTCTGAAGAGTAGTGCACTCCAAATTTAGGGCCAAACACGACATCGAAATTACCCATGACATAAAAGGAGAAGGGAAGACAACGCCACAGAGGTCAAAAACTTGAGTTTCCACAATGGAAATATATCGGCGCAACGGGAATGGCTGCACTGCCACCAAGGCATTAAGTTAGACTAGTGGTCAAGTGCCAACTACAGATGCTACTTATTTTAGTTCTGGCTCAATCCGTTCGCTTCGGCCTGTTGTTGTTCTGTGTTTGATGGAGTTTGGTTTGACTCCCGTTGGAACAATCTGTATAA

>DS499597:2218924-2219325

CCGCATATCAAATGGTATGTAACGATTTGGAATCAGTATCATCTGCAGACTGTTGGTCCCACAAAATCCAATGAGAGTTTTCACATCGGAGTCCGTGCTCCTTTCTTGGGCGCTGAGTTGCTTGGAAATCGACGGGATGACACGGTAGATCCATTCCCAGTACCATCTGTAAGGTAAATTTTGTAATCAGGTACGAATTATGTATGTAAAAGCAATATGAATAGTGAAATTGCATTTCACAATTTTCAGCCTACATTGCAGAATGCCAAGTAAAATTATTCGTTCAGGGCTCGTACACGTACGGAGCACCTTACGTATGCCTGATTTCTCAAGCTTGTGGTTGGTGAGTGAAATGACGCGGCCGATGGATAAGGTATGTCTCTTAATTGTCCACAGTGCCA

>DS499597:2300473-2300874

GCGATTACAGAGAAAGCAGACGATAGGTAGAGAGAACGTACGCTGGTTGGTCAATTACCATAGACAGGCGCACGTGCAGCATGCGACGAGTTCATTTGTGCATCCACTCCGGCTTGCATTTTGCATCAGTTTCAGATGATCTGGTTGGCTGGTCGGAAGTTACTATTACTTGGATGAACAAGTCCCTCGAATGAGGAGCTTATTGATCCATCACATTTCTAGCTGGCTTTCTAGCTCTGTTTTCCCAGATGTTCTCGTCAGAGTGGTTTTTATCCGTTGGATCCGTTGGACCCGTTGGACCCGTTGGGTTGGCGCTTCAGGAACCACGTCCGAGAACCTATTGGAGTCTCACCTGTTCAATCACCGTAGACATGTGACTGCGTTATACGTGGTCCGGGCAC

>DS499597:2302551-2302952

GACTATTCCATGCCACTGCTGTTATTATGACTTTTCGGCCGGTAGTGAGTTCTTCACTGCCCATGTAGTAATGGATCAGAGTCATCCATTCGACCGTGGATCAACTGGTATATGGGTCTTTCGCTCAGCCGTACTTCAGATGAGCCATAAAATCCACACTACTGTAATTAGCACTTCAGAATCGAATGATTCGCCTGATCGACTCAGCTAGTGATTGCTAGCATCGATTTGCCGAATAGCCGAGTAAAGTGGTGAGACCGCCAAGATCTCTTGTCAAGGCTTGTCTGTCGCCCTGTGGTTATTCGGACCGGTCGTACAGGTTGTACAGTCGTATGAGAATGTGCTTCTGGACCCCTGACAACTTTGCTATCCTCCAATGACCATGTACAGTGCATCAGCAT

>DS499597:2499469-2499870

CCCACTGGCCCCTGGGCAAACGAGCCAATGAGATCAGGACTTAGTGGGGCTCAATCATCACATGACTTCTAGTATTTGCATGTGATAATTGAATCTGTCAACAGAAATCCGTGTCGGGCGGTTGTGTTGGTGCGCCTCCAGGGGGTATCCATGCACATCCAATCAGGCAAATTGCCAACTGCCGTCATTCGGATCATCCGCAGCGGTCTCACGAGCAGTTGCCCTTTCGGTCTCTTCCCAGGTCCCCCTCTGGACTCCGTGCCTCCAAGATGATATCATGACACAACTTGATAAGGGATAAGGGCCCAGATAAGATGTGGCTGATGTCTCCAGCTTCCCCGCTGATTGGGTCTAGTGGGGAGTCAACAGTTCCCAGTAGATCCAGCCGATATTCTCAGAGA

>DS499597:2624726-2625127

GGCAGAGGAGTTGCGAATCCGGGCAAACATATTGCGGGCTTCAAGAAGGGCTTCGTCAACCTGGCCACTCTTGGCCAGGGCTACTGCGTACATAACCGTCGGCTGAACCATATCCGGAGTTGCCTGACCCACGGTGCTTAGCATCAGCCACAGGGGTCGAGCCTCTGACACATTGCCTTGCCGCACATGCAACGCCAGCATCGCAATGGCGGGGCCCTGACGCACAATCGGGTCTGAGGGGAGGCGTCCGTACGCTTCGGAGGCGGTGTTGAGATCACCGGCATCCGCCGCAGTGACGCAAATGCGGGCCATGGCATGATCCAAAGCATCACCGCGTAGCTGCTCCTTGGCGTGCTTCAATGCCTTTGCATGTTCACCTGACTTGAGACAGTACTGCACGA

>DS499597:2627301-2627702

GATCAGGTGGTGGCTTTCGTTCCCGGGTCAAGGGACCCATTGTCCAATCGAAGAACCGAGAGAGTGAATTCTCGCTGGGGCTGGAGGGAAAAACAGGAGGTATTTTTCACCCGTCCCTGATTCTTTGGTGGATTGCCGGAATTGTCTGGCTGTTTTGACAGGGGCGCAGCAGCCGTGACTCACCCGTCATTCCGACTTATTTTCTTGAAGCGCTTCGTTGGCTCCGTCGGGGATGCCATAACGCCGAGCGCCGATTAATGATAGTCCGTCCAAGGTCATTTAATTCCTTTAAGAAAATGACAAGTCGGTGCCTGTAAAGCATGCTTGTAGTCTCTACAGGATCGTTTTTTTTTTTTTTTACATGGATTATATGGTATATGATAATGCAACTCAAGGTGGCG

>DS499597:2703231-2703632

AGACCACTTGATCTGCACTCTCGCCTTGGGCTTGAGACAAGAAAAAAAAACAATATGGGGTGTCAGCAACAGTGATAGCCTGACATTTTCAAGTTCAAGACTAGTCTACGGTACCATTTACCCAAAACGGACCTCTTACACGACGCCGCGTCGCCGGGAGGGACTCCCCCCAGCCCTTAGGATCTGGTAATCCTTTAGGTACTTTCTGGTAATTATGATAGATTACCTTTCGCTCCTTACTTTGTATTAGCGTCGGTAGTGGCCGGTTCGTTACTCCAATCAGGATACGGAGATGGCAAATGACATGGATGAGGGGGTGCTCATTACGGTATTCCGCATGATTTTGGGGCGGTTACTGCGTTGTCATTCGTGCAAGACTATTAACAGGGGTGGGAGACGGG

>DS499597:2705873-2706274

CTTAGGTAGAGGCAGAGGCTGAACAAACGACCGTCAAGGACTCCAGGTACAAAACACAGTTTGATAGATGCCAATAGGTACCCCGGGCAGGTGACCAGTACTTTGTCATTCCCCGAGAGTGAAGCCCGACACTTGGCGTGGGTCGATGCCTGCAGGAAGCCGCATTTGTTGTTATCTTACAAGTTACGCTACAGCAGGATTACAGGGATATACATTACGTTACCGCCGCATGAGCGACTGCCATTCTCCGAGGACTGTCCTACAGAATCGACAGCACAATAAATCTACTCCGTAGGACATGGGCTAAGAATAAGTGCCCGTACCCGATGGAAAATCCCCTGCTAGTCTTCCTTGGCGGGCTGTTCTGACGTCTAAGATTATTAGGGTTGCCCTTCAGTGAC

>DS499597:2760796-2761197

CCAAGTTTGGCCGGAGGCTCGAGCTTCAAACTAAACGGCTGGTTCTGTAGGCTTGCTGTTGCTTGCTTTAATCCCGGGATGTAATGATTTTCAATCAGTCCAATGTCAACCATTACATCTTCTAAACTATAGCTGGACTAGTTCCAACGGCTTTGGAGCTTATCCCAGAACACAGGATGCGACGAGCCAGGCTAGCCGACTTGCGAGGATCATACCATCTTTCATCTCAGACCCAGCAGATCCGGCGAGCTCGCATAGCCACATGGGCAAACGCAAATGCCGGCGTTGTGATAGATAACAAAAGGGAATCTGACAGATAGAACTACTCCGTACCTCTACCACAGTCGATCCATATTCAGCATATCATTCAAAAGGGGTCCAATTGTGCTTCCTTACCTCGC

>DS499597:2765248-2765649

GCCCCAATTGATGACGACTTAAAGCACCAGGTTGGGGCCTCGTCCGAGGTGGAGCTTGGATGGATTAGGCACTCTTCCCGTTCTGGAATTTTGCTGGAACCCACAGGCGTCTCGCCTGGTGTGTCTTGATGTCATTATACTTTGTAGGGAGTGTTTCAGGCTGCAGTGCGATTCCGTCACTTGTGGCTGTCAAATGGCCGTGTGGTATGACAGTTCTGGACATCATTCCACCGATTCCTCTAACAATCCATTCCAGTGCAATGTAGTCCGCGTCGTGGCGAGATATGGGAAATTTTAGCTGCCTGGAAACCGTTGTCCCGGGTCACCTTACGCACCTCTCTACATGGTATTGGTAAACGGCTATATGTCCGTCCAGAGTCTTTACTGTATTGTCCGTTTGG

>DS499597:2806878-2807279

GGCTGAATAATCAAGAATATCCTCGGCGACTAATGCGGCGGAAATGTCTCGGGGGCTATGGAAGGAACGAGATGGTGGTGGCTCAAAATCACCTACTTGTGAATGACCCAAATCCCTGAAGCTACGGCGTATTGATAATAACAGATCAACTGAGGATAGTGCACCTTTGCAGTATAGTCACAATAATCGATTGGCGGCTGGTGGCGTGTACAACCTCAGGTACCTGCTGGATGTTAGGCTGATGAGGTGAGGCAACGAGTTCTGGGCTTCCAGGTCGCCGGAAACAAGGCTTAAAGCGGCGCCCCTGGATGACGTGATTCCGTTGCTTATGAATTGGAAACGGTCTGGATTAGACGTCAGCCTCAGGCATCAAGGCTCTCGCTCTGGTCTCAACTACCTGC

>DS499597:2816313-2816714

AAGGTTTCACCCTAGCACGAAAAAGGCTTTTGATGGGCACGTGCTCGCTCCACCAAGCGGGGCAATAGACCCTCTGTCACAGTTTCTACTTTCTAGAAGGATAAATCTTCCCTGGCGCTGACAGTCAAGTGACACTCATCAAGGCTTGGGTGTCAGAAGACACTGGGGGATTGAGTAAACTGTTTAGATTGGCCCGAGGTCTTCAGACCTCTGCGATCTAAACAGCGCAAAGCCTCAGGCTTCGACAATTTGTCAGGATGGTTGATCACGGCACTACTTAGGGCAGTTTGACTATACAGATGCCAGATTGGGTGGCAAAAGCCCCGCCAATCAATGCGATTGGCTATCTGCACAATCCCGATGTGATATGGGTTAAGAAGACAAAATCATCAGTAGTGTGA

>DS499597:2881078-2881479

TTCGTACTGAATATTCCTTACTATCAAAGTATAAACTACCAAAGAAAAAAGAAAAGAGCTCTCCCCTCCTTCTCACAAAAAAAAAAGAAAAAAAAAGAAAGAAAGAAAGAAAGAAAAAAGAAACAAACCCATGGAATGCCATGTACTCTGTAGCGCGTCACGAGCCTCATCCATCTCAGCAAGGCTATTCTTTCAGCGCCCCAGCCTATTTGCAACCATTTCTGACTGCGGATACAGCTCACACTATTGCACATAAACGACCAGTAAGACGTCCGAGAGAAATATGCGCCGATGTGGCACGGTCAGATGACTGCCGACTGAAGGCGACTGTTCTTATCGCCGTTGAACTTGACCCTTCCCGGTTGCTATTCTTCACCGGACTCGCGCGTCTGTTCAAGGTC

>DS499597:3018366-3018767

TAAGGCAAGCCGGTATTATCGATCGGTTACCATTCTACTGGTGAGACGGAAGTCAGGCGGATGATAAACAAACAAAGAGCCCTATCTCTGGATTATAAGTGGCTGCTGGGGGGATATATTACGATACCATACCACTGTTATTTTTCAACATGCCCGGATCACCGGCAATGTCCGCACTTACCGCCCAAAATTCCATAAGTAAAGAAGCCCTCAGTTGTTACCATACGCAACACTTTTCCTATTTCCATCAGTTTGTTGGGCTGCTAACCGTATTCTGTACATTCTATTCCTAAAACAAATTCTAGACTAGGCCTAATAAGGCAAGTGTGGCGTGTTACACTGAATTACGACCCAAGGAAAACGCAGCCACTCCCCTTCTTATCTTCACAAAACGTCCCATT

>DS499597:3032527-3032928

TTCGCAACATTCTTGTTTATCACAGCCGTCTCTGGAGAATAGGGAACCTGATGAGTCTCTTCCTCCTCCAGGGCACCTTACCTCACGAGTTAATGCGAGGTACATCCTGCGCCACATTTCCCGTTCCTGGACCAAAGCAAATCCCGATCCGTGGGGTGATCGGGATTTGGATCATTGGCTACGGATGATTAAGCCACTGCAGGACGCCCCTGCTTTTTGGCAGTTACTATATCAGACCACATCTCTGAGATCCTGATTGACCAGCACCAGGGGGAAAAAAGTTCCGTGATCGTACGGCCCTTGTGCCAGTAGGTATGCGCGCAGATGACCCTTCCGAGGCGGTGGGTGCGATATAGACCGCTTTCATTAAGCCCGCACGAGACTACGACCAAATCCCGTTG

>DS499597:3052914-3053315

GTATGGTAGGTACAGGTACCTACTAGGTAGGGATGAGCCACTCATTGGTGGTACTGAGGGAAGATCCCTCCAGTCGAGGCTGTCATTACTTTACATTTCTTTCTGTATCATCGGGCCGATAGTCCATCATGAGCACACCTAGGAAGGACACTGTAATTATGGAGGATTAAGATGATAGGGGCGTTACCTTAGTTTTCAGGAAAGCACACGCACTCACAAAAGACTACTCGCTCGTATCCCAGAATTGTGGAAGAATCTCCTGATCAATACCTTTACTAATAACACATCCTCAATGCACTGTGTACTCCCTTGTACCTCTGTAATTATCAGTTATGTTGGCAAGCCCAAATGTGACTACATATGCATGCTCCAAAGATTAAAACTGCTAGTCAGCTAATATC

>DS499597:3153564-3153965

AACCCTTTCTATTAAGATTTTTCTAGGAGGTTCCCACTTTGCATGTAACAACAGGTACCATAAATGCATTGTGACGCCTTTTTTTTCTGGATTTTCTATTTTTTCTGGACGGGCCCCTCTCATGTCTCCGTCACATCGCCTAAGGAAGGCACAATCCAGTGGGTCGAGGGAGAAAAAAGCGTCATTGTGGAGCTTACCGTACGCTTTACCATACGTGACAACAGTACAGTACCGCCATTTTCGCCGAGCTAGAAAAAAAAGGTTTTATTGTCTCAGGGCCGCAGTTTGACTTGTGGCCTGAGGCTTTCCACTCCAGTCGTAGTAGTCAATGGTGTGGTCGGTGACCCAGATGATTACATCATGAACTTGTCTAGGTGTTTACTATTATTATTCTATTATAT

>DS499597:3154657-3155058

CTGCCACGACAACAAACACGCAAGTCCTCGAGGCGCAATTTGAACATCTACCTGCTCTGGAACCTTTTGGACTAACCACAAATTGACAACACGAAGAGAAACAAATGACTCCTGATGATGTGCTTTGTAGCAGGCCGATTATCCGTCCACCAGGCCCGAATAGACCGAACCCAAGCCAAAGTGTAAGCGCCTGACCAGGGGAGCTTTTTTAGCACAGTAGAAATGCAAAGTTGAAGGTCAGCTGCCCGTCATCGGACCATCACGTGGCGTCATTCAGTGCTCTGGCGGATTCTCAAGGCTGAGTCTTGTACCAAAAGTGCGCGAGGTACAGAGGAATAGGAGGAGCGTTAATTTGGGCTCCCGGAGGCGGTCTAACGTAAGGTTTCCTCTTACCAGCGATG

>DS499597:3156616-3157017

AACCGGACCTTCTTATTGGTCTTTCGCACAATTGCCTCAGTCTAGTCTGTTTGCAACTGATTTTTCACACCATACGATAGACTAATTCTTTTAAAATATCCCCCATGCACCGTAAATCACTCAATCCATCAATCATCAGACGAGTCTGGACCGTGGTTAGATGCGGACGGGACCGTCTTGTTAACCCTGACGACCCTGAATTGGAACTATCAACTTTCCTTCTTCGGGCCCCATTGGCGGAGGACGCCGATGGCCACGAATAAGTCTTGTCGGTGCGCCATATGACAAAGCGATATCGATTATAAACTAAGTAGTTACCTAACGGATGAAGGTCCTCAGCACATTACGTGTAAGATCATGCCATTTGCTGGAGGTATGGGCAATTTTGATCAGAGAATTGT

>DS499597:3469071-3469472

TTTCCAGCGATATGAGCGCCAGCAAATTGAATCTATGCGCAGGTTGCAGTGTGCACCGGGTGCCTTGTACCTTGGATTCAGGATATCAAATGTGAGGTTAATGATTTGTCACATTGCCCACGTGAGAAGGTTCGTCTGGAGACGTTCTGCATCACATGAGACGTGCTGGGTATGTGTCCATCAAAAAAACGACAGCCAATAATAATAGAAGCAACAACCAACCACCTGTGAGGAAAATCCTGGTGGCCTGCCCTCTGCAGGATGTTTTGGGTTGCCCCGCAGGCTAGAGTCCCCGACACCACACTAAAACCCCAGAGGCATCTTTGCGCATGGCATGACCAGGGGAGGGGAGGAGGAGCAAAATCGCGCGACAGCAATTGCCTATGTATAGTGAGCGTTAG

>DS499597:3488568-3488969

ATGAGACGCCAGAACAGGAGGTGACCAAAGTACAAGACTGGGTATCTAAACACGGAATATCAAGCGCTGAAAACAAGGCAGGACAGATGGGTCTCATGCTCGCTGATGATGAAGAAACAACGAAAAGAATAAGCCCGAAATATCGAGAGGACAGAGCGCCGGCTATGCCCACCAACGCCATCGCGACAACGCATCAGAATAACGAGAAAAGAAAACGTGATGTAAAAGGAGGCAGATAAGACCGGGCCGGGAAGATTCGAGATGGCCATCAGGCTTGAACAGCAACTATGACAGCATCATCTCGGGTGAGTCATTGCGATTAGGTACAATGACTTAATCGGCGGCGCCTGGCACAATGGCGGTGAGATCGTGAAGATTGATAGGCTGATCCGTGGCGTAAG

>DS499597:3513202-3513603

CATTCCCTTATTAATGCCTTATTCTTTCCCGTCCACAGCTACACGAACCAGACCTTGACCACAGCCGAATGCGAATCTCTCGTTGGAACACTCCGAATGTCGTATGATCCAGCCATTATAAATCAGAGATGTGTATTTCCGGGAATGCTAGGAGTAAGTACACAGTATTCTGCACTCTGCACTCCCAGGATTCCATTCCCCTCGGTCTGTTGGGGCGCCACTAAGCCCAGTCAGTCCAATCGACAGGGATCAAGCAATAGCCCGAAGGAATCCATCTGTTGGAATGATTGGCCCAATCCAGCCATGAGATGGGGCCGGACAGTGTCTGATTAGCTGCTGCCACTGGGATAGCTCGAAAGAACTGACTAACTGCTCATGAGAAGTGTCAGCAAGTCTGCAAC

>DS499597:3706781-3707182

TATCAATCGGCATTCGGTGCTGAAACCAGTTAGACCCACCGTCTGGAGTCCACTGCTATTACGCGACGTCTCTTGGCGCGACAAGGGTCCTGGCCAACAATGTAACATTACTGCGTATCTTTGCAAGGATGAGAAAATTTTGTACCCGTGGTCGCATGTTCTAACCAGCTCTGAATGCACGAGCCAGGATCTGTGCCGTGCGTGTATCACCAGCGCAGTTGACGGATTCCGTGTGTATGCTGTGCCTGTGCCTCGGATTTGCCCAGGGGGCTGTCCGTGTAGTCCGTGTAGATCCCCCGACGCTCGCGGTCGTCCATGCATGCAAGACATCTTCACATGGCGTCTCTGGGTGTAGGGGTCAAGGACTGTGGATGGGGTCAAGGGAAGAGGACTAGAGACAG

>DS499597:3745576-3745977

CTAGCCCACCTATTTTGTAGGTTTGTGGGCTTGGTCAGCCAAGTAACTATTGCTTAGATTAGAGATCCACACACCCTTATACTACTTGGCAACTACTTCGTACCAATCACTGCAGATACAATGATGCCACATTGGCCATCTATTATGTACCACATACAGTGTTGAACGCCACCGAGCGCCGGTCTAGATTGCTGATGGGCTGACACTGCTTTACATTATCTCTTGTGGCCATTTCCGCGAACCCGGCCTGCTGACTTTTCGGCCGGGAGCCATGAACAAGAGTGAGCACCCGTCATATGATAGAAGGGCTGTCTCGACAGTTATCTTTCGTCAAACGATTGTCGCACCCGCTGTAAGGATACTATAGTTATATCATTTCAAAGTATGGCAATGACAGGAAA

>DS499598:140134-140535

CCTTGACCATGACCAAAAGTCCTAGTCTACGGAAACGGAGCCCACTGGTACCGACGGCGAAGGCCGAGCGAGCCCCACCCCTTGCATGGAGGAAATATGCAGCAAGAGCAAATAGATTGTAGCCAGTCCCTTGGCATTCGTTCACTTCTCGACCACCCCCTTCCCCCAATCTGATGATTGATGAACGCAATCCCCCACACAGATGACTATTGACACGGTGACTGCGAGAGTGATCCATGTGAAATGCTTTGTAAGGGATGATGTCGGCTCCGGATCATCTGATGGTAATCTGTCTGCTTTGATTTCAAATGATGACCAAGCATCATCCCAGAGGTATCTTTTCCTTCACATTGAATATTGCTCCCCTCCTCGAGATAGGAATAACTAATCAGTTCAAAGTT

>DS499598:257975-258376

TGTGCCAAACTTCGGAGGACGAGACTTACTCCGAATCAACAGCCTAAGGCATCAAACATTACTCCGTAGCAGCTTAGGCAATCATCCATCTTGGCGCCAGTAGTCCGATCTATCCGAGAGCGGAGTTGACACAAGTCACCCGAAGACTGCTAGGTTGCATCTGCATTTCACTTACCCGCCCCAGGCGACATTGCAGTGTCTGGCAGTCTGATACTGAGACTCCCTCAGTCACTGACGAGATTTCCTATGGCAGTTCAAATTCCCCCGCTTTAAATATGGCATGCGAGGTTAATACTCTGGAGGAGGCCGTCTCGGCCGTATGATCATGATGCCACCGAACCCCACGGGATGCGGATGGCCCACGCTTCACCGGTAGACCGGGGAATCCCGATTACAAGTGT

>DS499598:276810-277211

TCCGTACAACCCGAGCCTTTCTCTCGAAAGAAGAGGCCTCAATCTCCACCACCAGGGTTCAGGGATACGAACCCGACCACTCGTAATGAGACACACATAGTAGAACTGACGACAAAAAGATCCGATTCGTACCCGGCCACTATTCGACCGTCTGACATGTGTTCTGCGGTGGTCCCTGTGAAGACCAGCAGTATTGGTCAGGTACGCGACCCAGTGCACGCCACCCAGGCACACCAGTCTAACACTGGCTGCCACTTACCAACTGAGACCACAACGCAGCAGCAACCATTGTGATTTGCGGATGCTACCGGTGAACTGGCTCAGTTCTCGTAGACTCATCGTCATCCTTACTCCATATTTTTGACTGGTGGCATCCTGGCTCTACTCTGTAGAATCAGGAC

>DS499598:308316-308717

CCGATGGCCATCAGTGATTATAATCAGGTTAGTACTGCTGCTGCAAGGAGATAACGATGGTTATAAAAGAGGATCAACAATTAAAGGATACTAATTCTGACAATTGCCAACTGTATTGTAGCCAGGAACGTACTCTGTACCCAGGGCACCGACACCCAAGATTACGTATCTCAACCACTGCGGCCACGATAAACAAAAGGGAATGGTGCGCTTTGAATGGTCAAAAATGACGGGAGCCAGAATGGAGCCAGAATGGAGCCAGAATGGAGCCACGGGGCGAATCATCGACAGCATTGCAGCACAATTATTGGTGACAGTGTTACAGAATGGAGCCGACTAGAAGCAATGGGCTCGTTACCGAGCCTAAGAATCAGGATTCCTGGCAAATTTGGCTTGTGACG

>DS499598:381277-381678

CTGCTTATGGCATGGTCTACCAGTTTGATACCAACATACGAAGGAAATATCCAAGGTAGGAAATGAAAAATACATAGACACCGATACTAGAGCCCCACAGCCCCTGACACGCGGAACCCGGCGGAAATGGAAAGATCGAAAATTGCAAACAATCAAGTCCATGCATGGGTCCTTGGTGCGTCCAGCTGCGTAAGAACAGCTTTGCACAGCCCCCGTCCCGAAGCTAGTTTTGCAGCCCACATTAGTCCAGGCCCTGCTAGCCTACCACAGCCAGGGGTTGACTTTGTGCTTACCAGTAGCCTAACCACTAGAGAACCAGCAACGGAATCATCTATTTCTTACGTCCGTAAAGTACTGTGTAGGGATTGTATCACAAAGAATTTATTGCCGGGTATGCAGTA

>DS499598:436757-437158

AATTCCCAACTCGGAAGACCAAGTACAGTAGCTCATCCTCATACGACCACATCTGCTTCAACTTGCTGTCACCGTAGCGGAATCCTTCCGTTATCTTCGGCCAGCCCTCATTTGACAGTCGATGTTGAAGTGTCAACAACCTGACTTTCCCCAATTGACCCTCCTCCGCTGAGCTCCGAAATGAGGCGACCACGCGCGAAGTGATCAGTGCACTCGCACTGCATTGCAAAAGTGAGGCGTCAAGCGTGTGTGCTGGAGAGGTCTATTGCAGTCAGCGTTCACCTTTGGCACGGCATCTATTTATGAAGTACTCGAAGGATGCTCCAGATCATGTAACGGTTAGATGATCTTCACCACCTCGCAAAATGATTTTACTAGCCGCTCCTTATCTATGTATATTC

>DS499598:463477-463878

TAATTGGCTGGTAAGACACGGAGTTATTAGGTGTTTTTATTATGACAGGTAATACTAAGGTGTATCGATGTTTCAATGACCGTCGATGCACGGAGTGCAGAGTGTGATAAACTCTTCCTGACCATACCCAATCAGTCATGAGTCAAAATGGCGTGATGGGTTATCCTCCAGAATTTTGTTAGCCTCCAGCTACGCATCCAGTCTTTTTGGGCCCCATAATTGGTGTGCAGTGTACTCCGTACCTTAGTGGGATGTCGGAGAACGCTGGCCAGTCCGGCTCGGGGTCCGTGATCGGCCACTAATTTGCAACCTAGAAACAGTTTCAGTACCTGGGTACCAGATTACTGGTTCTTACCATACTTACTCATCTCACCATCTACGTTCTCAACAGCTGTCCTCTG

>DS499598:777288-777689

AAGATAACCCAATTGCAGCCAACCTCAAAAACTCCGCATTACGTCCAGTCTAAATCTCCAGTCAGAGCCAGTAAGCAAATGAGGCGGGTGGAATTATAATTGTTCAATCGATCGCGGTTGGAGGATTGTGTAACGTGTACCCACGGTCCTCGAAAGTGTCGTGGCAAGCCGTTACTCGCCATTGGCGTCCTTGCCTTGGATGCGGTGCGATGCAACATGATGCAGGGTGTGAAATGCAAGGCTGCGTTGCGCTGCGTGAGCGCAGTGCACCCGCTGCTTGTGCTAGCTTGGGTAGTACCGAAGTGGGTTGTTGGCTAGGTCCTCGTCTTGATACTGTTATTGCTGCGCTCGTGCTCTGAGAATCACTCGAGTAAGTACCTTGCGTGGTATGTAGCGATAGT

>DS499598:779129-779530

ATTTTTGAGTTGAACAGAAATAAGCAAACTGGCGAACCGCCGTTGGAAAAAGTCCGCGTGCCTGAACCATGAATGGATGTACTCTGAATCCGTTATCATAGGCCAGAAACTGGAACGAGAGGCTAGAAATGTCCCGGGATTTGTTAGCTGTTATCTCAAACTTCAAAGATGTCTATCACTTCCCGCTACTCCGTAAACTATCAGCTGTCACGCTGGACAAGCGATAGTTTGACATTGGACAGGATGGGTGGCCCCATAGAGTCGACGATCGGATCGCTTCCTATTTGCTTGGATGGCTCCAATTCCTTGGATGGGATACTTTTCTCTGTTTCTCCACTGCGATGGGCTAACTCTTGGTCTGGATTCGGATCTAGGGCAAAAGGAGGCGCAAGGCAGGAAGT

>DS499598:784640-785041

TCGACAATTAACGTAATCGGTAAGCGAGCGGATCACGCAACCGAGCGGATCACCATACCATAGAATTTGGCCGCGGCCGAAATTGGAGGCTATTTTGAACCCCTAAAACATGATTTTATTACTAAATCTAGTTATTCTTGCGCTGCGGACATTGCAATCGCCTATGGCCTAGAATATGGCAGTCACTGCAGCGTGGGGGTGCCCGTTGAGGGCGTTGTTCTGTATCTACTACCTGCTCTGGAGGGATAGTTGGGATAGCTTCAGCAGCCTGATCCCTGCGTTGAATGATCTCCTGCCCTTCCTGGATAGATAATCCCACTGCAGTTTCTATCTGCCGCCTAGATCACTTCTGCTTTTGAAGGTGCTTTTCATGTGCAGCACGTAGATCATGATTTTCCTTT

>DS499598:787446-787847

CATCCCAGCTCGTCAGCTGGCTGGGCCCACTGGGGCCAAGCACTAGGTAAACTTTCAGTCTCTGGCCCCAATCCCATATGCCGGCCGGAGACTAATGGGGCCCTTGTTTTAGTGCCTTCGCTGGCGGCGTGGCATTATGGCATGGCCTTTGTGGTCTTGGCTGGCGTTCAAGCTGCATTAGGGAGTACGGCACAGAGTCGGATCAAGGCTATCGATCCAGTCTGGTATATGTGAGGTCTCATGATACGACCCCCCGTCCGCCACGAGGCAAGTGTTTCTAAGTGTGGGCCTCAACCGTTTGACAATTCACTAACTCGTTCAGCCTTCGATATCCCTGCGACACAGATTCACAAGGAGTAATTCACTGTAGCCCGGAGTATTGTACTTAATATCAATGTAAT

>DS499598:916924-917325

GTTACAAGAGACATAGTACTACGGAGGAGAAGGCAGAATTGAGAGAGGCGGCAACGAAGGTGTATCCTCCCGGGAAATGCGGAGACTTACTTGCTGTCTTTCCACAAGGAGATGGACGCCTTGTCTCACCGTAGACATACCAGCCAACAGAGGGGGTGATAAATACATTGAGTGGTCGGCAAATACCGCAGCGGCGCAGCACTGTCTGAGGGCAGTGGAGGGACCTAACCTATGGGACTTGAAATGTTGACGAACGCCGTTGTCACCATGATAGTCTTGTCTCATGTAGGGGTATCTGATGGAGATCAATGATTGGCATGATCAGAAAATTTGGACTGTGAATTGCCACTGTCCCCCCGAGACCTCAGCTTCAGTGGCAGTAAAGATACACAATGCTACTG

>DS499598:921593-921994

TAGAAGCCCGGTGTCTGGAAGGAGAAAGAGTAAGAAACATGTCATTATCTCAAACAGTGATAACTCAAAGGTACATGTCAAGGCGTGCACAAGTAGCCACTCAGGTTCTAGGCTTCCACTCAGTGCCACTCTGAAAACTACAAATTTACATCAGAGACGTCAAAAGTTTTGACATAACCTGATCTCATCATCATGCAACGTACAGAGTACTATGTATATCCAGTGTCATTCAACAGTGCCAAGCCGGTTTCCACTGTCATTTCGCGCCTAGTGGGAATGGCTTCAGAGTAGCACGTCCTTATACTACGGATACTTTCTGTGACACTTTACAAACAATGATACATGCATGACGACTCTGGATGGATGATTCCGGTGACGATACTCAAATGCCCTAATTTATG

>DS499598:922833-923234

TCTAGCAGTGATTCAGAACTTATGATCAGCCCTCTTGCATAGACTGTCGACTAGTGTTCTAACTCAGGCCGTAGAAGACACCTCGAACACTGGACTCTTCCTTCTAGAGCGGCCCCCTTCAGTACCAGCCTAGAGGTTAGAAAGAATCCAAATCCAAACCTCAACACAGCCGGCACGTGTCTCCAGTGCCAAGCACGCGCCTTCAGCATTGATAGTAAGGGTAACATCCCCGTCTGGCGCCACCATAATCAATGATCTAGCCACCTTCGTTAGACACTGTCGATGTGCGCCGGTGATTGGAAGAGATTACTAAAACTGAAGCAATGGTAATCGGAGCATGATTCTGGACGCTGCAGTTCTACAAGTTTCCGCCAATGCCAAGCAGTTATATGTCCTCTTAG

>DS499598:954672-955073

CATAGCCCATAGCCCATGGACGTATGATCCATAGTTCGGATCGGCCATTCGGATATCCGGATTTAACTCAGACACAACAGTCGATGATGACAGCCGTTCTGACTAAGCATGGGGCAAGTGTCCGGGACAACGTCCGAAATCGGGGCGAGTTGCCTATTTGGAGTAAACCGTGCAGCAGACTAATCCCAAAGCAGCCAAAGGCCTAGCCCTTTCCATCAATCAGACTGTTCCAAAGAAGCAAAAGGCGCGCCGAAGCCGAGCCGAGACATCCCGGTTGGGTAGCACTATGTACCAGATTCTGTGCATAGCGTTCTTCCGCTGTATGCAGAGCAGACTAAGCCTTGTCATCGAGCTTCGATGGGTCTATATCTTGAGAGGGTGCTTGGCGGGTCAATTGTGTT

>DS499598:960822-961223

AGAAACTCATGTCCTCACACTGAGACAGATGATGGATAGATCTCGATCAGTTGATTTCACCAGTGCCTCGTCTTAAGTAACGTACGTCTGTACGTGTTGTGACAATGGTGATATGTGGGTCTCAACGGGCCAGTGGTGACACCGCATCACCGCACGTGCGGGCCTTGCGTCCTTAGGCCCGGGAGCTTAGCGATAGTCAGCTCGGAGCGACGCCAATCACGGCTGGATATGCTAACATGGTATTTCTCTGTCCGGGTTGTCTGGCGTGACTGCTCCAAAGATGTCCTCGAGTCAGGTCAGTCAGGTCAGTGGCGGGTCGGTGGGTGACTGGGTAGGCTAGAGTAGCTAGAGTAGCTAGAGCACTTATGCTTTTCTTTTTTTTTTTTTTTTTTTTTTCTAAT

>DS499598:1002543-1002944

CTCGCTACTACACTATTTGGTGCCAATCGAGTCACTGACAATGAATCCAAGCAATTGACAGAAAAATGGCAGCAGACAACAGGGAGGGAGCTGGAGCTGGAGCTGGCAAAAGCAGGATGGATGACGAATTTTTTTCTCGTGCGGTGGATCAGATTATGGGTCCGATAATTTGGTTCGACCCACCGGACCCAGAGCTATCCGCCAATCATAAACAGCAAAAAGCAGGCCGGCGTGTCGGAGTCAATAAAACCGGGCCGATTCGGGCTGCGGCTCCACCAGAACCGGAGTTTGTGCGCCAGAAACAACCAGAACAGGAAGAGCCCAACCACTGGTTTCGGCTGGAGGGAAAAATAGTCCTCTTGTTCCGAGGGAAACCTCCCAAATGTCTCACCGGCTGGGTT

>DS499598:1014405-1014806

CACCAGGGCTGGCAAACCGTACTTTTTATTTCTGACACCATCAACCCACTAATGCAGCTGAGGTTACATTATGTTTACAAGGGACCACGATCGACCAATCAAGGTGTCTATGTGAGTTGGTGGCAATCCTTCTCTCGGAGGCATTCGTAACCCGAACAGGAAATATGACGGGATTGGCGGGCCGTCCCCCAAAGGCCGCCTTGAGTCCAGTCGACCACAATCCACCATGTTGATATTATTGGACTGACGGAGGATCCACGTCTCCCCGTTAGTGCTGAAAATCAGTGTTTGATTACTAGCTTGGAGCATTGCTATTCGTCCTCTGTATCCTTGTACAGCCAACTCCGGGTGATAAGAAGGAGCCTGATTATACTCCGTACGGAGTAAACAGTCACAGCCAG

>DS499598:1025546-1025947

CTCCAGGGAGAACAGGAACCCCACCACGGTGCGAAAACTCGATCCGTCCAGCTCCCCTTGGCGCCGAGAATCGCGCGCATTGGCACTGGCATGGGCCGACCGCTTGGCACCGTCATTAATGGTCTGCCTCATCGTATCTGACATACCCCCCACAAGGCGATTTCCCACTTACTGTGCGCAGGGGACACACGTGCGCCAGGAAGCCAATCAGGGGGGGGATGCCAAGAGGATCCCTCTGCGTGCCAAGAAGGCGGAACTGGCGGGGCGAACGGAGTCCGTTACCATTCGCTGAGAACAGGAGCCTCGGGCGAATAAAATCTGTTATCCAGCTGCAGTGTGTTGGCCTGTGTACATCCTACTCGATCGATCGTAGGTACGGAGTTGAATTCTCAGCGAAACAG

>DS499598:1026614-1027015

TCTTTCCAAAGCACATCCTTGTCTTCGGAAGTGCTAGGAAGAGCTGGATACTATTTATGGCGTCGTGTGTCGAGGGCCCATTGACTCTGCCGAATTCCCGCCCACTCGGATGGATTGGGACGCAGCGAAAAGCATTGCTATTGGCTGGCTGGGTGTGAGTGGGTCAAACCTTGTGATGATCCGCCAATGGAAGCTTCTGGAGAGACGGTCTGATTGGTCAATATTCATTGTTCATTTGTGTGTACATGTACAGAACACATGGTACAGCGAGACGGATGCTGTTGCATAAGAAATGACTGGTCGATTACTCTCACATGCTGGTCGCCTCAGGCAGACTGATCCCTCTAGTAGTTAGTGAGGAGCCCGACAGTGATTGATAGAGGCAGCTCCATATATTAGGG

>DS499598:1170416-1170817

GGCGGTTGGTAAAGGATTTTTCATGTATGTGTCCGGATCAGATAACCCATATATATATACCTACCGACCGGCCGTTTTCAGAGTCACCTCCATACATCAGGTGGACGGAGTCACACCCGGCCCCGTGCTGGCGATGTAAAGTATAATCTAATCTTGGGCGTCCAACGATATTCACAACTCATTCAAAGGAGAGAGCCAGGGTGCATTTTCAACCGAGGGCCGACGTAATCCTGCCAGACAAGGGCCAAGGGAAGATTAGCGACCGGATCAGATTAGATCGGTAACGGTAAGGGGAACCGGGTGCCGTTGGTCGTACGGTTTACAATCCGGTACATCATATTTTACGAGGCACAACTTCTTTCAGAGACTCAAAGAGCTCTTATGACTTTATCGCTATGAAA

>DS499598:1184213-1184614

AGATAGTCCACATGAGATCAGGAAACCACTTTGAGTCGGGGAGGACACAACCCATGGAAATCAGGCAAGCTAGAACCATAAGCTGCTTTCATTCGCGGTTTCAAGTGGAGTGCTTACAGATTGCACAACTGCGGATAGTCCCAGGTGACAGAAAATTGAGTGGAGTCGATAGGCTGAAGTCGCAGACTGCCATTGACCTGGCGCTGTGGGCCGATCGGAAGATTCAATTTTCAAATTTCCCAGAAAAGGTCATATTGTTCACCAATGATTTTAAGACATAGTGGTTCCAATGCATACGTCGAGTCTAATAATCAATGACAACAGTAATTAACTAACTAGTTAACTTTAACTAATGGTTGTCTTCCACGAGGCATGACTTCGTAGGATCATCTCGCTAGTTC

>DS499598:1192400-1192801

CCAATGCTCACTTGCTTCTTCAGATTCCTCTTCTGCTTCTCTGCAGTCAGCTCCTCTGCTACCTTGCAACAATTTGGGAAGGAATCTTCTAAATCAGGCCATCCGAGCATCAAGCAATTAGACCGACAAAGGACAATTTCCAACAAACTTTCGGATGCAGGTACAGTACTCAATAGGGATTGCGTTATCTCTACGGTCGGAGCTCTCCGAAAGCTCTCGGTCATTCAGATTTGCGATATTATTTTGTCTTGGTCTGGGTGGTGTGATAGCTGATCGTTCGTTTTCAAAGGTCAATTGGACGCATTATTGCGCGATAGCATGTGAGACATGTCTTTTCGATGCAACCATTTTATCAAAACCGAGGGGTGGCACGCAGTATGTGAGCTGCCAATGTCCATGCC

>DS499598:1222178-1222579

CAATTATGATACATTTTTGCGAGTTGTTTGACATCTCTACTTGCTCGAACCGTACCATCAGACAAGATATCTGGGACGACTGGCGAATGGGACCTCATCCACTTCAAGGGGATCGTCGCACCCCGGAGCCGTTTGTCAACCAAACGTTTCCGCGCATGACGTAGGGCCACTTGATGAGAGGTGGTGAGCGGCCAGTCACATGATCCATAGTTGGTTCGTACATACCTTAGGTATTGTGTGCAACCCATAAAAAATCAGAAGACCTTACTGGCTTAGGCTTTCGGCTTCAACAGCCCAAGCCAAGAATACCAGGTTTGCCACCACACTACGTACCAGGTAGTCCATACACCGCACCCACAAGCCTGCGTAAACTTCTGTCACGCAGTCCACGGTAGCTTTTA

>DS499598:1230834-1231235

ACGTCAGCCAGTGGGCAGGCGGCGCTTGGATCATCGAGAGAAACCATAAACTTGTGAGTGTAGGTATGGTACCTTACAGTAAACGTACCACCTCACCGAAGATGTTTGAGCCTGTCACTTGATGTGTCTGGTCTCCTTGCTGCTTGCACCGGTTGTATGATCCTGAAACGCTGCACGAAGTACGTAGCTTCATGTGTACCAACTTTCTGTACTTCAAGGTATCCCTATGGATGGCGTCAGCTAAGCGGCCGTGAGCCACCGTAACCATGAGACATTACCGGATAGGCTGCACCCCCACTGCAAGACACCCAATAGAAAGAGTTTGGTGCCTTACTAGTTGGCGCTGTCACTAATATCATGACCAATGAGCATTGTGTCTGTCTGCTTGCTGGCTGGGTTCG

>DS499598:1257656-1258057

AACACAAACACTTTAGCAGGAGAGTTCAGAGCTTTTCACTCGAACAATAATCATCTGCGAATGTCGTCTTCTACCTAGCATCACACAGGTAGCTTCTCCGAACAAGTGGCCTGATATGCTTGCTGGGCAACACTGGCCTCATTCTTGACAAGCAGTATCCCAGTCTCGGTATGATGATGTCCGTATTACGTACTGCCAAAGATGGAGCTGCTGCACAGTCAGCCACCCTAGTTGCCAACAAATCCAACGCTTGGTCCGAACTCTGGCTATGATTGGTTGACTGGCCAATCCATCATCGGCGATTGACAACTGGACTGCCTTAGTCTCATGTACGGCAGACTACACTCACATATGATCCATGATCGCCTCATAATCTCCATACAGATCGTAATGTATGGCCA

>DS499598:1262843-1263244

CCGCCCGTTGTACTCCGTATCTATGTTTATTCACTTAAGAGTGCTCCGTACATTAGGAGGATGTATAATAAGCTCCTTTTGTCCTCAATATAGTTATGCCACTATTATTACTCAGCAACCTATTATGATAGATTGTACTTACAAGGTACTTGAAACGCCTCTTATTGAGTCATCGGGCCGAGCGACGACCGGAAAGGCAGTCTTCTAGCAGTTTCCCCTAGGTATCTGTAGAGAGTTTGGCCAGGCGCAGGCTGTGAATGGCCAAACGAAAACCAAGGAAAACGCCAGGCGGTGCCAGAAAAATGTCCCTGCCGCTCACTTGCGTCACGTTATCTGGCCTATCTCATCACTGATTCACTGTCGCCGTTTCTGGTCCTGGCCTGGCCTGACTAAGGCGCTTC

>DS499598:1503092-1503493

ATTTCCCAAAGTGGACAGATGGAGCGAGAGTGTAGAAATCGCTCATTTTGACTCCTTAATTCGACCTGTCGAACGGGATGGCAATTATGACAATCATGAGACTCTTGATGCTGGAAAGCGAATATCTTCTACTTCGAAACAAGCCACATGTCACGACATTGGTGCTTCGTAGTACAGGCGATATCATGCGATCCCGATTGAGAGGCGGCTGATCCAAGCATTGCCGCGATCCCGTTCACAGTATTTCCGGCTCACCGGACCTTGTTGTCCCCATGAACAATCCCGTCCCTGGCCAACTGTCGACACAACATTTAATCTCCGTCCTGCGACCAGATCTTATCTTCCTGTTCTTCCTCGCGAATACTCTCATTCTTCCTCTTAAATTCTCTGCAACCGCTGTC

>DS499598:1505617-1506018

GAGAAGAAGGTTGCCATGGCCAGTGTTCCTGAACCGGCCTACCTTTCTTAACATCTGTTGAGTGCGGGGATGTGTGTATAGGGAAACCTCATCAGAGTGGATTTGCCAGATCCTAAGCTGCCTGCGATGCGGAAAACGCCGTGGCCATATGACAGGCATGAAAGAGAGGATGTCCGAGGCAGTGGTGTGATGGACGTCCTCTTCCTCGTCATCACTCCCGCTCCCGCCTCCCGCCCTTTTCATCAACCATCAACCAAAGCAGAAGACGTCACAACTGTGCTCTAAATACTCTGAAATCTCATTTTCGTTGTTGTCCTCATCATATTACTGGAATTCATCCAAATATCTTTCTATGAGCTCGTACGACTGCCTGTTTTCGGGCAGTGATTTTTGAGAGCTTG

>DS499598:1512592-1512993

TTAGGTAGTTTAACTAAGTTTTAACTACGGAGTTGTTAGTTATTCGGCGCCGCTGAATCAGCGCTTAGATTTTACTAACTAACTATCTAGGGACATCATTAGCATTATCAGTATTATTCTTATGCATGCCGAAGCCGAGCAATCCGACAGTTGGTCCACCTGACTTTGACGAATCTTGGCATTGATTGTAGTGCGGATCTTTGATGGCTGCACAGCACCTCGGCATGTCAGGGAAGCCGAGTGCTCGATTTCGTACGAAATACTTCGTACAGGCGGCACCATTCTCCTTCGCTGATTTGACGACCTTGCCACGGTTATCGGTATCCCTCTTAGTCTACATGGGCCCACAGCAAAAAGACAGACCCTGTCTCTGTTGAACAGTGATGAGTCGGATGACTGTT

>DS499598:1527346-1527747

TGCGTGCCGCGGATACACTTGTTTACGCACTGATTATGTGAAAGTTACTGAAGATCTGGTTAATATACGTTACTCTGTACTCCGTATGCACCACGAAACCCCAGAAAACCCCTCTCAATAATAAGTGGATTCGGAAGGTCAGCAGATTCGCAAATTTGATAAGAAGTCCCTCCGAGTTTGAGACTATTCGAGTCTCGACCCCTTCTGGTTCCTGTCAGCTATTTCTAGCGCCCAAAATTGAGGAAGGTCATGCGACCGGAAAAAAGAAAGTCCTCCATTGATAAGCGGCGATTCTTGAGATCATTTGACTGGAAGATTATATAAGCAGTACGTCCGTTCCCTGCCTTGCTCTAACCCCCTTCAGACTAGCGGACTCGGTAAAATTAGTTTGGGTCGAAGAA

>DS499598:1653185-1653586

TGTTAAGGTGAGGAGAATGAGAAAGCTGTTAGTCACAGAAATTCGCTATTGAACTATGGATGTTCGATGGCAGCGGATGGGCACACCACCGCGTTTCCCGAACGGATGCACGGTCCAAGGTTGCAATGCGACCAGTAGGAGCAAACGGACGGACTGTCACTTGCGTAGAAAGCTATTAGCGGATCGATAGTCTGGAGAGTTTGCAGATTGATGGAAGAGTAGGAGTGGTGAGAGATGAGGTGGAAGAGGAGTCAGAGCATGTCAGGAGACCACAGGAGCAGAGGCAAAATTCCGGCTGATTGCTATTGATCCTACCCTCCGTTGTATGGTACAGCTTCTAAATAAAAACCTTCTCGAAAAAAGTGCCAGCAAAGCGGAGGCGGCAAAAAAAGAGCAGACAG

>DS499598:1654629-1655030

TCATGCGGAGGTTTGACACTTACATTTACCAGGGCATCATCGGATCATGATGGGCTGGGTCTCTGATTTGTCACTTGAGCTTTCTATTAGCAATGCAAATCCCCCTGGCTCTGCAACAGTCATCACCTGACCATCTCCAAGGATTATTACTCCGTGCTCCCTGCGAACTACTGTGTACTCGGAGTACTGTTCCAACTCCAACCAACTGGCAATAGTGGATTTCCGACCAGAAATTAGGTCAACCCGCTCCAGCAGGCAATAATAATGCTTGGCTCAAATACGTGCACTATTATTGCACAATGGCTGGACCGGATGCGCACTAGTCTGGTAGTCTAGATAGTTCCTGCCTGCGGACTCCGTTCATAGTCCGTCCATCGTCTCTTCTCACCTCAAGAACAGGC

>DS499598:1658604-1659005

GAGTATGCAAAAATTATACATCGGATCGGAATCACAAACGATAGTTAGATTCCCTGCAAGCGTAAGCCACCGTTGCGACCGGATAGAACCCGGCTGGGCTTGCGTCGTGACTTTGGTCTAGTTTAGTTTGCATTGCATTGCATTGCATTGTCTTGCCGTTGACATCACCACGCCACGCTGGAGGCAATATCCGGTTCATGATTGTGTCAAAAGACAAAAACCCTGCTGTACTTGGTGTACATGTCGATCGATGTACCGGGTCTAGAAACTTCTTATTTGTCGCTATATTTGTCTGTCCCTGACCGGTTGACTCGGCCTGCTGCCGAGTTATTTTTGCCACTCGTTGATGGATAACACAACAATCCTCCGTATTAGGAATGTTTCGGGGGAAAATTGGCTTG

>DS499598:1663915-1664316

GCCGAGCTTCCGACAGGCTGCAGCGCCGATGTAACTGCACGCAGTGTTAACAGGCCGGGCCGTTGTAAATGTAATACAGTGACTCTGAGTCGGAGGACAGTCCTCCAATCCAGCACTAGCCACGCAGTATGACGCATGTCTCCATTATAACTCTTCGCACCTCGCCCAATCTGGATATTCTTAAGAGAGCTCGCGTCAGGGGTTTACAGCCTTATTTATCTAAGATTTAATACTGTGACAGCTTATTGGACCGACGCATTATTCCCTGTAGGTTGTCTCTGGAATGCACCGCCTCGAGGAATGAGAGTCGGGAAATCCCGGGACTCAGCCACGCCCGGTGGGCAAAAACGGAAAAGCCCTAAGCACTGAGCTTCCCGAACAGGAAAGACCGAGTTTAGCCC

>DS499598:1679873-1680274

AACCCTAGAAATAGAGGGTAAGCACCCTGTGAGACGTCGTGCGCCCAGGGAGTTCCTGTCTGGGCGAGCAGGATTCGCAGGCGCCTTCTCCGGCTTGGCCGATAGCTTAGCATGTCTGATGATTTAGTCAAGTTGCATTTGACCTCGTGATCGCGATAAGAGCGCTGTCCAGTCGGCCACGTCCGATTGCAGAGTGGGATCATGGGGTGATTGTGACTCGTGGGCGAAAGGCCAAGACTCCCCAGTGAGGCATGCCGACTTGGCACGTTCTTGTTCCACCGGGAATCCGTTGGGGCTGCTTATTATTATCCCATTCATATGTTGCAATATGCATATATGCTTGTGAAGATGACGACATGCATGTATACTTGAAATATGTCTCAAAGATAAGGCCTTTTACT

>DS499598:1800727-1801128

CATATCAGTGCATACACCGCTCTGGTGACGATTCAAATCCGAATAGCCGACACGTCAGTAAGCCTTGATACTTAGCCAGCGCAAGACAGGAGACAAAAAGTTGCATATCAATGAGAAAGAGTTGACACATTAGAGCGCATGATGTGGACAGTCCTACTGATGCTATTAGTTGCTGGCTGATACTAAGGCCTGGTAATTTCTGGGATCTTCTGTTGGTGGAGCTTCTAGACGGTCAATGGCCCGTCTGACGGTCTGAGTAAGTCTCACCAACCGTTAAAGTCAGGCGATCAGAAGAGCCGAACGCTGTGAGCTGTCAGGGACGGCGCCCAAAAGAGCGGGGAGGATTCCATGTGAGGGGGCGGTGCTGTGATGCCAATCACACCGACGGATGATGCTCAGTA

>DS499598:1812206-1812607

GTGCATATTCTTCTGGTTAACAGGCAGATACTCCGTAAGGTAGTTCGTACTAGTGACGTTCAGATGTCAAACCGAGTGATATTCGCTCTTAAACTCCATAAGCAGCATGACTTCGGCGTCTGAACTTTCTTTCGGGCATGAGACATATGTGACGAAGTGCAGATGCTCCAGAACAGAGTAGCCGATTATTTTTCCTTTATCTACTCCATATGGAATAGTACCTTAGTATAAGGTTGTTTAGTGGTACCATGATGGTGCAGTGACACAGCACTTGGACATCCAGTGACAGATAACTGACTGCTGACTTCGAAGCATCATCCCCTTCAGATACTGGCTTGTCGGTGGTCGGTGGCCAGCGATCAGCGGCTTCGTCGTCTATTAATAAGCAATTTGCCTTTTTG

>DS499598:1829189-1829590

ATTGCTGAAGGTAGGTACCTAGACAATAAATGGAAAAGCATGTGCATCCATACGCGACATGGGACAGATCATGGAGGGTGTCAACGCGTTGTGGTTGGTCGCAGCCGGAGTCACGGGGCTGCAGTCGCGTGGACTCTCCCTCAACTCAACTCAACAACCTACAGAGTATTTTCACCTCTCGCACCTCTCCAGGTAGGCTAGATAGATAATGATTGGAAAGTTGAAGCGGCGCAGATAACTAGAATGTTCCAAAATGCGCGGTGCGACGGCACACCAAGGACGCTACTTCTGATTTGTAGAGTCAGAATCCAAGGTGGCGACGGGTTTCAATTCCAATCGCCTCCTTGAGTCGGAAATAATGCAAAGTTGCAGGTTCCCTGGCGAAAATGTAGAGCGTTTTG

>DS499598:1830742-1831143

CACTCCTGAGCATCGCATCTGGAGCTCTGATGGGCCCAACTAGAGAAGGGTATACGAGCAGATGGAGCCACCCCCGAGGTGTACCAAAGTATTCAGGTATCAACTGCTCCCAATCCCAAGGCAAATATGAAAAATTTCACCAAAAGGGACTCCTTTGTACCATCGTAACCAAAAATTAACCAATACACCATGTCTATCATTATCGGCTTTCTTCTCTGGCTAGACCTAGTCAGGCCATAGTGAGGAGGACTGCCCTGCAGCTCATTGCCCTTCTGGGCTGATGCACCCATCAACCAAGGCTGCATCCACTCACCCCCCTCCTTTACCCCCTTCTCCTTCTTTTTCTTTCTCTCTTCCTTTTTTTTTCTCCTTTCCTCTCCCTCTCTTTTGTTTCTTCTCTT

>DS499598:1834880-1835281

CTTCTCTGGTCCTTTCCCCCTCAGCTCCCTTATTAATCCTTTTTATTATGGAATGCTCTCATTGGTTTATCTGGCGATGGCGTGCTGAGACATGAGGCTCCCAGGCCACCAGGCTGCCAGGCATGAGCACTCAGCCCCCGTCCCTTGTGACTTGGACTGCATATGCCGTCTGGAGTGTGGAGTAGATAGGTCTGGGTCCGTGGCTAAGATGAAATTGGCTTTACATGACGGACACGAAGCTGCTGGCTTGCGTGGATTCACGCAATGTAATATCAATGAGACAGTCCAAGGAGGCTGTGATGAAACCGGCTTTCTTAGTGCTGTTGTGGAATTGATGCCTGGATTCTGGATGCGATGTTGCGCCGTGTTATTGGTCAATGGGTCGTGAAGAGTTCCTCCTT

>DS499598:1894551-1894952

ACCGCATCAACGATAAGAACCAGGAAAGTCGAGGATCAAGACATGCAAAAGGTTCTTGCTGATGCATCTGGCCAATGCTGGCGGGTTTGAAAGCGCCAGCACAGGCACAACGCCTGGGATCTATCGCCAATGAGGCTGCAGAACCCGCACATTGGCTGCCCGCCATCACATGCTAGTCGGTGTGACGAGGAGATCAGTCTGCATGCGCTAAGCGGGTGGCCTTAGCAGTAGTCTACGGAGTAGACCTGACGGGATGTGCTCAGAGCTAGTTGACCAAGTCAATTGGTTGCGAAACAGCTAGTTGTCTGTCGACCAGCAAAGCGCTAATAGAAGATCACGGGGGTGTCTGTGATGATGCATGCAGCATAGTCTAATTCTCATTATCCATTTTCATCATGCTT

>DS499598:2000600-2001001

GAAAATGGGACCATGGAGATGATTCCGAAGAATTGGGGACACAGTTGATCGAAGATACGGAGACCGTGTGATTTTCAAAAGCTTCCAGTAGATAATTGCAGTGTACGGAGTAATGCCACAGCGTAAAATCGCGTACCTGCGACTTATCAAACGCCTGGCATGGTTTCGCTTGGGTACGGAGTACTCCTCAGTCAATTCCTTATCTATGACTGCCCCTCCAAAACCCCGCCATGTCGCGTGGGTGGATCATAGCGCGCCTGCCACATTAATCCATGTTAGACAGTCAGACAGAATCCAAGCTAGCGGCCAATTCAGAAGCTATTACTCGGTCATATCATCTCTGCTCGACTTCCCTCTACAGAGCCAACGTTGTAGGAATCAATACCCGATGCCGGACCATA

>DS499598:2068290-2068691

GAAAGATCTGAAGCATTGACGGACGGAGACGGTGAGGAATGGGAATTGGATACATCGTTCTATGGATACATACCATGTCTAGAACCATCTGCTCTCCTCATCGTGACCACCACCTGGATTGCTTTGGTGGCATTTGGGAAAGAGGCGATTGCTCAAAGCGACTCGTGACTGACCATCCAGCAATTTCCATTACCCCCAGAAAATTCTAGGGATCATATGATTGGCTTCAAAGGCCGTTTCTGTTACGTTTGCCACGCCGCGCAGGGACGGTGTGCTCGTCAGATGATCCCTAAAATTTTCTTAAAGCAGCAAGGACTGTTGGCTGCCCACTGGAGTTTTTGCCCAATAGCAGACTGACCTGCTGACACAATTTTGACATATCCAATTCTGATTGGCTGTTG

>DS499598:2133887-2134288

CATGGTGGTTCCGCCCAAGTCCCAAGGCGAGGCGACGTATCGCCGTCTGCTTGTACCCATTGCGATGGAATACTGTCAAACTACAGTTTGAGTCTTGAGGCCGGATGGACGTCACTGGCGAACTACCCAACGACATGATTTTTGATTGGGCCACGTCTCACAGATCCAATAGCCCTTGACGAATTTAGGAAAAGAGTTTGGGAGGCCCCTCCCGAAAGCATCGTTAGGGCCGGGAAAAAGCAGGACGATCATGCAGTAGCCAGCTAGAGGCAGCGGGGCGAGAAAGGAAGCCGTCCAGTTATGGAGTTGCGTGGGTGGTAGATAACTGGGTACCGCAGTGTTGGTGGGGGTGGTGCCACTGATAGATAAGTCATGATTGTCTGTGTTGCTTTCTCTGTTTT

>DS499598:2248005-2248406

ATGTTCTTGTGTTCGCTTTCTTTCTCTTGATCTTGATTTGAACAACTGGCAGCGAAGTGTGGCGAAGCTCGTTCTCTTCGGATGCCGATCGGCCGAAGCGCTCCATGGTGGCTTGCAAGCGTTGACGTGCGTTGCACCACCAACCCTGTCTTTACCTCGCCAGAGTCCCTTGCTTAGTGTGCATTGCCTTGTTCTTGTTCTGGACATTCATGCTTGCTGTTCACTTTACTACTACAAGCTTCTGCTTTTATCTGAACTTGATCCGTAAAAATTAGAAATTCTGGCACAAAGCTGAGCCCTTACCCTGCGTTGTTCTCGCCAGGTGCGCGGGCTAAGTGTCAGTTTCGATATCCCAGGATATCGAAAATCTGAGTGTAGCCACGTGACCCAGTCCTGGCGGC

>DS499598:2317646-2318047 sph4B

TGTGATATCACGACTGGCCATCAACGGCACCACATGGCTTGATCTCTGCATATCGTCAGCACTAGGCTAGGCTATCTCCTCGTCCGAGCCTGAGGAACGAGTCGATAGATAGATACATAGATAGACATGGGTGGTCGCAGGTCGCCCCTGTCCATCAGGACCTCTATTTAGCTTTCTTTCCTCGCGTTATCGAGTATTGCTTTTACCATCTGTAATTGTTTCGCCATCGGACTGAATCTTACGACTTCATCCCCATCGGCACGATGGCCCCAAAATCAGGAGTTCTCGTCCCGTTGTACATCTACCCTCTGTCCACGACGACATGGGCTCCACTCTACGACGCGTGAGTCCACCAATCTTGATTTCTCTGTAATCGATCTTACCAAAGTTCCACTTGCATT

>DS499598:2355625-2356026

TGATCCTGTCCTTGTCCTTCGTGTCTCTAGAACAGATAGGTCGGAACACCGCTAGGACCACTAAGAATACCGTGCTACTACTTAGTGGGTCATCATCCCTGTAGTTTAATTCCACCGCTTGCATAGGTTCGTTGACCCAGGCACACAGTGGATGCCATGCGCCAATGAGGGTGTCGGGTGTACGGAGTATACTGATTCGACAAGTGCTCCCTGCTAAGGAGGCTTTCACATATCTTCCTAGAAGCTTACTCTATTGAAATTTTCCATCCGAGGCACTAGCTAGCTTCCGGGCATCAGTCGATTCTATCGGACCATCCCGAGTCGCCTCGACGGGCTACTCCACCGAACCATTTTCTCGTCCATGATCTGCCTACTTTACCCGGTTCTTGCTATTTTTGCTT

>DS499598:2397187-2397588

AAAGTACCTGTGGAAGCCGACGAGAGACCAGCGCCAGAACCTCCCTTCCCTGCTAGTACGGACGTCCTCAGGCTCACGACCAGTAAGGTCTGCCGACTTCTCCGGATGTCCGCCTGAGGTTCGAGGTCAGAAACGGACAAATGCTCAGGGTCTGAACACCTGGAGGGTCGGAGCGACTAGGGCGGACGGCTACGGACTCAGGGTCTGATGCTGATGACCCGGGCAGCAAGACGGGGTGCGTGGGCGCTGTGACATACGACCAGAAACACGCGACCTAGAGCTTCCGACATATACGATCGAAAGATCGCGGTCGTACGACCGGCACAGACAGAGGACCGCCAACCGGCGTCTGTACGAGGTGCAAGACCGTTAAGGTCGCCTGACTACATTGTTAGAATCCC

>DS499598:2500221-2500622

CAACTCTCAATCCCTCCCTCTCTTCCAACCGTACAGTACTGCACTATATATACAGTCAATTCCACCATTTCGTTTTTTTTTTGTTTTCGTTCTGGCCAGCTCCAATGCGGTGTTTGTTGGTTTGTTGGTTCCACCCTCGCGTCTGTCGGCTTGTCGTAAGCCCCGTTAGTACGTGAACTTAATACCGACGTTCTATGCAAGCTCGCTCTAGCGTCGGCGATAAGGATTGCGGCGGAGTAAGCCTCGCGCTTTTGGAACGCCGATGTGCGAGGGAACGCCGACCATGGGTTTACGTACTTGGTTGGTTGGTTAGGTAACGTAATTCCCGGCCGCTCCGATCACCCGGTCGCTCCGATCACCCACCCGCACCTTCAAATACTATACAACTCTACCAATTTCAA

>DS499598:2519577-2519978

ATGGCTGAGACCGGGTCGAAAAAACCAAAGGGTCATGTGAGGTCCCTTGGGGTCCTAGTCACCCCGGCACAGTCGGGAGGATGCCGCGTAGATTAAGGGGCCGTGGAGTGCTCACATGATATAATTGGTTTTGCTAGCTCCAATTAGTTGCAGGATTAGCTACCCCAAGCTCTGCCATGGACCAATCTCAACGTCGGGATGGGGGGGATGGACATGGCCGCGTGGGGTGGGCCTGCGGCGCGGTCTGGAAGCGGACATGTTTTCTTTTTCTGGACCCGTCGAGTTGCGAGAGACTGGTCAAACTACTTCGTACTTCGTAGATGGGTTCATCCATACAGAGGACACAGTCTGGAGCATGCTCGCAGGGGCGATACCAGTCCTCTCGGACACTGGACTGCCCG

>DS499598:2554361-2554762

GCCCCACAACTAGTCAATTACAACATGACTAGATTACCTACAGACGGATCTACCATATTTTGTCATCGTTGATAGCCGTCCAATACTCTTTAAAGTGGGACACTGAACTTGTCCACTCCAAAGTTTAATATCATGCCACTGCAGAGGGGCGGGTGGCAAACTAGGAACAGTCTTGTCAATTCAGACAAGTTGAATAGGAGGTAATTTTGAGTTGAAATGTCATATGAGGTATTTCGCCACCCTCGAATGTCTTTAATGTGATCTGTGGATCTGGATCATAGCTATTTCTAACGTCTCATGTGGGTGAGCAAACATTAAGCTAATATGGAAACTTTCTAGCTAGCTTTAGAATAATGATATCATGCCACCCAGCTAGCTCACTACCTGGATGCTTCAGACTT

>DS499599:156249-156650

ATGGTCGAATGATATAAGGCGACGGTCACTGATAGGTATGCTCAGATCACGGGAATCACAAGGGCCAACAATCAGAGTGGCCTTGCTGCCTGAGGCATTCAGTACCGGTGTCTGACAATCCGGATCCGTGGACGTTACTCCGTAAGAACGGGGCGTAAAAGTTCAAAAAGCACGACGGGTAAAGTCGGGCAGCGGGGAGGCGGAATGCACTGAATGGGAAATTCGGCCTGAGGGTCGCGAGTCGCGAAATGGTTCATGGGCATTATCCGTCCGTGGGCTGATCGTCCAGTTAGAACTGTGAACATGGTCACCGGGAAGCGGATTAAGTAAGTTTCCGGCTATCAGCGAGATGATTGGTAAAAGAGTTTCATAATCTTAGGCTAAGACTGATACCGTCCTTG

>DS499599:164313-164714

ATTCTGTACCAAAAATCATCCCATTAAGAATGAATTCACTGCAATAGAAAGCGCAGAGAAAAACAGAGAATAATGTCTGATAATAAGAGGAGAAGAATCACGTCATGTCTCCTCTCTAGGGCTTCAGGTCGGCCAGATAAAGAACCAGAAGGGAAAATGTGCTCGTAACCTGAAAAGAATCTGGAAGCCTCTGGGTGGGGGATTGTGGGCGATTGAATCGTGCTCGCCACTGGTCAGGAGATAGAGGGTGGATCTTTCATCCAACTCACAACACACAGGAAAAAGAAGAAAATTCCAATAGGGCAAAGATACCACGCCAAGTGGGCCCACTACCGCATAGGACCTAAGCGCTGAGTGGCTGAGACTTGGGCACCAGGCGATTTGAAATTCTGGAATAATCA

>DS499599:168193-168594

ACGTTCAGCCTTAGCTCGCCAAGCCGGCCAAAAAAAAAAAAGAAAAAAGGATGCCTTCTAGAACAATTATAGAAGCTGCCAACTGGAGTTACAAGAAGCCACATCACCAATAGTTCGTCAAGAATGCACTGTTACGGAGCACAGCGTTACACTTTCAGTAGCATCCTTCCTCTTACATTTCACCGAAGAGACTACTACCTGTATCCATCAACCGAATCCGTTCGTTCGATTTTTGCCACGTACAAAATTTTCAGGGGAATTAATGTGGCATGGTCATGCTACTGCGCCATCATCACCAGCGTAGCCATTCCTATTCTTCCCGATGGTTATCTTATCCACAGTATACTGCGGAGGTACACTGATAAGATTCGAACGATCGTCGAACTGGTGTTTTAACACTA

>DS499599:171308-171709

CTAGTTCTGCAGAATCCTCCCCCCCTCCCTTCACCCCCACCAGTAACAGGCTAGTCTCTCCGTAGGGAAGTCACAACCGTCATTCGCCATGCCTGAAACGGGGAAAGTCCATCTGTCGATCGTTGGACTCCATCATCTAGTTGCTGGGAGTCTCTCGTTGGTGTTATGGCTGGAGGGTATGGAGTACTGAGTGGCTCGAAGCTGCCGAACGTAGTATAGTGCGCGCTGCTGAAGAGGCACATCAGACCACAGAATCCTTGTCATCTGCCATCGGGATCGTCCGATTCTTGCCTCTGATGTCGGGACAAAGTTTCCCTTAGTCTGTCGAGTTGTTGATGAAGGTGAAAGACCAGCCCGATCATCCCATTGTTGATGATCTGTCTCCTCCAGCGAAGAGATGT

>DS499599:352888-353289

TGTGGACTCTTTACTCGAAAGAGTAACAAGATGTTTTAGCAAGGTGCTGATGGCCATCAATTAAACTACATGACCCCTGGGAACCATTCATCTAGAACCTCAAAGTACCTTGACCAGCCTGAATCAGGTGATCCACAAGCTATCCCACCGTCATTACCTGACTGCTTTCACAAAGTGTACCTAGTACTACCTAGTAACTCAGTAGATGTGACAACCTCATAGACGATGTAGATGCAGCAGCACGGAGTAGCTATTGCTCTCGGTAGTCAACAACTACTAGTTTCTGGATCGGTGGTCTACGATTGGGTCCTGAGCTGAAGATTTAAAACTCTCCTGTAAGTATTCTCAGAGTGTGGGATTTAAGCTCAACCGGTCGCGAGCTGGTATTTGGTATGTTCCAC

>DS499599:409622-410023

GGATGCTATCCTGTATTGCCATGGAGAGTGTAGAATCAGCCGGTTTCTGATTCGATCATTACAGCGCATGTCACACCACTAAGGCCTCGGGTAGTCTACGACCACTCTAGCCAAGTATCTAGTTGGTACCCTTTGTCTATGATTGTCTTGGACATCTTGTAGACTGTAACTAAAGCTGCAAAGCATGACGCTGCAGCGAAGTTGTAAAATGCGGGGTAAGGTATAGCTAGCAGTTCTGGGATCACACTATCAGCGCATCCTCATCGCTTTCTACATGATGATGATGATGAGGTTTGGATCACAGCCTGTATGGAATTAATGAGAAACTGTTCTCATTCAGACCAAACAAGCAATTTCCCAATCCATCCACGTTGCAGTCTACAGAGTCTGCTGAAACTCAG

>DS499599:417019-417420

GATAACAAGAATGTTCCCATCGAAAAATGCCTCACGTTGCACAAAAATAGTGCACAATTGGCTTTCTCTGATTTTTCGGGTCTCTGGTAACTCGTCAGGGATTTTCACAAGCTTACAACACAGCTGCGAGACTCCCAAGCTCCGACGCAATCCGATATTTCGGGATCTAAGAATAGCCTTGACTATCTATTGACTATGAAGACGGAGTATGCTTCTTTTTATCACGGGATCGAACAAATAGACCCAACTGCCCCTTTCTTTGCTCATATTAGGCTTTTTCGCAAAAAAAAAATGGCTTGCAGCTCGTGACTGCAGTTCTCTGGAGTCACCAGACCAGTCTGGAACAGCACTATCCAGCTCCCTTGCTCCTAGCTCAACCCCGAACAACTCAACTCCTACAA

>DS499599:430585-430986

CATTCCATTGACCCGTTAAGACCAGAAGCGCAGTATGACCTGGCAGCTTAGGCTCGCATCTCGCATGCGGCCATCTCCGTTGGCGTTGGGACGTTCAGTTTCCCGGCCGACTATTCGGTTACCGCGCCATCAGGCTTTCTGCTGGGCGGCAGGGCTACCCGCGGGGGTCTCTAGCCCCAGGACCTCTCCAGGTATGTTCCGGTTTGCTTTCGGGAGACTCTCCCTAGCTCCGTTTGGTCCCGAAAATCGTCCGATCAGCCAACCATGAACCATCATTGGATGCTGTTCTTTATGAGGCAATAAATGTATGGTCTGGCACTTGGCATACGGCCACGTCCCTTCCCCTCATCTCGTGACAAATGCGTCCCTTTTACTCCGTACCCACCCCACCTTTGAGTCAA

>DS499599:438927-439328

ATTCTCCTGGTTGGCGCACTACAAACTGCAGGATGGCGTCACAGCTTCTCCTTTGGGGCATTTGCAGCTTTCTTTGGATGGGTCCTTTACTTCGTAGTAACTGGTTACCTGGAGGTATCAAGGCCGTACGGAGTACTCCGTCGTTTCGACGCGCACTGGGGGCAGACGGTCATGTGGCGGGGCCCTGATCTGCGTCGTTCGATTTCTCCCTCTGCCAATCATAGTCACCCGGTGACCCGGCAAGGGTAAAACCGGGGCTTTCGGTGCCCCATCCTGGTTTCTACCCGGCGAACAATCAGCGGACAGTCGTTAGCGACTCCTCGTCCCTATTTTGGTCTTTACAAGGCCTCTGTTGGCCACTGAACGATGTTGGCACCTTGGAAATATGGCACTTAGAACAA

>DS499599:483251-483652

ATTTGGGTCAATGGCGATCATGACGGGCTGTCCTGGCCCGAGTCAGGGATGGATCCCGACCTTTGATTGCCGTGGATACTGGATGGGAAGAGCTACTAATGATAGATGGATACAATCGGAGAATGTGTGATTTTCCCCGATCACATGTCATTGTGTCAAATTGTCATGAACTGGTCCATGTTTACGCAAAGCTGAATTAAAGGAGCTTACTGGCAGCCTGGCACTCTTTTGATGTCACTATAATTTCATGTAGCGACGAGCCAGGAACGCTGTAGCTGGCTTCGTGGCTGTTGCAGACGAGAGAGTGTTGGTCCAGATGTATGCAGCATTTATGCAGGTGCCTGGGGGCTCAAGCACATGCAGGCCATAGTCCACATCCAACATCATATGGCTGAAGTCAT

>DS499599:484334-484735

CATGCCAGCCCATTAGCACATGTTGACAGACAAGACAGGATGAGCAATAGCAGTGATCATTGCTCGAATCACAGGTAGTCGTATAAAATCTTGAGGCTATCATGCGCAAGGTCGATCCCGAGGTTATATCTCTCGACATTTGTATTTCAATTGTGTGCCTGGAGAGGACGAGGATAATAACCACCAGAAGGGAATTCTAGAAAAACATCAGACGCCAATCAATCACTGCATATGCCACCTATCAAGCTCCATCTTGTGATTATGCCACCCGATAACCGGACCACCCGAGTAGCCTAAGCCCTTTGGAGGTAAACCCGGCGATCCCTGGCGTTATTCTTCACTATCATCAGGACCAGGGCCTCTGGCACTAAGTACACTAGATAATTGAAAGTATTATCATT

>DS499599:734579-734980

TTCCACACCCGCCGGCTGTCTCTGCCTTAGGCCAGGCATTCGAGGCACAGGAACCGGCTAGCATCGGGCTTCTATTCTCGGCATATTCCAGTTTGATCCTCAAGTCACGCGAGGTCGCAGGATGAGGTGGACGGGTACTCAGTAGTCGATTATTGTGCAAAATGGGTTGCACAAAGTGATCATGATCTTAAATTACCTGACCTTAGTCAAGCCAGATTATGCAACTAATGGAAGCCGAGGTCACCTGCTCACGGTCACGGGTTCGTTTGCATTCCAGCAAAAGTGACCGGCCCGGTCAAGCGGCCTGTATAATGTGGAGACATATTGGCCGGCCCGCGAACCGGTTGTCCTAAGACTCGACTACCATTCCCAGATTAGTCTTTGGTTGCTTGATGTCAGCT

>DS499599:821665-822066

AGAAAGCGGTCAAGAACAACGCAAGAAAGTGGATCGAAAGTTAATTTGATAGTTCCCAGGATAGAAGCTCGGTTCCAGGCCAATTTGGTATTGAAAACTTGTTATGGGTCACTTCGTCCCCCGTTTGGCTGACTCGGTGTCGCCACGTCTTCGCGCCACGCCACTCCTACCGCAACCGGTCGAAGGTCAGTTGCTCAAGGGGTTGGAGAAATCACTCGAAACACCTGGAGCGCGATGGGCTAAAGCGAAGATTCCCTGATACAATGCGGAATCTCAGGGCAGAGACTCTGATTCAAGGCAAATAATCATAAGACCTCCACAGGCTCATGGATCCCGCCTTTTTTTACCTACTCTGCACTACGAAAGGTAGTGCACGGCTATCGGCGGGAAGAGAGATCATG

>DS499599:909757-910158

GCACACCTAGTTTGTTCCTGCAACAAAAAGTACGAATTTACCAAATCAGATTGGCTCATGTTTGACTAGGCGCCCTTTCGTAACGCCACAGAAAAGATCTCCATAAATAAGTGCTTCTGTAGTCGACAGACGTGCATGAAGCCGTATCCCTTTCCCCAATCATATTTGCACCGATCTTGCCCATCAGCTGAAGGGACGCTGAAGGGACGTCATCGCGCGGGCTTAAATGTAGAGCTATTCCCATCTTCCTGTCCCGTCCCTTCGCTCCCCTTCCCCTCCTCCCTTCCCATCACTTACTGGCACGTCCTGTCCTGTCCCTTCCCCTCCCTTCCCATCCCTTCCCATCCCTTCCTGGCACGTCCTGTCCCTTCACACGCCGTCCCATCTTGTCCTTTGTCTTT

>DS499599:911757-912158

TGCTGAGTCATCAAGAGGGGCCATCCCAATCAGGGAAGACGGAAAAGACGTCTTCCGAAGATTGAAAGGTGTGTGCCATGGGATGTAAGAAAATAAATGATATACCCCACTGTACGAGACAAATGATACCTGATGCGAACATCTCACGCCCTTAAGATGGAAAGCAGCGCTTGTTTGTCACTCTAGAGCCCGGTTTTGTTTTCTTTGTCATCCATGAATCGGTAAGATGAGGGATGATAGCAAAAGATGGGGAAAGGGGTGACGGAACTGGACGGGACGGGACGGGACTGGACGGGACTGAAAGGGAACACCACACTTGAAGACAAAACGTCAAGTATAAGCTCTCCTCACTCCTATTGGTTGGATGCGCTGGAAAAATCCCTAAGCGATTGATAGAGACA

>DS499599:1145642-1146043

TTCACAAGGCAATCAATCAACGCCTTAGGCCAGTCTTAGGATGGGTGTTTCCATAGGATCATGGGGGGCAAATTGGGTGCAGAAAGGCAGCGTCAATTGTCGATAGCTTTTATTGGTTGAACAAGGGCCAGTAGATAGATGGAAGCCAATGGTTGTATCTGATGGCTCCCGCTGCTTTAGAGCGGAAATTTTGAGTGACCCGGTGCTTAGAAATCACATTTGGCCGGATATTGGTTCTCTCCGTGCCGACGAAGTTCCACGCGCCATATTATCTAGTTCCAGCCATAGAAATGCTCAGTCAGAAGCTGACTTGCTACTTCCGTGCAATGGTCCCTCTTTCAACCGGGGGAAATTAAACTTGCTTGCTTAGTTACTAATTAATCTATCAGGATGACTCATAG

>DS499599:1820983-1821384

GATTTGGGAGAGGCTGGCAGAAATCAACCAATAACACCAGCCATCTTTCTCATAAAAGAACCTGAGAGGGAGTCAAACAGCTTTGTTCTGTCAGATTATGTAGACTTTTTTATTCTGTGTTTATCTACTTTTAATTTTTAAATAATGTGTTGGCGTTCCAAGTTTTATAGCCACCACCTTCTTTCAATCACATCAAGCAGGTTGAGACTCGAAGTTTAGACAAGCTAAAGTGTAGTGGTGGATTCACAGTAGCAAATGACCAGCCACCATGAACTCCGGAACCCATGGAAGCTGATGGCGCCGGGCGGCCCCTGAAACCAGATCCACTCCGTTCTTGAGACGCAACAACGATAGGCTCGTAATTATCATGGGCCCTAATACAGGGCTCGTAGAAATGTAGG

>DS499599:1822029-1822430

CTTTTGTAGCTGAATGCAGCCATGAATAGAATCAGTTTCGCCACTCTGAAGACAACTAAGAGAATTTATGCTTGGGAGATTGTACGGTCTATCCACGAGGTATGGTCTGCGAATAATCCCTACGTATCAGCCTTGCCTTGGCAGAATAAGCAAGATAACACTGTAACACCAAGTCTTAAGCCTAAAGTGGTATGGTGCCGCAGAAATAAGTGTCTGCCGATCCTAATGGTCCGGCGTCAGGACAGCGTCCTCCATTTAAATCAACATATCACCAAATTCGCTCCCAGCTCTCACAATTCCGAAAAGTAAAAGTCCAGATATCTCAGCCTGAAGCTCGAACGTTACGCCCACTGTTGGCTGACAGATTGATATCCATGACAGCACACCGTAGACGGCTTCTC

>DS499599:1823329-1823730

TCTCACAAAGGTTTGCCAATATATAGTCCTCTGTAGGTGGGTTAGGCATACAGATTAGCTACAGCGCTGGTTGTTAGATAAGGTAAATGTGCATGACGCATTATCTATCACTCAGGTAAATGAAGCATGCAACCTCAGATCCAGGTGTTATCTACATCTCCGTCAATCGGCGGCCACGGTCCACAAAAGACCCTGTGGCGACCGAGGAAAGGGAGCCAGGAGCTGTCGAGCAAATGGTCAGATAATCCACTCGACAATTGCAGTGCTCTCCTTGCAGGATATTGTGCATTATCACCATAGTGTCATCTCCTCAAAAAGGACCCGATGCCCTGGCCGACGAATTTCCATTTCAAAAACGCGATCTCGAAGATTCTTCAATCCGCAAGTGGATATTACGCCGT

>DS499599:1902901-1903302

GAAATACAGAAAATTGCGATCATATTCCCGGTAATGATCAAGTGACACCTTCTGGCGATAGGAATCTGCTCCAAGAATTATACTGGACATAGGCACACGGAGTAGGTACGCCAAGAGAGCATGAGCCGCACCGATCAGATCAGACCATTATTTTCAGCGGTGAGTGCCTCGTAACAAGCATGTGTGGATGACTGACGAATTGAAACTTCGATCAAATAACTCCCAGTGTTGTTCTTGGAAAAAATCCTGTCCAAAGGACGTACAAATAGAGTAATTTGGCCCCGTCATCGAAGGAGGTCAATGCCTAAGCCCTAGTAGCTCCGATTCAGGCGCTAAAGCCCAGCTGGTAGTCTTGGTACCTGGGAATGAGGTCATCCCATGGCACGCCACTGGCCAATAGT

>DS499600:25495-25896

TAACATACGATTGAAAGTTGAAATGGATATGGAGGCAGTTGGTCCTAGTACATATAGCGTTCCAAGGGTGGCCGGGACGCCAGATGCGACGGTCTGCCTTGCCGCCCGTCTCAAAGTCATGAGCACAGCTGCCGCCTTTCCAGTCAGGATCCCTGCTGAATTTACCCAATCTTACTGCAGCTAGTGGAGACGTTAGGTGTTAGTGGCGGCTGCTGACGCTCATTAGTGTCTGACCACAACCAGAACTGTTTGTCTCGCGAATTGTCGGTTGGCTACAATGTGGTCATAGGAGTCAAAAAGCGCCGTCATAGTCTTCATTATCCACACTCCAATTATGTCCTCCCTTCACGATGTGAGGTGAGCGGCAAAAAATCGGATTGATGCTGCTGCTGTATTTTCGC

>DS499600:252850-253251

TAATCGTAAGATTTGGAGCAGGATTGATCAACAACACTGCAATCCAGAATGTACGTCCTTACTGCTTGTAATCATCTGAAGTGAACGATAATAGTAGACCAAGAATATGAGAACACCCTGAATTGGACTCCGGTGAAGCAATACTGAGCAGATCCTCAGCAGGGAGACAAGACATTCGCCCGGGAATTCCCGAGAGTCTCCGGCCGATTGCCCGCTGACAAATTTGAAGCCATTTCAGCGGTTCGGACTTGGGCAGTTTGATCCATGAGCTAAAAGTACTTGGCACCATTCAGCGATGGTGTGTAAAGTTTTTCTGGAATATTCAGGACCGAAGCTTAACCGTGGTTTCTTGCAACGTATTTTTTTTTTTTAAGGTTGGTGACAAAAATCAGGAAGAAGAT

>DS499600:255718-256119

TCTGGAGAAGCGACACGTGTAGGATGAACGCAAGGGACACAGATGATGATAGACGAAGCCTTTGATACCTTGACTGGCATGGATAAAACTCTGCGGACGATCGACGGTGGCGCCGCCCAGAATCCGATCTTAAGGGCATCTAGCACCCCGGCAAGCAACAGGACACCAGCAATTGGCAGGAATTTCCGCCACCTTCAGCGCAGCCAGGGCCGAATTGGGTCTAGTGTGGTCAGCTGAGATAAGATAAGCACTGGAAAAGGCGAACGGGGCGACCGGGCTGCGTCGGATTTCGGCGAATCGGACATGTTGGACATGTTCGACAGGTTCGACGACTTCAGGAACCGGAAAAAAGTCAATTCCGCCAAGAGTCTAGACGATTTCGGGTTAGTGTCGGGTTGCCG

>DS499600:256822-257223

AAGCTTAGTTGGGGATTGTGCTGAAGTAGACACCTGGGGTCAACTGCTGTCTCACTGCCGTCTGGTCCTTAGAGCAACCTTGGCTATCGCTCCGATATTGCTAGATTGCAGAATTGCTAAGCAAATGGTTAACTACCTCCGTTGGGGATTTCGGCTGAGCAACTGGCTGTATTCCATGGCGCTGAAACAAGAAAAAAATCCCGCATCATGCTTAGCAGAGGTGCATATAGTCCACAGACCACTCTGGAAGCAGCAAAGACCGATTCTCGGAAAGAGGCGGGTTCAACTCTGCTGCTCTGAATGCACTTCCACTGGTTCAGCAATGATGATGAACTGCCAGCCTCCGATGATAGCCATTTGACCAGGTCCGACAGTGGTCAGACATACCTATGTAAAGTATG

>DS499600:257682-258083

TCGAACCAGTCACACACGAAGGCGAGGGGATGGCGTGATGGCAACTGATCTTGTCTACTAACTTGTCATCAGGCCACGAGGTATGTCGAAGCTCAACCCTTGCTGTACACTGCCAGACGTGCTCCGGTAAAGCTTAAGAATCTCATGGTCTGTCTGCCGATCTATCACGCCTGATTAGATCACAACTAGATGCCACGATCTGCTGGTGCCCAGTTCGCCAGGCGCTCTAACCGCCATGGGAACCTGTCAAATCGAACACACGATTCTGCAAGGGATATCACGATTGCCACCGACCAGCTCGTCTGTCAGATTTCAGCGCTCAAGAGTGGAAGAGCTGACATGAGGTGATTGAGATCTGCCCACCATCCCTTGCATATTACGAGAAAGCGGTCTGTTCGATA

>DS499600:509517-509918

AGACATGCGACCGTAGGAATCCCTTTTATAACTGTCATTCAACCCCGCAAAAGATGACTGATGACAAACACGCAATGGTTCTTGTGTCTGGAGATGAACCCGATAAATGGTTTGATGGCACGCAATCAGATGGTTGAGCGATATTCAGTATATGGAGTTGATTGGATGTGACCCTTGGTGGTGAGATCATGGATATTACATGATGGCTTGTATTGGTCCATGGACGGTGATTTGCTTGCGGGAAAGACCGAGTTGGTGATGATCCATGGGTGGCCTGACTGACGATTCTGAGTGGTGAGATAATGGGCCGATCTGGGGTATTCAGTTGATATATCAAGATATCTTCATCAATTGACTTTCATCTTATCCGAGAGTGTGGTACATGAGGTTAATAGTACTCC

>DS499600:604758-605159

GTCACTTTGAGTTTCCCTGGTCCTTGCTCCTCAAATGATCCAAAATTGAAATTTCCAAGGGTCACCACTCCCCAGTGCACTGAAGCATTGTCGAGGACGAAGTTGATCCTGGACTTTGCTGGAGACGTCTTTTACCTCTGTAGTTCATTGCTCAAACATCCTCCACCACGCGCAGAAAGTATTGGAAGGAGACAGACCATCAATGCTCCATGCGTCCTGACACGTTGTCTGAGACATTGACCTAATGCATGTAAAACTGTACTGAACTCCTACTGGTGCACCTAAGGACACCTCAACGAGAGTATTCTATTTTGGGATACACCACGGAGTTAGAACAGCCCACCGTGAGCTTGTCAGCTCATCTATACTTAAGAACCATTGCCACTAATAACGATCTTGTC

>DS499600:607381-607782

GTATTGCGTAACGACATTTAATAATACTGCTATCCTTTTCAAACGATATTCTCAGGGCCTTAATCCAAAAAAAACGTAGCATTAAGGTGTTCCTGGGATATTTAACGGGTCCAGTGATTTACACCACCTTCGATCTGGTTCCAGCCAAAGCAAGATGAGAAAGAAGACGATGGAAGGAGAAAGACAAGCCCAAGCTTACACATTAAGAGAGGGAGAAAATATCACGATCCCACATGACCATTTCCACTGTACCCTCAGGTGGGGGTTTCATAACCCTTTTTTTTCCACATAATACTTCTGTCTGCCTAAGATTGGTACCGTCCACAGGTAAATGTAATACCCAGAACAATACAAGGCGGAAACTGGTCCAAAGCCGCAACTAATGATCAGACTAGAGCCAT

>DS499600:620633-621034

CAACAACATCAGTTCTCCCGTCGCATGACAGCAGAATATATTTCACAAACCAACCAGAATCCTAACTAATGACATTACCAAATCAATTATGAAAACCCAGAAATTATACAAAGGCGGAACCTGACTTTCCACAGGCAAGCTCTTCAGAGTACGAGCAAGACCTGGAAGATGGAACTTCCCTGGTCCAAGATCATGCCACCGCCGATACGGCGACCACACCACCTCATTCTGCCGCGCCACGATCCTGGACCACGCCATCCTATCATGCCGTCGTCAATTGAATCCGAATGATTTCTGTAATTCAATTGGATGATGCCTTTCAGCCTCCGTATTCACCCTACAGAAGCATCACGAACATGTGGGTATCGTCCATGTCTCAATCTCGGCGTCAGGTAAGGTGA

>DS499600:658199-658600

TGCCAATAGAGAGAGCATGGAATCCGATCTGTCTGTGCTTTTCTGCATCCACTCTACATCCGCGGGTGGCCATCTTTCAGTGGAAGAAGCACGGGCAGGTTGCAATGATACACAGACAGACAAGGTCAATCATCTTGGACTAACCAGAAAGGAACAAAGAGCCTTTGGGTGGATCGTGGAAAGAGATTGGGATCAATGGAACCTTCCTGCGGGCCGGGCCTACTGACTGTTCCAGAATTGGGAAGGTCACCGTCGGTGTAACTACCATAGCATCAGTTATGGCCTTCAAGAGTGTAAGGCGTGTAAATGGCCCCATTTCAATAATAACTCAACCCCGAATTGATTCCCGAAATGAAGTTGGTGAAGCGAGCAATGAGCTCAACGGATCAGACAGCTGAAGG

>DS499600:728863-729264

GACAAAGAACTTTGCAAGTTACCTGTTATTGAAAAGTTATGCTTGAAGAGGGCTAGATTACTTAGTGCTCTGTAGCTACGTGTGAACTAGAGAAGAGTGGGTATTGTCCGTTTATCCCCGTGTTCATCGTTATGAGCTGGTTTTGACTGACTCCATGGTACAACGAACTTGTCTCCCCACGCACACATTAGTTTTGTAAGGGAGGGAAATCCATGGAATAATTGGAAGTACTCCGTACGAAGTTACGATGTAATAAATGCGTACCTCTGTCAACAGCACCGGGGGATCACAGCAAAAGTACACGGACCATCAGTACTATTAGCTACTGGCCGACCAATTGGCCCACTGGAGCAAGGATCGGGATATCCGCGGTCGTTCCAGCAGCAAGTGGCCGCAAAGGG

>DS499600:767115-767516

CGCCAACTCCGGAGTAGTCAGGATGTCTAATCATGCAACTATGTAAGTCTGTGTCACCTGCGCACCGCCTCATTTACATCATCATCATACCCATAGTGCCTGAGTACACTCCTGGTAAGGCAGGTTGTGGAGGCTAGCGGAGTCTAGCGGAGTCAAGCAAGCAAACAAACAAAGCACGGAATTTAGCGAATACCCAAAATCTTGTCATGTGGCTCATGGCTATGAAAATCCATAGCGCACAGCGGAGACATTCCGTCAGTAGGGAGTCTGCACAGCCACTTTGTTCCTAAGGCCCACATCCAAGCCACTCTTTGGTTTGTGATCGAATGTACGGAGTACAGAAAGTTGGTACATACCGTACGGAGTGCAACCCGATGGTATCATTGTAAGGAACGGAGTAA

>DS499600:888294-888695

AACAATCAAGACATCCTGAAGCTCATCACAAGCCGGATGATTCTGACCGGGGCATGGACCGCCACCACCGATTTGGGAATCAAAGTATCGGATAATCTCGGAGCGCTGACGGGCCCTGACGGCCTCGCTTCTCTACTTCCCTGGCCATCTCCTCCTTCCAAGCCGTCGGCGGCCGACCCTTCCACATCAGGCAATGTTGCGCCATCCAGCAGAATTAATCAGCACCATACGCTCCAGTCTCTGCTTGCTTCCACAGTCGCCGGCGAAGCCAAACGGGCCAGCATGGATTCTCGACGGGACGGCCCATTCGCCAAAGAAGCCCAGCGCTATTATCCCGGCGATTGGTACCGAGGCGGAAAGGTGGACGACATCTGCGTTTTGATCATTGTGGCCGTTGAAGA

>DS499600:896086-896487

GTTGTTCACTGTCAAACACGAAAAATGCCAAGAGAGGATGGGAGGGCCAAGCGAGAATCGCGGAAAGCCCAAGAACAGCCCTATCAGAATCTGGGCATCGGAGTCCGGAAACCTTACACATAGAGTCATGGTCCCATCATGTTGTGGGTTCTGTAGAAATTTTGAAGATTCTCGAAAATGAGTACCTCTCTAATACAGTGAATTTTGCGCTTCGCCTGAAAAACCTTTCTTTCCATCTGTGGCCCTTACTACCAATCAGCTGTGGTAATCCGTACGGTCAAACAAGTTCTATTCCTACCTACCTCGCTTTGTCTGCGTAGATACGGCACTTGTGCCACGTCACATGAACCTGGGTTACAATGGGTAGCTACCTTATTAGTGATACAGTACTCCATAAACTT

>DS499600:1025838-1026239

TCACGAAGGTTCTGTACATTGATGGGGACCGAAAAGTGTCTGCGGGATTGATTCCTATTCAACTCGACCCGCCGGACCGCCTTTTGCCGCCTTTCCCGTGCAGCGGTTGGGGGGCAAAGGAGCGAATTTCGGCAGTGGGAAACGGTCCCAGGGAATAATAGTACTGCACCTAATGATGGGCTCGAGATGGAATCCTATTCACCTCTTTGCGGAATACATACCGATCATTTTCATTGGACATAACTTTGCTCGAGATCCAAAAAAGACCCATGGCCCAGGGAGACTGACTATTCAGCACGTGAGCACTCTGCATACCTGGAGTATCCTTCGGGGACAGTTTGAAACCGAATGCGCCAATCTAAAGGCTTCAGAGAATATGCGTCTAGTAGAGAAGGATTTAC

>DS499600:1028462-1028863

CCAGAACTTCTCTCAAAACGGCATGATCTCCAAAGCAGTAAGATGGTCCGCAATGGATAGAGGTAAAGCTGGTCATTGGTCAAAGAATCAATGTGTTACTCAGGTAAAGGGAAAAAAGGGAAGGCGAGAAAGTTTAAACGGAAAAAATTCCGAGAGAGTGACGCAGCTGCCCACCCCGCTGTGGTGGGAGCCACTCCGACAGTCAGGCCTTACCCAAACTGGCCTCCTAATGACCAATGGCAACTACATTGATTCGCTCAGATCTCCCTCTGCCTCTCAAAATCCCCGTGACTGTCTGTGGCGGCCAGCCAAGTAACGTGACCGGGCCGAGGACACGACTCCGATGTGGGAAACAGCAATGCTGGTTGGATGTTACAGTGTGGCTCAGGAAAACACGGAAG

>DS499600:1040487-1040888

TGCGCATCTCTGTTGTGTGGATAACTGCCTATCGCATGAGTAGTGGAACCTGTGAAGCTCTGGAAATTTGCTCCTAATGGTTGGATTTTCAATATCTCCAGACTAGACCCAGAAGAATCGACGAAAATCGAAGGTGACGGTAACATCGGCCGTTTGCATCTGTGCAACTGTACAAATGCAGAAGGCCAGTCGGAGTTCTTTTGTTGGCTCAAGTCCAGCATTTAAGGTAGTACCCGGTACCGGTCACGGGCTTGGGTGCTCCTCGGGTCGAAGTAGTATCGGTCAGCTAATCAATTCCAACTACTCCCGACCGAGGAAGTGGGGAAATAATACCAAATCTACTGATGAATATCGGATGACTTGGAATTCATAGATGCCCCATATGATAGAATTTAAATATT

>DS499600:1051359-1051760

GGTTGGACAAAAAGGTTCCATCTGGCTGGCAATCGACCTGACGTTACAAAAAGAGGTGGCAGACGGAACAGGTGACCTTGATGGGCATCATAGTAAACTTGATCCGAAAGGGATATTCTGATAGGAAAGTGGCAGAAAACTAGCCATATTAAGTTCCTACTGATAAGTATTAGGCGTTCTTCACTTTTAACGAATGACTCCGCTGTTGCATTCGGCATCGGACAGAAACCCGGGGAAGAGTGCAACCCTAGTATGCAGTCTGGCTGTGGCGTTGCATACAGTTTCATTCACAAAAGTCGGGCTTGGCTGTAACAGGCTAAAATTACAAATACATTTGTTCTGTTATGTACTGTAAAGAATAGGTAATGTATATTACAACATGAAATTACTGTTTCTGATGA

>DS499600:1256226-1256627

AACTGATTGAGGCAAAAGATAAGTGGGAGAAATTGGCCATGGGTGCAAGGCCATTCCATGTGGTTTGCGATTCCATTGAAGCTCTGGAAGACCGGAGTCGCGATGGCGATATCCTTGGCGGTTGGTTTAGAGAGTCTTGGAAGAGTGTCTGACCGCCGTAGCAGCGTTGATATTGGACATGAGGGACCAAGGGCTGGGGGCGGAACTATGGTGGTCGCTTGGCTGGGGCTTGGCTGGGGCTTGGCTGGTAAGCTGGAGTGTAACCTCACGGCTTGGCTGAGCGAAAATAGACCAATGAACCGAGGTACAGGCTATCAGACTATCAGGGGTCTCGCAGCCTGCCAATGAAACATGAAATCATGGTGGTCGATGATTGTGATATCAACTAAGTGTGTAGGACG

>DS499600:1281571-1281972

GGATAAACGATTGTTGCTGGGGAAGGGAAGAAAGTGAGAGGACGGAGTGAAAGTACTTTGACTTGGCTGGTAAACACAGATGTCAACACGCGATTTACCCTTGAATCTAACCACGATAATGCTAACTACCCCTATTTCAAACACCGTCTCGGGATCCGCCCTGACGCCGTTTGGATCAGGCGGGTGGCGTCCCCCAGACGCGGGCCGACGTCGTACGAGCCTATAATAGGCACCAGGATATTAGCGGAGTATTGCGTTCTAAAATAATCAAATATTGATTAAGGTAAGGTGAGATCGTGATCGCTTGTCCCTGCATAGCAACTATCCTAGGATCCACCTTCCTCCCGAGTGGCCTGTTCCTAGACCAGCCATTCGGATCATGCCATCCCGGGTAACTTGAC

>DS499600:1500938-1501339

CATGCTGCCGTCAACGCCCGTGGACTACCGAAGCAGCGCGTCCCGCATCTCCTCCCGACCCTCCCGAAAGAATATACCAATGACCCTCGATTTCATGATACGTGGACAAAGCTTACGGCTTTCTCGCTGGTGGAGTATGAACGCGTGGTGCTACTGGATAGCGACATGCTGGTCATGCAGAACATGGACGAGCTGATGGATATGGAGCTGGATGCGCCGGAGTTGGAGGGGAGTGGGAGCCGGGTGTTTGCGGCTAGTCATGCGTGTGTCTGCAATCCGCTAAAGAAGCCGCATTATCCGAAGAACTGGTATGATCTATCTGTCTCAAGAGGTTCCAGTGACTGGCTGAGACCGAGACTAACGGAAACTTGTACAGGATCCCGGCCAACTGCGCGTTTACA

>DS499600:1511161-1511562

ATGTGCAGGTTTCTGATCCTGTTATTACCTTGCAGTATGTGTTCTAAACGTCGTCACTCTGTTGAGGAATCAAGAATCCGACGTGGTCGGCCAGGTCGATCGCGACCCGGTGCATCCGTAGTAGTCTAATCGGTGGGTCGCTTAAGTGATGCAAAAGCCCACTAGAAGATTCTTCTTGATAATGTGGAATCGTGGTCACCAGATGCTTCTGAATAATAGTCCCTCTGCTACTACTGTGCCGAGCTCCGAGAGCTTCTGAAAATGGTAGTGCTTGGACATGTCTCAATACTGACCAACTCTCCGCGCCCAGTGGTGGCGCCTTGCGCAGAACTATATTACCTATTAAAGCGCATCACTTGAACTACTGTTGTTGCACATGACCGAGCGCCATGAAAGGTCAT

>DS499600:1725466-1725867

CAATCCCGTTCGTGCAGGTAATCCCGGTGTGTTGCAGCGTCTCTATCGAAACAATCCACATCACGAAACAATTCTCCGAGAACGCATTGACCATCAATTGAACTCCAGATACGGCGCAGGAATGGGCCGTTCGGTTCCACGGGCAGACTCCGTAGCTCTCAAGCTGTACTGGGAACTGTTAAGGTTGTCGCCTGCCATGTAACGTGGCGTTGCCGGGGGATGGGTTCGCTTGTTCCCGACTGAAACTCGATCTAATATTGTTAACGTCGACCTGTCTCGAACCCATTTAATCACTCACAGACCCAGCCGGACGACGACGATTCTGGTCCCGATTGGTCTACCCGTTGAGATTCTCTTCGGACGCTGGGGTCGTTTCGTCCGCAGCAGCCGGGCCAAGGCCA

>DS499601:258488-258889

TCTTGGTTTCACTATATCCGCGCATTCCCGGAATCAGTAATCATGGATGCTATTCCCCGACAATCATTACTGCAAGTCGACCGCGGCCCATGCTCCGTAGAGTCATTGGCCTGCTCCGTGGAGTACGGAGGAATTGGCATACATCATCGCAATATCGTCCTCATTTGACGAGCCATTCCTGCCCATCGTGGCGCTGCCATGGCATTCTTACTAAGGCCGTGACCCAGTGGCGTCCGACAGAATCTTCTATGAACAATGAGCCCGCCGCGTCCAGCCGTGGATCCCGAAGTTCTTCCTGTACTTCACCCTGACTGGTCATTTCCTATCGGCTCTTCCAATCGTAGGCACGGACCAAGCAGACCCGAAGCTGACGGCTTCTGGGCTATATCCGTGCCGACTAT

>DS499601:390762-391163

AAAGTGAAATCAGGGCATGGCACGAGTGGACCCCCCTGTCTTGAAGTCTGCCTTGGTCCTTCCGCACCGAGGAATTATTATTGATTATTCAGCCCAAACACTCCATTAACTAGTTGCTCCGTCCAGCTAGATCATCCCACTCATGGAGATTAGTCGACTGACGCCTTGACTGATTAAGCTTAGCTTGGCGCAGGGACTGGGTCGGTGGAACCCTGGTGGATAAGGGGATTTCGGAGGTTGAGGCCTGTTCTGGTTAGGGGGGTTTTCGGTGGGTGGGTGGCTATGGGTCAGGTTGTCCTGCACTGTAGGGAAAAAAAAAATTGGTGACTGGTTCGCCGTTTGCCCTTCATTTCACGGGTTTGATGGATTGACATACCGTAGTCATGAGGAAGTAGTTTGTT

>DS499601:453889-454290

CCAGGTCCAAGGATGCGCCTCGTCAAAGCCACAAACATAGTTCGGTGCTGGTAAGCCTGCAATCTCGACTAAGGTTGCATGAGGATGGTCAGATGAAGCTTGCTCATGAACACATTCAGTAGCATGACCAATGAAGGGGTGCCTATTAGCTGATTGGCTTCATGAGAAGCAGCGCCGTCGCTCTTGTTTCCGAACTTCTCATAGGGAGATAAGGTGAAAAACACGCATCTGGAGGAGATGCACCGGAAGTACTTGCAGATATGCAGAACACCACCAAGTCTCCAGTATCAAGTACCTGCAGAATCAAGCAGTGTTGGAAAACCGCTTTATCATTGAGCGCTAATTTGCAGATCGGCTGCTCGCCTGCCTCAACTCCCAACCGATTAGTATACGCAGTGGAG

>DS499601:497372-497773

AGTAGAATGGCTGCACCGCCACAATATTAACTCAAAGCTAATCTCAAAGGAGAAGCTCTGGAAGGCTAAGTTAATAGTGCGCCACATTACAGTCCGCTAGTGGTACCTATGACTATTCCGTATGGGTCTCTGGCTTCGGAATTAAGGAGTATGGTAAAGTACTCTGTACGAAGTACGGCTTTATCTGTCAGGAGAACCATCCTCCAATTGCCACCCTGAATCGTATCGGATCCCGTCTCTTGCCAATCTGGCAGCTTCATACCTCAAGTATTGAGCGCTGTGGAAGTTCTTTACTGGAAATCAAATTCGCACTGGTTCTTGCGACTACTTCGGTTGATCGACGTGTCCGTGAGCGATGTGTGGCTTCGGCATTGTGGACACTGATATATATCCCATGGACC

>DS499601:498516-498917

TACAGTGTACTCTGTATATTCGAGGTAGGTCAAAGCATGCCTGGTAGCTTTGATCACTGTCCCATAGAATATATTTAGAACTTTAAATCACTAGACTGGTCTTTTTGTTCATTTGTAGACACTGCTACGTCGATTGATGCGGGGCAAATCTGGCACCATAGCCATCTCTCAGCATGAGGCATTAATTTACTTCAGGGCATACCACGTGTGATGAAATCGCAATCATACAAAAGCCAGCGGCACCTGAGTATTGCGGCTTGCGCCTGGATCTTACACTGGAATGAATCGAGAATTTGAACAGAATAACCGAAGACGAGACCATTCAACGTCCATGCGCCGGATTAACTTTGCAAGTTCAAGCTAATTATGCGGCTAACGTGTTGGACTGTTTTCTATTCCGA

>DS499601:546621-547022

ATGTTAGATATGCTTGCTAAGGAGGGCCTTTGATGACGCTTAGTTTGGCCATTGACGAGAAATCTTGATGCCTAGAGATCAGCAACTGGAAGCTGCTGGAATGATGCCCCCTTTTGTCCTGTTCTGGAAACTTGGCGATACCCGATTAGACTAGCGGTTTCTATGACACGACAGTGACATTGTGATATCATGTCAAACGTTGGTATAGGTACATATCTTGACGCCGTCCAATGATGATCAGCCTTCTGCACAGCTTTCTGAATCAGTGGTAGCGTCGAGAGTGGCGGTGCAGCTCTATTTTCGTAGAATGCGAGTGGCTTTGCTCGAATCTTGGCCTAGACTCAGAAGAACAGCAGATCAGGAGAACCAGAATTAGCCAATAAGAATGACAAAAGAAATGA

>DS499601:560822-561223

GTTTTTCATTGATGGAACTGCTTGATCCATTAATCTGGTAGTGATCCAGGCCAGACACTGTGAAACACTCAATGACAATTGATGCATCAGTTGAATGAATGGTGTTGTCTTGTCAAGTGATGGTAATGGTGGTGGTGGTGACCCTTTACGGAGTAGCATGATGATGATGATTGTGGTCCATCCACTATGAGCCTTTACCTTGTAAAAGTCAATACAGCCATAGGACCATCACAGTCCACAATCGACGGAGATTCTTGTGGATATTGGATGGAATCGGGCGAGACGGAAGCTGTGGAATTCAGAATTTAGTCTAGATACCCGGGTTGATGCGGGATAGATACCAATCCATAGAGAATTGCACTATTTCGAGTAGCACCATTTGGAAGTAATAGCACACTAGT

>DS499601:568139-568540

ATCTGCTTATAATAATCACGTAGTACCAGTATCTACACAAGAAGCAGTCATACCTATCACCATATTACCATATCTCTCTATGATATCTCTTTTGCATGGTCTAAAATCATCCTTCCCTCTGCCCCACCAAACGTCTCAGCTTCGATGAACTGGCGCTTTCGCCCCAGGTTATCGCGCCGTCAAGGGGTGCCAGCTCATTATTTATCCACTCTAGAGATCAACTGACAAACTCTCCTGGGAAGGTCACATTCCTTTCCCTGATCTCCTCCGCCATGGATTCTCATCCCCGGCCGCATAGTCCGGGCCCTGCAGGGCAGGTTTCCTGCATTCCTACCCACTCCTCCTCAACTCCAGCGGTTCTTCGCTGTTGTTGTGGTCGTAATGATTGTGCATTCCTCCAA

>DS499601:649641-650042

GGAAGTAGACATCAATTCACAGACTCAGAAACAAGGTAGGGACAGCTCACTTGCAGTCCAGAATAGTACCTCGTCACACATACGTCTAGAACAGCAATGGGGGCAAGCAAAATCAGCTAAACATCTAACTACTGTGGATGCCCCCTTAGTCAGGGGCCCTGGCCAGATGCCAGATCGTTGCCTTAGGTACGGAGTAGACAGCCTCAATTCACCTTTCCATACAGGGTCAGCATTGCGACACATGCACCCTTCTCACAGCCCGATTGGTCACAACTATTACCAACCTTCATGTGGTGCTCGCCACTATTCTACATTCTACCCGGGGTCATCTTTTCCGAGTCCTCTGCATATACCACTTTTGGCCACAGTCTCGACAGTCCAGCGGCAAACTTGTCATTTCA

>DS499601:823269-823670

AGGGGCCTGTGGATCAAGCCTAGTTGTCTAGCACTAGATGTGTAATAGAGGTAGTGGTACAAAGGACACGTTATTTAAGTATTTTTCTTCACTTTACTAGTCCACATAATAGGTGATCTTTAGCCACACTGGAAGTGGCATCCAGGGATCGACTGCCCTGAGGACGTTTCAGTGGTTAGCTATTGTCATGAGAGGTACAGTATGGAAGTAGCGGATTTGCTGATAACTACTCCCAACTAGCCACAAATGGAGAGAGGACCCATGGTGGTATGAGCACTTGAACACAAAATGGAGAGATAGCTGGATTCAACTGGGCTTTTTAAGGTACAGGTAATTGTACCTAACAATGCTAACAATGTAACCTATCAGCATGAATGTGACGCGATGTAGCACAGTCTACC

>DS499601:825756-826157

CTGGAACATAATTTTCAACTACTGCTGGCAAACGCAGTGGGTGCATACTAGGTTAATCACTCGCCCCCGGGGGCGGCTTTACTCTCGCCACAGAATGAAGATTTTCATCTCAGCTGATCAAACAAAGATCAACAGTTTCCTTACTACCTTTCCTACCCCTAGACTTGGTGGCCCACAGTGCTGTTGTTGTGATTCTTCTCATCATCCAATTTCTACAGGGCAGTATTGTCGCGCCATTGCAGCCAATGACGTCATCGCCTGGTCCGCAACTACAAATTCTCCTCCCAACGGCCGTCATCTCCATCGACATTCCCATCTACCATCCGCGCAATCATCCCTAGGGTCGATCAACCAGTACTATTGGCTGGGCTGAGATGCCATTGTCCTCAAGAGTACATACC

>DS499601:901271-901672

CGTTTGTGATTAGAGACTGCCGACTACCACCGTTGCACCATCAAGTGTCACACATACCACAATATGCGGACCTACCTACTGACCAGCCCCCATTAAGAAGCCTGTCAAGAAGCCACCATCAGGAATGCTCCAGAGCAAATAATCAACATTCAACAGTGTATGCCTCGCCCTGCTCGCAGAAAACAAAGTGGATTTCTCTTGTATATATCCAATTGTTTTCTACAGACGATTGGGAGATCTAAGGAGTGAAATGGAAGATGGTGACACAGTACTGAAACGGTAATGACAGAACAGAAACATCTGGTGGATTGGTTACGTATGGAGCCTCGAGAATGTAACTCTGATGGCGATCACCACGTCCGCCTACGACTACGTACGTCGTTAGGTTCACCACTATTTAC

>DS499601:921616-922017

TTCAAAGAATCCACGGTGACAGTTAGGAGGATTCTTTGTTATCCAGTCTCTTAAGCAGCATTCTGCAAGGAATCTTCGATCAAGTTGATCTAATAGGCAGACCTCAAGAGCAGCAGACGCCGCCACAGGCTGAGGCAAGATAGCTTCAGAACTGCAGTCATCCCACCCTTGCTTGATGCAGATTCTCTGAAGCATTCCACAATGGATTCTCCTCTGTGGCATTGAGCTCTGTGGAGCACCACGAGTGGGCGGTTATGTAGGCAATTTCCTGCGCTGGCCGCCTGTAGCCCAGTTAACGCTTGTATGCCCCTCCATCAGCCCCTCCTCCTACACCACCATTTACCATGAAGTCATTGGTTCAGAAGTGTGTATGCTTTTTCCTCAAGCTCGGGCGCAGGGAA

>DS499601:958498-958899

AGGCAACATCAACGCATGTCTGGAAAATCAAAGGCGGTTTATGAGACAACTCCCACTGCGTCTATTATTCGTACTCCAATCATTGATACGGAGCAGTTAAGGGGTGAGAAGGGGACGGGAGCAAGCAACACCCTATCATATGACACTGAAGCCCATATCACTGGAGACAGGGATGTGTCGTTGCGCGATTTTGTTCGGTTGAACTTGTTTTCGGCGGGAGACTGATGCCCGTTGCCGGTCGACTCCCGGTCGCGCGTCCAATCCCAAACCGTTGCGTTTCAAGTACGATCTCTACTCATCTTGTAGACACAGTGTGTCACTCGATCCGGTAATAGAAATTCAATGTTATGTCAATATAGCATATCACTAGTCATTAGGTTTACCAGTGAAAAAAACCACGA

>DS499601:960065-960466

AGTTTGGAAACCGCCAGCCAGGTGTATGGTTGGGGATATGGTTCTCGGGAAGCAAGCCACTGAAGTTATCAGCCTTCTTCCCTCGACCTTCTAGAAGCAAAGTAGCGACATCACCTGAGTATGATACAAGACATCAACACACAACAATTCCTTATCTGCGTTGTGTTTGGTCGCGTGGCCCTGTCCATATCAGGATCATGAGCTTACTCCGTACTATTGGTTTCAGATGTAAGGAATGATACCTATGTAAGTGTTATCTCTAGGCCTGCCAATGGAATTTTTTGATTGGTTAGTAGGGTATCGCGCGGAAGATGTGGTGCGGAGACTAATTCCTAATGAGAATTAGCGGGTTTCATTCTTTCGAAGTGATGACGGCTTGTCCGGTGAGCCCCACCCCTCCT

>DS499601:1100431-1100832

AGGGGTGATATCCACACTAGATGAGGAGATAGGTGGTGGAGAGGGTGTCCCTCTAAGGCATGCATCAAGATCAGGCGCGTAGACATCTGGAATTTGTTCCCATGCCTGGATAGCAAGATTGTCGACTATCTTACTACCATCAACAGGCCAGATACCACGATCTTTGAAGGCCTCACGGATAATTCGTTGGTTGAAGGCTTTCTCCCGTACAGGTCCAATCACCCGTAAGAATTCTGACTTCCCTACTGGCTCACCAGCCCAGTAAGATAGCTCATTATTTATACGTCGGAAGTGTTGCTTATAGCTCAAGAATGGCTTGCCATCCAGTGGCTGGCAAAGGTGTGTTGTATGAGGAAGGAATCCAAAGGGAATAACCCCATTATCTTCGCATGTCTGTAAGA

>DS499601:1236271-1236672

ATGATGAGCCCAGGTGCTTCTCCGCAGGATGACTATAATCGTTGACCAACTCCCCGTCAGCTTGGAATGTCTACAGTGTTCTTTGATCTTCCACCGCATGTCACACAGATCTATATCGGACCTTTTCCTGTGTATAAGTGGCAGGACATGTCTTCAACTTACAATACGCAGTCATTTGCATGCCACGAGCCAGGGGCAGTTAATACTCCTGATGGCGAGCCACGGTAAACTCCGGAACGCACGATATCCACTGATTCGATACCAGATCGGAGGCAGGTGACCCTAGAGAAAAACCCGCCAAAAGATCTCGCGACCGAGGGAGAGCTTATAGCAGTAACCGAATGACTATACTTAGGCCTATCTAACTATATGATCTCTTGATATGTATTATACAAACAATG

>DS499601:1452478-1452879

GCCATCAGATCGCCCGCCCAACAACCTAGCTTATGGCCCTGAGAGCGGTAATCGTTCTATACCCAGCTTTTGTTTGGTTCGTTGCAGATTTGACAGTTGGAGGGCATCAAAACCAACAGAACTTGCAATTTGATGATGAGATTGGTCTCCGTTTGACCTCGGGACCTCATGATCCCAGACATCTGATCATTATCAGTCCACCCTGTTGTCCTTCTCCGTCCCTCCCCATCCCGTCTGCTTTCATCATAGCAACACACGTCTCGGCCCTGCATTGTCCGAATCCAATCCGTTCGTTGCCCCAACCATCATCAGCTTGTGGACCCTGGGGTGAATGCAGTTGAACAGTTAGGCTCTCCTTGCCCTGCCTTTTTCCGTCTGCTCACTACTTTTTTTCGCCTTCA

>DS499601:1466395-1466796

AAATTGAATAATAGAATGATGATTGATAGGCCCGGTAGATGCCATTCGACAGAGATATACAACCAGAAGACTGTCCACGTGTCATAAGGAGTCATGGCATATACATCACCAGGCCAGCACCAAGTGACCAAGCATCCAGATAATGGCGTGATATCTCTGCATTATTGCGTCGACATGCGGGCCTCCCCGGTGCTGTACCAGCGTACCGGGAATCTTATTTTGGCGACTCCTCAATGCATATTCCGGGGTCGGACAATGTATGTGACTGAAAAGCAATATGCTCAGAATCTAGACCGGCGTCTCATGTCGATGGTAGGAAGATTTCCGCGCAGGGTGTCCGTTGCTGCCGTAGCGAGAATCGGCCCGTCACGAGACCACCTTCCGGTCGCTTCGTCCTTCAA

>DS499601:1715583-1715984

ATGAGCAATCCATGAGCAATCCATGAGCAATCCATGAGCAATCCATGAGCAATCCATGAGCAATCCATGAGCAATCCATGAGCAATCCATGAGCAATCCATGAGCAATCCATGAGCAATCCATGAGCAATCCATGAGCAATCCATGAGCAATCCATGAGCAATCCATGAGCAATCCATGAGCAATCCATGAGCAATCCATGAGCAATCCATGAGCAATCCATGAGCAATCCATGAGCAATCCATGAGCAATCCATGGACAACTCAGGAAGATTTAGAGTGTCTCTGCATAAGGTCATTTCGGGAACTGTCTGGCGACAATGGCTACAGAAAGCCTACAATTCCATTCCAAAAGCCATCCTGCTCTTAGTCTACTCCACGGATGAGCTTCTAACCACTTGTG

>DS499601:1775717-1776118

GAAAAGGACACGTCAAGTCAAGTCAAGTGATTTCATTCGCCGTCCCAGCCTATCATAATCAATGGCTACGGAGTATGTCTTGCTGAGAGTGATGTTACACGTAGTACTCCGTACACGCCCAAGAAGGCCAATGGGCCCACTCTTCAAAGGAGATGACATAACCCATAATATGGCTCAATAAGACCCACTTAATCTCGCCTGCCCCATTCAGACATGAGTTGAGCACTCTGGAATTAGAAATGAGCCTCGAATTTTACTGGCGCCGAAGACCGGATGGCGCCATTGCCAATGAATATTTTTCGTATGGAGCTTCAAGAGTAGACCACACCTTCAATCCACGTGACTCATTAGCGAGGTCACAGCCACGGTTCCCCTGCGCCACCCGCGCTAACAGATAGCTC

>DS499601:1806980-1807381

CGACTCCCAGAACACCTCAATCTGGGCCCTGTGAAGCAAAAGACGAAGGAATCAAAGTGTATCAAGGCTGAGGATGCTGGTTTACTAGCGAATTGGTATAATCAGCTTGCCAATGTGGTTAAAGATACACCACCACGATTGGTATACAACTTTGATGAATGTGGCTTCCGACCTGGCGAAGGCAAGGCAAGGAATGTGATTGGATTAAAAGGTTCTTGCCTTGATCTTGCTGAATCTGAGAAGGGTGAGAATATAACAACTATTGAATGTATTGCTGCAGATGGTTGGCAGATGGATCCATGGTTTATCTTTAAAGGCAAGCTCCTACTCTTTTAGACCATTCTTTTCTGACCTTTCTTGCTTCCTAAAGGCAACGGGATCTTCATGGAATGTTGGTTTAA

>DS499601:1815362-1815763

TTAAAGGCTTTGTGATGATTAGCTTGCAATCTGCAGGTGATCTGGTAGACTGCCGAGGTAGGTACCTAGGTCCTGAAGCTTCCTAATTTGCTACATTGGTGGTATACTAATTTTAGTGGTTAAAAATCGGTATTTTCTGCCGTGAAACCGCTAAGACTGCCACGACCCTAGGGACTACACTTGATCTTTTACTTAAACTGGAAGGAGAACTCATACCAACACGGCCTGGCGTGCCGGTCTCACCAACCTGTTAGTATACTATGAGTACAGCTCCACAACGCGGCCTCGTTGGGGTACGTTTACCCTTTTAGGTATACGCGGGCCGGTCGTTACGCCATTCGTAGATGAAATTTGTTAGCAGGTCATCCCAGCTGCATTTTCGACCTCTTGTGCCCCATGAA

>DS499602:88229-88630

CCATACGTGGGCAGTTTGCGATCATGCTCTGGTCCACAGTGAAATTATTTGCTTGTCAAGTGACCAAGAGCTCCTTAGAGTCGGAAATTAGCCTCCAAGCCAAGCTTGTCGAGCTGCGCCACCCTTCTTTTGTGTCTCAAGGCACAACTGTGACACACATTGCCTGTTGATATACTCCGTACACGGCATCGATAGTTACTTGTCAAGATTAGATAGTCCGAAGCGATGGGCCAAGACCAGTGTGGTTTTATCGTTTTCCGGCATCCCATGACATGGATCTGAACAATCGGCCGCTGGCACCGAAATGGCCCCCGATCGTCATGAGCCAGCCCACTCATGACAACAGCTCTTGGGTACACCATATGACATGGGCATCAGGGGCTTCATTGAATCTCTGGATA

>DS499602:281335-281736

TTACCAGATTCACCCCTCTTTTTTTTTTTTGTCTGTGACGGATACAGTACGTGACATAGCCTATAGGACATAGGTAGCCTGCTTCCAGGCGCAGATCATTCGCCTGGTGGGCCGATTTGTCCCGTAGCCTGTCGGGTCCTGGGTCAGCGAATCAGGGGTGCTTGGACCCGGGACACGCGGATCAGCAGGACCCGCTTTTTGAAACCTTGATTGGTAGGACCAGGTTATGTGCTTGGCTACCTGGCTGCCTTACATAGGCAGATAGGTAGGTAGTTGATGGTAGGCATCGCAGTGTGTGGTAATCACTCCATTTCATTCGGGTTGAGGTACGTAAGGTAGCTGCTGTGGATTGTGGGTGTGGATGTGCTGCGCCTCATGTGACTGACTGGATATGCGGACTC

>DS499602:345169-345570

CCGAAATGTCGGCGACACTTCGGTGGAATTCAATCCAATTGCATCACCTGTTTGTAGTCAACCCTCTGACGGCTGCCAGTTAGCTAATGCGAATGAGGCATTTAACACTGGCCGATCCCGAAAATGTGCAATTGCTGAATCAATCATCCGGCGAAGTGCCGGCCCTACCGGGATGTTGATTGCTCGCTGAACGCCACCAAAGACGGACACAATCCGAGACGCATCAGCCAAGAAAGGCTGCACTTGCCCTCGAAACGTACCCGAGAGTTTCATTCGTCGTGGTGGGTTCAAGATTCCGTGCCAAAAGGCTTAGTGTGGTGTCCCAGAAGATCAGACCACGAAAAGGAACAACCAAGGAACAATTCAGCCGTGGGGGGCTAAGGTCACGTGCCTACAAGCCA

>DS499602:491234-491635

CTGGGTCTCAGGCCACCACTTGCTGCCTTCCGCCAAGAAGCCCCAAGACCATCCAGTGAGGGAAATTGCCCCAAGGCCGCCAAGGCTCCCGCTCTTGGCGCTGTGGTTCCGGTTGCCCCACGACGTGCCCGGCTCAGCTAATCAGAACACTTATCCGGAAACGATTGGCCAATCGTTTGCTGTAATGCTTGGCGGCTTCTTGGCATTGCCCAGCCCAAGGTGCTCACGTGGGTGGGATGATGGGCATGTCGACTGTCGTGTGCGTCATCGGGCTGGGAGGCGGCAAATGCTCGCGCACCGACCCACGGAATTCGGCCAAGATTCTGGATCGATTGCCCCTTATGTATGCGAAGGTTTGGGGATGTGATTGGTGTGCAGCGTAAACGAGTACGGATTGATAA

>DS499602:496504-496905

CCCAGCCCGAGCGGCACAGCAATCCGCAGCCGAGCTAAGCTAGACTGGGGTCGGGGCCATCAGATCCCTGCTAAGCGCCGAACTATGGGGCATATCGGTAGCCAACACCACTTTACTATACTGTCACGCGAGTCATAAATCGCAGATGGCCCAGTTCCAGCCCTGACAAGAGTGTCACACTTGTTCTATTACGGAATGGGCCGCTTCCCGATGTAACCGCACAAGCCGCTATCTTCCCTGGAATGTTGGCACAAGGCTACCTATTCAGGCGCTCAAGAATGCCGATGCCTGAATTCGCAGTTGACTGGCAAGTTTCATGAGCGATGTGGCTTCACTTTTGGCCAGAAACATTGGCTGTTCCATCTCAGCGAGCAGGGTTTCAAACTTGGATGCCTTGGTGC

>DS499602:562534-562935

GTCTCCATTATTATCAGCGAGGTCCGGCGGAAGCTCTGGAGCCCTAACTATCCTTCGGTCGTCCTGGATGAGGCTAAAAAGCTACATTCTCACATCGTCTGACCTTCAACGTCCGGCACAGAGCTCTTGCAGGGGTTTCAACTCATCATTAGCCACGTATTACACCGAAGTGGAGTATTCGGAGCTCAGCTCGACCGAACATCGACAATGCCAAGCCCGAATATGCGGTCCGAAATTTAGGTCGCCAGACAGTGTGCTACCCTATGATGCAACCCATGCTGATAGTGGGACGATTTCGGCCAGCTTGAGATTTATAATACAATCAAGGGATCTGCCGAGGTTGCTGAGCACTTGACAAGTCAGCGAAGAGCAGTTGACCTTGCTTATGCAGGATACGGCTA

>DS499602:573536-573937

TACAATTGACACCTGTAGGCAAAAGCAGGTCTTGATCGATAAATATACCCATCCAAGTCTATCGGATGATGCCATTCCTTCATCCCCTTATATGTACTGATCCGTGCTCGCAATGACATCAACGCAGTTTCCCCTAGACCACGCTAAATACCATGATTGTTTAAACACAAGCTGATTGGCCATTCTTGAATCAATCATGGCTATAAAATGGAAACCCCCGCAAAAAGCCCTCTCCTTTCCTCCGCAGCTTCTCCTCTTTGGGAATAGGGTCGTATGATGATCAGGCCACTGTAACGCCACTTCCGCAGCATCACCTTATCCACCTCAGGCACTGCCTCAATCCCGATCCCCTGACTGAAGTGGCATATTAGCCTTTCTATCCCTATATCTGTCCTTCAGTT

>DS499602:576646-577047

TGCAGCATGGCGGAGAATTCATGCGCAACGCAGCAGGTAGTCACGCAGTCTCAAGCAATAACTCTGGATGCACATCCTCATCATACCACTCCCCCAAACCTCGCTCCACAGCTGGTCGCGCCGCAATGCGATCCACCCACTGTTGTAAATGAGGTAACGGAGCCAACTCATTCTCCGAGAGTCCAATGCGTCGAATATTTCGCACCCAGGCCCAGGCATTGATATCAGCAATGCTGTATTTGCCCTGACCCTGCCCAGCCAGATACTCCCGTGTGCCGCCACCATATCTCCCTGAGAGACGGATTTCAAGTACGTCGTACACCTGCAGCAGTTCTCGCTTGCAGCGGTCTACTGCCGCTAGTTTTTCTCAGTAAGCACGCATAGAAACCCCAAGGCAACAC

>DS499602:577755-578156

GGTGCACTGAGATAAATACAAGCAAAAAAGCGATGTATTATTGCTTAGCTGCAATCAGAGGATCAACAGCGTCTCCTACTTTGTTTGGTTGATTGCGATTGCGTGATAGATGCATTGTATGAGAACGGATTGGATACTTGCTACATCTAAATCGCACTACGTGTCCCCCGGTACATATCTGCTTTGAAATCACTATTAGTCACTAAGATAAGCCTCCACATCGTATCATTCAATCCCCCTACTAGCCCGAGCTATGGCTTTACAGTTTCATATATCTAGTCTAGAGGCTTCTATGAAGCGAATGTGCTTGTGATTTCGCTGTCAATTGAACGGATGTCACCCCAATGGTGGTAGTCATTTAGAATGGCGCTTGATTCTCCGAAGCCAGAAACCTCTTATAT

>DS499602:660460-660861

AATGAGGGACCTCGCACTGCGACACTGTACAACTCTGGAATGGGAGACCACAGAGTGGTAGAGTGGCGCCTGGACATGGTACCATAAGGTAAAACCCCGGATCGAATGCTCGAGCTGACCACCAAAACCCGGCCTGTGCTGGTAGAAGCTAAACCATCTAGAGCCAAGTATCTTGCTTAGACCTCTCTCCTGGAATGCCTCATTGGTCATGGATTTGTGGCGCGGCCATCCCCGGACGTTCTGGCCAAACAGATGCTCGGCTCTCGGCCGGCCTTGTTGTCGGGCTGACTAGTTCTGGTACAGTCTCGAAGCTGTAGCCAGTAAGGGAACTAGCTACGCGCTTCCAGAGACACTAGCCCCGGCGACGGTTCCTTCTCGGGCCTAATTTCCGTGTTGTACAC

>DS499602:765869-766270

GCTACAGAAGGTCTGGTCCTAGTGCAGATCAAAGGACATTCCAGCTCAGTCGAGTTATTGAGATTCAGTCATCGATCATCACTTTACTCTGTGGAGAAAATCATGGCAAAATCCGAGGTTCTCCGATGCGGGAGACCAGCACTTCCATCCAAATACGTACGGAGTATTGCCACCATCGCACTGAAATGGACTCGCTAGCCAATCATCCGAGTGGATTCTGGAGACAGAATATTCTACCTGTCACAACGATCCCATGCCCCATAAACGGCTGATCGTTGCCGCACGATCACGCGTTGGCAGCTACTGAGAGCATTGATCTCATGGTCCAATTTCCAATCATAAAAACCGATAAATCATCATTATAATCGACTGTAGATCCTTTTGTCTGGGCAGTCAATGGA

>DS499602:770996-771397

TTGGAATTCTAAGAAAACGACTACAGAGTACGTACGCAAGACGGTGCCACTAACCGCCGGTCGCACCCCTTTTAGCCTCCAACTTTAGGCGAGAACTGCTGGAGCGGGCGTCCCACGGGCTAAGCTCCTCTGAGACAAAAAGGGGGGCGCTAGCATAGTAGTTGCCCACTCACTCCACTCTGGGTCTGGACCCTGGTCACTTCACGTGGCAGACATCGGCTCCGCTTGCAGGTGTCGCTCGGGCCTTCCCGAGTTTGGCACAAAAGCCGATGCAAGGCTTGTCATGGCGCCTTCCTCCTGACAATCCTGGTCAGTGGTCTGTTCTATCCATGTAACTCATATAATACAGAGTTCAGGCTCCTAGTACGGAGTATCTTACGTCTCACACAACGGTTGGTTTG

>DS499602:773047-773448

AAGGGAGATCCTAAGTTTTGCGCTAGAATGACCAGAAATCAGGTTTCATTTCGCTTCTGGGGTCCTTATATGCCATGGTAATTTGGGATTCTTCGAATTGAAAAGTATATTAGGCATTCGTGTTTCTGACTGGCAAATGGTATGTCAGTACCACTCATCTTCCAACTTTAACTTACGATGTAACGTCATGGCCCCCCGTCATGAAAACTGATCGTATCGACTACTGAAACAGGTATAGTCTACCTCTGCAAGACCACCAGATCTTCGGGCGTGGCATTTCCGCACCTTACCCCAAAAGGCTTAGTGATGTGCAGTAGAAGGTTTGTGGCCGAGGACTACCCTAGGAACGGTTATCAGGCTACCAGTGATTTACCTACTGCAATTAGAAGGCCAAACGACAG

>DS499602:848985-849386

GGTGATATCCACACTAGATGAGGAGATAGGTGGTGGAGAGGGTGTCCCTCTAAGGCATGCATCAAGATCAGGCGCGTAGACATCTGGAATTTGTTCCCATGCCTGGATAGCAAGATTGTCGACTATCTTACTACCATCAACAGGCCAGATACCACGATCTTTGAAGGCCTCACGGATAATTCGTTGGTTGAAGGCTTTCTCCCGTACAGGTCCAATCACCCGTAAGAATTCTGACTTCCCTACTGGCTCACCAGCCCAGTAAGATAGCTCATTATTTATACGTCGGAAGTGTTGCTTATAGCTCAAGAATGGCTTGCCATCCAGTGGCTGGCAAAGGTGTGTTGTATGAGGAAGGAATCCAAAGGGAATAACCCCATTATCTTCGCATGTCTGTAAGAAAT

>DS499602:872213-872614

CACATGTATCCCCCCCTCTTATCTCTGTCACTGACCGCCAGTCTCGGTCTTCCAGTAAGAACGGTTCTTGGGTCAAATCCATGAACAATTGACACGCCACTGCCCACATACCGCCGACTTGCATGTTATGGCCTGCTGTCCGCGCGCCGCTGCCCGGCCCGTGGGACGTGCCGTTAGCTCCACGGCGCAGCCCATTTCTCGTCAATTGGGAGTTATCCACTTCTGCAGGCGTTCGAATTCGTCGATGCCGCTTACCATGTCTGGTACTTGTTCCATGGACGCAGGAGCAACGATTTCTGCATCAATGCCCGAATTCACAACGCAAAAGTCTTGTACAAGCCGTGGTGTCATCTTCAGGGCCAAGCATTCGCTTTCTGGTGCCTGACACACAGTCAACTAAA

>DS499602:875070-875471

GTTCGCAATACGGACAGGGGAATCGATTGACTTGGTTGGGCTGCGCAACGGGGTGCTGTATCCCGAACCTCGCGCCTAAGACAGCAAGTTAGTTACGAACCATGGAATGGCAAGATCTTGCCAGAGTCACTAGCACAAAGCGACTCAGTTACTGGGCAAGCGGCCGTTAGTGAGGTGGAGACTGCATCCTGGAAAGCCCGGAGACCAAGGCCACAGTGACGGAGTGACTAGTGACCCCTGACCACTGACCACCCAGAGCTGGAGCCTGGAGGCCTGGAAAGGGGGCCAAGGACAGCCCACTCAGCCCGATAATGCGTAGCCCACCAGCTGACGGTGGGTTTTGGCGGCAACGGAAAGACGCGGACGGCAATGCACCGCGCCGATCGGGTTCAAAAAAAAAA

>DS499602:876512-876913

CAAATGGCATCAGCAAGGTGAGACCGAATAGGCACAATAGGTCGGAGTCGAACCCTCAGGATTAATTTCAAGCTAGGGCCTTAATATATTCCGGGTCACACGATCCATCCCTGATCTACTATGTGGGTGTGTACCTCTGTGTGACAAGATTGCAAATGTCCTGATTGGCTTCGTAAGCATCATAAGTACCTTTTTCGGTTAGAGAAACGGTTCATTATAAATTATCCAAACAGGGAAGCGGGGACACAGAACCGTTTGCCAATTTAGGATAGCAACTGTGATAACCTTACAGATACCTGCAGTGACTTGCAAACGGTCATGAGTCCAGAGCGCTGTCTCTGTTGGCTGATCAGGCTGTTCCAAGCCTGACGGAATAATGATAGTAATTGCTTTCTCTTCAC

>DS499602:880901-881302

CCGGCGTCTCCTCTAGCCAGGTAGCCAGCCTACCAGCCTACCAGCCTAGTACTTACGGTAGTGCCACTGGATTCATGCACTAGGCCATTTAGTTTGGTTTCCCAAGTTCCCAACTCCGCAGGTCAAACTTTATGTTTGCCCCCTCTTCAGACTCGAGCCTATGCCAAACTCGCTACCTGAGTGGCTGGCGGGACTGCCAAACGCCCAACCCGAAGGCTTACAGCAATTCAGCTGATCCCCATGGAACCATGAATACTTTAGCTGCGTCTTAGGGGAGTCCCCGTCCATGTCTATGGGTCACTGGCGCGTTGATTCGGACGGCCCACTGTGTCTTGCGCACCGCACCGTCCGTCTCTCATTGGGTTTCCCTGTTCCCTTAGTACCTTACCTCCTAGTATCTA

>DS499602:896223-896624

TAATCTGATATCTACAATCAATTGCCAGTTTGCCATCAATAGAAATTTCCTTATGACTTCACTGACCCAGAAGTTGCCAACCGATTTGTCAACTTGTCTGGTCTAGATAGCAGCCAGAAGTGGTGCATCCGCCGATGAAGGGATCCGTGGTGGGGTTCTCCCAGTCTCGGGCGGTCTTCTTGATAATACGCCCCAAGCCAGCAACTCAGCGATTATTCCGTTCGTCCCCCAATCGATGAGTTTCGGTATCCTTCTGCTTTGGATCACCTTATGGATGTCTCCAGCGGCCCGTTGGCACATCATTTGCCGGCTTTGTCAGGGGCTGCTCCAACCGTCCGCGGACCAGATGGTGTATATATTGACCGCCATGGACCCCTCACCCGCCTCATCTTCTTTTCTCT

>DS499602:912678-913079

TGCCTATTAGAAATTTGTCTGTGCTCTGAACTATTTCCTCTAGACTTAAAGTGTACGGTGATTGGAGGTGTGCTGGCCTGCAGATGGCACCTGTTAGAACCTAACCCGATGGAGACAACTAGGAACCTGGAGAGGCAGAGGCACATGAGATGATCTGTCGCGGACTCTCGGGAATTGTAGGAGTCCGAACTACAAGGGCCAGTCCATGCTCTAAAACTGGAGATGATGAATGTAAGGCAGGGACTAGGGAGAGATTACGGAGTACACGTTGCCGCACCGCCTCCTGGAGACCAAGAACTACATTCGGAATGCTTCATTGTTTACTCAGATGTGCGCGAGGATCACCGGGGCATCTACAAGGATTGACATTCATGCTGCTCTCGGAGGAGGAAACTCTGGCA

>DS499602:946299-946700

ATATGGTGGTGACAATGGAAGACACTCTGGTCAGACACTTCCTCACTAGTCTCTAGGCACTTTGTACGGAGTAGCAGCCTCCATAATACAGATTTAAGTGTTTTTGCCGAGCGAAAGTAGTCAGGGAATTGGATGTTCCACTATCGAGTCGAATCCGAATCCTTGCAGCAACAAAACACTCTCGTGACCTGACAGTCGAAACTGGTAATATTACGATACAGTGCGAACTTGTGAGTTGGCCTCCAAATATAATCTTCCAAGGATTGTCCAATAATCAGCTGTGCCAAGACTATTATTCTCCACCGCTGGGCCTGGACAAAGTATAATCGACTAGCTTGTTACGTTTGCACTGAGTATTATTGTAAGGATCCGCTCATGAATGCTTCGATGTCAGCCAGATT

>DS499602:983347-983748

GGGAGCAAATACAGCCCAGGTGGTTGGTTGGCTGACCACCAATAACTTTGGGTCCGTTGTGAAGGAGCTGCGGAATCAACCCGGATAGGAGTCTTCATTACCCTCACTCCTGAGTCCGTACTGGCCGGCCCGTCAAGAGCAGCCAGTCATGCAGTTTGTGGCACACCAGGTTTAGTTAGCAGCGAGGTTTATCCCATGGGGCCTATAGTGATGATTCATTTCGCCTTAATGTAGCATAGCTAAGAAACGTGGAGGAACTCTTTCGAATCCTGTCATTCACCTAGCCGATATCGTGATATTCATGATTTTCTGACGACACCCTTGCCAGCAAACGTTCATAGACTAGCGGTAGTTGAAATCATAAGTTCATAATCATGTTCATTATAATGGTCATAAGGATA

>DS499602:1029351-1029752

CACACAACTGACTGCGGCAGCCCACCCGATCTCCCTCGACAACGCCCCCCGACTCCGACAGATTGGTGCTCCCGGCAGTTACTCATGGGGCTTAGCCTCATGGGCCACCCGTATTCCTATCCTATCTACTATCTCGGTTCAGCTGAACTCTAATGGGTTTGCTCCCAATCCACAGAACGAACAATCCGCTTCTCATGCCCATCATTACTGAGTTAATAAATAAGGACTCGGTCAACGTATGGGTGTCCGTTCAGACTAACTGTATTCATCCTAGGCACCGGCCAAAGAGGCTAAGAGGCTAGTCTAGAGCTCGAGCTAAGAGGCCCGAGACCAGACTCTCCAACTCTGATTCGCTCAGCGCTCGGCTGCGTCTTTGTGAACCATGGATTCCTGGCCTGTGG

>DS499602:1083581-1083982

CTAACAACGAGATGTCTCCTACGGGATTTGATTGGATATTATTTGTCACACGTCCTAGTGGGAGGAAAGCCACATCACAAGCCGATTAAACCGACCCTTGATCTATCAACAGGCGCTAATATGATTTTGTTTTGTTTGTGAGGGGTTGTTCTCCAGCTCATCATATTTAAACTTTCCAACTGCATCCTACTGCTGCCTATTGCCTCCTATTGCCTCCTATTGCCTCCTATTGCCTCCTATTGCCTCCTATTGCCTCCTATTGCCTCCTATTGCTTCCTATTGCTTCCCTTTGCCCTCTCGCCCTATCTGTTAGACTAGAAAATGAGAACCAAAACCTTGCAGAGTATTCAAGATATGGTAGAACAATTGAACCGACGGCAGTCGATGCTGGGTGACTACGA

>DS499602:1085503-1085904

TTTTCGGTGTCTCTCCTGTTTGGAAACTTGCGAACCCCTCATGGAGGGGTAAGACGTCACGGTCGGGCCGAAAGTCTCTTTCCCTACATGTGGCTGACCAACGCCACACCTGTTTTCGCCACGACTTCTCCGAGTGGTCCTCCTTATCTCTTCCTATTGGCTCCCCGTTACACTATTGTACCTTATTGCTCCCTATTGCTCCCTATTTCTCCCTATTTCTCCCTATTGCTCCCTATTGCTCCCTATTGCTCCCTATTGCTCTCTATTCCCTATTGCTCCCTATTGCTCCCTATTGCTCCGTATTGCTACCTATTGCTGATCTTCCGGGAAATCCCAGGAAACGGCGCATGTCTGTATTTTATCGGATCTAGATCCGCCGAACCGCTCATTTTCTGACAAGT

>DS499602:1097501-1097902

CTTGGTCATTTGGTTTTGTCGCCTTGGCACTGGGCTTTTCTTTTTGAGGTTGAGGCAGGAAGGGGGAGAGAGTTTCTAGCACTGTTTAATGACGAAGTACCTACCGTAATTGGGATCTGGTAAATTACCCAATCGACCTTTGGGCAGGGAGTGCATTTCCGGCAAGGATGAGATAGCGCTGTATTTTTGGCAGGGATTGAGAGAGAGATGTAAATTGGCAGGGATGGGAGAGTCTCGTATTGTTGGAGGGATGAGGTATTATTGCCACTGTAACTTTCCCCTAAACATGTAACTTACTGACCTTTTACAAGATGTTTTACGGGGTATCATTCGGGGAGGAGGGAGAACAGTTTCACAAATACACCTAGCGTTTCATGATTCCCCTAGAGCCTCTTTCTGAT

>DS499602:1158125-1158526

CAGCCATGGAACTGCAATGTCAATCTGACCAGTTTCCATCTCTGTTCCGCCAGAGCCAATTCCTATTGGAACTCATTTTCGTCACCCGGGAGATCAGAAACTCAACATACGAACAACCGGGAATCGGCAACTGCCTGTGTTCTGCATGTCTCAGACTTGCAGCGTTGAGGTTCTCGGCAGAGTCGAGATTGGTTGGTCTCCGCACACTCGTTGACTCTCGTCTGCAAGCCAAGCCTCGCCGATCAGTCTATTGCATCATGGGATCACTATAAATATCAATCTCGTGAGACAAACAGGCCGTCAAGCGTTGCGGGTTGCACGGAGCTGGATCTGCCAGCCCGTGTCTTACAACTCATCAAATAACAAATTGCAAACGCGGGGGGCCCTGTTACTCCACAATT

>DS499602:1351487-1351888

CTTTCCTACCATCAAAGAGGCTTGCCTCCGTGGCAGGTGACATTGTAGTCCGCTTTTCCCCGAGTCACTATGCACAGTACCAATCGATAACAGGCGAAAACTTGCCTAGATCCCGATAGTGGTGAATGAACAAGGTTTCTGTTAAGTGATTCGAACTATGCACTCGCACTAGTCTCAGAAGACCCAATATACTCTTACCTTGGCAGGTTCGAACATTAGCTCACTAACAAGCCCACTCCAAACCCGGGTGCCTGACACTATTACTTGGTCCTTTGGGGGTGATATAACCGGGCCGCGCCGCCGACAACATGGCGCTATGACCAATTGAAACCAGAATCGATTGAAAAGGCCAAGGCGACTAAGCACTAGGTCAAGATCGATCACAGGTCCATGGGCTTGCC

>DS499602:1448073-1448474

TCGATTTGACCGACTTCAAACATGTGGTCAGGAGTACGGATCGAGTTTGGTACTTATGTTCGATGTTGCCTGTATCGTTATGTCCCCTTGGCAGTCACACAACCCACCCTAGCTGATAAATCCCTTTCTGGTGAGGCGTTTAGGTGGTCAAGACCGCCTGATCCATCTGCAAATGAATTAAAGAATAAAGAAAGGAGACTCAAACAGGATTAGCGCCTCCTTCACTTCCAAGATTTCGTGCGTCGGTTAGAAGCGTTGTACTACAGCACAATTTTCATGCTACTCATGCCGAATTCTACATCTTACTGGCGTTGTTTGATTTGCCTGAACCAAAACAAAAATGACTCACGAGTGCCAACAAGGCCTGTTCCTAGCGTTTACCCAACCCTTTATACAGAACC

>DS499602:1463063-1463464

TCTGACCAGGAAGTCTCCTATTGGTTCAATGGACTCCATGCTGCCTTTCGGATTATTATTTTATATTCTTGATTTCTTTGACTTGGCATCTCGGGTCAATGTATGTATGTATGTATGGTATGTATGGTATGTATGGTATCTATGGTATCTATGGTATCTATGGTATCTATGGTATCTATGGTATCTATGGTATCTATGGTATCTATGGTATCTATGGTATTTATGGTATCTATAGTATTTTTGTTGGGGCTTGGCACCTTGTATATTTGGTATCCTTGACATTTATCATATGACATCAGCAAAGCTTTTTCGGTAACGTAATCGGTAAGCGAGCGGATCACGCAACCGAGCGGATCACCATACCATAGAATTTGGCCGCGGCCGAAATTGGAGGCTATTTT

>DS499602:1469067-1469468

TGGATCTTTGGTATCTTTGGTAGCGTCTTTTTCCTTTGGAGCTTGGCATCATGTATTTTTGGTAGCTCTTTTGGGGGTGACATCATGTGACTTTGGTATCTTTGGTATCTTTGGTATCTTTGGTATCTTTGGTATCTTTGGTATCTTTGGTATCTTTGGTATCTTTGGTATCTTTGGTATCTTTGGTATCTTTGGTATCTTTGGTATCTTTGGTATCTTTGGTATCTTTGGTATCTTTGGTATCTTTGGTATCTTTTGACATTCACATCTTTGCTTTTTTTACCTTTGGAATCCGATTGGCGTCGCGTATCTTTAGTGTGTGCAACATTGATTTAGCATTGGTGCCTGATTGTAGACACACAAAGACTGTAGAGCTCTTTCCATATTTGGCTAGTATACAT

>DS499602:1473063-1473464

TTCGTGCTCTGCCCAGCACCAGTTCCATCCTTCGCTATGGTTTATAACATAATAGTGTGTATCTTATGTACCTTCGGTATCTTTGGTATCTTTGGTATCTTTGGTATCTTTGGTATCTTTGGTATCTTTGGTATCTTTGGTATCTTTGGTATCTTTGGTATCTTTGGTATCTTTGGTATCTTTGGTATCTTTGGTATCTTTGGTATCTTTGATATCTTTGGTATCTTTGGTATCTTTGGTATCTTTGGTATCTTTGGTATCTTTATGGCTTGGCATTCTGGCGTTGGTTGTCTTGTATTATTGGTGCCTTTGCTATCCCGCCAGAGCTTCGGGAATTCTGGGTTGCAGTTCATTGCTTGGCCTCTGAGAGCCCATTTAGCTACAGCCTACTTGGTATCCAG

>DS499602:1485990-1486391

GTGTTCTCGTTGCCCAGGCATCTCACACAGAGTTCACTGCACTAAATCCGATACAGCCACCAGTAACGACGGCCATACTGAGTGTAAATTACAACAATACTATGATGGCTTTCTGCATGTCAAGCCCCTTGTATTTTTGGTCTAATCGTATGGAACACCCATGCCTCGTAATTGGTCATTGGCAAGTTACGCTTGCCGTCGATTCTCTTAATCTGGTTCATCAATTTAAAGTGGATGTAAATATTACACAAACATGGTATGATATAGTAAAAGTAAGGAGTAAGAGTGTACGAGAAACGATTTTCGGAGATAAATTACGAGCCTACGAGTCTCGGCGTCATTAAATCATGTCTGTAAAATAAGCCAGCATTACAGTCACTTTGGCTACAGGAATATTCCTG

>DS499602:1521505-1521906

CTGGTGGGTTCAGGGTTCAGCTTAGCTGCCGCAAGGGACGGAGATCTCTTTTCGGCCGATAGAAATGACTATAGTCATGCTGTGTTCTAAGAAGCTTGTCTCTTATACATTAACCAATAATGTCAGGGTTAATATGGAGATGCCTTGATAATCGTACGACCTCGGACGGAAGTCCAATTGGATGTGAACATGCATACTACTGGCGTTGCGCTCAGTTGCCCTTAAGTGGCATGACGTCAACGAGCTTTCGTACCATTTTTTCGGGGATACTACTGGCGTTGCGCTCAGTTGCCCTTAAGTGGCATGACGTCAACGAGCTTTCGTACCATTTTTTCGGGGAGAACCAGTCTAACCCCGAGAGACGTACTGTCTGTCCATGAGAATTGAGTCGTCTAACATAT

>DS499602:1531119-1531520

CAACCCTGGTGTGAAACAACGCTAACCCTATTTTGGAATGGAAGGAACAATATTTCTCCGACTAAGCAAAATATTGTAATGACATGTAAAGTTACATATATAACGTGACCTAATAATATAGCTACTGATAAGTATTAGGCGTTCTTCACTTTTAATGAATGACTCCGCTGTTGCATTCGGCATCGGACAGAAACCCGGGGAAGAGTGCAACCCTAGTATGCAGTCTGGCTGTGGCGTTACATACAGTTTCATTCACAAAAGTCGGGCTTGGCTGTAACAGGCTAAAATTACAAATACATTTGTTCTGTTATGTACTGTAAAGAATAGGTAATGTATATTACAACATGAAATTACTGTTTCTGATGACTGTACAATTTTGTACAAGTACAGTGTGCGGAGGC

>DS499602:1562894-1563295

GCTGGTAGTTAGTTAATGGTTCGAGGATACTCCCCAATGACGCCAAGCATTTTGACGTGTTTCTATCCCTCCCTGCTTCTCTCCTGCGGAAATCTTACACACAGTACATAGTACACTCACCAGCGTCATTTCAACTAGCAAGATGATCTAGCCCAGAAAAGGAAAGAGGCGGCTTGCATAGCGCCTCGACCCAAGTGGGCCCGAGAGTTCATCGTAGTTGATCACCAATTGACCATAGTTCACCAGTTCTATTCTCAGTTGGCTAGCTGCACCGGAAAAAAATGGAGGAGGCTTGCTTAGCGCCTTAAGGGAAGTGGACCGAACGGTTGATCGCAGTTGGCCCAGTTAAACGCCGATTTGTGTGTACCATTCTTGGTGGGGCGATCTATTGAGCGAAACCA

>DS499603:182664-183065

TCTGGAAGCGACCATGGAAAGAGGTGTGAGCGCTCAGGCAGACCAGCAGATGCTGGACCCCCTCCCAATAGGAATATGAAAATCGAAGATTAGGCCGATAAAGGCCATTGTCAAGTGCCCTGACTCCTTGCAAATCAATGCAAATACGATATTCTCCGACCAGGCAAGCAGCGCGTGAACCAGCAGGATGGAAGCGGGCCAACTCGGAAACCGATATTACATCCCACGCCTCGGCGAGTTGCCTGATTTGAATTCTGGTACAACGTGGTTAAGGTTAATTTGACTGGGAGAGACTCTTTACGACTATGCTTGAACGACTTGTCATATGCTCAGTAGAAATGTTTACATTCGTCACTTCTCTTCAACCTCTCTTCATCTTCATCGACAGTTTTCAAATCAGC

>DS499603:213849-214250

GGTCCCCAGTAGGTAAGTACCTACCTAAGGTAGGCAGCAGGTAGTCCCAACCAATGGAAGCCGACCCAGGGTCTATTAGGGCCATTAGATTGGTTAATGCACACAATCCTGGCGACCATTGGGCAGCAATACGAGATCTTCCCTGATTTGCTCAAGAGAATCTGCTAGAACCCAGCAAATAATGTACGAATTAGCTGCCAAATTTCCTTTTCGCCCAGGACTTAGCACGTTCGCCAAATCGGAGGACGATGTGGCAGAGTCAGCCAAGACCTCTTGAATGTAAGTATTCCACCCGTCGCTTTGTGGGATGGCAGCCACATGGAGTTTCATTCGCCACTTGAAATTTACTCATTCGTTCTCAGTAGCTGTGGAGCCGCTATGATTGGCGAGGACTCACAGAC

>DS499603:380719-381120

CACCAATTGCATTGCAGTATGACGATGGCGTAGGTAGTATCCATTATCCAACATAAACAACTAAAACACCACTGATAAGTCGATGGTGATAAGGGAGAATGTCTATAATAGGCCTGGCCTTTGTCTTTGCCAATGTCTCGCCCTAAGCTTGGTCGCTACAAGCATGAGTGGCCGAGGCTTGCCAGGGCTTGCCGTAGCTTGCTGTAGCTTGCCTCCGCTAGATGAGGCCCGATATTAACTAGCTTGGCATTAGACAATGGCAATACGTTGGCATGATATCTCCGTAAGACTTGCTTGTATTTGCTATTATGCCTTCTTGGGGCCCAATTGCGCCTTCTTGGCAAGCAGGGGGATCACATCTTGGAGGGTTCATCTTGTCCGCACTCCGCGCAGGATCGAGA

>DS499603:388975-389376

CACTACCATAGTGTGACAGTAGTCCAACTAGCCAAATGATCCACGGTCCAGTCACCGAGCCGGCACGGATGCCGGGCCTTAAGGGTCTCTCTGCATGCCACTCAGTGGTGTGATCCCGGATCTCTGATTGCGAGGACCCATCCCGTCCCTGGCGCGTCAGCGGCGTGAGATTTACCTCCGCCGTGGCTGTTAAGGTTGAGCCCGCGGGTTCGATCAGAGTCGTGGATTTCGGATTCCGTGGGGCGTGTGATACACCTGCATGATTCATTCTCCCGAGAAGTGATGCTGTCGGAACAGTATGCACTACAGTAAGGTGATCTTTTTGCTCTTGATTCCGAGTATCTCGTCCGAGAGGATTTGTGAGTTGGCGTGTGACCTCGCCTCATCTTGAGGAACATGTC

>DS499603:400998-401399

GTAAGCAAACACACTTCCACCAGCGATTGGGTATTCCTCCCAGCTGTCAAAGAGATGTCGATCTATGAACGGTATCGAATGCGATCCTAATAGTCGACAGGGGAATCCTGCACGGTAATGCTCTCCAGAGTCCAGATGGTCTACTTCCATGCCACTCCGTATCCATCACCCCATACTTTACTGCATGCTTCGCCTATTTACCTCGTTTCTAATTGGTCTCGCACAATTAGCTGCCGTTACCTAACAGCCTGGCGTCAGGCCACTTTGCCTACCAAATCCCGGCGCGAGGATTTTTCGGATGGGAGGCCCGGTTATTTCTCGGGCCCGAGCCCCAGTGCACAGAAAGATTCTGAATTGGCTTCAGAAGCCGAAAATCGCGCTGTGCCACAGTGGCCGTCGTC

>DS499603:406405-406806

TGGGTATCACTCTCAGTAGGTAACAGTAATATAACAGAGATCTCCTTGAACATAGGAGTCGAGGAGTCTGTGGACTACCAATCAGGGGAGGGCAGGGTTGGATCATCCAAGGCCAGTCAGAAGCTGTGGAGGCCATATCCACACCCAAAATGCCGTCTGAATGGTCCGCGACGATCATCTCCCGCGTCCCAGTCGGCCGATCCATTCCCCATCCGAGGTGCCAGGATCACCCCCTTTCGTCTTGGTCTTCCCACCCATTCGAATGCCACATCCAATATTCTTGCAGATAGTCGTTTTTGACTTTGCAAGTGCTCACACCATGTCCCTCATGTCCCTCTAGTCGTATCTGGCCCGTGGTACCTGTTCCCTGGTCCGACCCGATGCAGATAACTCCATTTGTC

>DS499603:409941-410342

CACAGGGCGCAGCGCAGTGACTGCGTGGCGATACATCACTACAGGTACGGATACAGGTACAGAGGACTGTAGGCTGTACAATCAAATCAGCGAGTAAAACATGGAATGACGACGTCCACGGGTCGATTCGGATGAGGTTGTGCCAATCACAATATCACCCATTCGGATCCACCCAGCCCACTGTGTCAGGGTCAGTTCTTTCAGTTGGTGATGGCGACGATGATGGCGATGATGATGATGTACTACAGTAATGCTTGCACCATACAATAGCCAAGTGAATACACTACATCATAATCAATCTATCCGGCTACTAGGACTAGTGTCTTGTCTCCAAGTTTCCCTACTTGTAGTGATGGCACAGGAGCGACTACTGACTCGTAGAGTATTTTCCAGAACCAAGA

>DS499603:427705-428106

AGTTCAGATCAACAACAGTGTCGCAGAAGGACCACAGTCACAGCCACTCATTATGACACACGTGGCTTGTTTGAAAATACCTCACTCAGTATCATGCATCCTATCCACTACGGAATAATCTCTACTCTATCATTAGGCTGTCAACCCAGAAAGACAGTGTTTCTGCCTGATTCTTTGCTCCAAGGCAAGGGGCTATGTGGCTCGTTAAGGCTGAACCCGAACCACTCCGCTGATACACTACTGTGGCTAGTAAGAGTCCAGGGGTATCCGATTGGGTGCGACTCGTGAGATCCACACACCAGCCTTGATGTGGCGTCGAAGCGGAGGGACTGTGGACGTTGTCGATTGTCAGGGGTTTGGTTCCTTGTACGGAAGTCGTGCTGGTGGACGAGTCATATGTT

>DS499603:496481-496882

AGCAATATAGATTCAAGAACTTTACCATCAGCTTTTGCTGCTTGTGTCCCGTGATGTTTCGACTCCCTCAAGAATAGCCGATGTATAAAAATTTAGTAAACTTTTCATGGGCATAACAACACCCCATTTCCCGTCGTGCCCGGCCCGGACTCCGTACCCACAAGCACAACTCAGCTACTCGTCTCGTTTACTGTCACATGTCTCAATCTCGTACGATTTTCAGTCGGTTCCTTTTTCCGATCAAGGTATTGTCGGTGAGCAGTGAACAATGACACGATAACCGTTGTTACTGGATAATTGTCCCATCTCTAAGAGGCCTTCTCCAAGCTTTGAATTCTCGCATTCTTTCCGTGATAACATTCCCGAGTCTTGGGAGCCAAAGCCGACTGTAAACGCTCGGC

>DS499603:527088-527489

TCGTCAGTGCAGCGAACACAGCGAACTAGAGATGTCATAGCTCTCTCACTGCAGGTCTCAGCCCTGTAGACTGAAAGCTACTGCTTCTATTGACAAGGCTGAAAGTCAATCCCATTCCCGTGGTGACGCTGATGAATCAAATCCTCTACCCGGGTGCCAACTGCCGACTGTTCCGCAATAGGACAAAGAAACCTGTGCTCGCCTGGAGATTGTCGGATGGGCGACGAAAGCTCAATCACATGATGGTTGCAGGTGCTATCTGTAGGGTGATCTACTGGTTTGTTGTGCTTGTCGATCATGTCCACTTCTTTTGACCCTTCCCACTGTTGGCGCTACTGCCGGAGCCAAAGCCAATCCCAACGTCTATTGAATAATGAGAAAGGAAATAGACTCAAACATTT

>DS499603:653946-654347

CATTAGAATGAAGCGTTACTAGTGGGCCATTTTTGGTAAGCAGAACTGGCGATGCGGGATGAACCGAACGCGAGGTTAAGGTGCCGGAATGCACGCTCATCAGACACCACAAAAGGTGTTAGTTCATCTAGACAGCCCGACGGTGGCCATGGAAGTCGGAATCCGCTAAGGAGTGTGTAACAACTCACGGGCCGAATGAACTAGCCCTGAAAATGGATGGCGCTCAAGCGTGCTACCCATACCTCGCCGTCGGGGTAGAAACGACGCCCCGACGAGTAGGCAGGCGTGGGGGTCCGTGACGAAGCCTTGGGAGTGATCCCGGGTCGAACGGCCCCTAGTGCAGATCTTGGTGGTAGTAGCAAATACTCAAATGAGAACTTTGAGGACTGAAGTGGGGAAAG

>DS499604:139323-139724

TTTTGTCTACCCTGGGGGATTGTACAGTTCTATTAGCGATTGTTGTGCCTTCAATGGGGTTGTATATTGGCAGCCCATAGTGATCGGAGTAAAAAACAGATTGAGGATATTATCCAAGTCTCAACATTCTTACCCGATATGTCTGCACTACATGGGAAGATCACGGATGCAATCGTCAGCACTCTATCCTGTGGCAAATCTCCTCTGTCTTATCTCCGTGATCAATTTAAGTATCGGACCACGGATGGAAACTAATGACGTATGGCAAGCGCACCTTGATATCTGACGGATGAGCTGATTCTTCTGGAATATTATGTAGCCTCATTTCTGTGCTTCCGGTAGCCCCATTATATGCAGACTGTCACCGCTTAGCATCCGAGGCCTGTGGTGACAAGGATATC

>DS499604:187368-187769

CGTTGCCGCGTTGCAGAAGCCGAACATCTTGGTCTTGGTGGGTTCCTCGACATCCATTTTCCAGGGAATGTGCTAAGGAGGAGAGAAAGAATGCCTTCCCGTCGTTCACGAGAGAGCAGGCGCTGTTCATCCACGGCAAGTCCCACCCGATTGTGCAGCTGGCTCCCCATCCGCACCAGAAGACCAGTATCTTCTCCAACGGTACGCCGTAATCCCCTTCCTGGTCAAGAGAACAACGCAACTAACCAGCTAGTGGCCAACGTGGTCGACCCCGTCCGGCCGGAGACCTGGGTGTTCCACTATTGCCTGTCAATCTGGACAGCCGACGACGCCCCTGAAACCGCCGAGGAGCGCCGCGCCCTTTTCAAGCACTACATGAGCCAATACTGCGAGCCGTACAG

>DS499604:226577-226978

GATCTACGTGCTGCACATGAAAAGCACCTTCAAAAGCAGAAGCGATCTAGGCGGCAGATAGAAACTGCAGTGGGATTATCTATCCAGGAAGGGCAGGAGATCATTCAACGCAGGGATCAGGCTGCTGAAGCTATCCCAACTATCCCTCCAGAGCAGGTAGTAGATACAGAACAACGCCCTCAACGGGCACCCCCACGCTGCAGTGACTGCCATATTCTAGGCCATAGGCGATTGCAATGTCCGCAGCGCAAGAATAACTAGATTTAGTAATAAAATCATGTTTTAGGGGTTCAAAATAGCCTCCAATTTCGGCCGCGGCCAAATTCTATGGTATGGTGATCCGCTCGGTTGCGTGATCCGCTCGCTTACCGATTACGTTATATCGCTTGATACGATGAGTC

>DS499604:452536-452937

CGGCTTGCGTTGTTGGACCTAGACCTGCGAGTATAGTACTTCAATGCACGGAATGATCGGGTGTATCACTCGAACTTATTAAATCACCCGAGATTCAACTGCTTGATACATCCGCTCAGACAGCCCACGCCAAGATTCTTGGTTTCCCGCAACAAGATCATTATGGACGATCTTGGGCACCGCCCCTCTTGGCCTCCGCCGGCCCTTCCACCCTCACTGGCTATTCTGGTCTCGGCGCCAAGTACGTAGTGGTCCGCTTATGCTGATCCTCCCACTTCCGGATTATGTCTCTGTGATGGCACGGACATTGAGATAACGGGCATCCCGCATGGCCAGAAGCCCATTCAGGGACATTAAAAACTAATTCCCTTGGCATCACGCCACCCTACCGCCATGGTTCA

>DS499604:479716-480117

CCAGGGCACTTAGACGGGGGCTGCACCCGAAGCATCCTCTGCAAATTACAACGCGGACCCCGAAGGGGCCAGCTTTCAAATTTGAGCTCTTGCCGCTTCACTCGCCGTTACTGAGGCAATCCCTGTTGGTTTCTTTTCCTCCGCTTATTGATATGCTTAAGTTCAGCGGGTATCCCTACCTGATCCGAGGTCAACCTTAGAAAAATAAAGTTGGGTGTCGGCTGGCGCCGGCCGGGCCTACAGAGCAGGTGACAAAGCCCCATACGCTCGAGGACCGGACGCGGTGCCGCCGCTGCCTTTCGGGCCCGTCCCCCGGGAGAGGGGGACGGGGGCCCAACACACAAGCCGTGCTTGAGGGCAGCAATGACGCTCGGACAGGCATGCCCCCCGGAATACCAGGG

>DS499634:240-641

CACGTTCAACTAAGCAACAAGCGCTTCTTACATATTTAAAGTTTGAGAATAGGTTAAGGTTGTTTCAACCCCAAGGCCTCTAATCATTCGCTTTACCTCATAAAACTGATCCGCGTTACTGCTATCCTGAGGGAAACTTCGGCAGGAACCAGCTACTAGATGGTTCGATTAGTCTTTCGCCCCTATACCCAAATTTGACGATCGATTTGCACGTCAGAACCGCTGCGAGCCTCCACCAGAGTTTCCTCTGGCTTCGCCCTATTCAGGCATAGTTCACCATCTTTCGGGTCCCCACAGCTACGCTCGTACTCAAATCCATCCGAAGACATCAGGATCGGTCGATGGTGCGCCCCGCGAGGGGGCTCCCACCTCCGTTCGCTTTCACTGCGCGTACGGGTTTG

>DS499642:120-521

AGAGGTGAAATTCTTGGATTTGCTGAAGACTAACTACTGCGAAAGCATTCGCCAAGGATGTTTTCATTAATCAGGGAACGAAAGTTAGGGGATCGAAGACGATCAGATACCGTCGTAGTCTTAACCATAAACTATGCCGACTAGGGATCGGGCGGTGTTTCTATGATGACCCGCTCGGCACCTTACGAGAAATCAAAGTTTTTGGGTTCTGGGGGGAGTATGGTCGCAAGGCTGAAACTTAAAGAAATTGACGGAAGGGCACCACAAGGCGTGGAGCCTGCGGCTTAATTTGACTCAACACGGGGAAACTCACCAGGTCCAGACAAAATAAGGATTGACAGATTGAGAGCTCTTTCTTGATCTTTTGGATGGTGGTGCATGGCCGTTCTTAGTTGGTGGAG

>DS499643:619-1020

ACCCGCTCGGCACCTTACGAGAAATCAAAGTTTTTGGGTTCTGGGGGGAGTATGGTCGCAAGGCTGAAACTTAAAGAAATTGACGGAAGGGCACCACAAGGCGTGGAGCCTGCGGCTTAATTTGACTCAACACGGGGAAACTCACCAGGTCCAGACAAAATAAGGATTGACAGATTGAGAGCTCTTTCTTGATCTTTTGGATGGTGGTGCATGGCCGTTCTTAGTTGGTGGAGTGATTTGTCTGCTTAATTGCGATAACGAACGAGACCTCGGCCCTTAAATAGCCCGGTCCGCATTTGCGGGCCGCTGGCTTCTTAGGGGGACTATCGGCTCAAGCCGATGGAAGTGCGCGGCAATAACAGGTCTGTGATGCCCTTAGATGTTCTGGGCCGCACGCGCGC

>DS499646:634-1035

TCAAATTTGAGCTCTTGCCGCTTCACTCGCCGTTACTGAGGCAATCCCTGTTGGTTTCTTTTCCTCCGCTTATTGATATGCTTAAGTTCAGCGGGTATCCCTACCTGATCCGAGGTCAACCTTAGAAAAATAAAGTTGGGTGTCGGCTGGCGCCGGCCGGGCCTACAGAGCAGGTGACAAAGCCCCATACGCTCGAGGACCGGACGCGGTGCCGCCGCTGCCTTTCGGGCCCGTCCCCCGGGAGAGGGGGACGGGGGCCCAACACACAAGCCGTGCTTGAGGGCAGCAATGACGCTCGGACAGGCATGCCCCCCGGAATACCAGGGGGCGCAATGTGCGTTCAAAGACTCGATGATTCACTGAATTCTGCAATTCACATTACTTATCGCATTTCGCTGCGT
