# Supplementary material for: The Transcription Factor SomA Synchronously Regulates Biofilm Formation and Cell Wall Homeostasis in Aspergillus fumigatus
Source: mBio. 2020 Nov 10;11(6):e02329-20. doi: 10.1128/mBio.02329-20 (PMC7667024; doi:10.1128/mBio.02329-20)
Supplement: TABLE S5 [file mBio.02329-20-st005.doc]

**Table S5. S**trains used in this study

| **Strain** | **Genotype** | **Reference** |
| --- | --- | --- |
| ***Aspergillus*** | | |
| A1160 | Δ*ku80, pyrG* | FGSC |
| WT | *A1160, pyr4* | (1) |
| AN991 | *Wild type* | FGSC |
| AF293 | *Wild type* | (2) |
| AFc06 | *Clinical isolate* | (3) |
| AFc08 | *Clinical isolate* | (3) |
| ∆*uge3* | ∆*uge3::hph* | (2) |
| ∆*ptaB* | ∆*ku80, pyrG,* ∆*ptaB::pyr4* | (4) |
| ∆*stuA* | ∆*ku80, pyrG,* ∆*stuA::pyr4* | This study |
| ∆*medA* | ∆*ku80, pyrG,* ∆*medA::pyr4* | This study |
| *Tet-somA* | ∆*ku80, pyrG, pyr4, tet(p)::somA::ptrA* | This study |
| SomA-Flag | ∆*ku80, pyrG, pyr4, somA::5×FLAG::hph* | This study |
| **Yeast** | | |
| *Candida albicans* ATCC10231 | *Wild type* | (5) |
| *Cryptococcus neoformans* H99 | *Wild type* | (5) |

References

1. Jiang H, Shen Y, Liu W, Lu L. 2014. Deletion of the putative stretch-activated ion channel Mid1 is hypervirulent in Aspergillus fumigatus. Fungal Genet Biol 62:62-70.

2. Gravelat FN, Beauvais A, Liu H, Lee MJ, Snarr BD, Chen D, Xu W, Kravtsov I, Hoareau CM, Vanier G, Urb M, Campoli P, Al Abdallah Q, Lehoux M, Chabot JC, Ouimet MC, Baptista SD, Fritz JH, Nierman WC, Latge JP, Mitchell AP, Filler SG, Fontaine T, Sheppard DC. 2013. Aspergillus galactosaminogalactan mediates adherence to host constituents and conceals hyphal beta-glucan from the immune system. PLoS Pathog 9:e1003575.

3. Li Y, Zhang Y, Zhang C, Wang H, Wei X, Chen P, Lu L. 2020. Mitochondrial dysfunctions trigger the calcium signaling-dependent fungal multidrug resistance. Proc Natl Acad Sci U S A 117:1711-1721.

4. Zhang S, Chen Y, Ma Z, Chen Q, Ostapska H, Gravelat FN, Lu L, Sheppard DC. 2018. PtaB, a lim-domain binding protein in Aspergillus fumigatus regulates biofilm formation and conidiation through distinct pathways. Cell Microbiol 20:e12799.

5. Li X, Li Y, Wang R, Wang Q, Lu L. 2019. Toxoflavin Produced by Burkholderia gladioli from Lycoris aurea Is a New Broad-Spectrum Fungicide. Appl Environ Microbiol 85:e00106-19.
