# Supplementary material for: The Transcription Factor SomA Synchronously Regulates Biofilm Formation and Cell Wall Homeostasis in Aspergillus fumigatus
Source: mBio. 2020 Nov 10;11(6):e02329-20. doi: 10.1128/mBio.02329-20 (PMC7667024; doi:10.1128/mBio.02329-20)
Supplement: TABLE S6 [file mBio.02329-20-st006.docx]

**Table S6.** **Primers used in this study**

| **Name/Purpose** | **Sequence (5’ to 3’)** |
| --- | --- |
| ***Tet-somA* construction and confirmation** | |
| Tet-somA P1 | CCGTTGTCATCCTTGATAG |
| Tet-somA P2 | GTCAAACCACAGTGGGAAATGG |
| Tet-somA P3 | GACAGTATAATACAAACAAAGATGCAAGAATCCGTGTTACAATTTCCTGTG |
| Tet-somA P4 | CCGCTTGAGCAGACATCACC ATGGCGACGGCAATTTCG |
| Tet-somA P5 | GGCATAGGCACCAGATCAGTCT |
| Tet-somA P6 | GATAGTCCTGGAGAGCATGG |
| Tet-ptrA F | TCTTGCATCTTTGTTTGTATTATACTGTC |
| Tet-ptrA R | GGTGATGTCTGCTCAAGCGG |
| Tet-somA SF | CTATCTTCTTGCGCCTCT |
| Tet-somA SR | TAGACAGGTGGAGAAGGC |
| Tet-ptrA down | AAGCACGCCTCCCTGTTTA |
| Tet-ptrA up | ACAACGCCAAGTCATTCCG |
| **Genes deletion and confirmation** | |
| StuA P1 | GAGCCTCGTCTTCCACTTCC |
| StuA P2 | CGTATCATCCCTTCCGTTTG |
| StuA P3 | CGATTAAGTTGGGTAACGCCAGTAGCTGTCGCTCACCTGATT |
| StuA P4 | ATAAGTAGCCAGTTCCCGAAAGCCGTGGGAACGAAAGCCTGTG |
| StuA P5 | TTCGGCTTGCCAATAATAAC |
| StuA P6 | CATTAGCCCTGCGCCTGTAT |
| StuA SF | TCCTCTGGACCGAGCACATA |
| StuA SR | GAAGTTTCCGTCTCCGATTGT |
| MedA P1 | CCCTTTCGGCTTCATCTGCG |
| MedA P2 | GGACAGGTGTCTCGGTTGAA |
| MedA P3 | CGATTAAGTTGGGTAACGCCAGAAATCCAGCGACTCGTCTA |
| MedA P4 | ATAAGTAGCCAGTTCCCGAAAGCTGGTTGTTTCGGTGCTGTTA |
| MedA P5 | TGTGATGTACGCTACGAGTGC |
| MedA P6 | TGTTGGCCCTCTGAATGGTG |
| MedA SF | TATGTGACCGCTACCTCGCTCGTT |
| MedA SR | GGTCGCACGGCCATCTTCAT |
| Pyr4F | TGGCGTTACCCAACTTAATCG |
| Pyr4R | GCTTTCGGGAACTGGCTACTTAT |
| cPyr4R | GGTCCAAGTGGAAGTAGGTAGTGAC |
| Cpyr4F | GGAATAAGAAATGGCGGAGGAT |
| Hph SF | AGATCATGGTTGACCGGTGCC |
| Hph SR | CGGAGCATTCACTAGGCAACCAT |
| Chph R | GCCGTCAACCAAGCTCTGATA |
| Chph F | TCTTGGCTCCACGCGACTA |
| **SomA-FLAG construction and confirmation** | |
| SomAflag P1 | GGTAACCATGCTCTCCAGGAC |
| SomAflag P2 | CCTGATGATGGCCCGTCAAGAG |
| SomAflag P3 | CATTCCCGGGGATCCCTCGATAAGCCATCTCCGGCGCCGGTCTCAA |
| SomAflag P4 | ATAAGTAGCCAGTTCCCGAAAGCTAAATGAGTCCGTCGGTTC |
| SomAflag P5 | GTGCATCGAGGACGAAGTTG |
| SomAflag P6 | GCAGTAGTCGCTCAGAGAAC |
| Flag SF | CTCGAGGGATCCCCGGGAATG |
| Flag-hph SR | GGCACCGGTCAACCATGATCTGACATTCCTTTTACCCGGGCTA |
| Hph-flag F | TAGCCCGGGTAAAAGGAATGTCAGATCATGGTTGACCGGTGCC |
| **RT-qPCR** | |
| RTtub F | TTCCGTCCCGACAACTTCGT |
| RTtub R | TCACAGCCTTCAGCCTCACG |
| RTmedA F | CGGCAAGACCAGCTAATCCG |
| RTmedA R | ATTGCGAGTGCCCTAACCCA |
| RTstuA F | ATGGTCGTGCAAGAATCGCA |
| RTstuA R | GGGAATGGAGGTCTGCATGG |
| RTgtb3 F | CACTCCTCCGTGGACTGCTT |
| RTgtb3 R | ACGGATGAAACCGCCTTGGA |
| RTega3 F | ACGACAAGTCCACCATCGCA |
| RTega3 R | GCCCAGATCCGAGTCCTTGA |
| RTsph3 F | AGAATGTGCGGCTGCTAGGC |
| RTsph3 R | CAGCTAGAGCCGGGTTGGAA |
| RTuge3 F | GCTGTTAGCCTCCCAGTACC |
| RTuge3 R | GGACTTGGTCGTACCCCAT |
| RTagd3 F | ACGCGGACGTCTTCAAGGAG |
| RTagd3 R | GTTGTGCAGACCGGTGATGG |
| RTchsA F | GGCGGGAAGGTTGACGTTGA |
| RTchsA R | AAACACCCAAGCGAGCACGA |
| RTchsB F | TGGTCAGTGGCTGTGTTGGC |
| RTchsB R | CACCATACTGCGCCGTCGAT |
| RTchsC F | CGGCTGGGAAAGAAGGGCAT |
| RTchsC R | CCGCCTCGGGAACGTCTGTC |
| RTchsD F | GGACAAAGCCGAGCAATGCG |
| RTchsD R | CGTTTCCGAGCGCCTACCAT |
| RTchsE F | CCAACGGCCCGACTCTCATC |
| RTchsE R | CGCCGAGACGTGTAAAGGCA |
| RTchsF F | ACCAGGTGGGCACAGGAAGA |
| RTchsF R | TCCCTGCCTCTGTGCTCTGT |
| RTchsG F | GGGCCGTTTGATGACCCTCAG |
| RTchsG R | CGACACGTCCAGGGACACCAA |
| RTcsmB F | CGGTGGCCTGAACTCGAGAC |
| RTcsmB R | AGACAGCTGCGGATCGCTTC |
| RTfks1 F | CCGCACGACGACTACTACGG |
| RTfks1 R | AACGTTCATCACCGCGACCA |
| RTchs7 F | GAAGGTGCTGCGTCCTTCGT |
| RTchs7 R | GGGAACCACACCGGCATCAA |
| RTchs3 F | TCCTCCAGTTCCGCCAGTCT |
| RTchs3 R | TGGAAACGGCCCATTGCTGT |
| **SomA recombination and confirmation** | |
| pet30-SomA F | GAAGGAGATATACATATGATGAATCAGATGAATGTGACGGGG |
| pet30-SomA R | CTCGAGTGCGGCCGCAAGCTTTAAGCCATCTCCGGCGCCGGTCTCAA |
| Pet30 F | GGAATGGTGCATGCAAGGAGA |
| Pet30 R | GCACTAAATCGGAACCCT |
| **EMSA** | |
| Cy5labbled | Cy5-AGCACGTGGTCGAAAG |
| EMSA agd3 F | AGCACGTGGTCGAAAGCACGTTACCAAACAATACCCG |
| EMSA agd3 R | AGCACGTGGTCGAAAGGTATGTCATCCGGACTGAGG |
| agd3control F | AGCACGTGGTCGAAAGGCTACGATTCAGACTGCAGC |
| agd3control R | AGCACGTGGTCGAAAGCCCGAGATAGTTGGTATTTCCG |
| agd3mut F1 | CGAGGTCGTCAGCCCAACAGTAGA |
| agd3mut R1 | GTATGTACCCATGGGTGCTGTATGTATGTATACATG |
| agd3mut F2 | CATGTATACATACATACAGcaccCATGGGTACATAC |
| agd3mut R2 | ACCATCACCAGTCACGGATGGT |
| agd3 fusion F | GCTCTTATGTATGCATGTCTCC |
| agd3 fusion R | ACGTCTGGTTGACAGGCATGCA |
| EMSA ega3 F2 | AGCACGTGGTCGAAAGCGCTAAAGCTTAGCGTGGATCG |
| EMSA ega3 R2 | AGCACGTGGTCGAAAGCCTACTATTGAATGGCGTTGA |
| EMSA ega3 F1 | AGCACGTGGTCGAAAGACCAGGGCAAGTCGTGGATT |
| EMSA ega3 R1 | AGCACGTGGTCGAAAGTAAGACATTCCCAAGCATCCGG |
| EMSA sph3 F1 | AGCACGTGGTCGAAAGAGTCTGCCAGATCTCAGGCTGA |
| EMSA sph3 R1 | AGCACGTGGTCGAAAGAGTAGTCAGGCTTGGCATGAG |
| EMSA sph3 F2 | AGCACGTGGTCGAAAGGATGGCAACTACACGCGTGAGA |
| EMSA sph3 R2 | AGCACGTGGTCGAAAGACGTCGGTCAACTCCTGCAGA |
